# Targeted strategy by curcumin and tideglusib biomimetic nano-systems alleviates oxidative stress and inflammation under ischemic stroke

**DOI:** 10.1080/10717544.2025.2585599

**Published:** 2025-11-25

**Authors:** Jiajia Li, Yiliang Yang, Meng Lin, Yitian Du, Yiwei Peng, Yu Zhou, Datong Gao, Yanxia Zhou, Xinru Li, Xianrong Qi

**Affiliations:** a Beijing Key Laboratory of Molecular Pharmaceutics and New Drug Delivery System, School of Pharmaceutical Sciences, Peking University, Beijing, PR China

**Keywords:** Ischemic stroke, biomimetic nano-system, platelet membrane, neuroprotection and function repairment, tideglusib, curcumin

## Abstract

Ischemic stroke represents one of the leading causes of disability and death worldwide. Neuroprotection aimed at mitigating oxidative stress and inflammation is crucial for improving the prognosis of patients. However, the inadequate accumulation of drugs at the ischemic site significantly restricts their clinical efficacy. We found that platelet membrane (PLTM)-biomimetic nanosystems loaded with curcumin (Cur@PLTM) and tideglusib (Tid@PLTM) actively targeted the ischemic brain and facilitated transcytosis into the ischemic parenchyma via caveolin-dependent transcytosis, mimicking the recruitment of platelets in damaged cerebral vessels. This represented the first application of tideglusib nanoformulations in treating ischemic stroke, further demonstrating that the therapeutic effects were associated with M2 microglia regulation. Additionally, Cur@PLTM and Tid@PLTM synergistically scavenged reactive oxygen species (ROS) and promoted the secretion of neuroprotective cytokines via redox and cellular regulatory mechanisms to mitigate ischemia/reperfusion (I/R) injury. Overall, this platelet membrane-biomimetic nanosystem offers a prospective strategy for targeted brain delivery and combined treatment through antioxidative and anti-inflammatory approaches against ischemic stroke.

## ARTICLE HIGHLIGHTS

Tid nanoformulations treat ischemic stroke and regulate M2-type microglia.

Both Cur and Tid in PLTM-NPs synergistically regulate oxidative stress and inflammation.

Tids have strong potential to scavenge ROS in microglia and brain endothelial cells.

Caveolin-dependent transcytosis of PLTM-NPs facilitates their ability to cross the BBB.

## Graphical abstract

**Figure:**
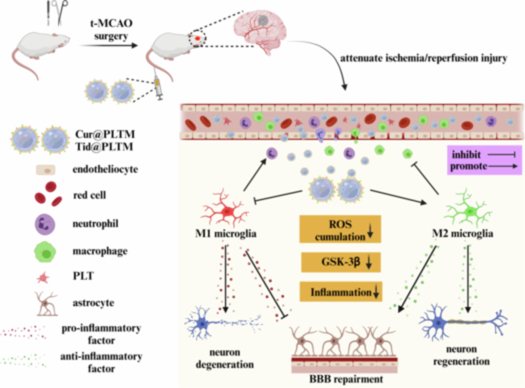

## 1 Introductions

Stroke is the second leading cause of death with the highest disability rate in the world (Murphy et al. 2020). Among all stroke types, the incidence of ischemic stroke accounts for more than 80%. Most developing countries have high stroke mortality rates and disease burdens (Donkor 2018). From 1990–2019, the absolute incidence rate and mortality of ischemic stroke in China doubled, and the relative incidence rate increased by approximately 70% (Ma et al. 2021). Thrombolysis, which restores blood flow to the ischemic site, is the main clinical treatment for ischemic stroke. The administration of recombinant tissue plasminogen activator (r-tPA) within 4.5 h after stroke onset is the most effective method for preventing ischemic penumbra injury (Patel et al. 2020). Unfortunately, studies have shown that after vascular blood flow, the influx of O_2_ further accelerates reactive oxygen species (ROS) generation and the inflammatory response, thus aggravating brain injury, which is called ischemia/reperfusion (I/R) injury (Jurcau and Ardelean 2021).

Pathological processes that arise during ischemia, such as excitotoxicity, ion imbalance, oxidative and nitrative stress, inflammation, blood‒brain barrier (BBB) breakdown and cell death, are the basis of injury after reperfusion (Doyle et al. 2008; Fisher and Savitz 2022). After blood reflows, brain cells try to reestablish a normal ion balance. The activity of the Na^+^/Ca^2+^ exchanger promotes the overload of intracellular Ca^2+^ and further damages mitochondria, leading to signal transduction resulting in cell death and the generation of ROS. O_2_ is the key contributor to the increase in ROS after reperfusion. Disruption of the mitochondrial electron transport chain results in the accumulation of free radicals during oxidative phosphorylation. In addition, NADPH oxidase, xanthine oxidase and nitric oxide synthase produce more free radicals in the presence of O_2_ (Jurcau and Ardelean 2021). Free radicals attack DNA, aggravate mitochondrial dysfunction and lipid peroxidation, destroy protein structure, and eventually cause irreversible damage and death of brain cells (Rodriguez-Vargas et al. 2012; Bachi et al. 2013; Sun et al. 2018; Su et al. 2019). ROS strongly activate microglia to the M1 phenotype (Jurcau and Simion 2022), activate the complement system (Alawieh et al. 2015) and facilitate the dysfunction of endothelial cells to upregulate the expression of P-selectin, PECAM-1, etc., promoting the infiltration of leukocytes and aggregation of platelets (PLTs), which hinder brain repair and account for the no-reflow phenomenon (Stoll and Nieswandt 2019; El Amki et al. 2020). At present, I/R injury is key for poor prognosis, but there is no feasible treatment in clinical practice. Therefore, studying the repair of I/R injury is highly clinically important.

In view of the harmful oxidative stress and inflammation associated with ischemic stroke, the combination of antioxidative and anti-inflammatory therapies has great potential for alleviating nerve damage and improving patient prognosis. Curcumin (Cur) can scavenge free radicals and activate nuclear factor erythroid 2-related factor 2 (Nrf2) (Yang et al. 2009; Gonzalez-Reyes et al. 2013), inhibit the mitogen-activated protein kinase (MAPK) and Nf-κB pathways of microglia/macrophages (Li et al. 2016; Tian et al. 2018), inhibit apoptosis and protect the BBB (Subedi and Gaire 2021). In the acute phase, M1-type microglia are the early key signals that cause the inflammatory cascade (Jin et al. 2013; Rajkovic et al. 2018; Jian et al. 2019). In contrast, M2-type microglia assist central nervous system (CNS) reconstruction in multiple ways (Qin et al. 2019), including clearing cell debris, releasing nutritional factors such as TGF-*β* and IL-10, promoting the proliferation and migration of neural precursor cells, facilitating axonal regeneration, protecting oligodendrocytes, etc. (Hu et al. 2015). Based on the powerful dual-edged nature of microglia, we focused on reversing microglia from the M1 phenotype during I/R to the M2 phenotype to promote CNS repair while eliminating inflammation. Tideglusib (Tid) is an irreversible, non-ATP competitive GSK-3β inhibitor. GSK-3β is highly expressed in the CNS and is immediately activated after the onset of ischemic stroke (Rana and Singh 2018), where it plays a pivotal role in microglial polarization (Luo 2012). Suppression of GSK-3β converts microglia to the M2 type (Xia et al. 2015).

The BBB blocks most drugs from entering the brain, which is a pivotal obstacle for adequate drug entry to the ischemic site, resulting in difficulties in achieving effective therapy for I/R injury (Tian et al. 2021; Li et al. 2021). In this context, targeted nanodelivery systems provide new perspectives for optimizing drug properties and lesion accumulation with better controllability and efficacy (He et al. 2021; Parvez et al. 2022). Poly(lactic-co-glycolic acid) (PLGA) has excellent biocompatibility and degradability and high encapsulation efficiency for hydrophobic small molecules, making it a reasonable carrier for brain delivery (Elmowafy et al. 2019). To further improve drug accumulation, biomimetic targeting is a promising approach. During ischemic stroke, platelets recruited and activated in damaged cerebral vessels play important regulatory roles in stroke progression (Rawish et al. 2020). Activated platelets highly express *P*-selectin, GPⅥ and integrins, which mediate the adhesion and subsequent recruitment of platelets (Gawaz et al. 2005; Nieswandt et al. 2011; Tomaiuolo et al. 2017). Therefore, we expected that coating activated platelet membranes (PLTMs) on the surface of PLGA nanoparticles could enable active targeting by equipping the natural performance of platelets (Peng et al. 2024).

**Scheme 1.**
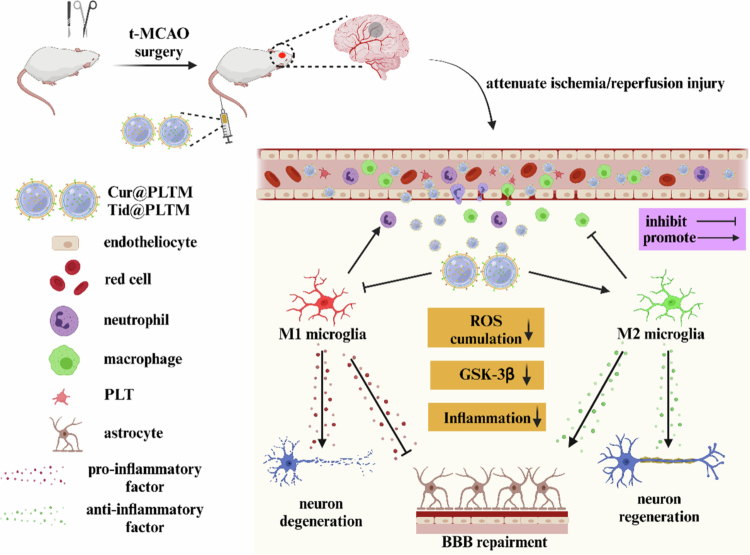
Schematic of neuroprotective effects of Cur@PLTM and Tid@PLTM in t-MCAO rats. Created with BioRender.com.

We first constructed PLTM biomimetic nanosystems of Cur and Tid to alleviate oxidative stress and inflammation after I/R injury. In the temporary middle cerebral artery occlusion (t-MCAO) model, Cur@PLTM and Tid@PLTM actively targeted the ischemic area, where Cur and Tid exhibited synergistic effects, including scavenging ROS, inhibiting GSK-3β and eliminating neuroinflammation (Scheme 1). Our study revealed that PLTM-NPs had prolonged circulation time, active targeting, enhanced antioxidation and the ability to induce M2-type microglia and were safe when applied to the brain. To our knowledge, Tid and its nanodelivery system are the first to be used to treat ischemic stroke by inducing the transition of microglia from the M1 type to the M2 type to relieve neuroinflammation. This study provides a new perspective for the combination of neuroprotective agents and biomimetic nanosystems aimed at promoting neural restoration and improving prognosis.

## 2 Materials and methods

### 2.1 Materials

Poly(lactic-co-glycolic acid) (PLGA, 50:50, intrinsic viscosity: 0.55−0.75 dL/g in hexafluoroisopropanol) was purchased from Lactel, USA. Curcumin (Cur) and tideglusib (Tid) were purchased from Selleck, USA. Span-80 and polyvinyl alcohol (PVA) were purchased from Aladdin, China. 1,2-Distearoyl-sn-glycero-3-phosphoethanolamine-*N*-methoxy (polyethylene glycol) (DSPE-mPEG; PEG has a molecular weight of 2000) was purchased from NOF, Japan. PMSF, RIPA, SDS‒PAGE loading buffer, Bradford kit, SDS‒PAGE gel preparation kit and Nissl staining solution were purchased from Beyotime, China. DMEM (high glucose), antibiotics (penicillin-streptomycin), trypsin, Hoechst 33342 and 4% paraformaldehyde (PFA) were purchased from Omacgene, China. Cell Counting Kit-8 (CCK-8) was purchased from Yeasen, China. DMEM (low glucose), 1,1ʹ-dioctadecyl-3,3,3ʹ,3ʹ-tetramethylindodicarbocyanine perchlorate (DiD) and amiloride hydrochloride were purchased from Meilunbio, China. AnaeroPack was purchased from Mitsubishi Gas Chemical Company, Japan. Mouse TNF-α/IL-6/IL-10 and human/mouse TGF-β1 uncoated ELISA kits were purchased from Ebioscience, USA. The CD206 polyclonal antibody and CD86 monoclonal antibody were purchased from Immunoway, China. FITC-conjugated anti-mouse CD86 antibody was purchased from Biolegend (USA). Goat anti-rabbit lgG H&L/Alexa Fluor 594 was purchased from Bioss, China. Rabbit anti-mouse GFAP/NeuN/Iba-1 monoclonal antibodies and CD31 polyclonal antibodies were purchased from Abcam, UK. The silicone-coated occlusion line was purchased from Beijing Belo Biotechnology Co., Ltd., China. 2,3,5-Triphenyltetrazole chloride (TTC) was purchased from Sigma-Aldrich, USA.

### 2.2 Cell lines and animals

BV2 (mouse microglial cell line) and bEnd.3 (mouse-derived brain microvascular endothelial cell line) cells were cultured in DMEM (containing 10% FBS and 1% penicillin‒streptomycin) at 37 °C with 5% CO_2_.

Male SD rats (250−280 g, *n* = 40) were purchased from Beijing Vital River Laboratory Animal Technology Co., Ltd., China, and fed under standard conditions. Male rats were selected to avoid possible effects of hormone fluctuations in the females. All animal experiments were performed according to the guidelines of the National Institutional Animal Care and Care and Use of Laboratory Animals of Peking University, with approval from the Animal Ethics Committee of Peking University Health Science Center (assigned number: LA2019188). The authors adhered to the ARRIVE guidelines.

### 2.3 Extraction of PLTM

The EP tube was pretreated with heparin sodium and sodium citrate. Whole blood was collected from the inferior orbital vein of SD rats (250−280 g, *n* = 2) and centrifuged at room temperature (RT) at 200 × *g* for 10  min. The supernatant was collected to obtain platelet-rich plasma (PRP). PRP was centrifuged at 200 × *g* for 5 min to further remove red cells. The supernatant was collected and centrifuged at 800 × *g* for 20 min to obtain platelets. The platelets were resuspended in PBS containing 0.5 μg/mL thrombin, placed in a shaker (THZ-C, Suzhou Peiying Experimental Equipment Co., Ltd., China) and incubated at 37 °C for 30 min to activate the platelets. The platelets were subsequently freeze-thawed repeatedly 6 times and centrifuged 2 times at 4 °C 15,000 rpm and 10 min to obtain the PLTM.

### 2.4 Preparation of PLGA-NPs and PLTM-NPs

PLGA with Cur or Tid (0%−10% w/w PLGA) was dissolved in 1 ml dichloromethane containing 0.01% (w/w) span-80 and added to 5 ml of 1.5% PVA solution with 0.4 mg/ml DSPE-mPEG. The O/W emulsion was obtained in an ice bath by ultrasonication (JY92-ⅡN, Ningbo Xinzhi Biotechnology Co., Ltd., China) at a power of 45%. The emulsion was then immediately evaporated at RT for 1 h to form drug-loaded PLGA-NPs. PLGA-NPs were centrifugally washed twice and dispersed in PBS. PLGA-NPs were mixed with PLTM and ultrasonicated for 4 min in an ice bath at a power of 15% to form drug-loaded PLTM-NPs.

### 2.5 Size and morphology

The particle size and zeta potential of the PLGA-NPs and PLTM-NPs were measured via dynamic light scattering using Zetasizer Nano-ZS (Malvern, UK) at 25 °C for 11 runs per sample. Transmission electron microscopy (TEM, JEM-1400, Leica, Germany) was used to characterize the morphology of the PLTM-NPs after they were dropped onto a carbon grid, negatively stained with uranyl acetate and air-dried.

### 2.6 Encapsulation efficacy

The encapsulation efficacy of Cur and Tid in the nanosystems was determined via UV spectrophotometry (UH5300 UV spectrophotometer, Hitachi, Japan). First, UV absorption standard curves of Cur and Tid dissolved in DMSO were prepared. UV absorption of Cur was measured at 422 nm at concentrations ranging from 0.5−8 μg/ml. The UV absorption of Tid was measured at 290 nm at concentrations ranging from 6−50 μg/ml. The UV absorption of Cur and Tid in Cur@PLGA and Tid@PLGA was measured after they were dispersed in DMSO. The encapsulation efficiency (EE%) was calculated as follows:

$$\text{EE}\%=\frac{C_{\mathrm{int}}∙V_{\mathrm{int}}}{M_{\mathrm{total}}}\times 100\%,$$

where C_int_ is the concentration of Cur or Tid in the PLGA-NPs. V_int_ and M_total_ represent the volume of PLGA-NPs and the total amount of each drug, respectively.

### 2.7 Protein contents of PLTM-NPs

RIPA buffer and PMSF were added to extract proteins from PLTs. The total protein from the PLTs was collected from the supernatant after centrifugation. The protein concentrations of the PLT, PLTM and PLTM-NPs were determined via a Bradford kit. SDS‒PAGE loading buffer was added to the samples, which were subsequently heated at 95 °C for 10 min. SDS-PAGE gel electrophoresis (Mini-Protean Cell-1658001 small protein gel electrophoresis system, Bio-Rad, USA) was performed. After electrophoresis, the gel was placed in boiling water for decolorization. Then, Coomassie blue solution was added, and the mixture was incubated for 20–30 min. After staining, deionized water was added to decolorize the gel, and photos were taken with a gel imaging system (Tanon-5200, Tanon, China).

### 2.8 Cytotoxicity

BV2 and bEnd.3 cells were seeded into 96-well plates. After incubation with a series of concentrations of Cur@PLTM (0–20 μM) or Tid@PLTM (0−30 μM) for 24 h or 48 h, the cells were incubated with CCK-8 solution. The absorbance was measured at 450 nm using microplate analyzer (iMark, Bio-Rad, USA). Cell viability was calculated as follows:

$$\text{Cell viability}\;\%=\frac{{\text{OD}}_{\text{i}}-{\text{OD}}_{\text{i}-\text{blank}}}{{\text{OD}}_{0}-{\text{OD}}_{0-\text{blank}}}\times 100\%,$$

OD_i_ represents the absorbance of each experimental group; OD_i-blank_ represents the absorbance of the corresponding blank medium; and OD_0_ and OD_0-blank_ represent the absorbances of the PBS group and its blank medium, respectively.

### 2.9 Hemolysis

Whole blood was collected from an SD rat (250−280 × *g*) and centrifuged at 1500 rpm for 5 min to separate red cells. The cells were diluted with PBS (1:50, v/v). Triton X-100 (2% w/w, positive control), Cur@PLTM (2.5−30 μM) or Tid@PLTM (10−40 μM) was added. All the samples were incubated at 37 °​​​​​C for 4 h and centrifuged at 1500  rpm for 5 min, after which the supernatants were observed for hemolysis.

### 2.10 Cell uptake and the mechanism

DiD was chosen as the fluorescent probe to prepare DiD-loaded DiD@PLGA and DiD@PLTM. BV2 cells were seeded in 12-well plates and then divided into two different groups: one was cultured normally, and the other was subjected to oxygen and glucose deprivation/reperfusion (OGD/R) using AnaeroPack to simulate oxidative stress. After 30 min of reperfusion, DiD@PLGA or DiD@PLTM (DiD: 10 μg/ml) was added to both groups, and the mixture was cultured for 4 h. After that, the fluorescence intensity of DiD was detected via flow cytometry (FACS Calibur, BD, USA). Confocal microscopy (TCS STED, Leica, Germany) was used to observe the uptake of BV2 and bEnd.3 cells subjected to OGD/R.

BV2 and bEnd.3 cells were seeded in 12-well plates to carry out OGD/R. At the beginning of reperfusion, chlorpromazine (20 μM), dynasore (25 μg/mL), amiloride (EIPA, 25 μM) or sodium azide (NaN_3_, 1 mg/ml) was added, and the mixture was pretreated for 30 min. After the inhibitors were removed, DiD@PLGA or DiD@PLTM (DiD: 10 μg/ml) was added for 4 h. Cell uptake was assayed by flow cytometry.

### 2.11 *In vitro* BBB model

BBB model *in vitro* was established (Yang et al. 2017) to investigate the trans-BBB capacity of PLGA-NPs and PLTM-NPs. bEnd.3 cells were seeded at 4 × 10^4^ (Patel et al. 2020)/well in transwell chambers (equipped with a 0.4 μm cutoff microporous membrane). The medium was changed every two days, and transendothelial electric resistance (TEER) was measured with a cell resistance meter (Millicell ERS-2, Millipore, USA). After 5 d, a formal experiment was performed when the TEER was constant, and a slightly excessive volume of medium was added to the transwell chamber such that there was no significant decrease in the liquid level, which was considered successful construction of the BBB model. The established BBB model was either cultured under normal conditions or pretreated with 10 μg/ml lipopolysaccharide (LPS) for 12 h to simulate an injured BBB and then cocultured with BV2 cells under normal conditions or pretreated with 1 μg/ml LPS for 24 h in a plate.

Coumarin 6 (C6) was used to indicate the translocation of PLGA-NPs and PLTM-NPs across the BBB. Thus, C6@PLGA and C6@PLTM were prepared. C6@PLGA or C6@PLTM (C6: 10 μg/ml) was added to the transwell chamber and incubated for 10 h. bEnd.3 and BV2 cells were fixed with 4% PFA and stained with iFluor™ 594-wheat germ agglutinin (WGA) and DAPI to label the cytomembrane and nucleus. Then, the slides were sealed with glycerin: PBS (9:1, v/v). Internalization of C6@PLGA and C6@PLTM in bEnd.3 and BV2 cells was observed via confocal microscopy.

The contents of C6@PLGA and C6@PLTM in each part were quantified to compare the efficiency of the nanoparticles across the BBB. After incubation for 10 h, the medium in the transwell (upper) chambers was collected, and bEnd.3 cells were treated with 1% Triton X-100 to collect C6@PLGA and C6@PLTM internalized by bEnd.3 cells, which were then lyophilized and redissolved with DMSO to detect UV absorption. Concentrations were calculated according to the UV absorption standard curve of C6.

### 2.12 t-MACO model

Male SD rats (250−280 g, *n* = 38 for all *in vivo* animal testing) were anesthetized with 2% isoflurane to minimize suffering. The common carotid artery (CCA), external carotid artery (ECA), internal carotid artery (ICA) and vagus nerve were exposed and separated at the incision area slightly left of the neck midline. The CCA was ligated temporarily, and 2 lines were inserted into the ECA, where the distal end was ligated and the proximal end was loosened. Coagulate the ECA branches between the two lines. The ICA was temporarily closed with a vascular clamp. The distal end of the ECA was coagulated. A small incision was made between the two lines, and the occlusion line (treated with 5000 U heparin sodium before use) was pushed from the ECA into the ICA through the ECA incision. After the ICA was reached, the line at the proximal end was fastened, and the clamp was removed. The occlusion line was pushed until a sensation of slight resistance was reached, indicating that the line had exceeded the proximal end of the anterior cerebral artery and blocked the blood flow of the middle cerebral artery. The line was fixed for 1.5 h and then removed. The residual ECA was ligated, and the line at the CCA was removed to start reperfusion. During the entire surgery, the rats were anesthetized, and the temperature was maintained at 37 °C. For the sham operation group, the CCA, ECA and ICA were separated and ligated in the same way without occlusion.

### 2.13 Anti-oxidative capacity

BV2 and bEnd.3 cells were seeded in 12-well plates. Oxidative stress was induced by OGD/R. After 30 min of reperfusion, free Cur, Cur@PLGA or Cur@PLTMs (Cur: 5 μM) were administered and incubated for 24 h. DCFH-DA was used to quantify intracellular ROS. DCFH-DA (10  μM) was added to BV2 and bEnd.3 cells, which were subsequently incubated at 37 °C for 20 min. The fluorescence intensity was measured via flow cytometry.

Similarly, the fluorescence of DCFH-DA was also observed via confocal microscopy. BV2 and bEnd.3 cells were seeded in confocal dishes and subjected to OGD/R. Then, the cells were incubated with 2.5 μM or 5 μM Cur@PLTM for 24 h. ROS were detected after DCFH-DA staining.

### 2.14 Detection of cytokines

BV2 cells were seeded in 6-well plates and incubated with 1 μg/ml LPS for 24 h to induce M1-type BV2 cells. Fresh media containing different Tid preparations were added after LPS treatment, and the anti-inflammatory effects of free Tid, Tid@PLGA and Tid@PLTM were investigated after 24 h of incubation. The medium of each group was collected and centrifuged. The levels of TNF-*α*, IL-6, IL-10 and TGF-β1 in the supernatant were analyzed via ELISA kits. IL-10 and TGF-β1 levels in the OGD/R model were analyzed via the same method.

### 2.15 CD86 and CD206 expression

LPS was used to induce M1-type BV2 cells. After that, fresh medium containing PBS, Tid@PLTM (20 μM) or 30 ng/ml IL-4 was added, and the mixture was incubated for 24 h. The cells were fixed with 4% PFA, permeabilized with 0.1% Triton X-100 and sealed with 2% BSA. A total of 100  μL of diluted CD206 antibody (primary antibody, 1:50, v/v) was added to each dish and incubated overnight at 4 °C. BV2 cells were then incubated in the dark with goat anti-rabbit IgG/Alexa Fluor 594 (1:100, v/v) at RT for 1 h. The nuclei were stained with DAPI. The expression of CD206 in each group was observed via confocal microscopy. CD86 expression was detected by flow cytometry after PBS or Tid@PLTM (20 μM) treatment for 24 h in LPS-induced M1-type BV2 cells.

### 2.16 Distribution and targeting of PLTM-NPs *in vivo*

t-MCAO rats (250–280 g, *n* = 6) were established. DiD-loaded DiD@PLGA or DiD@PLTM (DiD: 0.2 mg/kg) was administered by intravenous tail vein injection at 30 min after reperfusion, and anatomical analysis was performed after 24 h. The rats were euthanized via inhalation anesthesia with 5% isoflurane to carry out cardiac perfusion. A small opening in the right ventricle was made, and 50 mL of PBS was pushed into the left ventricle for cardiac perfusion to exclude blood interference. The main organs (heart, liver, spleen, lung, kidney) and brain were subsequently removed and placed in ice-cold PBS for imaging immediately (IVIS SPECTRUM, PerkinElmer, USA) *ex vivo*. The brain was immersed in 4% PFA to prepare coronal frozen slices (8 μm).

### 2.17 Analysis of intracerebral cell uptake

Frozen slices of the brain were subjected to immunofluorescence staining. The slices were fixed with 4% PFA and washed with PBS. The slices were immersed in 0.2% (w/w) Triton X-100 and then blocked with 5% BSA for 1.5 h. Rabbit anti-NeuN, anti-GFAP, anti-Iba-1 and anti-CD31 antibodies were diluted (1:200, v/v) with PBS containing 1% BSA. The antibodies were added to the slices, which were subsequently incubated at 4 °C overnight. The next day, goat anti-rabbit lgG H&L/Alexa Fluor 594 (1:500, v/v) was added, and the samples were incubated at 37 °C for 1 h in the dark and washed with PBS. The slices were sealed with fluorescence decay-resistant reagent including DAPI. The colocalization of DiD with astrocytes, microglia, neurons and endothelial cells (ECs) was observed via confocal microscopy.

### 2.18 Grouping and dosing *in vivo*

t-MCAO rats (250–280 g, *n* = 28) were randomly divided into 7 groups, with 4 rats in each group: control (G1, no treatment), free Cur (G2), free Tid (G3), Cur@PLTM (G4), Tid@PLTM (G5), Cur@PLGA + Tid@PLGA (G6), and Cur@PLTM + Tid@PLTM (G7). Cur and Tid (10 mg/kg) were administered via intravenous tail vein injection at 30 min after reperfusion. The sham group (250−280 g, *n* = 4) was set as the healthy comparison with no ischemia. Animals were euthanized via inhalation anesthesia with 5% isoflurane at 48 h after treatment.

### 2.19 TTC staining and infarct volume analysis

The brains were quickly removed on ice after cardiac perfusion and frozen for 1.5 h at −20 °C. A 2% triphenyltetrazolium chloride (TTC) solution in normal saline was prepared, and the frozen brain was sectioned coronally into 6 equal slices. TTC was immediately added to the slices, which were subsequently incubated at 37 °C for 20 min in the dark. The slices were reversed for another incubation. Slices were fixed with 4% PFA overnight, and the infarct volume (%) was calculated:

$$\text{Infarct volume}\;\%=\frac{{\text{V}}_{\inf}\;-{\;\text{V}}_{\text{edema}}}{{\text{V}}_{\text{total}\;}-{\;\text{V}}_{\text{edema}}}\times 100\%.$$

where V_inf_, V_edema_ and V_total_ were the infarct volume, edema volume, and total brain volume, respectively.

### 2.20 Neurological function score

After 48 h of treatment, the neurological function of the animals was tested before dissection. The motor-perception (mNSS) scale consists of motor, sensory (visual, tactile and proprioceptive) and reflex tests. Higher scores indicate more severe injury (1−6: mild; 7−12: moderate; 13−18: severe) (Shen et al. 2006).

### 2.21 Nissl staining

The brains were removed on ice quickly after cardiac perfusion and stored at -80 °C. Coronal frozen slices of the brain were made (20 μm). The slices were fixed with 4% PFA and washed with distilled water. Nissl staining solution was added, and the samples were incubated at RT for 15–20 min. The slices were subsequently washed according to the protocol and sealed. Images of the ischemic and normal cortices were taken via a panoramic scanner (Nano Zoomer, Hamamatsu, Japan).

### 2.22 CD86 and CD206 staining in ischemic cortex

The brain slices from the control and Tid@PLTM groups were fixed with 4% PFA, permeabilized with 0.3% Triton X-100 and sealed with 5% BSA. A total of 150 μL of diluted CD86 or CD206 antibody (primary antibody, 1:200, v/v) was added to each slice and incubated overnight at 4 °C. The slices were then incubated in the dark with goat anti-rabbit lgG/Alexa Fluor 594 (1:400, v/v) at 37 °C for 1 h and sealed with fluorescence decay-resistant reagent including DAPI. Images were captured via confocal microscopy.

### 2.23 H&E staining

Frozen slices of the heart, liver, spleen, lung, kidney and brain were stained with H&E dye. The slices were first fixed with 4% PFA for 15 min, washed with water and immersed in hematoxylin dye for 4 min. After washing and bluing, the slices were immersed in 95% alcohol for dehydration for 1 min and then dyed with an eosin solution for 15 s. The slices were finally dehydrated and sealed. Slices were observed under a pathological section panoramic scanner or microscope.

### 2.24 Statistical analysis and reproducibility.

Each experiment was independently repeated at least three times, unless mentioned otherwise. The experimental data are expressed as the means ± standard deviations (SDs) and were analyzed with GraphPad Prism 8. The significant differences among groups were analyzed by one-way analysis of variance (ANOVA) in IBM SPSS Statistics 26.0. *p* < 0.05 was considered statistically significant (*p < 0.1, **p < 0.05, ***p < 0.001).

## 3 Results

### 3.1 Characterizations of biomimetic nano-systems

**Figure 1.**
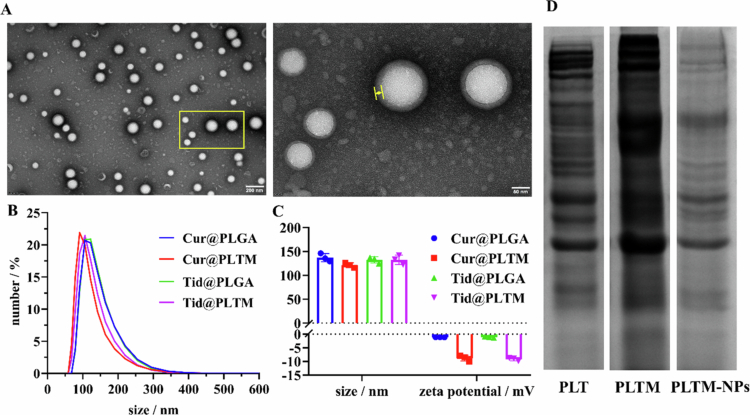
Characterizations of PLTM-NPs. (A) TEM images of PLTM-NPs. Scale: 200 nm (left) and 50 nm (right). (B) Hydrodynamic sizes of PLGA-NPs and PLTM-NPs. (C) Particle sizes and zeta potentials (*n* = 3). (D) Protein retention of platelet (PLT), PLTM and PLTM-NPs by SDS‒PAGE.

TEM revealed that the surface of PLGA-NPs was covered by a PTLM layer with a thickness of approximately 10−20 nm (Figure 1A). Statistics of size distributions (Figure S1) of 275 particles were calculated under different fields, and the average diameter of the PLTM-NPs was 100.34 nm, which increased by approximately 23.95 nm after the PLTM coating. The DLS results revealed that the mean diameter by number distribution of Cur@PLGA, Tid@PLGA, Cur@PLTM and Tid@PLTM was approximately 120−140 nm (Figure 1B, C), with a small PDI and good dispersibility, suggesting the wrapping of the hydration layer. The zeta potentials changed from −1.117 mV and −1.084 mV for Cur@PLGA and Tid@PLGA to −8.760 mV and −9.153 mV for Cur@PLTM and Tid@PLTM, respectively (Figure 1C**)**, indicating that the PLTM coating increased electronegativity. PLTM coating should protect the core nanoparticle from premature hydrolysis and biodegradation, as well as good biocompatibility. No obvious change in the size of PLTM-NPs was observed within five days at 4 °C (Figure S2). SDS‒PAGE revealed that the PLTM extracted by repeated freeze‒thaw cycles contained almost the same amount of protein as native platelets (PLTs) did (Figure 1D), and rich protein was also retained after ultrasonication. The encapsulation efficacies of Cur and Tid were approximately 94% and 89%, respectively. To characterize the release behavior of PLTM-NPs, the cumulative release of Cur from Cur@PLTM was monitored at 37 °C in neutral (pH = 7.4) or acidic PBS (pH = 6.0 with 0.5 mM H_2_O_2_) to simulate normal or ischemic conditions (Figure S3). No obvious release difference was observed within 36 h, whereas the cumulative release of Cur under ischemic conditions doubled within 100 h, which verified the protection of the PLTM layer and accelerated release of core PLGA-NPs in the ischemic brain by faster ester hydrolysis. These results indicated that PLTM biomimetic nanosystems were successfully constructed with a suitable particle size, good dispersibility and high encapsulation efficacy, which enabled their penetration across the BBB and active targeting by equipping the natural performance of platelets (Peng et al. 2024).

### 3.2 *In vitro* safety and cell uptake

**Figure 2.**
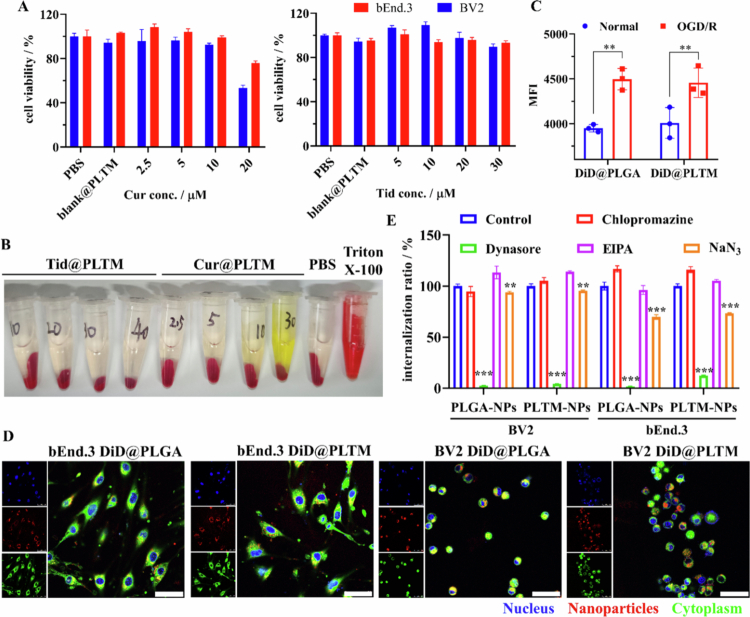
Safety and cell uptake of PLTM-NPs. (A) Cytotoxicity of Cur@PLTMs and Tid@PLTMs in BV2 and bEnd.3 cells at 24  h (*n* = 3). (B) Hemolytic assay. (C) Uptake by BV2 cells under normal or oxidative stress (OGD/R) conditions (*n* = 3). MFI: mean fluorescence intensity. (D) Confocal images of BV2 and bEnd.3 cell uptake under OGD/R conditions. Scale: 50 μm. (E) Effects of different inhibitors on BV2 and bEnd.3 cell endocytosis by PLGA-NPs and PLTM-NPs (*n* = 3). **p < 0.05, ***p < 0.001.

BV2 and bEnd.3 cells were used to investigate the cytotoxicity and appropriate dose of PLTM-NPs. In these two cell lines, the viability of the cells treated with blank@PLTM at 24 h **(**Figure 2A) and 48 h (Figure S4) was similar to that of the PBS-treated cells, indicating that the vehicle had no cytotoxic effect. Cur@PLTM and Tid@PLTM caused no significant damage to cells at doses of 0−10 μM and 0−20 μM, respectively. At higher doses, the cells showed some damage. In addition, we observed that BV2 cells were more susceptible to nanoparticles than bEnd.3 cells were, which implied that nanoparticles may not damage brain capillaries. The hemolysis test revealed that no hemolysis occurred when the red cells were treated with 0−30 μM Cur@PLTM or 0−40 μM Tid@PLTM for 4 h (Figure 2B), and the morphology of the red cells did not change at high concentrations of Cur@PLTM (30 μM) or Tid@PLTM (40 μM).

Next, DiD was used as a fluorescent probe to investigate cell uptake and mechanism. No significant difference was observed in the uptake of DiD-loaded PLGA-NPs and PLTM-NPs under normal or oxidative stress conditions, but oxidative stress clearly increased the uptake of both types of nanoparticles (Figure 2C), which is favorable for promoting drug internalization. The confocal microscopy results revealed that both PLGA-NPs and PLTM-NPs were distributed mainly in the cytoplasm (Figure 2D) after being effectively internalized by BV2 and bEnd.3 cells under oxidative stress. We further explored the uptake mechanism. Chlorpromazine inhibits clathrin-dependent endocytosis through anchoring clathrin and adaptor protein 2 complexes to endosomes, thereby preventing the assembly of coated pits at the inner surface of the plasma membrane. EIPA inhibits macropinocytosis by lowering the pH in the submembranous region at the site of macropinocytosis to impair actin polymerization (CHANG et al. 2014). Dynasore is a noncompetitive inhibitor of dynamin GTPase activity. Dynamin is a 100-kd GTPase that assists in endocytosis by helping form endocytic vesicles and pinching off newly formed vesicles from the plasma membrane (Liu et al. 2006; Roux et al. 2006; Abban et al. 2008). BV2 and bEnd.3 cells were pretreated with chlorpromazine, dynasore, EIPA or NaN_3_ for half an hour prior to the administration of PLGA-NPs or PLTM-NPs. In these two cell lines, chlorpromazine and EIPA did not affect the uptake of PLGA-NPs or PLTM-NPs, whereas dynasore and NaN_3_ pretreatment resulted in significant reductions (Figure 2E). These findings suggested that the mechanism of the internalization of PLGA-NPs and PLTM-NPs by BV2 and bEnd.3 cells was predominantly caveolin-dependent endocytosis, which was energy dependent. In this study, we demonstrated that PLTM-NPs did not significantly affect cell viability and did not cause hemolysis; thus, PLTM-NPs have good safety in cells and blood circulation. PLTM-NPs can be effectively internalized by BV2 and bEnd.3 cells via caveolin-dependent endocytosis, and oxidative stress conditions might facilitate drug entry into these cells.

### 3.3 Anti-oxidation and anti-inflammation of PLTM-NPs

**Figure 3.**
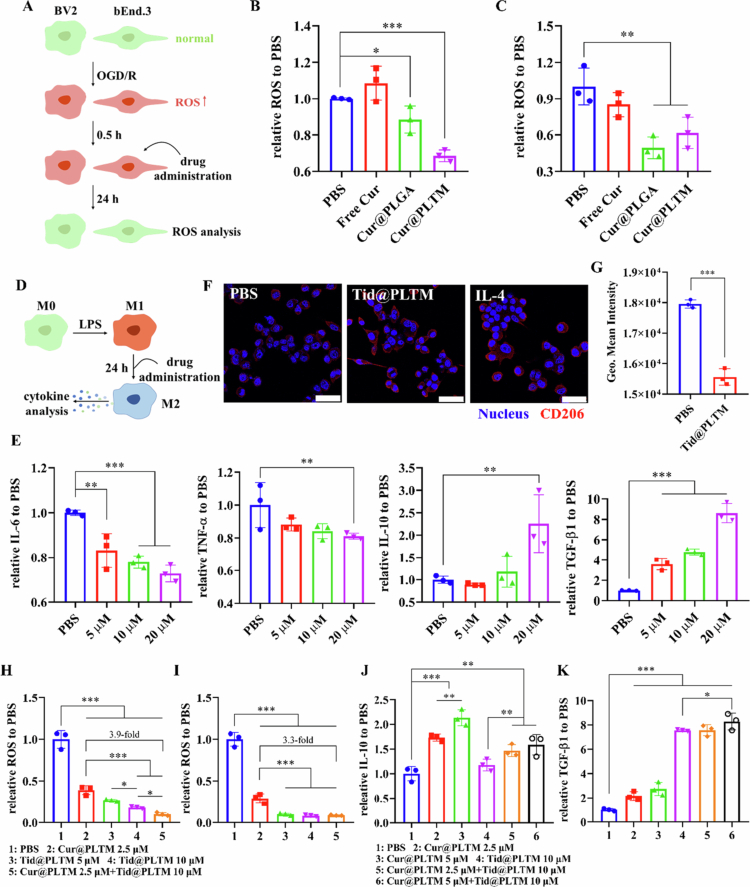
Pharmacological evaluations of PLTM-NPs. (A) Schematic of Cur@PLTM treatment. (B, C) ROS scavenging in BV2 (B) and bEnd.3 (C) cells treated with free Cur, Cur@PLGA or Cur@PLTMs (*n* = 3, the Cur concentration was 5  μM). (D) Schematic of Tid@PLTM treatment of BV2 cells. (E) Cytokine levels in BV2 cells after treatment with different concentrations of Tid@PLTM (*n* = 3). (F) Immunofluorescence of CD206 expression induced by Tid@PLTM (20 μM). IL-4 (30 ng/ml) was used as a positive control to induce M2-type microglia. Scale: 50 μm. (G) CD86 expression in BV2 cells after PBS or Tid@PLTM (20  μM) treatment for 24  h, as determined by flow cytometry (*n* = 3). (H, I) ROS scavenging in BV2 (H) and bEnd.3 (I) cells after Cur@PLTM or/and Tid@PLTM treatment (*n* = 3). (J, K) Anti-inflammatory IL-10 (J) and TGF-β1 (K) expression after Cur@PLTM or/and Tid@PLTM treatment (*n* = 3). *p < 0.1, **p < 0.05, ***p < 0.001.

Cur has multiple antioxidative functions. Oxidative stress in BV2 and bEnd.3 cells was induced by OGD/R, after which the degree of ROS scavenging by PLTM-NPs was investigated (Figure 3A). DCFH-DA was used as ROS probe, which can be hydrolyzed by esterase and react with ROS intracellularly to generate DCF with fluorescence proportional to the ROS level. We first compared the ROS scavenging efficiency of free Cur, Cur@PLGA and Cur@PLTM. At the same concentration of Cur, the antioxidation effects of Cur@PLGA and Cur@PLTM were greater than those of free Cur (Figure 3B,C). Cur@PLTM had the best ROS scavenging effect on BV2 cells, indicating that PLTM-NPs could optimize the antioxidative effect of Cur. Similar results were observed by confocal imaging, where ROS decreased after treatment with Cur@PLTM (Figure S5). These results demonstrated that Cur@PLTM optimized the antioxidative capacity of Cur, which is associated with activation of the Nrf2 pathway (Ashrafizadeh et al. 2020).

After ischemia onset, microglia are rapidly activated to the M1 type along with GSK-3β overactivation and thus secrete a variety of proinflammatory factors, such as TNF-*α*, IL-1, and IL-6, leading to an inflammatory cascade (Rana and Singh 2018). Therefore, the inhibition of M1-type microglia could effectively mitigate damage caused by inflammation (Patel et al. 2013). Tid has been reported to decrease neuroinflammation by inhibiting GSK-3β, thus promoting CNS reconstruction (Joshi et al. 2022). The ability of Tid@PLTM to induce neuroprotective M2-type microglia and the anti-inflammatory effects of free Tid, Tid@PLGA and Tid@PLTM were investigated (Figure 3D). LPS was used to induce M1-type BV2 cells (Lee 2019). Tid@PLTM decreased the levels of IL-6 and TNF-*α* and increased the expression of IL-10 and TGF-β1 in a dose-dependent manner (Figure 3E), which was greater than that of free Tid and Tid@PLGA (Figure S6), suggesting the strong anti-inflammatory effect of Tid@PLTM. Additionally, in BV2 cells under oxidative stress induced by OGD/R, Tid@PLTM caused drastic release of IL-10 and TGF-β1 (Figure S7). CD86 (a costimulatory molecule) and CD206 (macrophage mannose receptor) are markers of M1-type and M2-type microglia, respectively. Confocal microscopy revealed weak CD206 expression in the PBS group. With Tid@PLTM treatment, enhanced CD206 expression was observed, similar to that in the positive control IL-4 group (Figure 3F). Additionally, CD86 expression was reduced after Tid@PLTM treatment (Figure 3G). Tid@PLTM successfully induced the polarization of BV2 cells from M1 to M2. These results suggested that Tid@PLTM could inhibit inflammatory BV2 cells and promote their repair functions by inducing M2-type microglia to secrete neuroprotective IL-10 and TGF-β1, verifying the important role of Tid@PLTM in inflammation and the regulation of the microglial phenotype.

We further investigated whether the combination of Cur@PLTM and Tid@PLTM had synergistic antioxidative and anti-inflammatory effects. The results revealed that Cur@PLTM and Tid@PLTM had strong antioxidative effects, which reduced the ROS level (Figure 3H,I). When Tid@PLTM was combined with Cur@PLTM, the ROS levels were reduced 3.9-fold in BV2 cells and 3.3-fold in bEnd.3 cells compared with those with Cur@PLTM alone, which synergistically enhanced the antioxidative effect. We found that Tid has antioxidative capacity comparable to that of Cur, which is because Tid inhibits GSK-3β, leading to the accumulation of Nrf2 in the nucleus and promoting the expression of multiple antioxidative enzymes downstream, thus providing synergistic antioxidative efficacy (Yu and Xiao 2021).

IL-10 and TGF-β1 are the main indicators used to evaluate synergistic anti-inflammatory effects. Both Cur@PLTM and Tid@PLTM could induce the expression of anti-inflammatory IL-10 and TGF-β1, and the combination of PLTM-NPs increased the levels of both cytokines in a dose-dependent manner (Figure 3J, K), indicating a synergistic effect on anti-inflammatory modulation. Cur@PLTM can inhibit oxidative stress and Nf-κB (Li et al. 2017; Ran et al. 2021) to inhibit inflammatory progression.

### 3.4 Evaluations of BBB penetration *in vitro*

**Figure 4.**
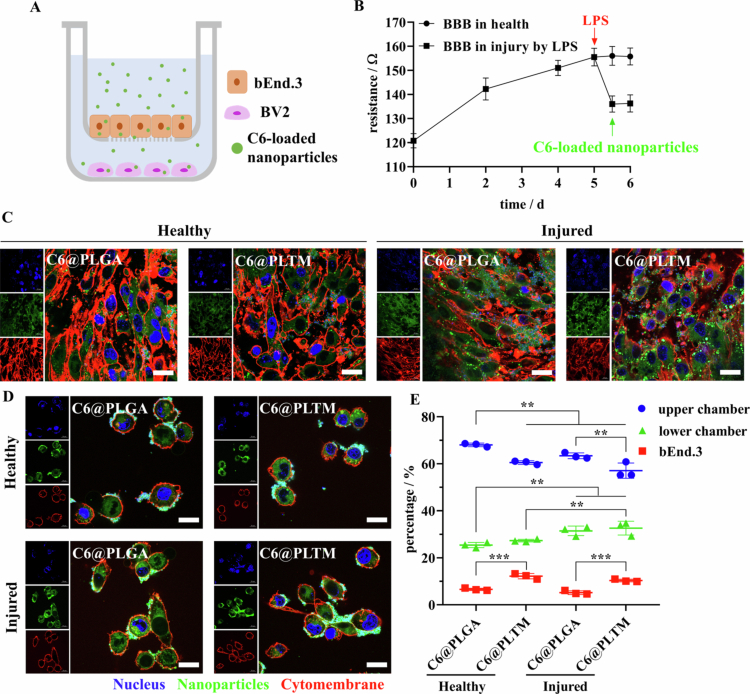
*In vitro* cross-BBB evaluations. (A) Schematic showing the experiment across the BBB *in vitro*. (B) Changes in transendothelial electric resistance (TEER) in the BBB of bEnd.3 cells throughout the experiment (*n* = 4). (C, D) Confocal images of C6-loaded PLGA-NPs or PLTM-NPs in bEnd.3 (C) and BV2 cells (D) under healthy or injured conditions. Scale: 20 μm. (E) Percentages of PLGA-NPs and PLTM-NPs in the upper chamber, bEnd.3 cells or lower chamber quantified by the C6 concentration (*n* = 3). **p < 0.05. ***p < 0.001.

First, bEnd.3 cells assembled a transwell BBB model in which LPS was used to mimic the injured BBB during stroke attack (Figure 4A). The TEER of the assembled transwell BBB model in health was approximately 156 Ω, while it was reduced to 136 Ω by LPS treatment (Figure 4B). Coumarin 6 (C6)-loaded PLGA-NPs or PLTM-NPs were added to the transwell chamber for 10  h, and no significant changes in TEER were observed after PLGA-NPs or PLTM-NPs treatment (Figure 4B). Clear green fluorescence of C6-loaded nanoparticles was observed in bEnd.3 (Figure 4C) and BV2 cells (Figure 4D), indicating that PLGA-NPs and PLTM-NPs crossed the BBB and were subsequently internalized by BV2 cells. Considering that the cumulative release of C6 from PLGA-NPs and PLTM-NPs was less than 6% by dialysis in PBS (Figure S8), this BBB penetration revealed that C6 could be transported across the BBB mainly in the form of nanoparticles.

The specific percentages of nanoparticles in the upper chamber, bEnd.3 cells and lower chamber were quantified (Figure 4E) by the concentration of C6. Under conditions of LPS-induced BBB injury, the overall uptake and transport efficiencies of PLGA-NPs and PLTM-NPs were 36.60% and 42.94%, respectively, which were significantly higher than those under healthy conditions (31.97% and 39.50%, respectively) because LPS destroyed the normal structure of the BBB and increased permeability (Peng et al. 2021). Moreover, PLTM-NPs had the greatest amount of transport to the lower chamber under both healthy and BBB injury conditions, indicating that PLTM-NPs entered the ischemic parenchyma faster. In addition, more PLTM-NPs were internalized by bEnd.3 cells under both healthy and injured conditions, suggesting that the biomimetic modification of the PLTM could increase the interaction between nanoparticles and endothelial cells. In summary, transwell models were used to simulate BBB injury successfully during stroke, and when the BBB was impaired, PLGA-NPs and PLTM-NPs could enter the brain via caveolin-dependent transcytosis, and PLTM-NPs were more efficient at crossing the BBB.

### 3.5 *In vivo* distribution and active targeting of PLTM-NPs

**Figure 5.**
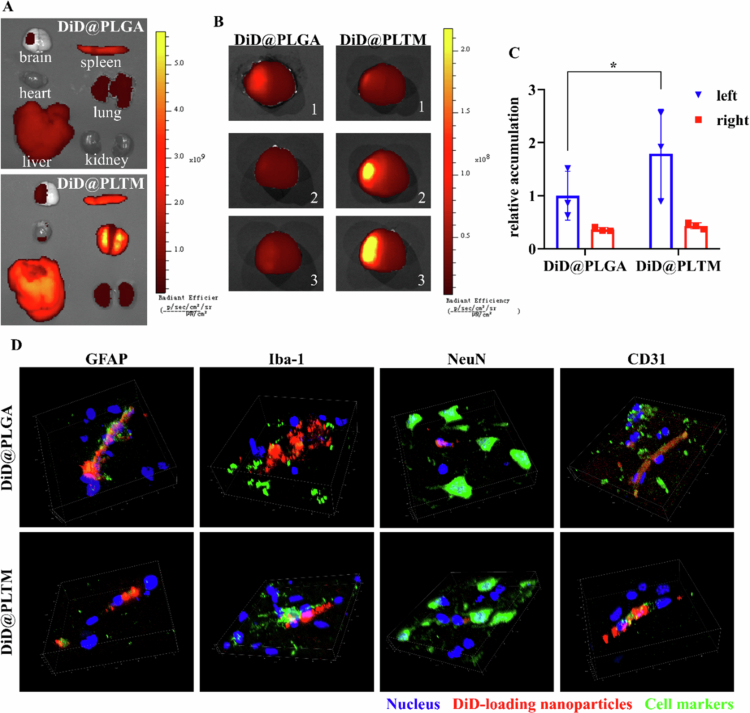
*In vivo* distribution and active targeting of PLTM-NPs. (A) *Ex vivo* distributions of PLGA-NPs and PLTM-NPs in main organs. (B) Intracerebral fluorescence intensity of PLGA-NPs and PLTM-NPs. Compared with the other samples, sample 1 in the DiD@PLTM group presented mild ischemic injury and therefore presented lower accumulation. (C) Semiquantitative analysis of fluorescence intensity in the left and right brain (*n* = 3). (D) Colocalization of PLGA-NPs and PLTM-NPs with astrocytes (GFAP), microglia (Iba-1), neurons (NeuN) and endothelial cells (CD31) in the ischemic cortex. *p < 0.1.

The distributions in the main organs and the targeting to the ischemic areas of PLGA-NPs and PLTM-NPs were investigated in t-MCAO rats. DiD-loaded PLGA-NPs and PLTM-NPs were intravenously injected into the tail vein after 30 min of reperfusion. Twenty-four hours later, the animals were euthanized, the heart, liver, spleen, lung, kidney and brain were dissected, and fluorescence images were taken *ex vivo*. Compared to PLGA-NPs, PLTM-NPs presented stronger fluorescence in the brain and peripheral organs (Figure 5A), indicating that coating the PLTM could effectively prolong the circulation time of the nanoparticles *in vivo*. PLTM-NPs crossed the BBB and accumulated in the ischemic region of the left hemisphere, and the intracerebral fluorescence intensity of PLTM-NPs was significantly higher than that of PLGA-NPs (Figure 5B). PLTM-NPs preferentially entered the ischemic hemisphere, where the content was 0.9 (less accumulation due to slight mild ischemic injury),1.9 and 2.6 times higher than that of PLGA-NPs in the three rats and 4.2 times higher than that in the normal hemisphere (Figure 5C), indicating that interactions of PLTM with damaged cerebral endothelial cells (ECs) provided active targeting feasibility, further enhancing the intracerebral accumulation of PLTM-NPs. The colocalization of PLGA-NPs and PLTM-NPs with astrocytes, microglia, neurons and ECs was observed in the ischemic cortex (Figure 5D) after labeling with GFAP, Iba-1, NeuN and CD31, respectively, indicating that PLGA-NPs and PLTM-NPs progressively penetrated the brain across ECs and astrocytes and were taken up effectively by microglia and neurons. More PLGA-NPs and PLTM-NPs were observed in the ischemic cortex than in the normal cortex, which also confirmed their ability to passively and actively target the nidus (Figure S9). In this study, we demonstrated that the accumulation of PLTM-NPs in the ischemic area in t-MCAO rats was greater and that coating the PLTM prolonged the circulation time of the nanoparticles *in vivo*. In addition, PLGA-NPs and PLTM-NPs could be internalized by a variety of cell types in the brain, thereby exerting the antioxidative and anti-inflammatory effects of Cur and Tid.

### 3.6 *In vivo* efficacy and safety of PLTM-NPs

We have proven that PLTM-NPs have good antioxidative and anti-inflammatory effects *in vitro* and can actively target the ischemic hemisphere *in vivo*. In this study, the therapeutic effects of drug-loaded PLTM-NPs were further explored at the animal level.

**Figure 6.**
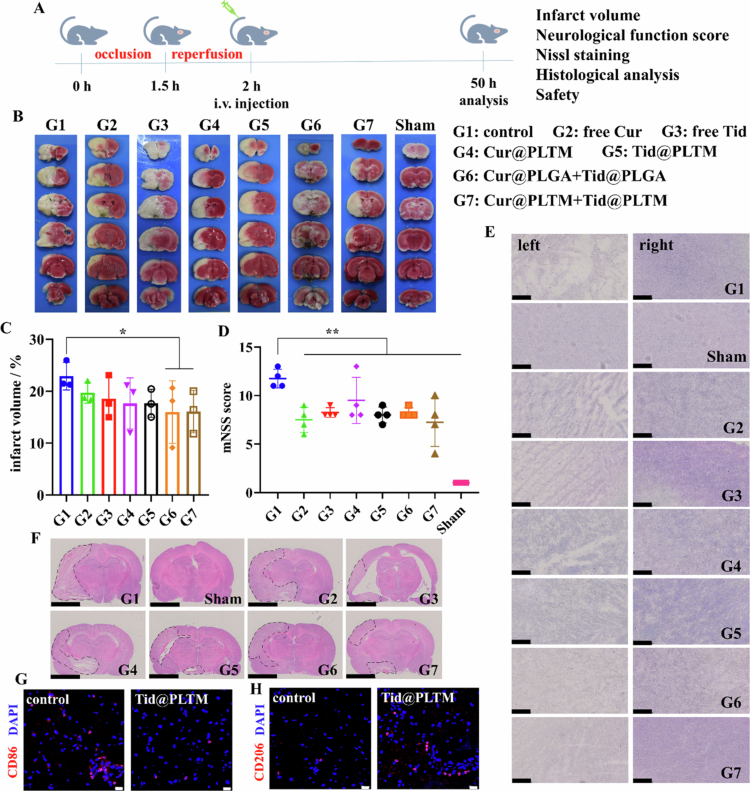
*In vivo* efficacies of PLTM-NPs. (A) Treatment schematic for t-MCAO rats. (B) Triphenyltetrazolium chloride (TTC) staining. (C) Infarct volume (%) analysis (*n* = 3). (D) Neurological function score (*n* = 4). (E) Nissl staining of ischemic (left) and normal (right) cortical areas. Scale: 250 μm. (F) H&E staining of brain slices. The infarct site was marked. Scale: 5 mm. (G, H) CD86 (G) and CD206 (H) staining of the ischemic cortex in the control or Tid@PLTM groups. Scale: 20 μm. *p < 0.1, **p < 0.05.

Cur@PLTM and/or Tid@PLTM were administered at 10 mg/kg to t-MCAO rats via intravenous tail injection at 30 min after reperfusion. The infarct volume, nerve function and degree of neural injury were analyzed (Figure 6A). TTC was used to identify the infarct area in ischemic rats (Figure 6B). The infarct volume (%) was quantified (Figure 6C). There was no infarction in the sham group (infarct volume was 0%), but severe cerebral ischemia (infarct volume was 22.9%) was observed in the no-treatment control group (G1). The infarction areas of all the treatment groups were between those of the sham and control groups. The mean infarct volume of the treated rats was less than 20.0% (Figure 6C). The groups that received Cur@PLTM and Tid@PLTM together (G7) had the smallest mean infarct volume, 16.08%. The mNSS was used to assess neurological deficits after I/R injury, with higher scores indicating more severe neurological damage. After treatment with Cur@PLTM and Tid@PLTM (G7), the mNSS decreased from 12 in the control group to 7 (Figure 6D), suggesting that Cur@PLTM and Tid@PLTM partially alleviated neural deficits in t-MCAO rats. Nissl staining was used to evaluate the function of neurons. Large and abundant Nissl bodies indicate a strong ability of nerve cells to synthesize proteins. In contrast, when nerve cells are damaged, the number of Nissl bodies decreases or even disappears. Compared with those in the normal right cerebral cortex, the number of Nissl bodies in the ischemic left cortex in all surgical groups was significantly lower. Among these effects, neuron loss in the control group was most serious, while the neuron loss and functional impairment were alleviated after different drug treatments (Figure 6E). H&E staining was performed to observe the histological differences between the ischemic and normal hemispheres. The tissue density of the treatment groups, especially G6 and G7, was higher than that of the control group (Figure 6F), thus alleviating neuron death and loss. To further understand the M2-type polarization of microglia induced by Tid@PLTM, CD86 and CD206 expression in the ischemic cortex after Tid@PLTM treatment was explored by immunofluorescence (Figure 6G,H). Compared with the control group, the Tid@PLTM group presented lower CD86 and higher CD206 expression, confirming the M2-type polarization of microglia *in vivo*. We also investigated the *in vivo* safety of each group, mainly by evaluating the histological changes in the heart, liver, spleen, lung and kidney. No tissue anomalies were detected via H&E histological staining, indicating that PLGA-NPs and PLTM-NPs caused no substantial damage to the main organs (Figure S10). In summary, PLGA-NPs and PLTM-NPs had certain therapeutic effects on t-MCAO rats, both of which could reduce infarct volume, neural deficits and neuron loss with no histological damage to the main organs.

## 4 Discussion

Oxidative stress and the inflammatory cascade play multiple destructive roles in stroke progression, aggravating neural dysfunction and hindering tissue repair. Therefore, antioxidation and anti-inflammatory effects are highly important in the prognostic therapy of ischemic stroke. Anti-oxidants that promote free radical scavenging via redox reaction are the main direction, among which vitamins, edaravone, *n*-butylphthalide and Cur are commonly used (Li and Yang 2016). Cur has promising applications in scavenging ROS, enhancing cell resistance to oxidative stress and anti-inflammation, and has shown excellent therapeutic effects in experimental animal models (Thiyagarajan and Sharma 2004; Kakkar et al. 2013; Miao et al. 2016; Farooqui 2016; Shin et al. 2020). Although Cur has excellent neuroprotective potential, it features poor solubility and stability, low bioavailability and short half-life, and is easy to degrade, which limit the efficacy and clinical translation of Cur (Shen et al. 2021).

Restricting excessive inflammation in the acute phase is another important therapeutic approach. M2-type microglia are powerful mediators of CNS reconstruction (Zhao et al. 2017), and diverse targets for facilitating M2-type polarization have been developed (Kim et al. 2019). Tid, a GSK-3β inhibitor, has positive effects on several types of CNS diseases (Horrigan et al. 2020; MARTíNEZ-GONZáLEZ et al. 2021; Bahmad et al. 2021) and has been shown to have antiapoptotic effects and inhibit astrocyte activation during hypoxic brain injury in newborn mice (Wang et al. 2016). The prophylactic potential rather than the posttherapeutic effect of Tid in t-MCAO rats was demonstrated via the modulation of pGSK-3β S9, apoptosis and neuroinflammation (Joshi et al. 2022). In clinical trials, Tid has shown some benefits in patients with progressive supranuclear palsy, congenital myotonic dystrophy and other CNS diseases. We first applied Tid nanoformulations to treat ischemic stroke and demonstrated that the therapeutic effect of Tid was related to its ability to regulate M2-type microglia.

The different states of cells affect the uptake of nanoparticles. BV2 cells under oxidative stress presented increased uptake of PLGA-NPs and PLTM-NPs (Figure 2C). The destruction of the cell membrane plays an important role. Lipid peroxidation by ROS during oxidative stress is detrimental to the structure and function of the membrane and alters fluidity and permeability, allowing easy traversal of the membrane (Yadav et al. 2019). Research has shown that large amounts of ROS decrease the thickness of the cell membrane and that the phospholipid arrangement becomes disordered (Hu et al. 2019), causing cells to be more permeable (Wang et al. 2020).

Cur@PLTM and Tid@PLTM show superior ROS scavenging and anti-inflammatory ability compared with free Cur and Tid at the cellular level (Figures 3B,C and S6), implying better therapeutic potential for ischemic stroke. M2-type microglia, which promote CNS recovery by accelerating the clearance of tissue fragments and the secretion of multiple neurotrophic factors (IL-10, TGF-β1), were successfully induced (Figures 3E–G, S7). IL-10 can inhibit neuroinflammation and astrocyte proliferation and regulate synaptic pruning (Burmeister and Marriott 2018). TGF-*β* has neurotrophic effects and can promote neuron and oligodendrocyte differentiation, neuronal migration and axon elongation (Meyers and Kessler 2017). Moreover, IL-6 and TNF-*α* were downregulated, especially by Tid@PLTM (Figures 3E, S6), demonstrating that Tid@PLTM was effective in limiting the inflammatory cascade. The combination of Cur@PLTM and Tid@PLTM resulted in synergistic antioxidation and anti-inflammatory effects (Figure 3H–K) due to different molecular mechanisms, providing experimental evidence for the combination administration of neuroprotective agents. In detail, as a powerful antioxidant, curcumin has been confirmed to inhibit ROS and NOS generation, mitochondrial dysfunction, and inflammation via the MAPK and Nf-κb pathways (Hu et al. 2023) and to increase the expression of antioxidative enzymes (GST, SOD, HO-1, etc.). The activation of Nrf2 (Duan et al. 2022) results in excellent protection against various brain injuries. In addition, activated GSK-3β is a key regulator that induces the degradation of Nrf2 via phosphorylation (Yu and Xiao 2021). Tideglusib modulates the Nrf2/ARE pathway via GSK-3β inactivation to promote the nuclear translocation of Nrf2 and the expression of NQO1 and HO-1 to ameliorate oxidative stress (Armagan et al. 2019). Additionally, tideglusib can regulate NF-κb and STAT3 to restrain ROS and inflammation (Duda et al. 2020; Lai et al. 2023; Chen et al. 2024). In summary, both curcumin and tideglusib have various protective mechanisms to have synergistic effects on antioxidation and inflammation.

We further demonstrated that PLTM-NPs have a superior ability to cross the BBB both *in vitro* and *in vivo* via transcytosis, contributing to biomimetic modification to prolong the circulation time of nanoparticles and provide active targeting to ischemic cerebral vessels via interactions of GPⅥ, cell-adhesion molecules (ICAM, VCAM), CD40/CD40L and integrins on PLTM with fibrinogen and E-selectin/P-selectin upregulated on injured ECs (Shaik et al. 2021). During I/R injury, cytoskeletal rearrangement, increased transcytosis and alterations in tight junction proteins occur in ECs, contributing to stepwise BBB dysfunction (Jiang et al. 2018). Caveolae-mediated endothelial transcytosis (especially that mediated by caveolin-1) is enhanced within hours after ischemia, prior to and independent of tight junction disintegration (2 days later) (Al-Ahmady et al. 2019; Zhou et al. 2021). Therefore, the administered PLGA-NPs and PLTM-NPs at 30 min after reperfusion were delivered to the brain mainly by caveolin-dependent transcytosis (Figures 2E, 4). Compared with nonspecific PLGA-NPs, PLTM-NPs packed with properties of activated platelets could have specific receptor‒ligand interactions with injured brain endotheliocytes to achieve receptor-mediated transport. In activated endotheliocytes, P-selectin exhibited substantial colocalization with caveolin-1 (Tylawsky et al. 2023), which effectively primed *P*-selectin-bound PLTM-NPs for uptake by caveolae-mediated endocytosis and transcytosis across the BBB. After crossing the BBB, the nanoparticles are subsequently internalized by astrocytes, microglia and neurons, thus exerting therapeutic effects on a variety of cell types. This divergent protection provided a more comprehensive opportunity for CNS restoration.

In this study, the biomimetic modification increased the concentration of drugs at the ischemic site *in vivo* (Figure 5A–C) because of the low immunogenicity, which reduced elimination of the NPs, and active targeting to facilitate drug entry via receptor‒ligand interactions. We also verified the M2-type polarization of microglia by Tid@PLTM *in vivo* (Figure 6G,H), which confirmed the anti-inflammatory regulation by Tid@PLTM. Although Cur@PLTM and Tid@PLTM ameliorated neuron loss and functional impairment in t-MCAO rats, the effects were less than expected (Figure 6). This may be related to the inability of a single dose to effectively ameliorate such a large infarct size (22.89% in the t-MCAO model). Multiple doses are expected to accumulate more drugs at the site of cerebral ischemia and should have a better effect. Additionally, loading more drugs in nanosystems is something that needs to be explored. Furthermore, explorations *in vivo* of oxidative stress, inflammation and microglial polarization, including markers and cytokines, would help to clarify the mechanism and specific influence related to neuroprotection. To date, nanovehicles and targeting strategies are still powerful tools for improving drug properties and therapeutic benefits.

In summary, platelet membrane-grooming PLGA nanosystems have been constructed with good biocompatibility and can be used for the delivery of neuroprotective agents into the ischemic area of the brain via active targeting and transendothelial transport. Owing to the strong multipotent antioxidative effect and microglial M2-type polarization capacity of Cur and Tid, synergistic administration was more beneficial for antioxidative and anti-inflammatory therapy. At present, more studies have focused on nanodelivery systems for the treatment of ischemic stroke, which greatly expand the effects of traditional neuroprotective agents and provide more directions for drug combinations. Nanotherapy is expected to improve the therapeutic effect and prognosis of stroke in the future.

## Ethical approval

The study obtained approval from the Animal Ethics Committee of Peking University Health Science Center (assigned number: LA2019188). All animal experiments were performed according to the guidelines of the National Institutional Animal Care and Care and Use of Laboratory Animals of Peking University. The authors have adhered to the ARRIVE guidelines. Male SD rats (250−280 g, *n* = 40) were kept in a clean environment at the proper temperature (20 °C−26 °C), humidity (40%-60%), 12 h day/night cycle, and free access to standard solid chow and water. The rats were anesthetized with 2% isoflurane during surgery in the t-MCAO model to minimize suffering. At the end of the study, all the animals were euthanized using inhalation anesthesia with 5% isoflurane.

Justification for the use of animals: The rat t-MCAO model is crucial for exploring and assessing the safety, toxicity, drug distribution, targeting and efficacy of biomimetic nanoparticles for treating ischemic stroke. Despite the difference between rat and human conditions, the rat t-MCAO model presents the disease commonality of stroke and plays a vital role in biomedical research.

## Supplementary Material

Supplementary material Supplementary material_20250731.docx

Supplementary material Original Images for Fig S1_Fig S9.zip

Supplementary material Original Images for Fig 1_Fig 2.zip

Supplementary material Original Images for Fig S10_2.zip

Supplementary material Original Images for Fig 5.zip

Supplementary material Original Images for Fig S10_3.zip

Supplementary material Original Images for Fig 3_Fig 4.zip

Supplementary material Original Images for Fig S10_1.zip

Supplementary material Original Images for Fig S10_4.zip

Supplementary material Raw data.xlsx

Supplementary material Original Images for Fig 6.zip

## Data Availability

The data that support the findings of this study are available from the corresponding author upon reasonable request.

## References

[cit0001] Abban CY, Bradbury NA, Meneses PI. 2008. HPV16 and BPV1 infection can be blocked by the dynamin inhibitor dynasore. Am J Ther. 15(4):304–311. 10.1097/MJT.0b013e3181754134 18645330 PMC2519006

[cit0002] Al-Ahmady ZS, Jasim D, Ahmad SS, et al. 2019. Selective liposomal transport through blood brain barrier disruption in ischemic stroke reveals two distinct therapeutic opportunities. ACS Nano. 13(11):12470–12486. 10.1021/acsnano.9b01808 31693858

[cit0003] Alawieh A, Elvington A, Tomlinson S. 2015. Complement in the homeostatic and ischemic brain. Front Immunol. 6:417. 10.3389/fimmu.2015.00417 26322048 PMC4533015

[cit0004] Armagan G, Sevgili E, Gürkan FT, et al. 2019. Regulation of the nrf2 pathway by glycogen synthase kinase-3? in MPP+-Induced Cell Damage. Molecules. 24(7):1377. 10.3390/molecules24071377 30965670 PMC6480928

[cit0005] Ashrafizadeh M, Ahmadi Z, Mohammadinejad R, et al.. 2020. Curcumin Activates the Nrf2 Pathway and Induces Cellular Protection Against Oxidative Injury. Curr Mol Med. 20(2):116–133. 31622191 10.2174/1566524019666191016150757

[cit0006] Bachi A, Dalle-Donne I, Scaloni A. 2013. Redox proteomics: chemical principles, methodological approaches and biological/biomedical promises. Chem Rev. 113(1):596–698. 10.1021/cr300073p 23181411

[cit0007] Bahmad HF, Chalhoub RM, Harati H, et al. 2021. Tideglusib attenuates growth of neuroblastoma cancer stem/progenitor cells in vitro and in vivo by specifically targeting GSK-3β. Pharmacol Rep. 73(1):211–226. 10.1007/s43440-020-00162-7 33030673

[cit0008] Burmeister AR, Marriott I. 2018. The Interleukin-10 family of cytokines and their role in the CNS. Front Cell Neurosci. 12:458. 10.3389/fncel.2018.00458 30542269 PMC6277801

[cit0009] Chang, C-C, Wu M, Yuan F. 2014. Role of specific endocytic pathways in electrotransfection of cells. Mol Ther Methods Clin Dev. 1:14058. 10.1038/mtm.2014.58 26052524 PMC4448742

[cit0010] Chen M, Fang Y, Ge Y, et al. 2024. The redox-sensitive GSK3β is a key regulator of glomerular podocyte injury in type 2 diabetic kidney disease. Redox Biol. 72:103127. 10.1016/j.redox.2024.103127 38527400 PMC10979123

[cit0011] Donkor E S. 2018. Stroke in the 21(st) century: a snapshot of the burden, epidemiology, and quality of life. Stroke Res Treat. 2018:3238165. 30598741 10.1155/2018/3238165 PMC6288566

[cit0012] Doyle KP, Simon RP, Stenzel-Poore MP. 2008. Mechanisms of ischemic brain damage. Neuropharmacology. 55(3):310–318. 10.1016/j.neuropharm.2008.01.005 18308346 PMC2603601

[cit0013] Duan C, Wang H, Jiao D, et al. 2022. Curcumin restrains oxidative stress of after intracerebral hemorrhage in rat by activating the Nrf2/HO-1 Pathway. Front Pharmacol. 13. 10.3389/fphar.2022.889226 PMC9092178 35571134

[cit0014] Duda P, Akula SM, Abrams SL, et al. 2020. Targeting GSK3 and associated signaling pathways involved in cancer. Cells. 9(5):1110. 10.3390/cells9051110 32365809 PMC7290852

[cit0015] El Amki M, Gluck C, Binder N, et al. 2020. Neutrophils obstructing brain capillaries are a major cause of no-reflow in ischemic stroke. Cell Rep. 33(2):108260. 10.1016/j.celrep.2020.108260 33053341

[cit0016] Elmowafy EM, Tiboni M, Soliman ME. 2019. Biocompatibility, biodegradation and biomedical applications of poly(lactic acid)/poly(lactic-co-glycolic acid) micro and nanoparticles. J Pharm Investig. 49(4):347–380. 10.1007/s40005-019-00439-x

[cit0017] Farooqui AA. 2016. Therapeutic importance of curcumin in neurological disorders other than alzheimer disease [M]//FAROOQUI A A. In: Therapeutic potentials of curcumin for alzheimer disease. Cham: Springer International Publishing; p. 297–334. 10.1007/978-3-319-15889-1_8

[cit0018] Fisher M, Savitz SI. 2022. Pharmacological brain cytoprotection in acute ischaemic stroke — renewed hope in the reperfusion era. Nat Rev Neurol. 18(4):193–202. 10.1038/s41582-021-00605-6 35079135 PMC8788909

[cit0019] Gawaz M, LANGER H, MAY A E. 2005. Platelets in inflammation and atherogenesis. J Clin Investig. 115(12):3378–3384. 10.1172/JCI27196 16322783 PMC1297269

[cit0020] Gonzalez-Reyes S, Guzman-Beltran S, Medina-Campos ON, et al. 2013. Curcumin pretreatment induces Nrf2 and an antioxidant response and prevents hemin-induced toxicity in primary cultures of cerebellar granule neurons of rats. Oxid Med Cell Longev. 2013:801418. 10.1155/2013/801418 24454990 PMC3885319

[cit0021] He W, Zhang Z, Sha X. 2021. Nanoparticles-mediated emerging approaches for effective treatment of ischemic stroke. Biomaterials. 277:121111. 10.1016/j.biomaterials.2021.121111 34488117

[cit0022] Horrigan J, Gomes TB, Snape M, et al. 2020. A Phase 2 Study of AMO-02 (Tideglusib) in Congenital and Childhood-Onset Myotonic Dystrophy Type 1 (DM1). Pediatr Neurol. 112:84–93. 10.1016/j.pediatrneurol.2020.08.001 32942085

[cit0023] Hu P, Li K, Peng XX, et al. 2023. Curcumin derived from medicinal homologous foods: its main signals in immunoregulation of oxidative stress, inflammation, and apoptosis. Front Immunol. 14:1233652. 10.3389/fimmu.2023.1233652 37497225 PMC10368479

[cit0024] Hu X, Leak RK, Shi Y, et al. 2015. Microglial and macrophage polarization-new prospects for brain repair. Nat Rev Neurol. 11(1):56–64. 10.1038/nrneurol.2014.207 25385337 PMC4395497

[cit0025] Hu Y, Zhao T, Zou L, et al. 2019. Molecular dynamics simulations of membrane properties affected by plasma ROS based on the GROMOS force field. Biophys Chem. 253:106214. 10.1016/j.bpc.2019.106214 31272076

[cit0026] Jian Z, Liu R, Zhu X, et al. 2019. The Involvement and therapy target of immune cells after ischemic stroke. Front Immunol. 10:2167. 10.3389/fimmu.2019.02167 31572378 PMC6749156

[cit0027] Jiang X, Andjelkovic AV, Zhu L, et al. 2018. Blood-brain barrier dysfunction and recovery after ischemic stroke. Prog Neurobiol. 163-164:144–171. 10.1016/j.pneurobio.2017.10.001 28987927 PMC5886838

[cit0028] Jin R, Liu L, Zhang S, et al. 2013. Role of inflammation and its mediators in acute ischemic stroke. J Cardiovasc Transl Res. 6(5):834–851. 10.1007/s12265-013-9508-6 24006091 PMC3829610

[cit0029] Joshi B, Singh D, Wasan H, et al. 2022. Tideglusib Ameliorates Ischemia/Reperfusion damage by Inhibiting GSK-3β and apoptosis in rat model of ischemic stroke. J Stroke Cerebrovasc Dis. 31(4):106349. 10.1016/j.jstrokecerebrovasdis.2022.106349 35152130

[cit0030] Jurcau A, Ardelean IA. 2021. Molecular pathophysiological mechanisms of ischemia/reperfusion injuries after recanalization therapy for acute ischemic stroke. J Integr Neurosci. 20(3):727–744. 10.31083/j.jin2003078 34645107

[cit0031] Jurcau A, Simion A. 2022. Neuroinflammation in cerebral ischemia and ischemia/reperfusion injuries: from pathophysiology to therapeutic strategies. Int J Mol Sci. 23(1), 10.3390/ijms23010014 PMC8744548 35008440

[cit0032] Kakkar V, Muppu SK, Chopra K, et al. 2013. Curcumin loaded solid lipid nanoparticles: an efficient formulation approach for cerebral ischemic reperfusion injury in rats. Eur J Pharm Biopharm. 85(3 Pt A):339–345. 10.1016/j.ejpb.2013.02.005 23454202

[cit0033] Kim SH, Noh MY, Kim HJ, et al. 2019. A therapeutic strategy for Alzheimer's disease focused on immune-inflammatory modulation. Dement Neurocogn Disord. 18(2):33–46. 10.12779/dnd.2019.18.2.33 31297134 PMC6609533

[cit0034] Lai S, Wang P, Gong J, et al. 2023. New insights into the role of GSK-3β in the brain: from neurodegenerative disease to tumorigenesis. PeerJ. 11:e16635. 10.7717/peerj.16635 38107562 PMC10722984

[cit0035] Lee KY. 2019. M1 and M2 polarization of macrophages: a mini-review. Med Biol Sci Eng. 2(1):1–5. 10.30579/mbse.2019.2.1.1

[cit0036] Li C, Sun T, Jiang C. 2021. Recent advances in nanomedicines for the treatment of ischemic stroke. Acta Pharm Sin B. 11(7):1767–1788. 10.1016/j.apsb.2020.11.019 34386320 PMC8343119

[cit0037] Liu J, Kaksonen M, Drubin DG, et al. 2006. Endocytic vesicle scission by lipid phase boundary forces. Proc Natl Acad Sci. 103(27):10277–10282. 10.1073/pnas.0601045103 16801551 PMC1502448

[cit0038] Li W, Suwanwela NC, Patumraj S. 2016. Curcumin by down-regulating NF-kB and elevating Nrf2, reduces brain edema and neurological dysfunction after cerebral I/R. Microvasc Res. 106:117–127. 10.1016/j.mvr.2015.12.008 26686249

[cit0039] Li W, Suwanwela NC, Patumraj S. 2017. Curcumin prevents reperfusion injury following ischemic stroke in rats via inhibition of NF‑κB, ICAM-1, MMP-9 and caspase-3 expression. Mol Med Rep. 16(4):4710–4720. 10.3892/mmr.2017.7205 28849007 PMC5647023

[cit0040] Li W, Yang S. 2016. Targeting oxidative stress for the treatment of ischemic stroke: Upstream and downstream therapeutic strategies. Brain Circ. 2(4):153–163. 10.4103/2394-8108.195279 30276293 PMC6126224

[cit0041] Luo J. 2012. The role of GSK3beta in the development of the central nervous system. Front Biol (Beijing). 7(3):212–220. 10.1007/s11515-012-1222-2 25688261 PMC4327837

[cit0042] Ma Q, Li R, Wang L, et al. 2021. Temporal trend and attributable risk factors of stroke burden in China, 1990–2019: an analysis for the global burden of disease study 2019. The Lancet Public Health. 6(12):e897–e906. 10.1016/S2468-2667(21)00228-0 34838196 PMC9047702

[cit0043] Martínez-González L, Gonzalo-Consuegra C, Gómez-Almería M, et al. 2021. Tideglusib, a Non-ATP competitive inhibitor of GSK-3β as a drug candidate for the treatment of amyotrophic lateral sclerosis. Int J Mol Sci. 22(16):8975. 10.3390/ijms22168975 34445680 PMC8396476

[cit0044] Meyers EA, Kessler JA. 2017. TGF-β family signaling in neural and neuronal differentiation, development, and function. Cold Spring Harb Perspect Biol. 9(8):a022244. 10.1101/cshperspect.a022244 28130363 PMC5538418

[cit0045] Miao Y, Zhao S, Gao Y, et al. 2016. Curcumin pretreatment attenuates inflammation and mitochondrial dysfunction in experimental stroke: the possible role of Sirt1 signaling. Brain Res Bull. 121:9–15. 10.1016/j.brainresbull.2015.11.019 26639783

[cit0046] Murphy S, Werring J, D J. 2020. Stroke: causes and clinical features. Medicine. 48(9):561–566. 32837228 10.1016/j.mpmed.2020.06.002 PMC7409792

[cit0047] Nieswandt B, Pleines I, Bender M. 2011. Platelet adhesion and activation mechanisms in arterial thrombosis and ischaemic stroke. J Thromb Haemost. 9(s1):92–104. 10.1111/j.1538-7836.2011.04361.x 21781245

[cit0048] Parvez S, Kaushik M, Ali M, et al. 2022. Dodging blood brain barrier with “nano” warriors: novel strategy against ischemic stroke. Theranostics. 12(2):689–719. 10.7150/thno.64806 34976208 PMC8692911

[cit0049] Patel AR, Ritzel R, Mccullough LD, et al. 2013. Microglia and ischemic stroke: a double-edged sword. Int J Physiol Pathophysiol Pharmacol. 5(2):73–90. 23750306 PMC3669736

[cit0050] Patel P, Yavagal D, Khandelwal P. 2020. Hyperacute management of ischemic strokes: JACC focus seminar. J Am Coll Cardiol. 75(15):1844–1856. 10.1016/j.jacc.2020.03.006 32299596

[cit0051] Peng Y, Yang Y, Yang Z, et al. 2024. Bionic immunoactivator copresenting autophagy promoting and costimulatory molecules for synergistic cancer immunotherapy. NaRes. 17(3):1710–1724. 10.1007/s12274-023-5933-2

[cit0052] Peng X, Luo Z, He S, et al. 2021. Blood-brain barrier disruption by lipopolysaccharide and sepsis-associated encephalopathy. Front Cell Infect Microbiol. 11:768108. 10.3389/fcimb.2021.768108 34804998 PMC8599158

[cit0053] Qin C, Zhou LQ, Ma XT, et al. 2019. Dual functions of microglia in ischemic stroke. Neurosci Bull. 35(5):921–933. 10.1007/s12264-019-00388-3 31062335 PMC6754485

[cit0054] Rajkovic O, Potjewyd G, Pinteaux E. 2018. Regenerative medicine therapies for targeting neuroinflammation after stroke. Front Neurol. 9:734. 10.3389/fneur.2018.00734 30233484 PMC6129611

[cit0055] Ran Y, Su W, Gao F, et al. 2021. Curcumin ameliorates white matter injury after ischemic stroke by inhibiting microglia/macrophage pyroptosis through NF-*κ*B suppression and NLRP3 inflammasome inhibition. Oxid Med Cell Longevity. 2021:1552127. 10.1155/2021/1552127 PMC8497115 34630845

[cit0056] Rana AK, Singh D. 2018. Targeting glycogen synthase kinase-3 for oxidative stress and neuroinflammation: opportunities, challenges and future directions for cerebral stroke management. Neuropharmacology. 139:124–136. 10.1016/j.neuropharm.2018.07.006 30017999

[cit0057] Rawish E, Nording H, MüNTE T, et al. 2020. Platelets as mediators of neuroinflammation and Thrombosis. Front Immunol. 11:548631. 10.3389/fimmu.2020.548631 33123127 PMC7572851

[cit0058] Rodriguez-Vargas JM, Ruiz-Magana MJ, Ruiz-Ruiz C, et al. 2012. ROS-induced DNA damage and PARP-1 are required for optimal induction of starvation-induced autophagy. Cell Res. 22(7):1181–1198. 10.1038/cr.2012.70 22525338 PMC3391023

[cit0059] Roux A, Uyhazi K, Frost A, et al. 2006. GTP-dependent twisting of dynamin implicates constriction and tension in membrane fission. Nature. 441(7092):528–531. 10.1038/nature04718 16648839

[cit0060] Shaik NF, Regan RF, Naik UP. 2021. Platelets as drivers of ischemia/reperfusion injury after stroke. Blood Adv. 5(5):1576–1584. 10.1182/bloodadvances.2020002888 33687431 PMC7948278

[cit0061] Shen LH, Li Y, Chen J, et al. 2006. Intracarotid transplantation of bone marrow stromal cells increases axon-myelin remodeling after stroke. Neuroscience. 137(2):393–399. 10.1016/j.neuroscience.2005.08.092 16298076

[cit0062] Shen LM, Li MC, Wei WJ, et al. 2021. In vitro neuroprotective effects of macrophage membrane-derived curcumin-loaded carriers against 1-methyl-4-phenylpyridinium-induced neuronal damage. ACS Omega. 6(47):32133–32141. 10.1021/acsomega.1c04894 34870034 PMC8637945

[cit0063] Shin JW, Chun KS, Kim DH, et al. 2020. Curcumin induces stabilization of Nrf2 protein through Keap1 cysteine modification. Biochem Pharmacol. 173:113820. 10.1016/j.bcp.2020.113820 31972171

[cit0064] Stoll G, Nieswandt B. 2019. Thrombo-inflammation in acute ischaemic stroke — implications for treatment. Nat Rev Neurol. 15(8):473–481. 10.1038/s41582-019-0221-1 31263257

[cit0065] Su LJ, Zhang JH, Gomez H, et al. 2019. Reactive oxygen species-induced lipid peroxidation in apoptosis, autophagy, and ferroptosis. Oxid Med Cell Longev. 2019:5080843. 10.1155/2019/5080843 31737171 PMC6815535

[cit0066] Subedi L, Gaire BP. 2021. Neuroprotective effects of curcumin in cerebral ischemia: cellular and molecular mechanisms. ACS Chem Neurosci. 12(14):2562–2572. 10.1021/acschemneuro.1c00153 34251185

[cit0067] Sun MS, Jin H, Sun X, et al. 2018. Free radical damage in ischemia-reperfusion injury: an obstacle in acute ischemic stroke after revascularization therapy. Oxid Med Cell Longev. 2018:3804979. 10.1155/2018/3804979 29770166 PMC5892600

[cit0068] Thiyagarajan M, Sharma SS. 2004. Neuroprotective effect of curcumin in middle cerebral artery occlusion induced focal cerebral ischemia in rats. Life Sci. 74(8):969–985. 10.1016/j.lfs.2003.06.042 14672754

[cit0069] Tian T, Zhang HX, He CP, et al. 2018. Surface functionalized exosomes as targeted drug delivery vehicles for cerebral ischemia therapy. Biomaterials. 150:137–149. 10.1016/j.biomaterials.2017.10.012 29040874

[cit0070] Tian X, Fan T, Zhao W, et al. 2021. Recent advances in the development of nanomedicines for the treatment of ischemic stroke. Bioactive Materials. 6(9):2854–2869. 10.1016/j.bioactmat.2021.01.023 33718667 PMC7905263

[cit0071] Tomaiuolo M, Brass LF, Stalker TJ. 2017. Regulation of platelet activation and coagulation and its role in vascular injury and arterial thrombosis. Interv Cardiol Clin. 6(1):1–12. 10.1016/j.iccl.2016.08.001 27886814 PMC5154246

[cit0072] Tylawsky DE, Kiguchi H, Vaynshteyn J, et al. 2023. P-selectin-targeted nanocarriers induce active crossing of the blood–brain barrier via caveolin-1-dependent transcytosis. Nat Mater. 22(3):391–399. 10.1038/s41563-023-01481-9 36864161 PMC9981459

[cit0073] Wang H, Huang S, Yan K, et al. 2016. Tideglusib, a chemical inhibitor of GSK3β, attenuates hypoxic-ischemic brain injury in neonatal mice. Biochim Biophy Acta. 1860(10):2076–2085. 10.1016/j.bbagen.2016.06.027 27378458

[cit0074] Wang L, Sha Y, Wu D, et al. 2020. Surfactant induces ROS-mediated cell membrane permeabilization for the enhancement of mannatide production. Process Biochem. 91:172–180. 10.1016/j.procbio.2019.12.009

[cit0075] Xia CY, Zhang S, Gao Y, et al. 2015. Selective modulation of microglia polarization to M2 phenotype for stroke treatment. Int Immunopharmacol. 25(2):377–382. 10.1016/j.intimp.2015.02.019 25704852

[cit0076] Yadav DK, Kumar S, Choi EH, et al. 2019. Molecular dynamic simulations of oxidized skin lipid bilayer and permeability of reactive oxygen species. Sci Rep. 9(1):4496. 10.1038/s41598-019-40913-y 30872693 PMC6418262

[cit0077] Yang C, Zhang X, Fan H, et al. 2009. Curcumin upregulates transcription factor Nrf2, HO-1 expression and protects rat brains against focal ischemia. Brain Res. 1282:133–141. 10.1016/j.brainres.2009.05.009 19445907

[cit0078] Yang Z-Z, Gao W, Liu Y-J, et al. 2017. Delivering siRNA and chemotherapeutic molecules across BBB and BTB for intracranial glioblastoma therapy. Mol Pharm. 14(4):1012–1022. 10.1021/acs.molpharmaceut.6b00819 28252970

[cit0079] Yu C, Xiao J-H. 2021. The keap1-Nrf2 System: a mediator between oxidative stress and aging. Oxid Med Cell Longevity. 2021(1):6635460. 10.1155/2021/6635460 PMC8106771 34012501

[cit0080] Zhao S-C, Ma L-S, Chu Z-H, et al. 2017. Regulation of microglial activation in stroke. Acta Pharmacol Sin. 38(4):445–458. 10.1038/aps.2016.162 28260801 PMC5386316

[cit0081] Zhou M, Shi SX, Liu N, et al. 2021. Caveolae-mediated endothelial transcytosis across the blood-brain barrier in acute ischemic stroke. J Clin Med. 10(17):3795. 10.3390/jcm10173795 34501242 PMC8432094

