# Supplementary material for: Targeted strategy by curcumin and tideglusib biomimetic nano-systems alleviates oxidative stress and inflammation under ischemic stroke
Source: Drug Deliv. 2025 Nov 25;32(1):2585599. doi: 10.1080/10717544.2025.2585599 (PMC12667299; doi:10.1080/10717544.2025.2585599)
Supplement: Supplementary material — _20250731.docx [file IDRD_A_2585599_SM5399.docx]

Targeted strategy by curcumin and tideglusib biomimetic nano-systems alleviates oxidative stress and inflammation under ischemic stroke

Jiajia Li, Yiliang Yang, Meng Lin, Yitian Du, Yiwei Peng, Yu Zhou, Datong Gao, Yanxia Zhou, Xinru Li, Xianrong Qi*


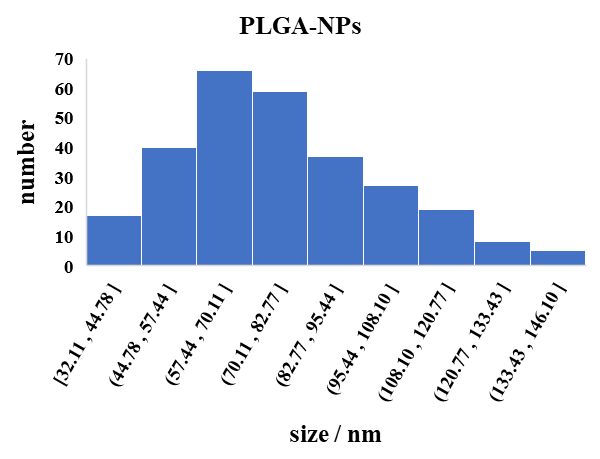

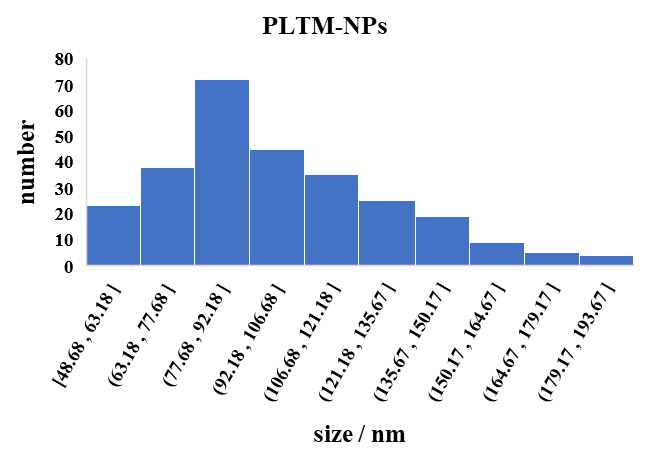


Fig. S1 Size distributions of PLGA-NPs and PLTM-NPs by TEM.


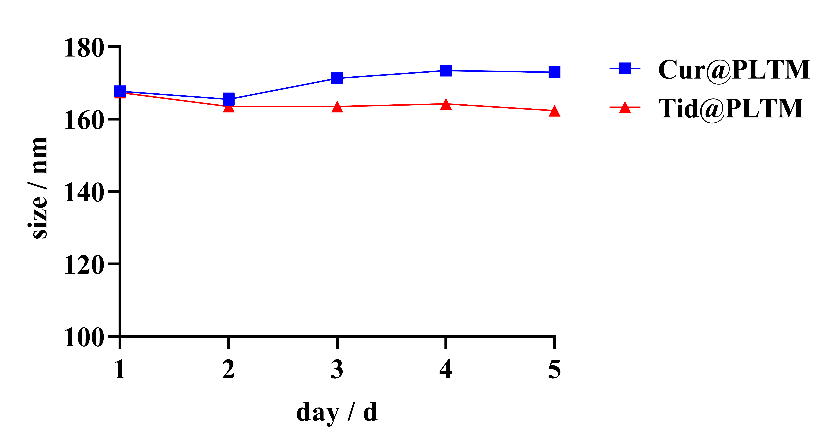


Fig. S2 Size change of PLTM-NPs within five days at 4℃.


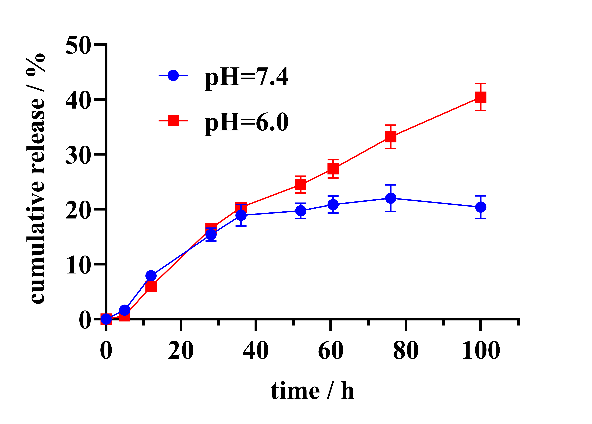


Fig. S3 Cumulative release of curcumin from Cur@PLTM within 100 h (n=3).


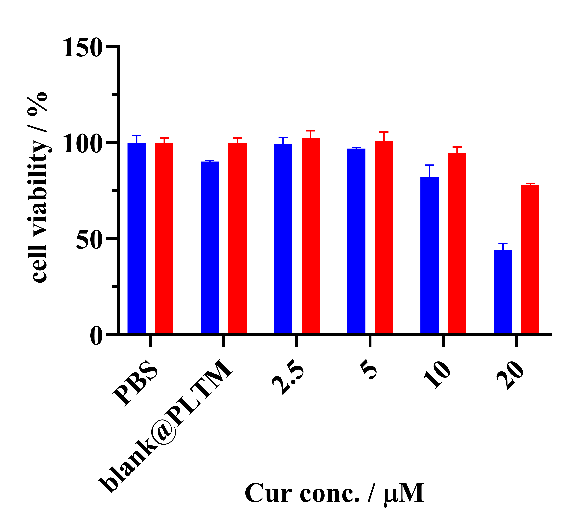

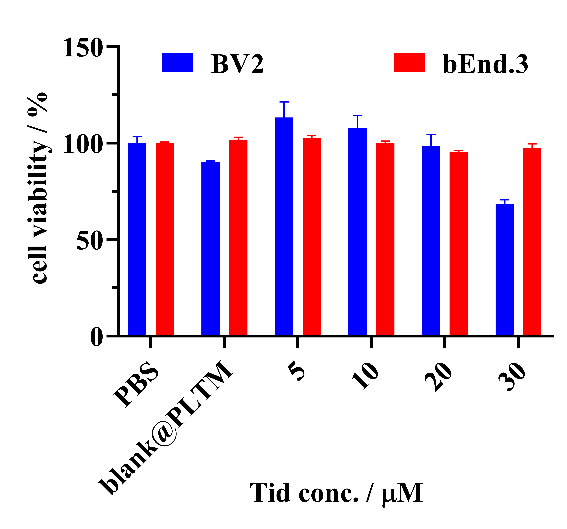


Fig. S4 Cell viabilities of BV2 and bEnd.3 treated by Cur@PLTM or Tid@PLTM for 48 h (n=3).


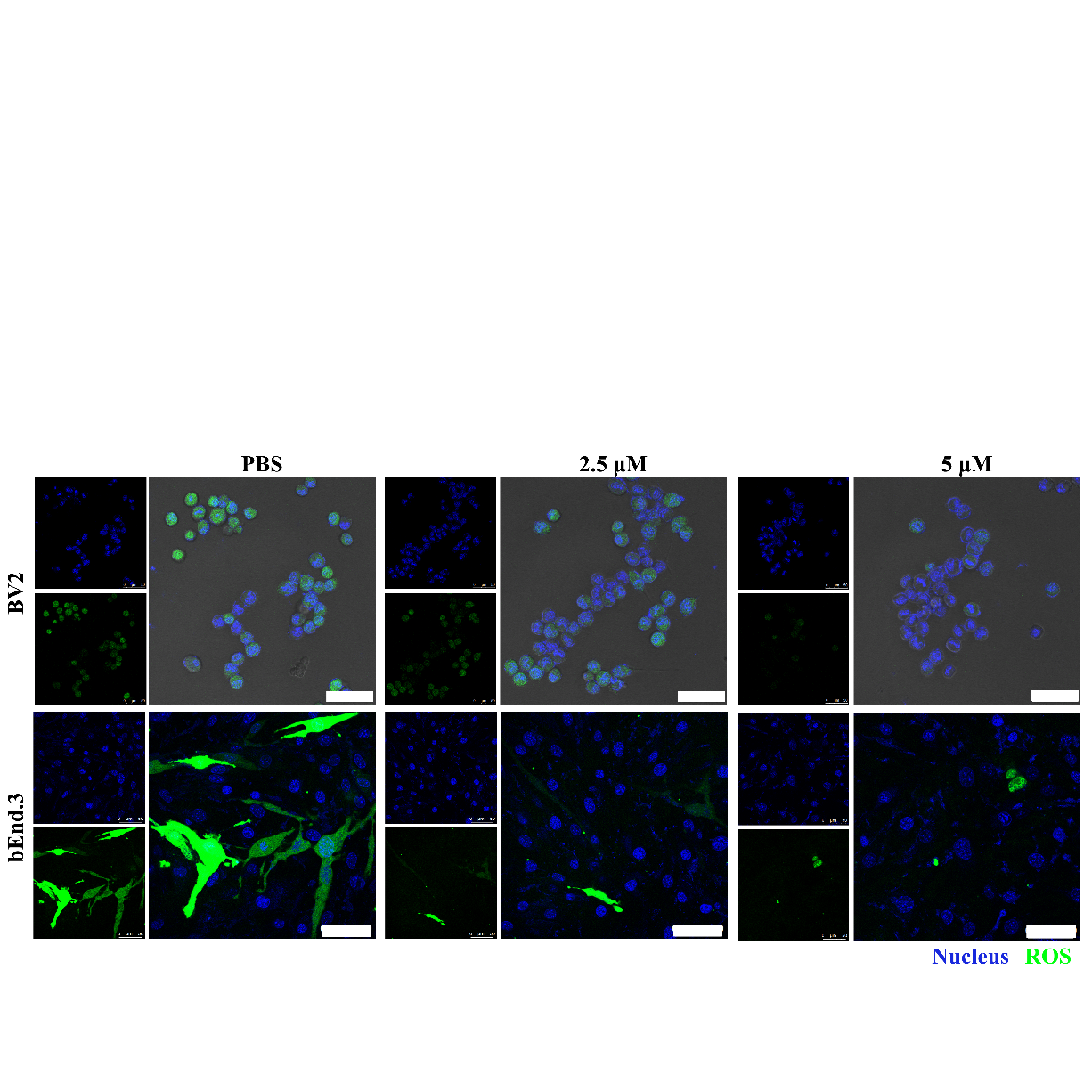


Fig. S5 Confocal images of ROS in BV2 and bEnd.3 cells. The concentration of Cur@PLTM was 2.5 μM or 5 μM. Scale: 50 μm.


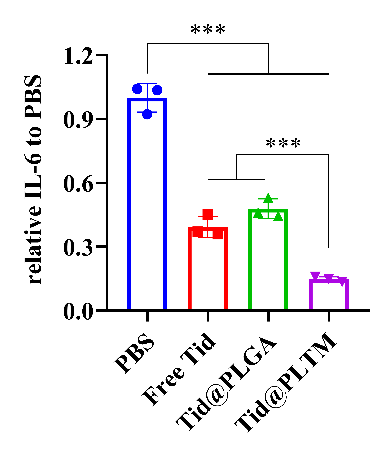

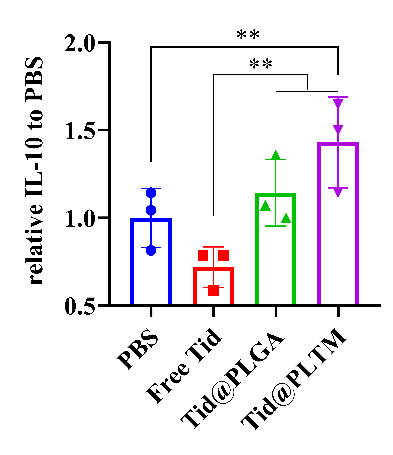

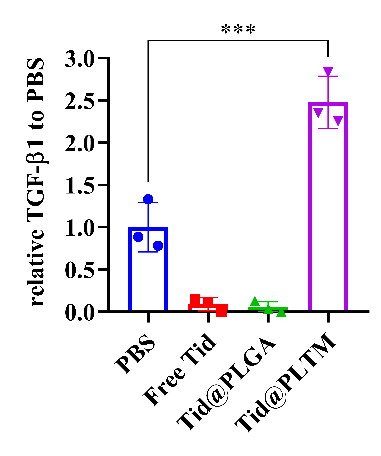


Fig. S6 Comparison of cytokine levels induced by different formulations at the same Tid concentration (20 μM) in BV2 cells (n=3).


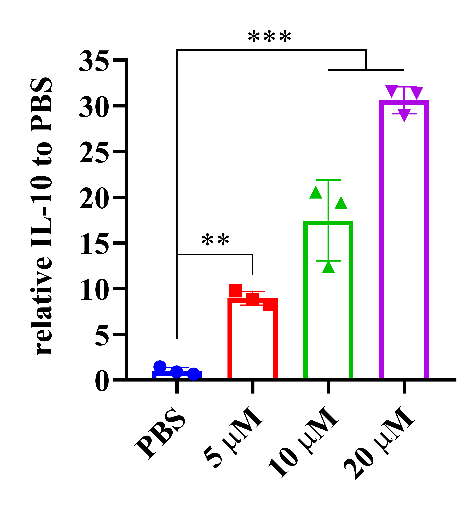

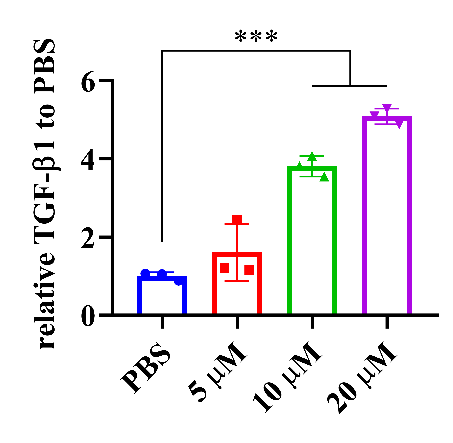


Fig. S7 Effect of Tid concentration in Tid@PLTM on cytokines levels of IL-10 and TGF-β1 in BV2 cells conducted OGD/R (n=3).


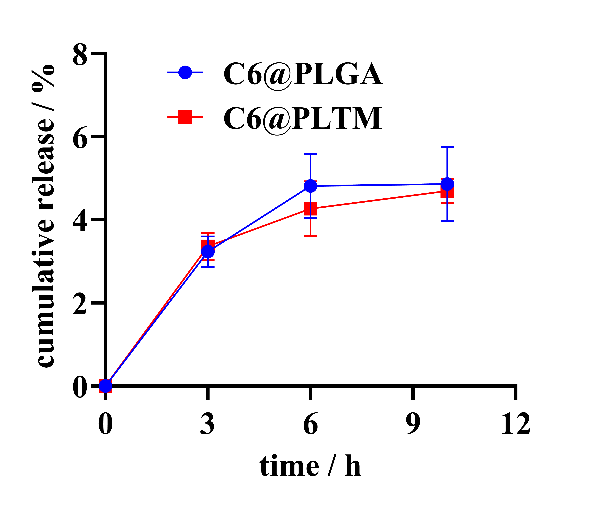


Fig. S8 Cumulative releases of C6@PLGA and C6@PLTM within 10 h (n=3).


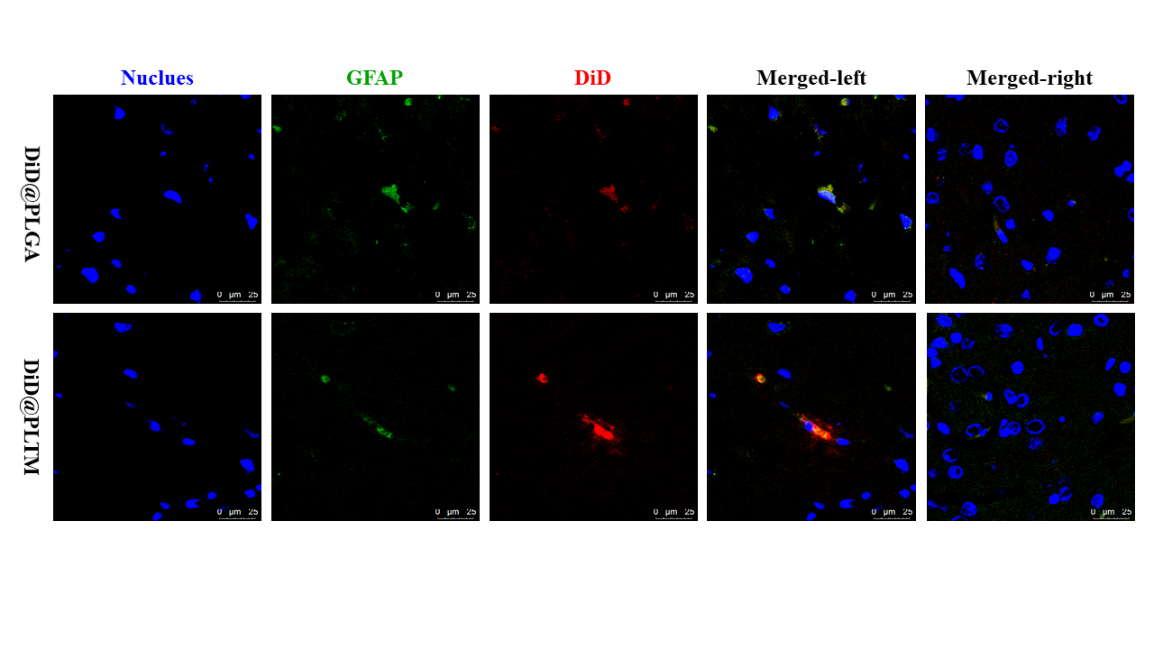

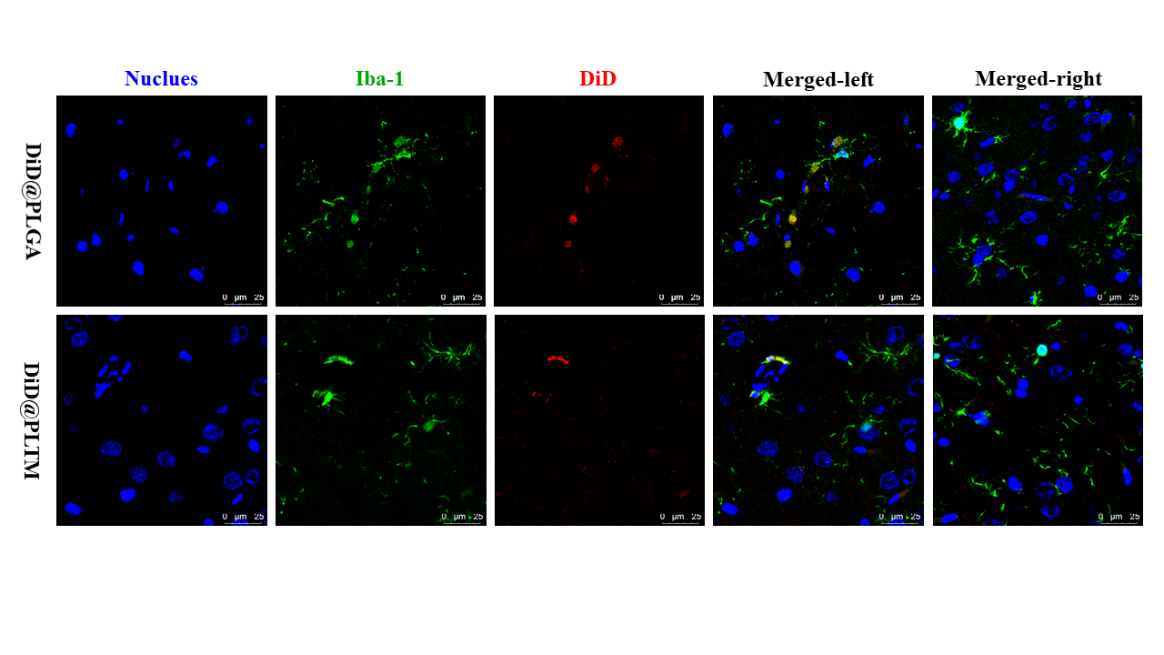

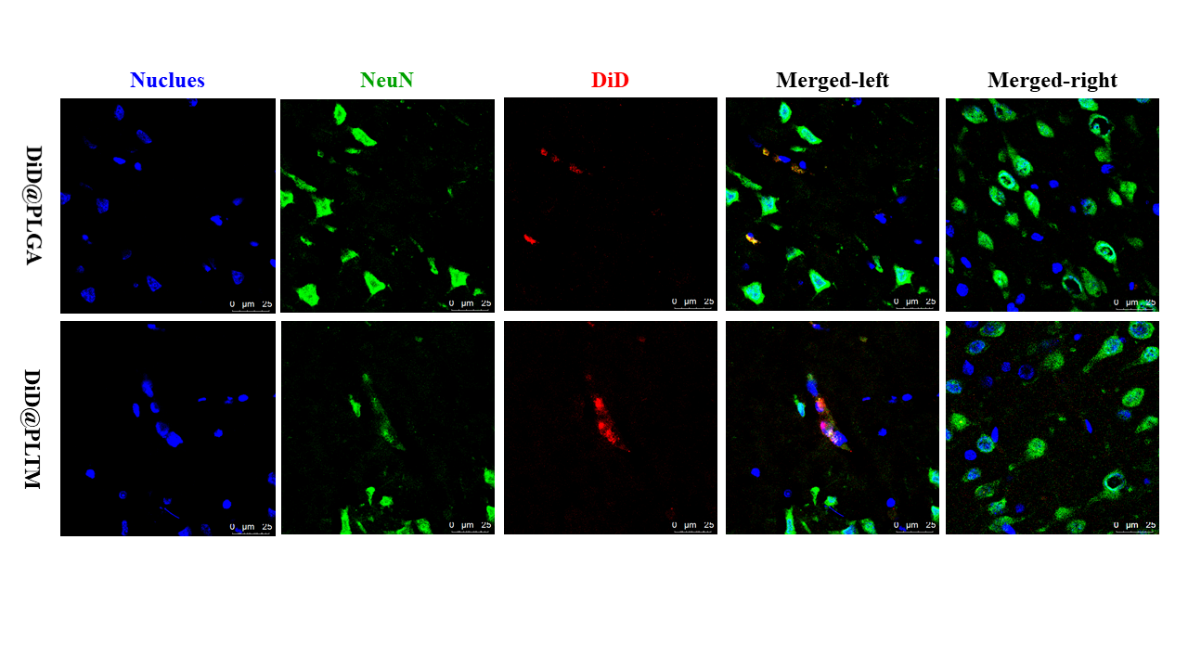

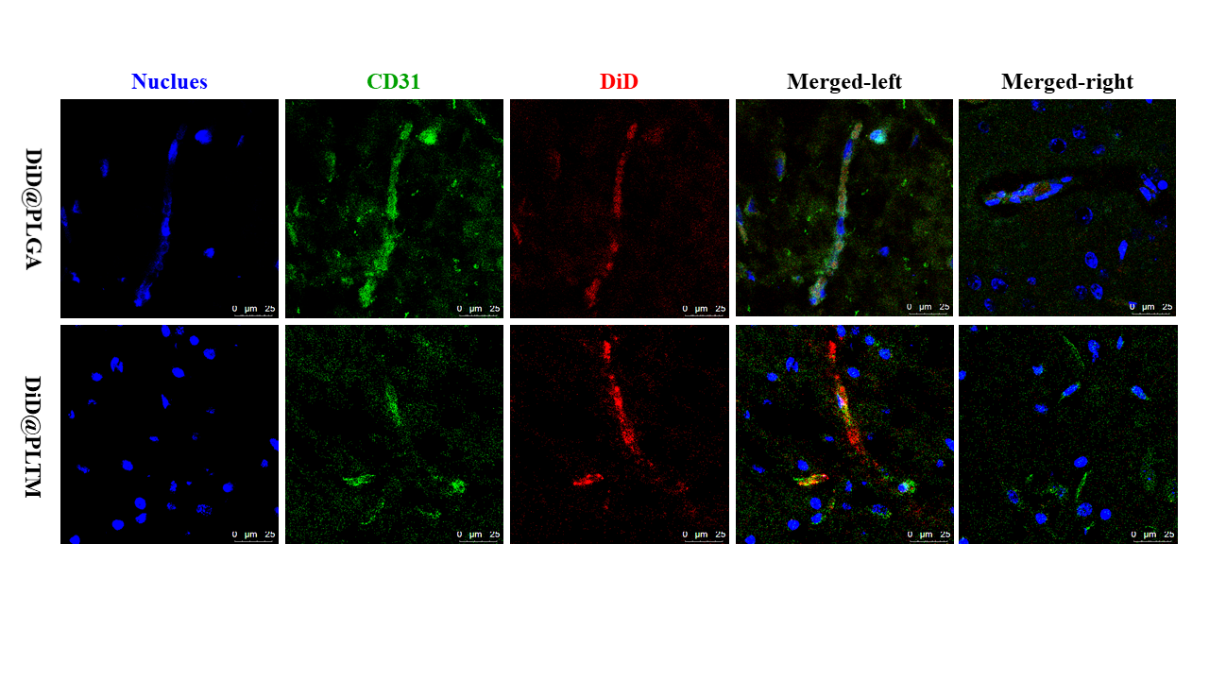


Fig. S9 2D confocal images of DiD-loading PLGA-NPs and PLTM-NPs co-localized with astrocytes, microglia, neurons and endothelial cells labeled by GFAP, Iba-1, NeuN and CD31 respectively in ischemic (left) and normal (right) cortex. Scale: 25 μm.


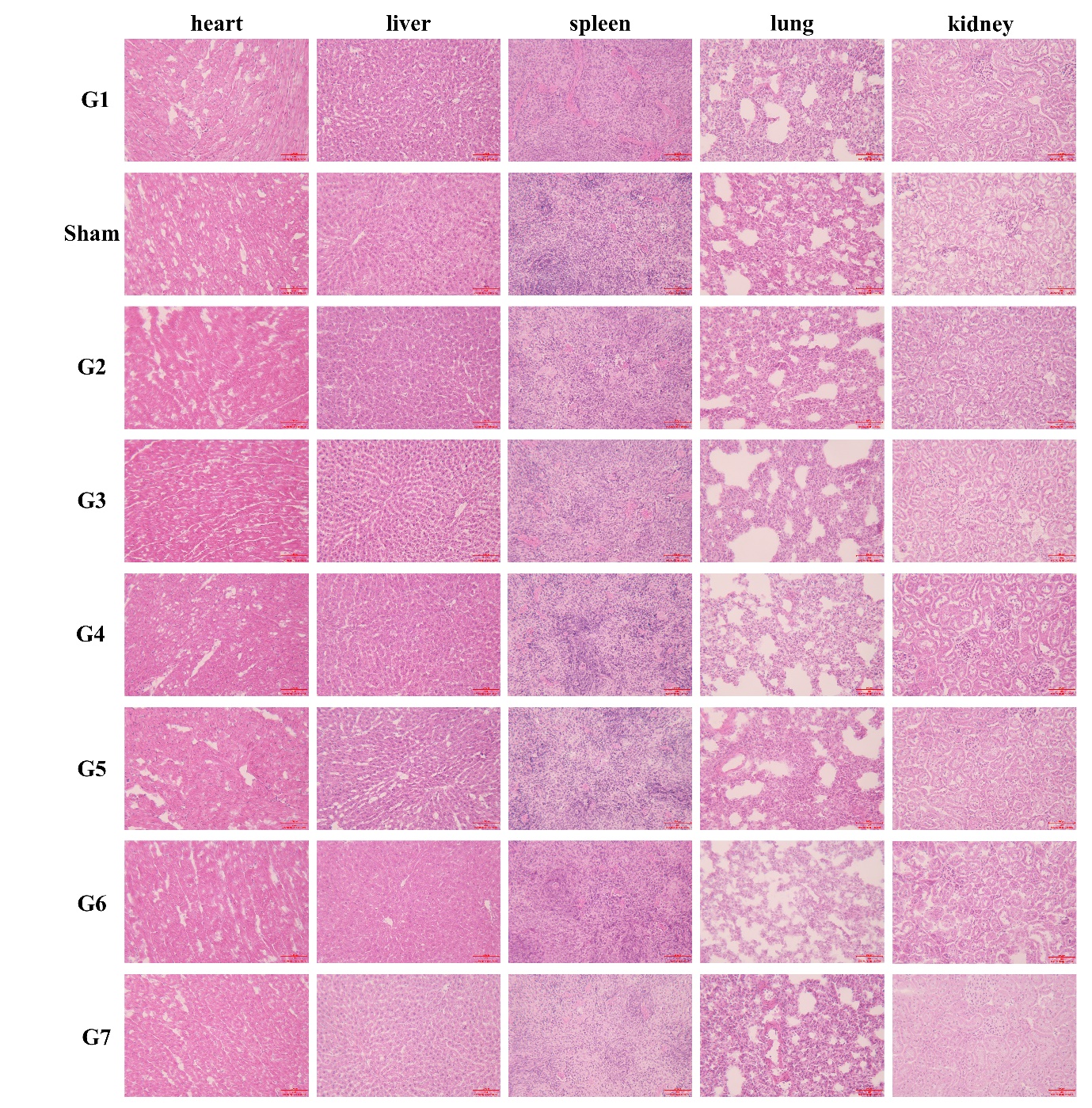


Fig. S10 H&E staining of main organs. Scale: 100 μm.
