# Supplementary figures and images for: Targeted strategy by curcumin and tideglusib biomimetic nano-systems alleviates oxidative stress and inflammation under ischemic stroke (part 1 of 3)
Source: Drug Deliv. 2025 Nov 25;32(1):2585599. doi: 10.1080/10717544.2025.2585599 (PMC12667299; doi:10.1080/10717544.2025.2585599)

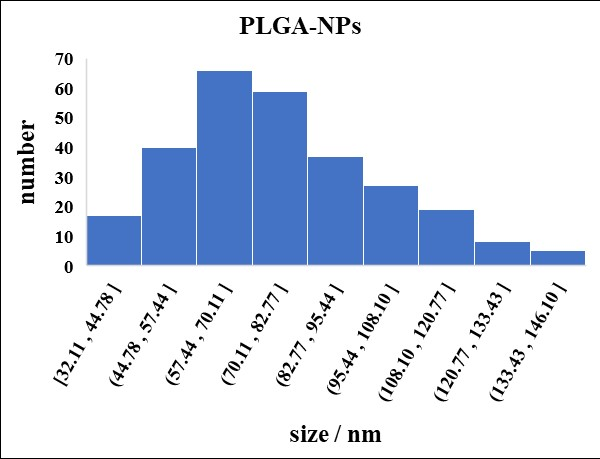

Supplement: Supplementary material — Original Images for Fig S1_Fig S9.zip [file IDRD_A_2585599_SM5400.zip › Original Image for Fig S1 (PLGA-NPs).tif]

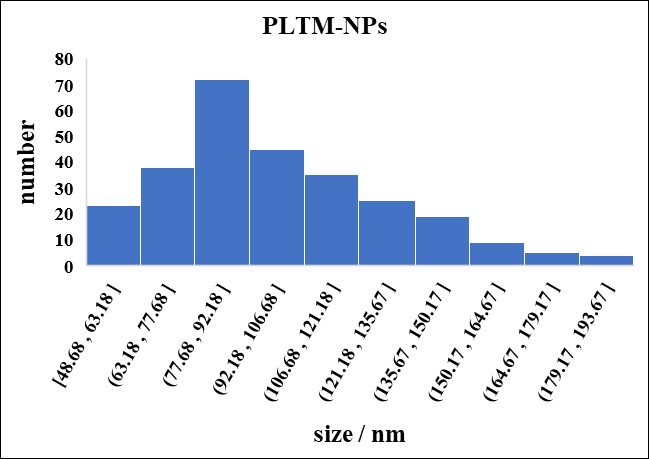

Supplement: Supplementary material — Original Images for Fig S1_Fig S9.zip [file IDRD_A_2585599_SM5400.zip › Original Image for Fig S1 (PLTM-NPs).tif]

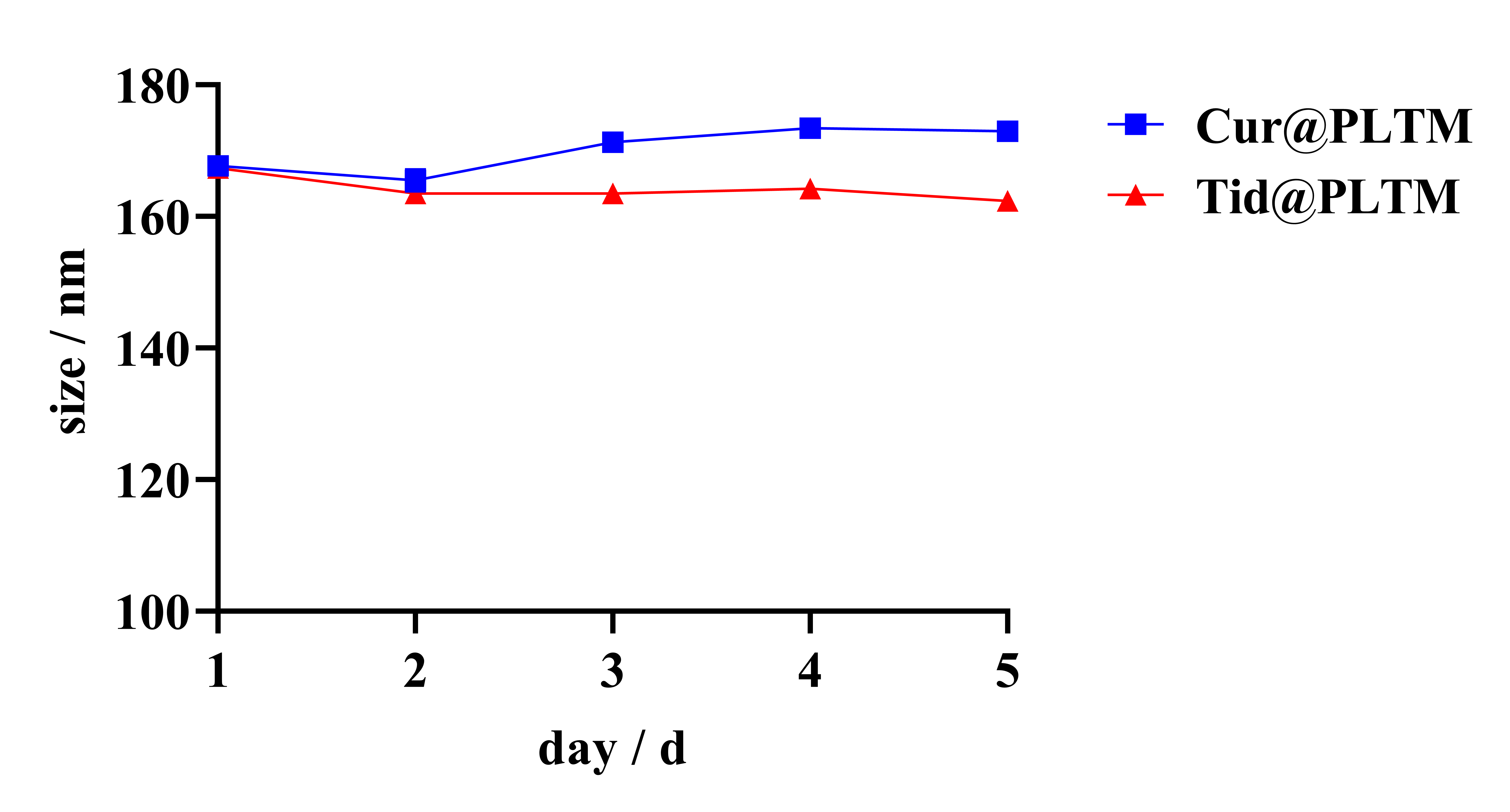

Supplement: Supplementary material — Original Images for Fig S1_Fig S9.zip [file IDRD_A_2585599_SM5400.zip › Original Image for Fig S2.tif]

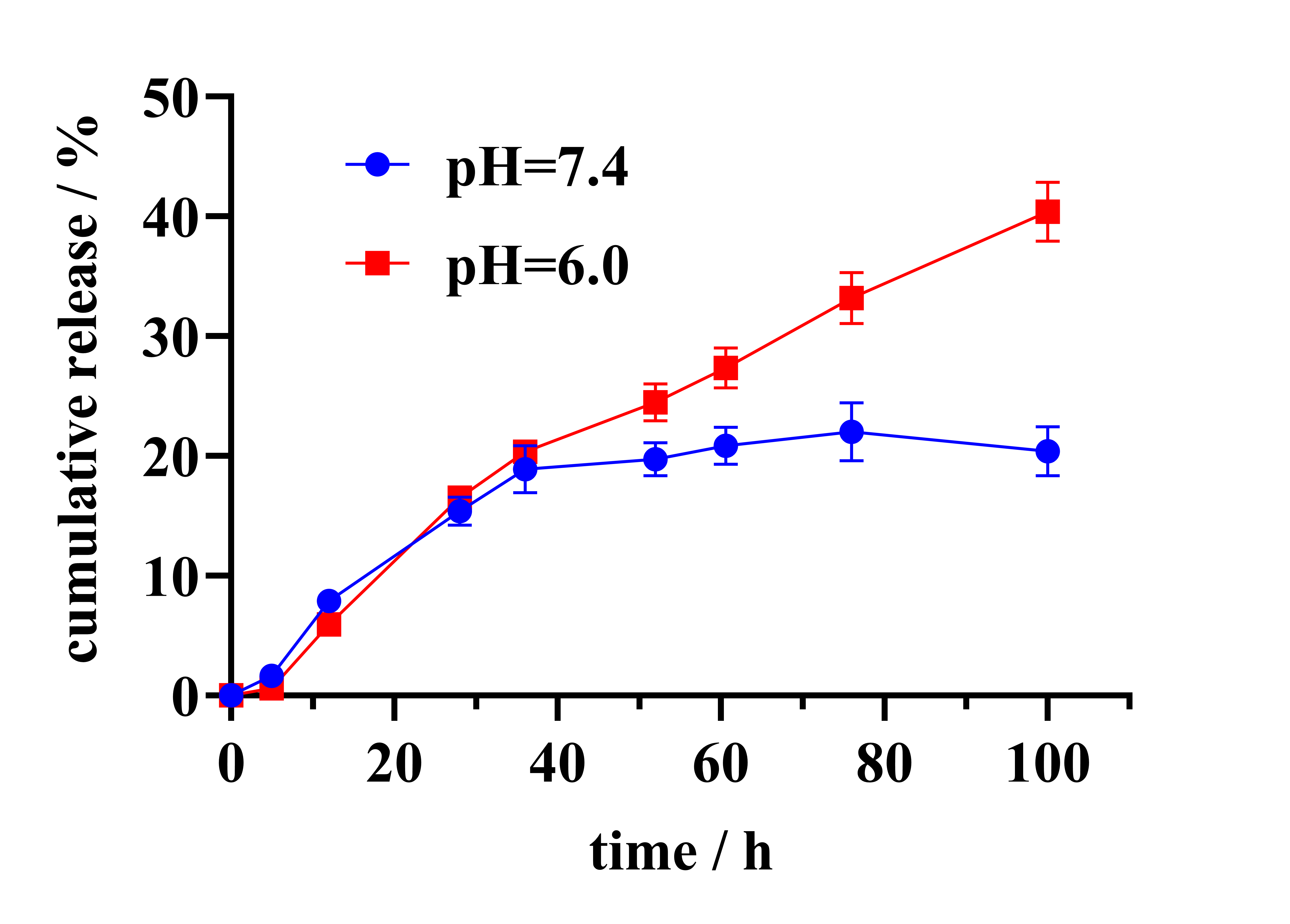

Supplement: Supplementary material — Original Images for Fig S1_Fig S9.zip [file IDRD_A_2585599_SM5400.zip › Original Image for Fig S3.tif]

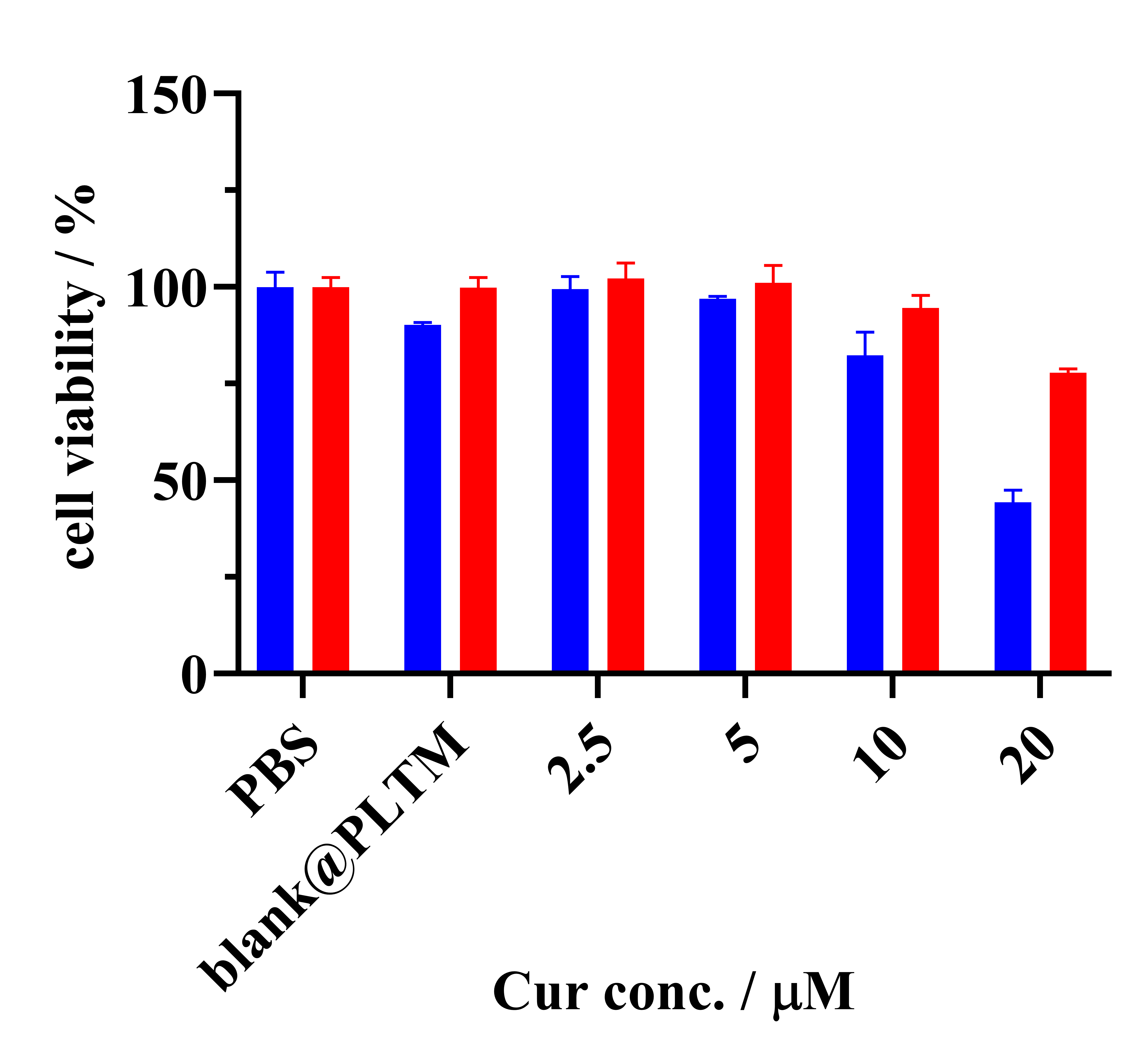

Supplement: Supplementary material — Original Images for Fig S1_Fig S9.zip [file IDRD_A_2585599_SM5400.zip › Original Image for Fig S4 (Cur@PLTM).tif]

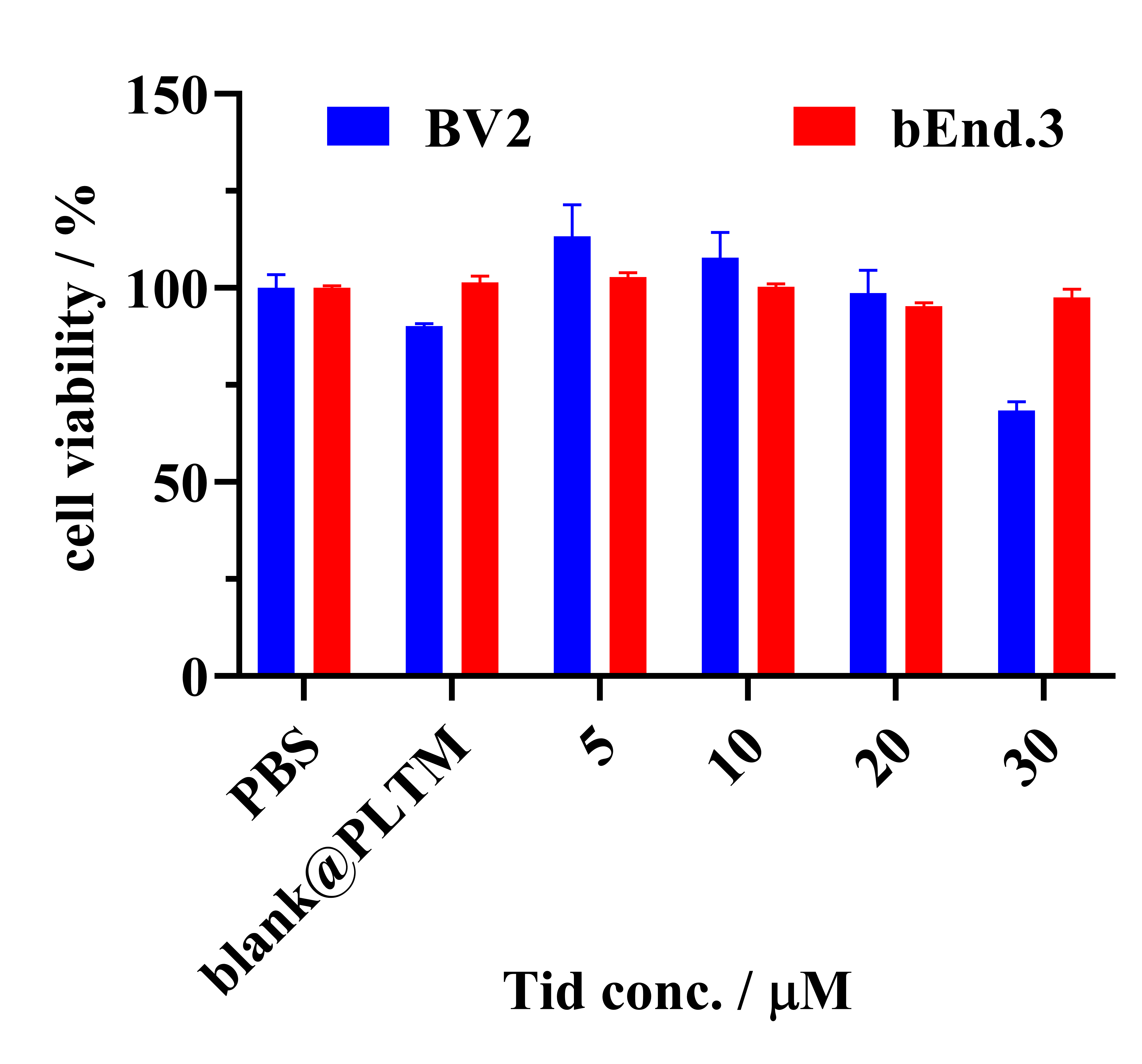

Supplement: Supplementary material — Original Images for Fig S1_Fig S9.zip [file IDRD_A_2585599_SM5400.zip › Original Image for Fig S4 (Tid@PLTM).tif]

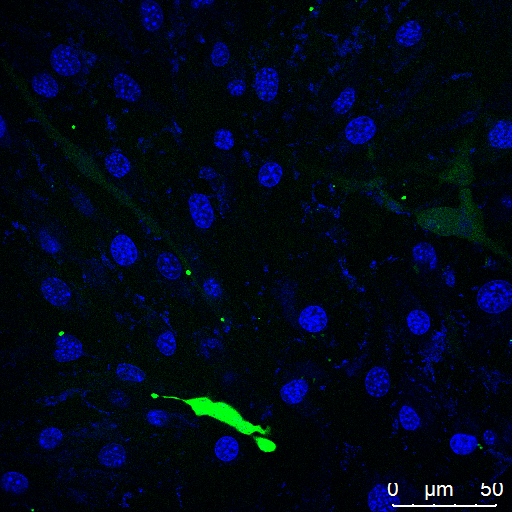

Supplement: Supplementary material — Original Images for Fig S1_Fig S9.zip [file IDRD_A_2585599_SM5400.zip › Original Image for Fig S5 bEnd.3 (2.5μM-merged).tif]

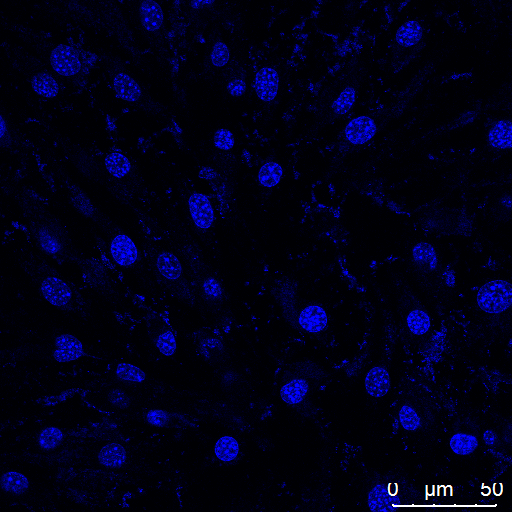

Supplement: Supplementary material — Original Images for Fig S1_Fig S9.zip [file IDRD_A_2585599_SM5400.zip › Original Image for Fig S5 bEnd.3 (2.5μM-nucleus).tif]

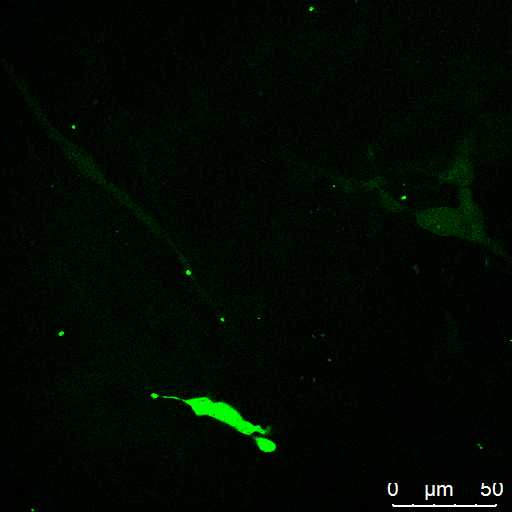

Supplement: Supplementary material — Original Images for Fig S1_Fig S9.zip [file IDRD_A_2585599_SM5400.zip › Original Image for Fig S5 bEnd.3 (2.5μM-ROS).tif]

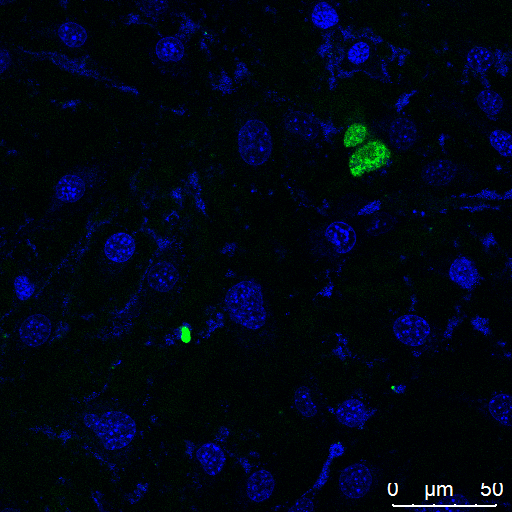

Supplement: Supplementary material — Original Images for Fig S1_Fig S9.zip [file IDRD_A_2585599_SM5400.zip › Original Image for Fig S5 bEnd.3 (5μM-merged).tif]

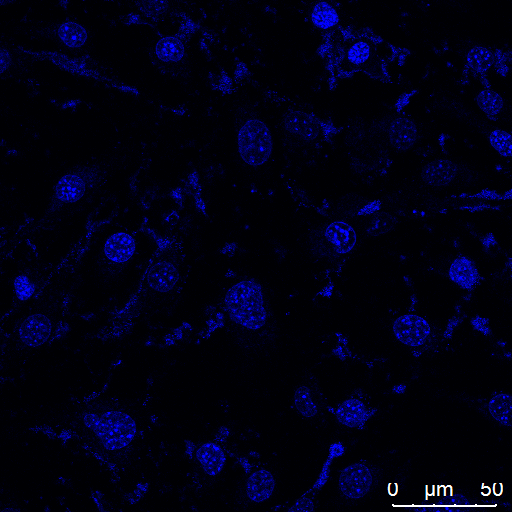

Supplement: Supplementary material — Original Images for Fig S1_Fig S9.zip [file IDRD_A_2585599_SM5400.zip › Original Image for Fig S5 bEnd.3 (5μM-nucleus).tif]

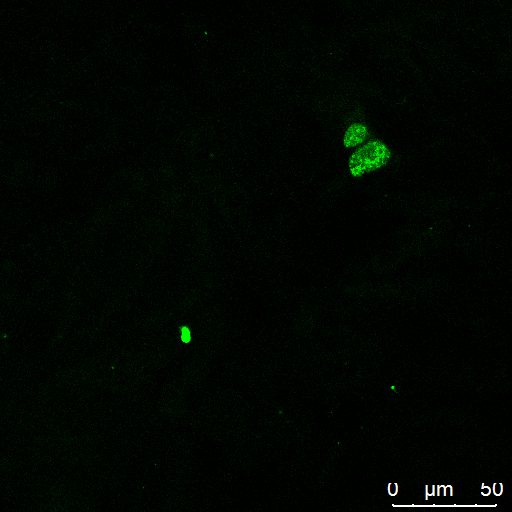

Supplement: Supplementary material — Original Images for Fig S1_Fig S9.zip [file IDRD_A_2585599_SM5400.zip › Original Image for Fig S5 bEnd.3 (5μM-ROS).tif]

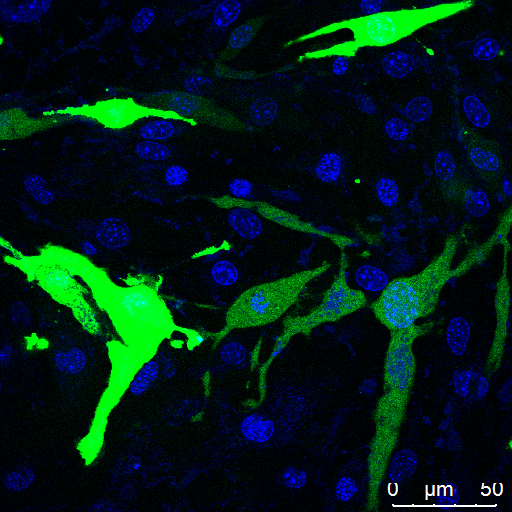

Supplement: Supplementary material — Original Images for Fig S1_Fig S9.zip [file IDRD_A_2585599_SM5400.zip › Original Image for Fig S5 bEnd.3 (PBS-merged).tif]

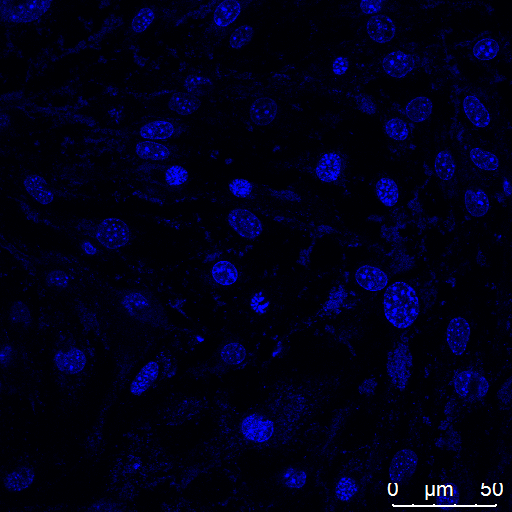

Supplement: Supplementary material — Original Images for Fig S1_Fig S9.zip [file IDRD_A_2585599_SM5400.zip › Original Image for Fig S5 bEnd.3 (PBS-nucleus).tif]

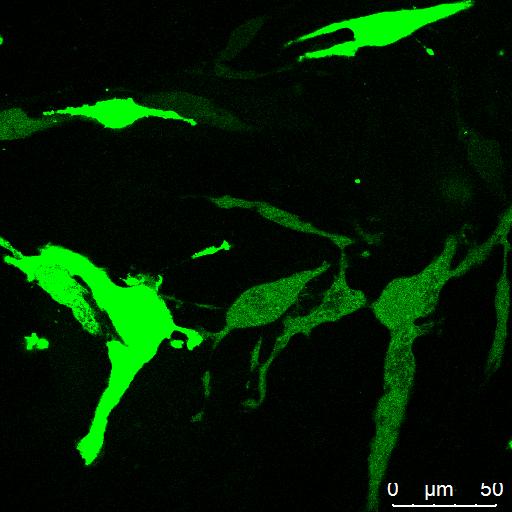

Supplement: Supplementary material — Original Images for Fig S1_Fig S9.zip [file IDRD_A_2585599_SM5400.zip › Original Image for Fig S5 bEnd.3 (PBS-ROS).tif]

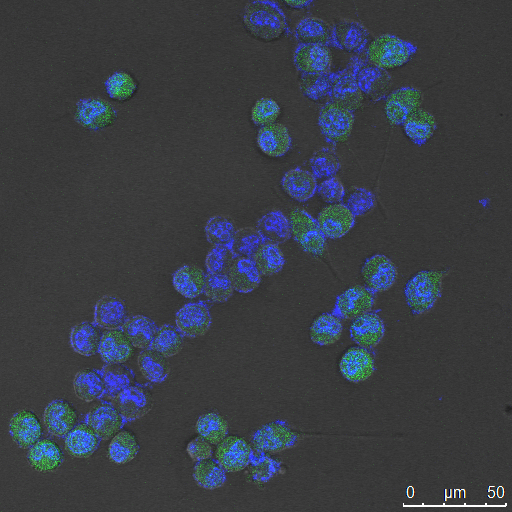

Supplement: Supplementary material — Original Images for Fig S1_Fig S9.zip [file IDRD_A_2585599_SM5400.zip › Original Image for Fig S5 BV2 (2.5μM-merged).tif]

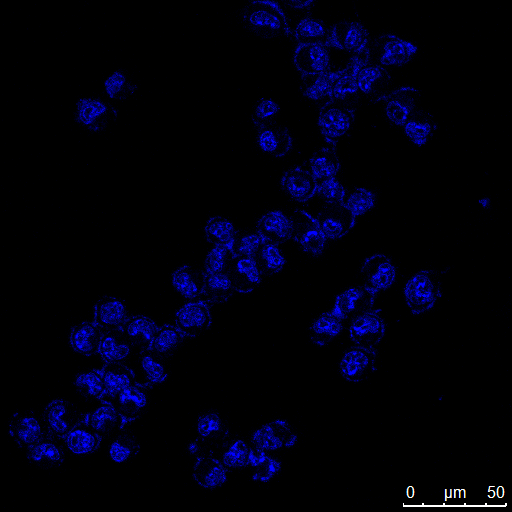

Supplement: Supplementary material — Original Images for Fig S1_Fig S9.zip [file IDRD_A_2585599_SM5400.zip › Original Image for Fig S5 BV2 (2.5μM-nucleus).tif]

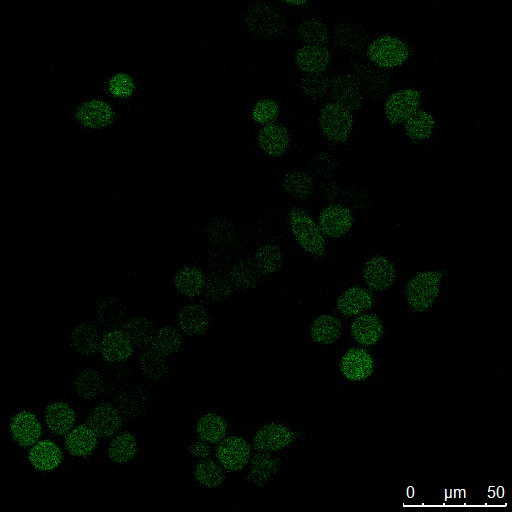

Supplement: Supplementary material — Original Images for Fig S1_Fig S9.zip [file IDRD_A_2585599_SM5400.zip › Original Image for Fig S5 BV2 (2.5μM-ROS).tif]

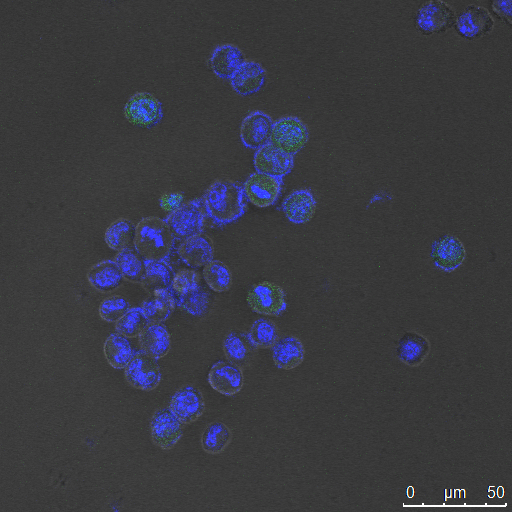

Supplement: Supplementary material — Original Images for Fig S1_Fig S9.zip [file IDRD_A_2585599_SM5400.zip › Original Image for Fig S5 BV2 (5μM-merged).tif]

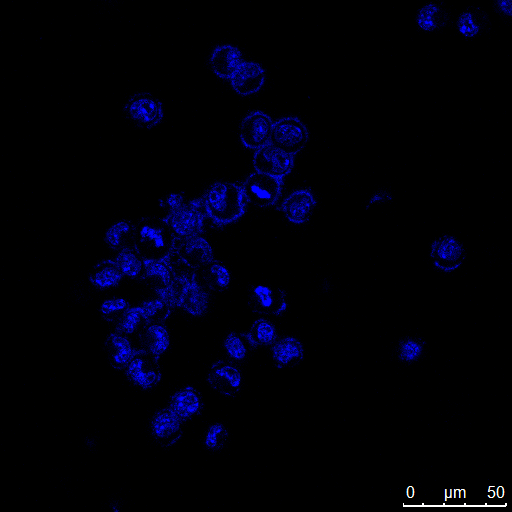

Supplement: Supplementary material — Original Images for Fig S1_Fig S9.zip [file IDRD_A_2585599_SM5400.zip › Original Image for Fig S5 BV2 (5μM-nucleus).tif]

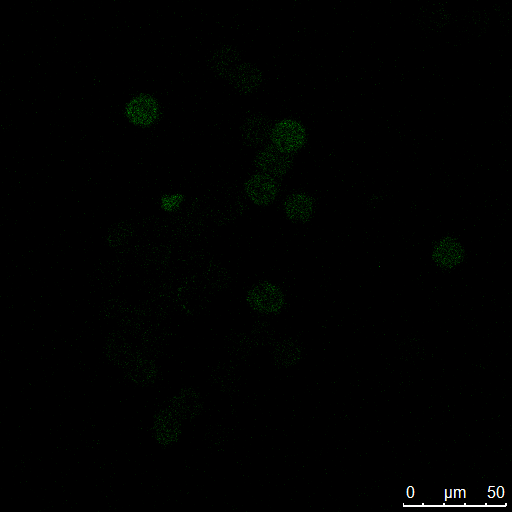

Supplement: Supplementary material — Original Images for Fig S1_Fig S9.zip [file IDRD_A_2585599_SM5400.zip › Original Image for Fig S5 BV2 (5μM-ROS).tif]

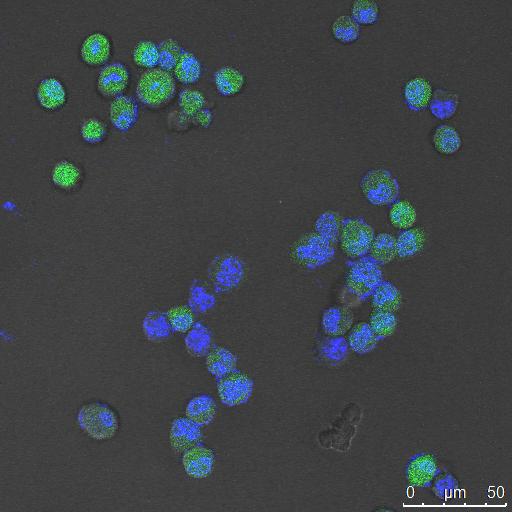

Supplement: Supplementary material — Original Images for Fig S1_Fig S9.zip [file IDRD_A_2585599_SM5400.zip › Original Image for Fig S5 BV2 (PBS-merged).tif]

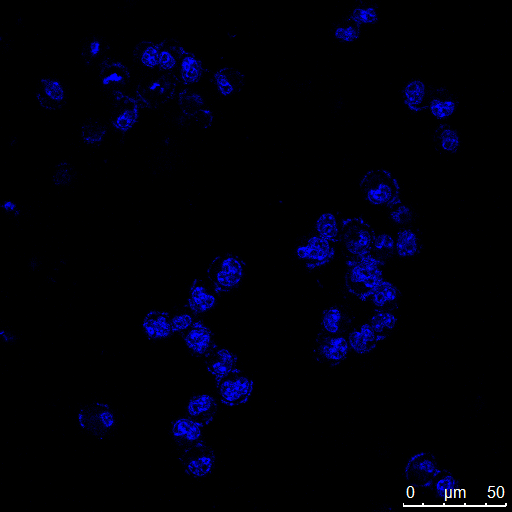

Supplement: Supplementary material — Original Images for Fig S1_Fig S9.zip [file IDRD_A_2585599_SM5400.zip › Original Image for Fig S5 BV2 (PBS-nucleus).tif]

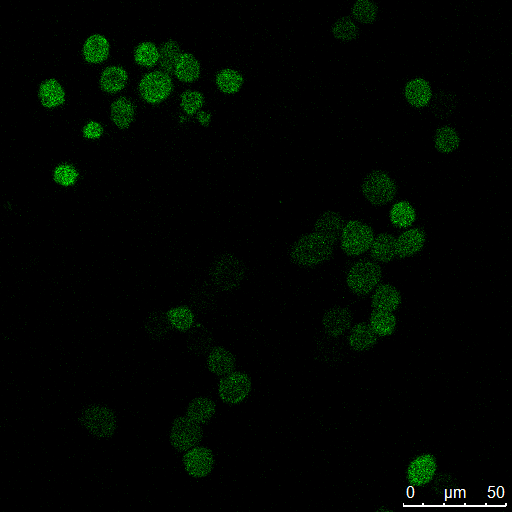

Supplement: Supplementary material — Original Images for Fig S1_Fig S9.zip [file IDRD_A_2585599_SM5400.zip › Original Image for Fig S5 BV2 (PBS-ROS).tif]

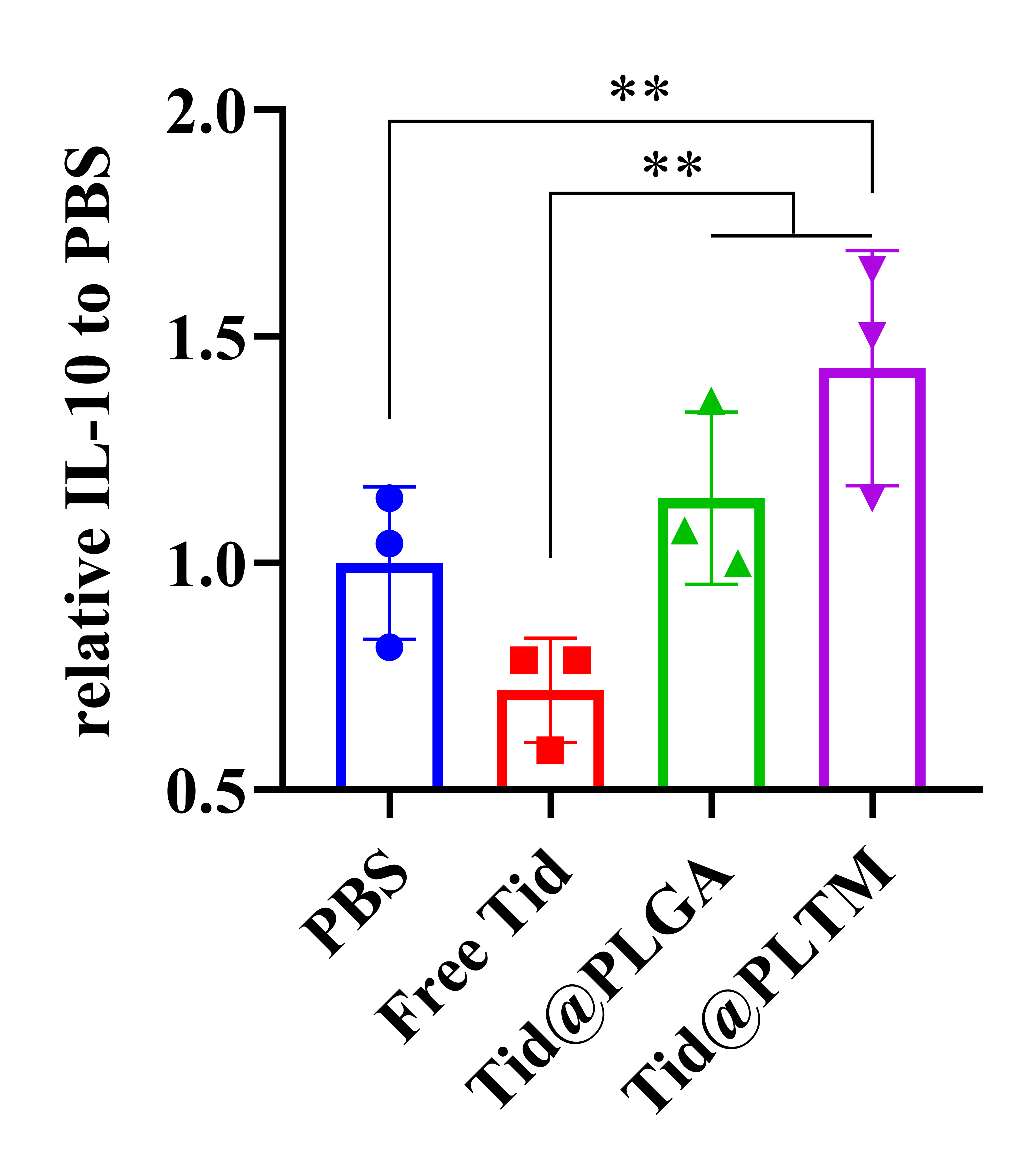

Supplement: Supplementary material — Original Images for Fig S1_Fig S9.zip [file IDRD_A_2585599_SM5400.zip › Original Image for Fig S6 (IL-10).tif]

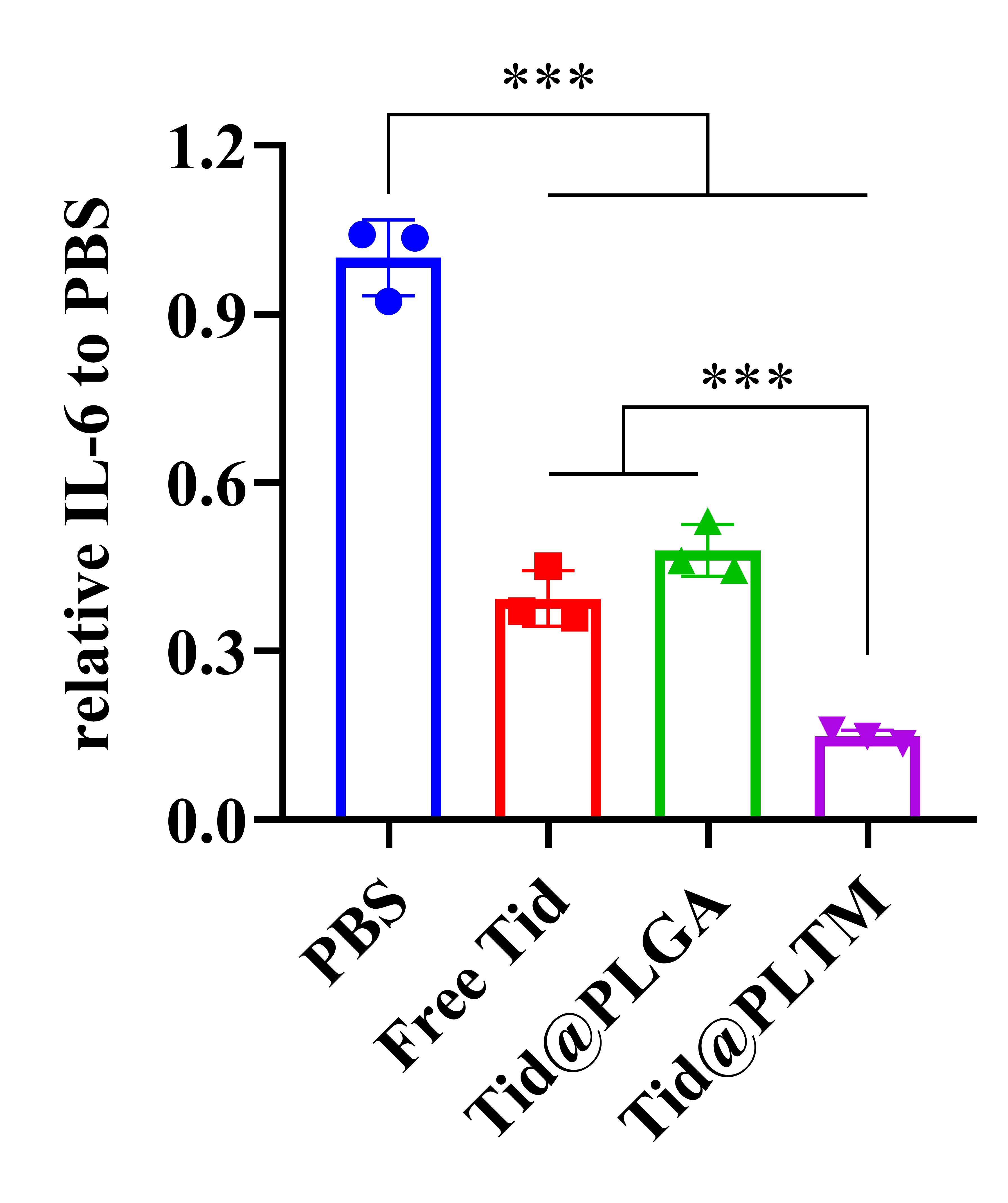

Supplement: Supplementary material — Original Images for Fig S1_Fig S9.zip [file IDRD_A_2585599_SM5400.zip › Original Image for Fig S6 (IL-6).tif]

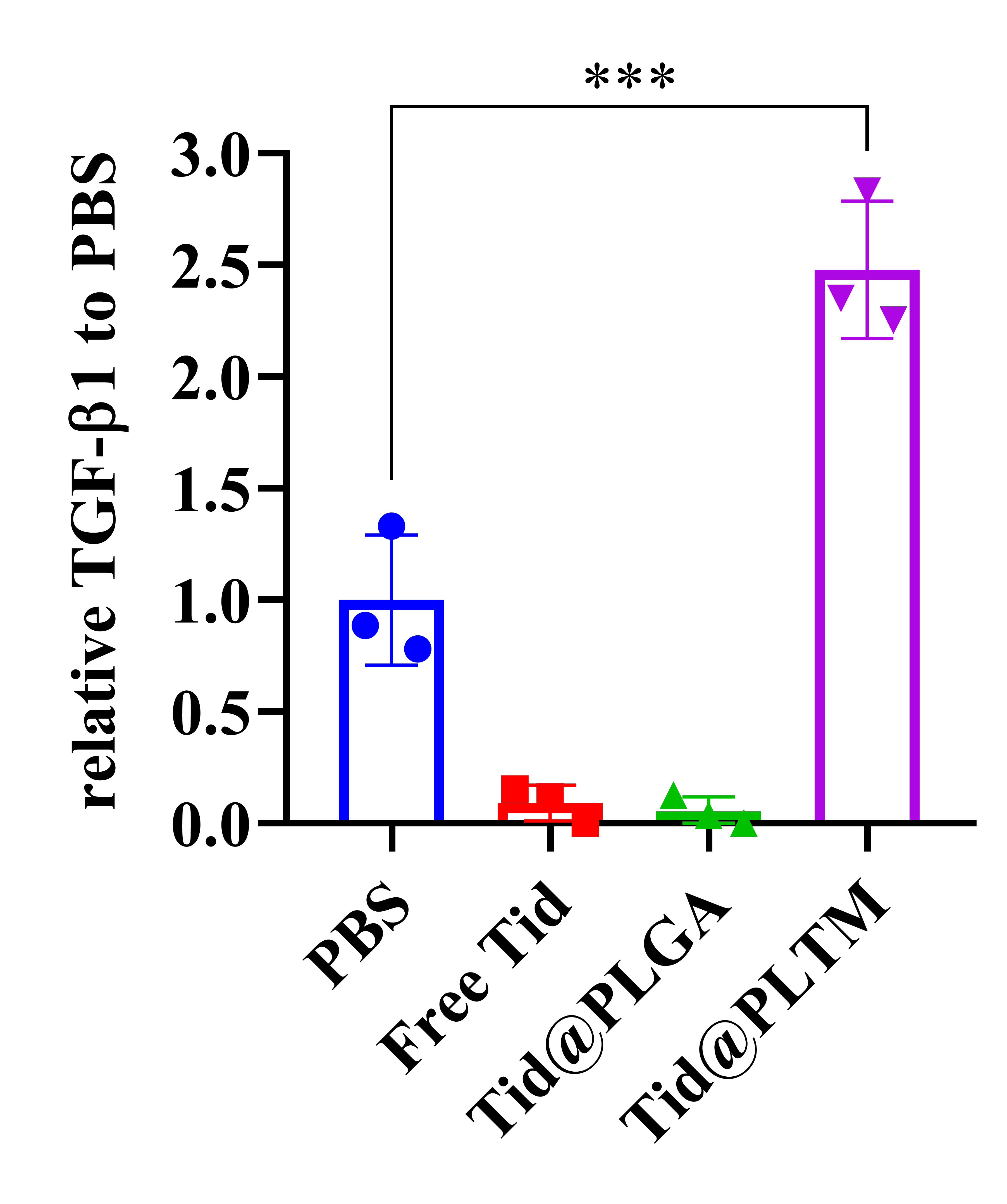

Supplement: Supplementary material — Original Images for Fig S1_Fig S9.zip [file IDRD_A_2585599_SM5400.zip › Original Image for Fig S6 (TGF-β1).tif]

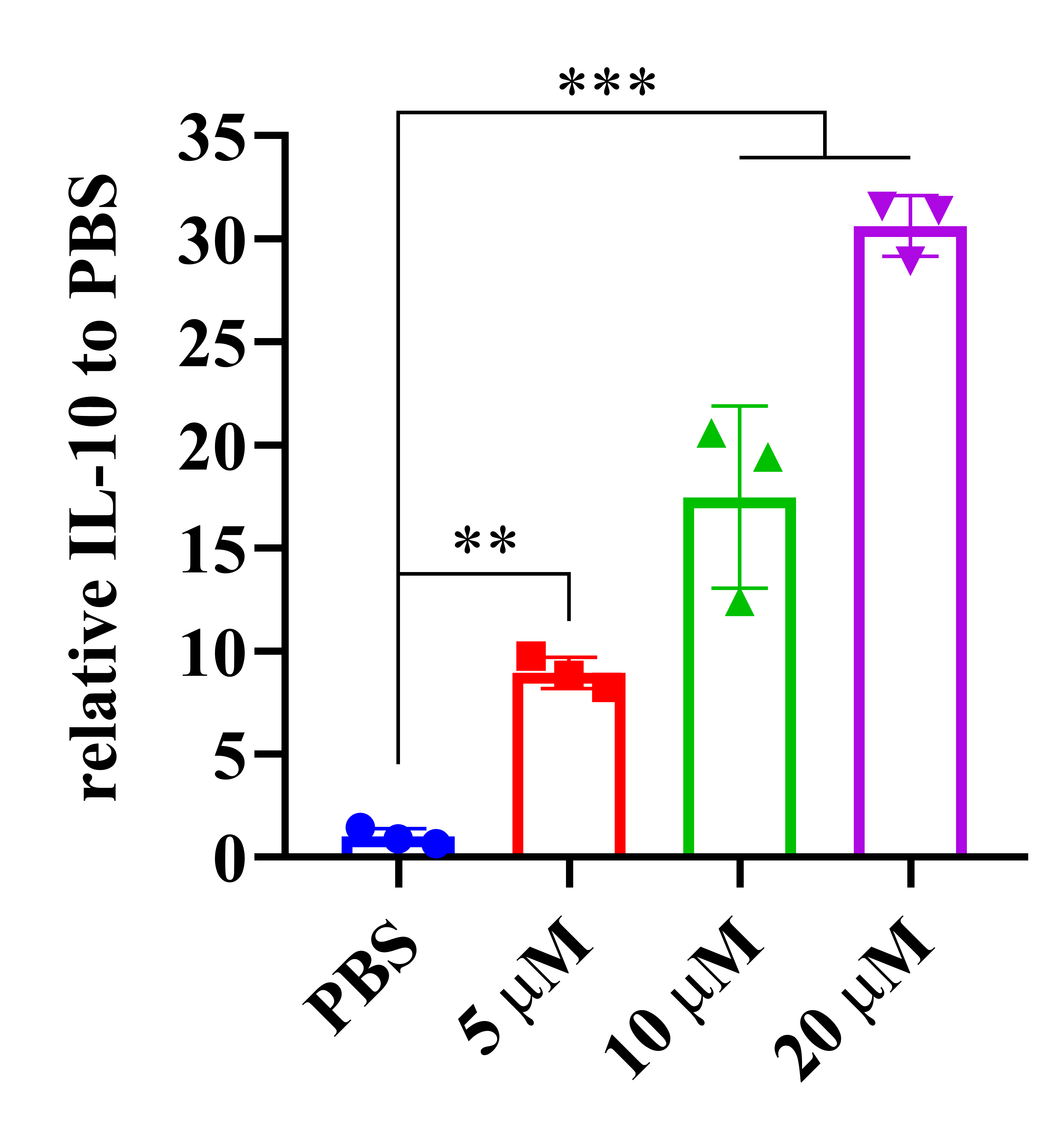

Supplement: Supplementary material — Original Images for Fig S1_Fig S9.zip [file IDRD_A_2585599_SM5400.zip › Original Image for Fig S7 (IL-10).tif]

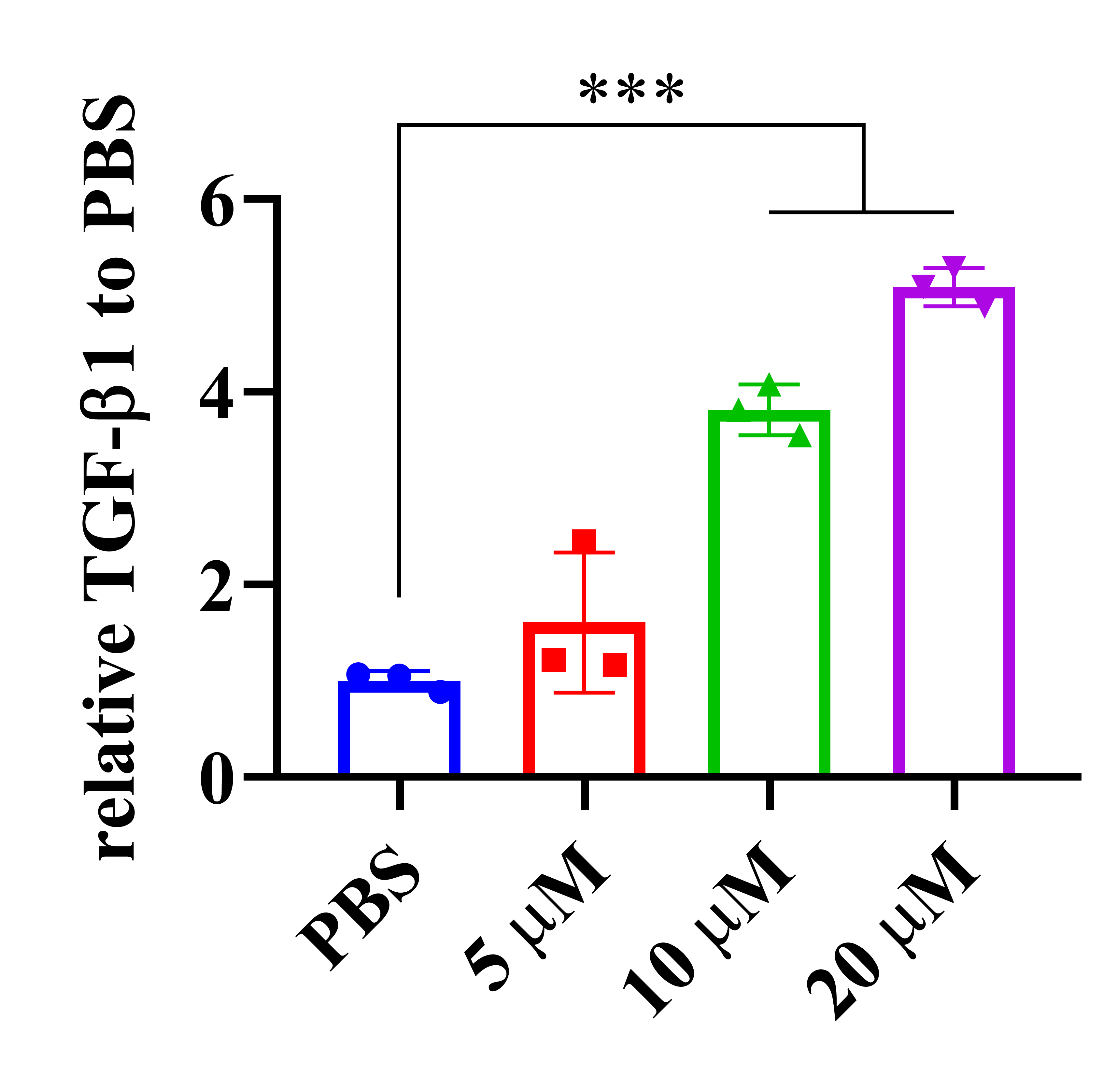

Supplement: Supplementary material — Original Images for Fig S1_Fig S9.zip [file IDRD_A_2585599_SM5400.zip › Original Image for Fig S7 (TGF-β1).tif]

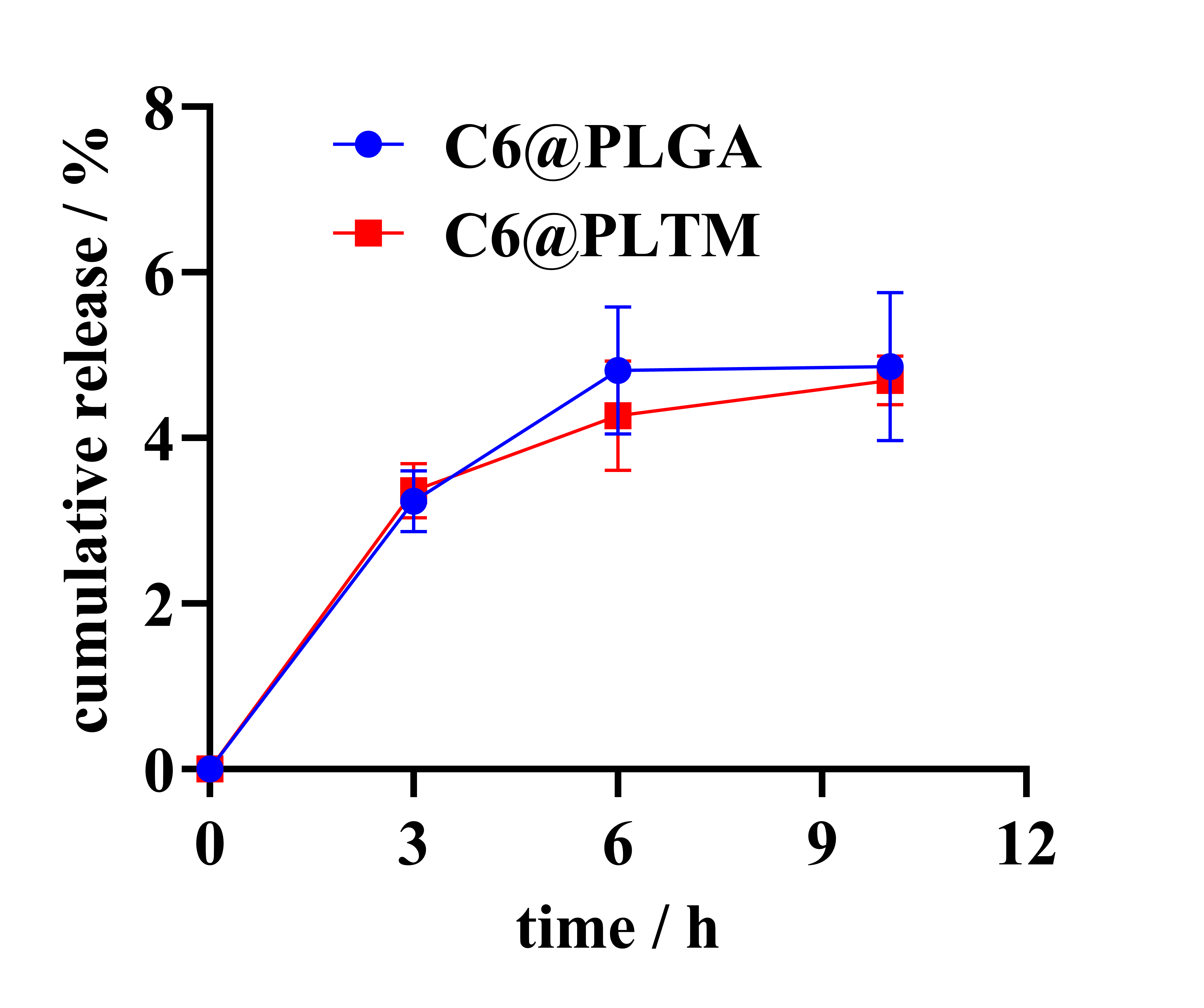

Supplement: Supplementary material — Original Images for Fig S1_Fig S9.zip [file IDRD_A_2585599_SM5400.zip › Original Image for Fig S8.tif]

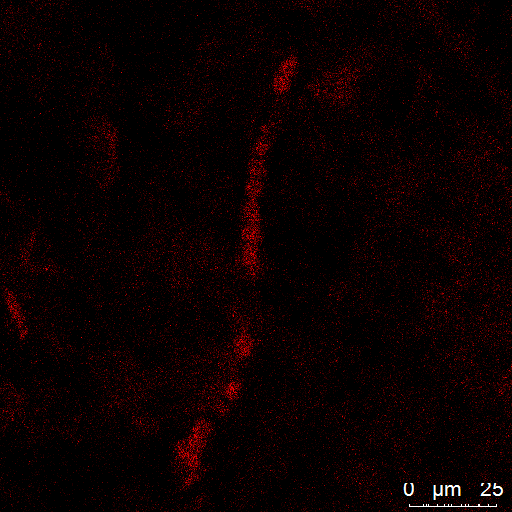

Supplement: Supplementary material — Original Images for Fig S1_Fig S9.zip [file IDRD_A_2585599_SM5400.zip › Original Image for Fig S9 DiD@PLGA left (CD31-DiD).tif]

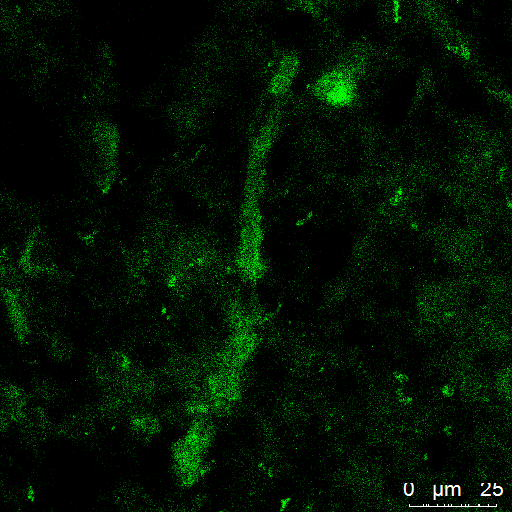

Supplement: Supplementary material — Original Images for Fig S1_Fig S9.zip [file IDRD_A_2585599_SM5400.zip › Original Image for Fig S9 DiD@PLGA left (CD31-marker).tif]

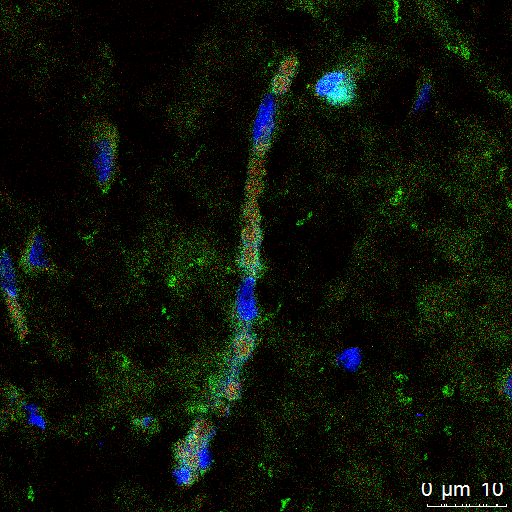

Supplement: Supplementary material — Original Images for Fig S1_Fig S9.zip [file IDRD_A_2585599_SM5400.zip › Original Image for Fig S9 DiD@PLGA left (CD31-merged).tif]

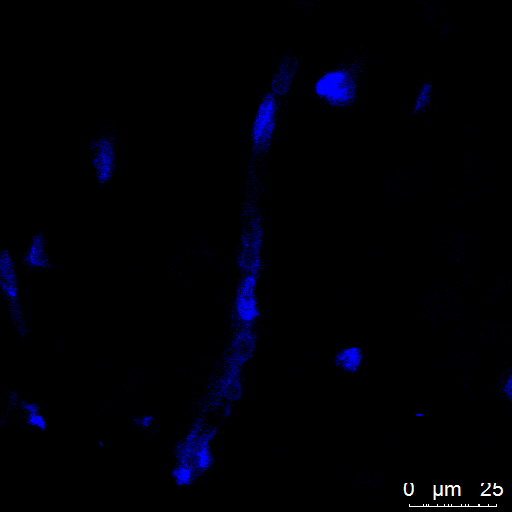

Supplement: Supplementary material — Original Images for Fig S1_Fig S9.zip [file IDRD_A_2585599_SM5400.zip › Original Image for Fig S9 DiD@PLGA left (CD31-nucleus).tif]

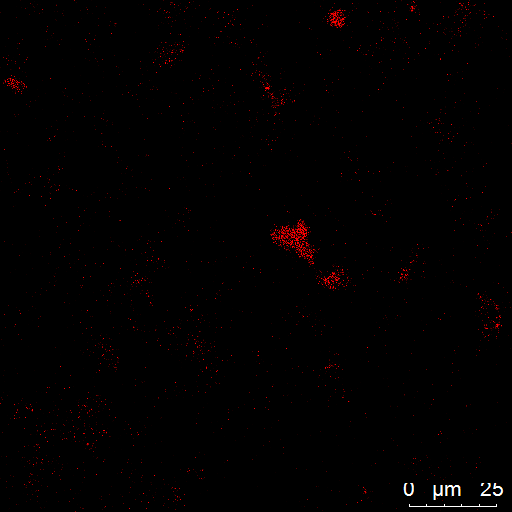

Supplement: Supplementary material — Original Images for Fig S1_Fig S9.zip [file IDRD_A_2585599_SM5400.zip › Original Image for Fig S9 DiD@PLGA left (GFAP-DiD).tif]

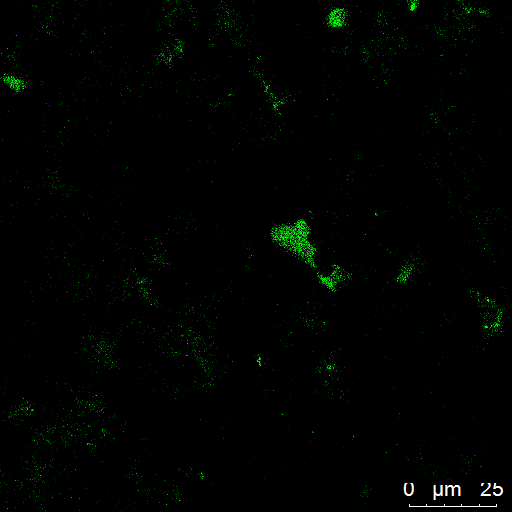

Supplement: Supplementary material — Original Images for Fig S1_Fig S9.zip [file IDRD_A_2585599_SM5400.zip › Original Image for Fig S9 DiD@PLGA left (GFAP-marker).tif]

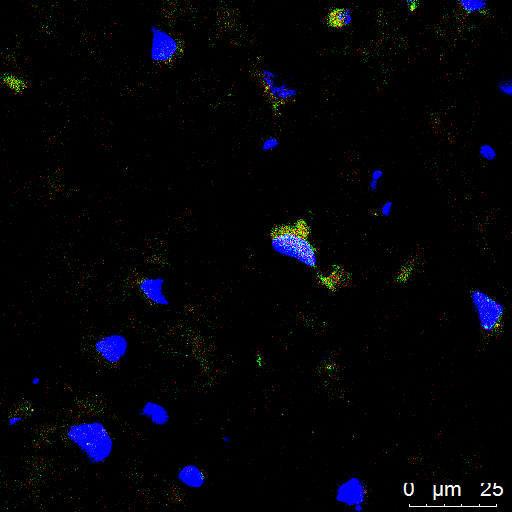

Supplement: Supplementary material — Original Images for Fig S1_Fig S9.zip [file IDRD_A_2585599_SM5400.zip › Original Image for Fig S9 DiD@PLGA left (GFAP-merged).tif]

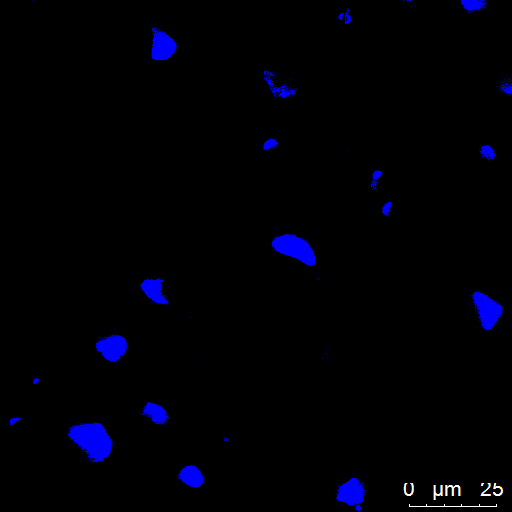

Supplement: Supplementary material — Original Images for Fig S1_Fig S9.zip [file IDRD_A_2585599_SM5400.zip › Original Image for Fig S9 DiD@PLGA left (GFAP-nucleus).tif]

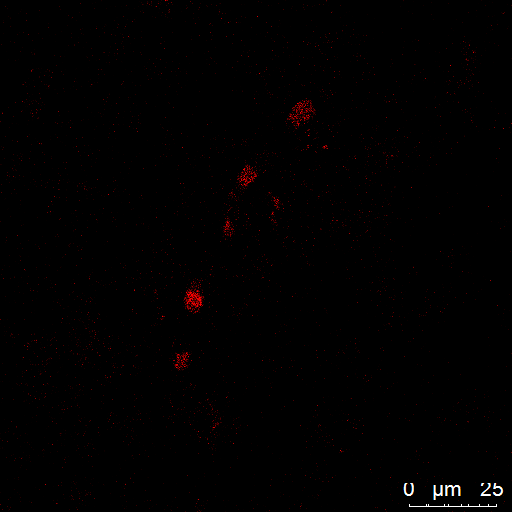

Supplement: Supplementary material — Original Images for Fig S1_Fig S9.zip [file IDRD_A_2585599_SM5400.zip › Original Image for Fig S9 DiD@PLGA left (Iba-1-DiD).tif]

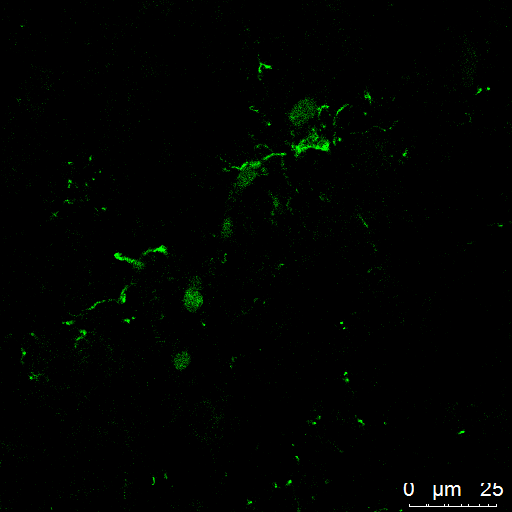

Supplement: Supplementary material — Original Images for Fig S1_Fig S9.zip [file IDRD_A_2585599_SM5400.zip › Original Image for Fig S9 DiD@PLGA left (Iba-1-marker).tif]

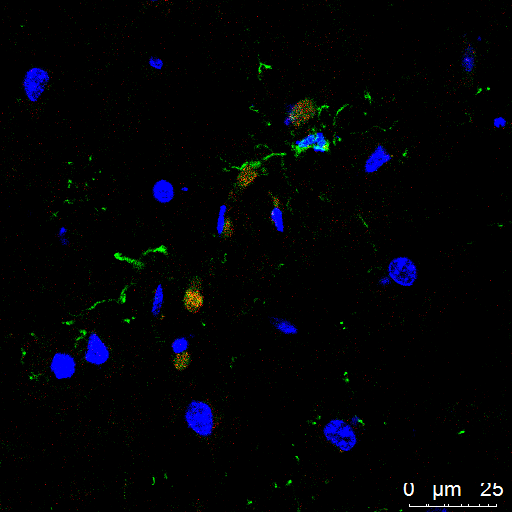

Supplement: Supplementary material — Original Images for Fig S1_Fig S9.zip [file IDRD_A_2585599_SM5400.zip › Original Image for Fig S9 DiD@PLGA left (Iba-1-merged).tif]

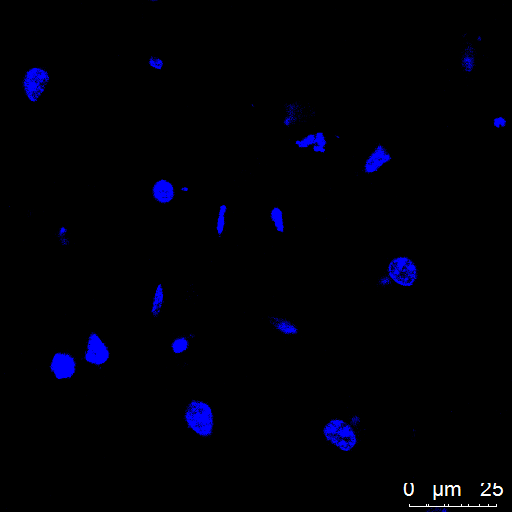

Supplement: Supplementary material — Original Images for Fig S1_Fig S9.zip [file IDRD_A_2585599_SM5400.zip › Original Image for Fig S9 DiD@PLGA left (Iba-1-nucleus).tif]

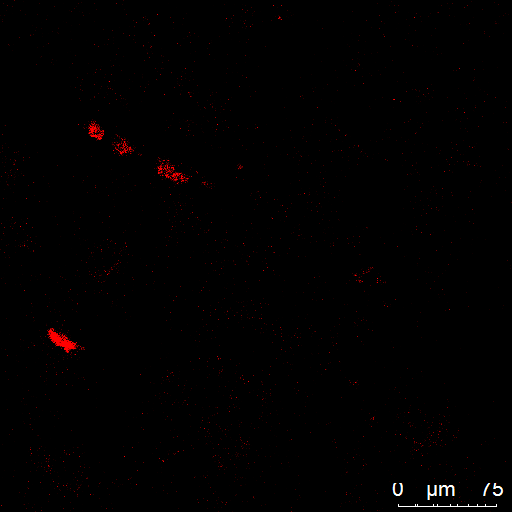

Supplement: Supplementary material — Original Images for Fig S1_Fig S9.zip [file IDRD_A_2585599_SM5400.zip › Original Image for Fig S9 DiD@PLGA left (NeuN-DiD).tif]

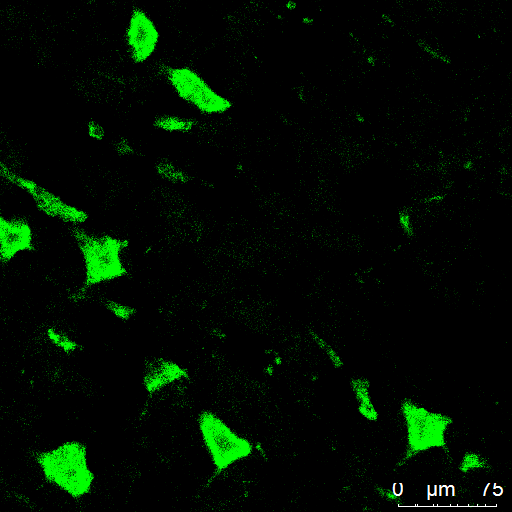

Supplement: Supplementary material — Original Images for Fig S1_Fig S9.zip [file IDRD_A_2585599_SM5400.zip › Original Image for Fig S9 DiD@PLGA left (NeuN-marker).tif]

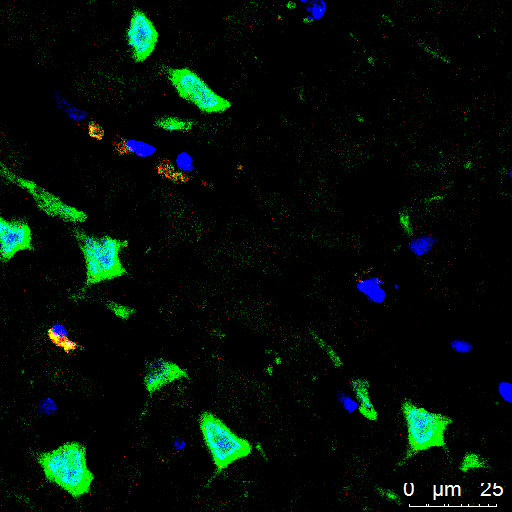

Supplement: Supplementary material — Original Images for Fig S1_Fig S9.zip [file IDRD_A_2585599_SM5400.zip › Original Image for Fig S9 DiD@PLGA left (NeuN-merged).tif]

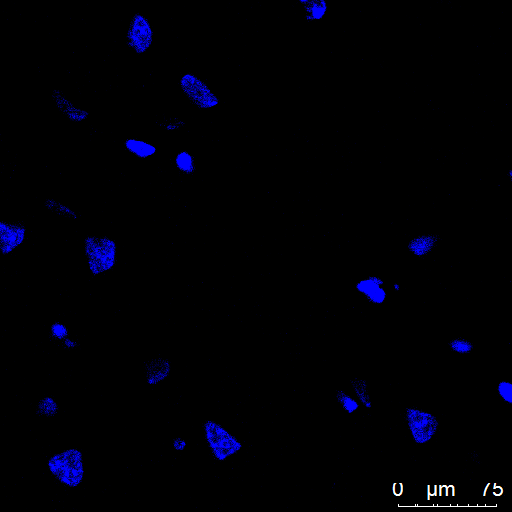

Supplement: Supplementary material — Original Images for Fig S1_Fig S9.zip [file IDRD_A_2585599_SM5400.zip › Original Image for Fig S9 DiD@PLGA left (NeuN-nucleus).tif]

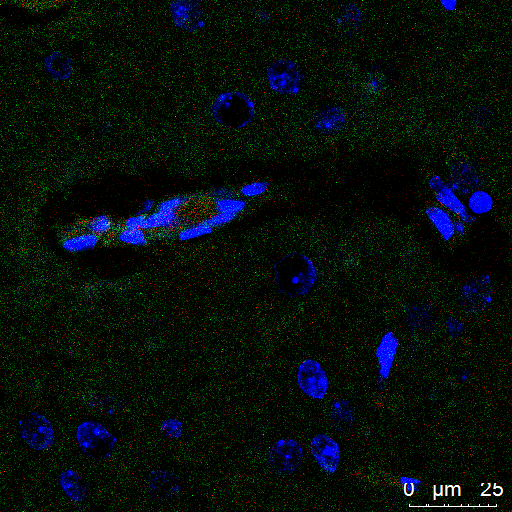

Supplement: Supplementary material — Original Images for Fig S1_Fig S9.zip [file IDRD_A_2585599_SM5400.zip › Original Image for Fig S9 DiD@PLGA right (CD31).tif]

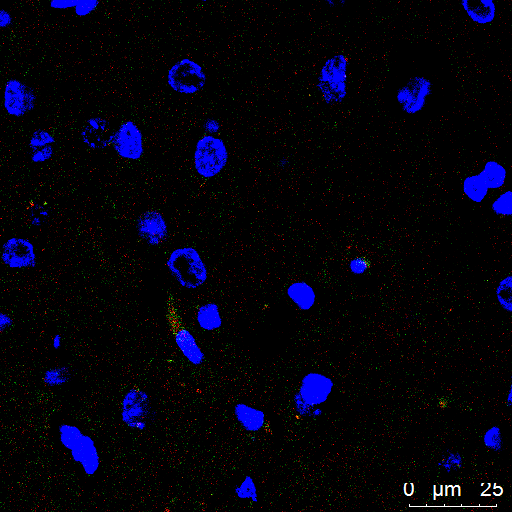

Supplement: Supplementary material — Original Images for Fig S1_Fig S9.zip [file IDRD_A_2585599_SM5400.zip › Original Image for Fig S9 DiD@PLGA right (GFAP).tif]

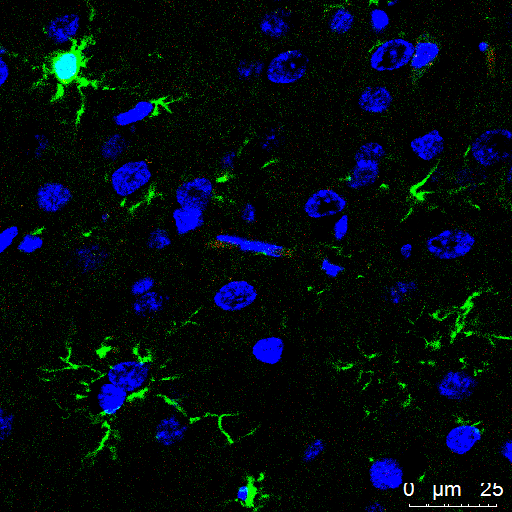

Supplement: Supplementary material — Original Images for Fig S1_Fig S9.zip [file IDRD_A_2585599_SM5400.zip › Original Image for Fig S9 DiD@PLGA right (Iba-1).tif]

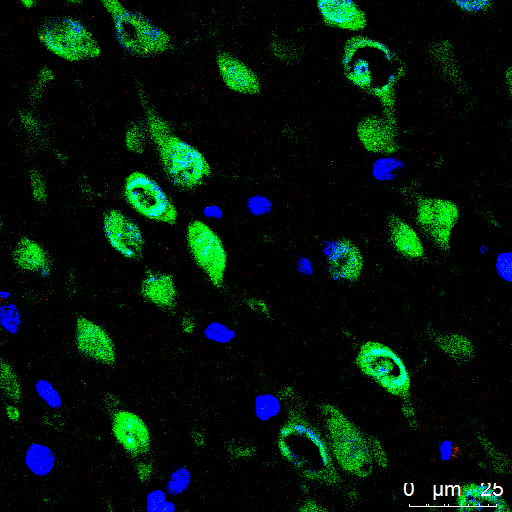

Supplement: Supplementary material — Original Images for Fig S1_Fig S9.zip [file IDRD_A_2585599_SM5400.zip › Original Image for Fig S9 DiD@PLGA right (NeuN).tif]

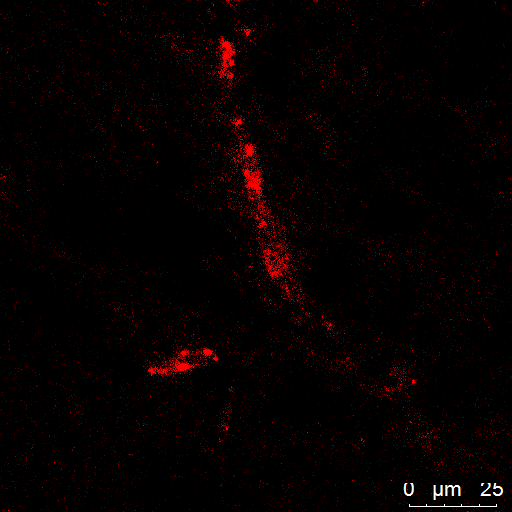

Supplement: Supplementary material — Original Images for Fig S1_Fig S9.zip [file IDRD_A_2585599_SM5400.zip › Original Image for Fig S9 DiD@PLTM left (CD31-DiD).tif]

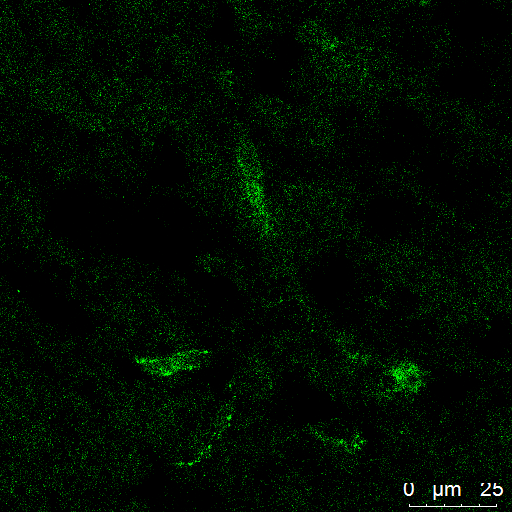

Supplement: Supplementary material — Original Images for Fig S1_Fig S9.zip [file IDRD_A_2585599_SM5400.zip › Original Image for Fig S9 DiD@PLTM left (CD31-marker).tif]

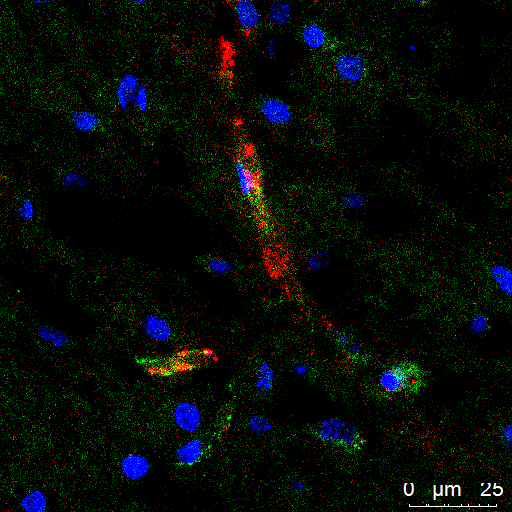

Supplement: Supplementary material — Original Images for Fig S1_Fig S9.zip [file IDRD_A_2585599_SM5400.zip › Original Image for Fig S9 DiD@PLTM left (CD31-merged).tif]

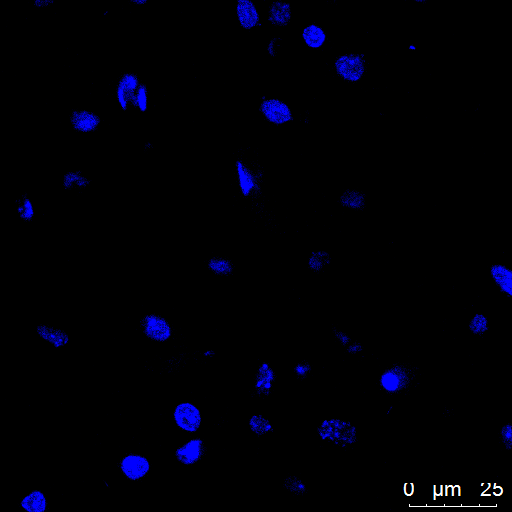

Supplement: Supplementary material — Original Images for Fig S1_Fig S9.zip [file IDRD_A_2585599_SM5400.zip › Original Image for Fig S9 DiD@PLTM left (CD31-nucleus).tif]

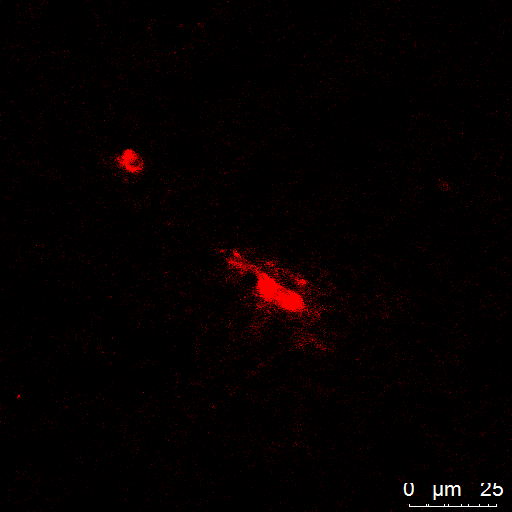

Supplement: Supplementary material — Original Images for Fig S1_Fig S9.zip [file IDRD_A_2585599_SM5400.zip › Original Image for Fig S9 DiD@PLTM left (GFAP-DiD).tif]

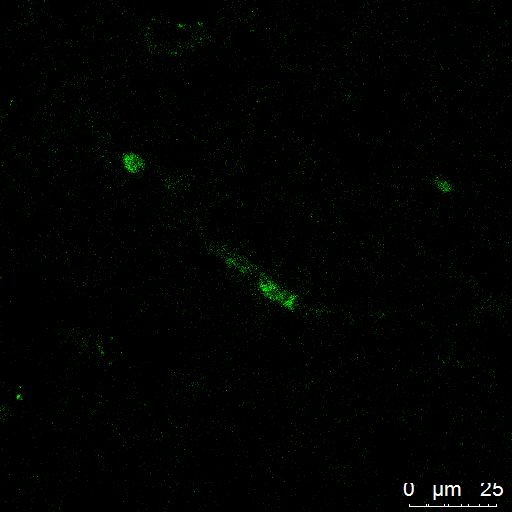

Supplement: Supplementary material — Original Images for Fig S1_Fig S9.zip [file IDRD_A_2585599_SM5400.zip › Original Image for Fig S9 DiD@PLTM left (GFAP-marker).tif]

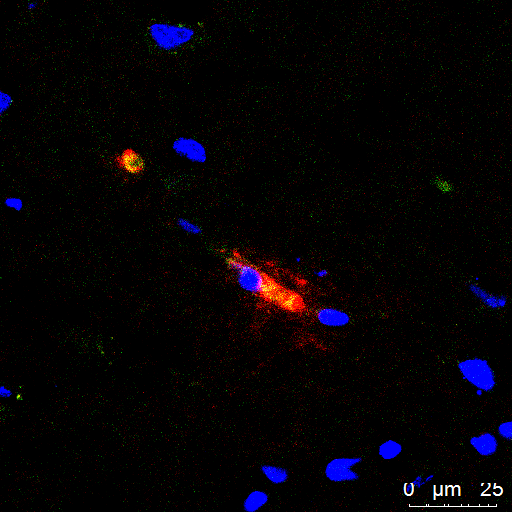

Supplement: Supplementary material — Original Images for Fig S1_Fig S9.zip [file IDRD_A_2585599_SM5400.zip › Original Image for Fig S9 DiD@PLTM left (GFAP-merged).tif]

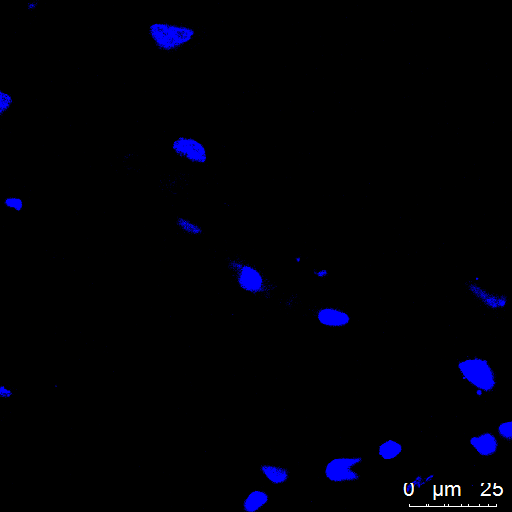

Supplement: Supplementary material — Original Images for Fig S1_Fig S9.zip [file IDRD_A_2585599_SM5400.zip › Original Image for Fig S9 DiD@PLTM left (GFAP-nucleus).tif]

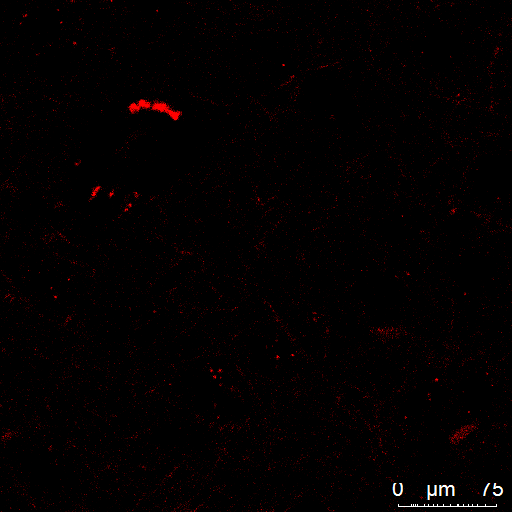

Supplement: Supplementary material — Original Images for Fig S1_Fig S9.zip [file IDRD_A_2585599_SM5400.zip › Original Image for Fig S9 DiD@PLTM left (Iba-1-DiD).tif]

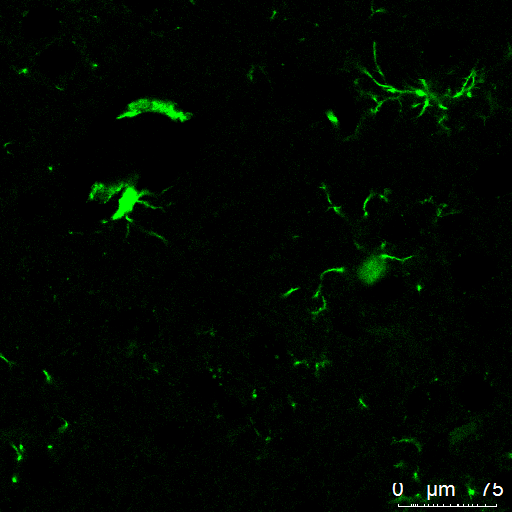

Supplement: Supplementary material — Original Images for Fig S1_Fig S9.zip [file IDRD_A_2585599_SM5400.zip › Original Image for Fig S9 DiD@PLTM left (Iba-1-marker).tif]

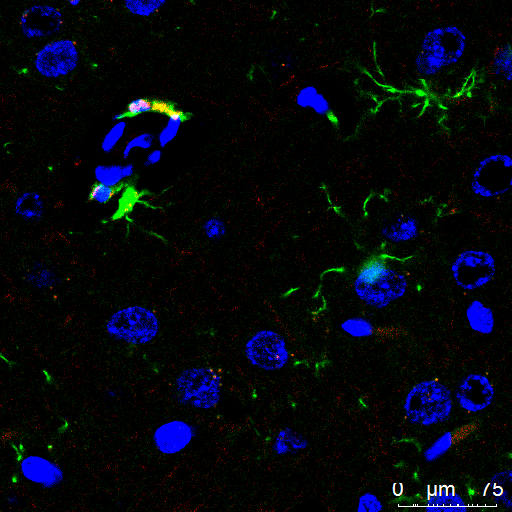

Supplement: Supplementary material — Original Images for Fig S1_Fig S9.zip [file IDRD_A_2585599_SM5400.zip › Original Image for Fig S9 DiD@PLTM left (Iba-1-merged).tif]

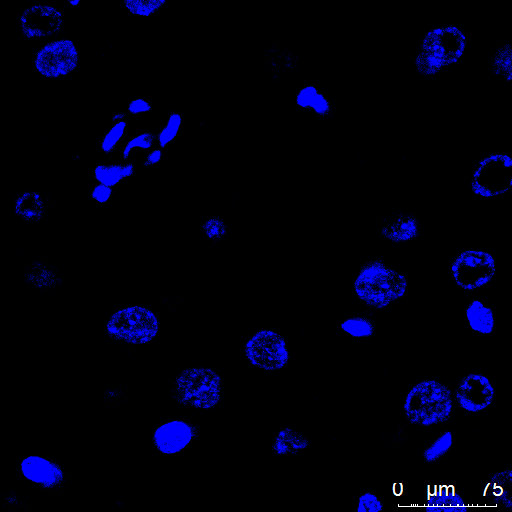

Supplement: Supplementary material — Original Images for Fig S1_Fig S9.zip [file IDRD_A_2585599_SM5400.zip › Original Image for Fig S9 DiD@PLTM left (Iba-1-nucleus).tif]

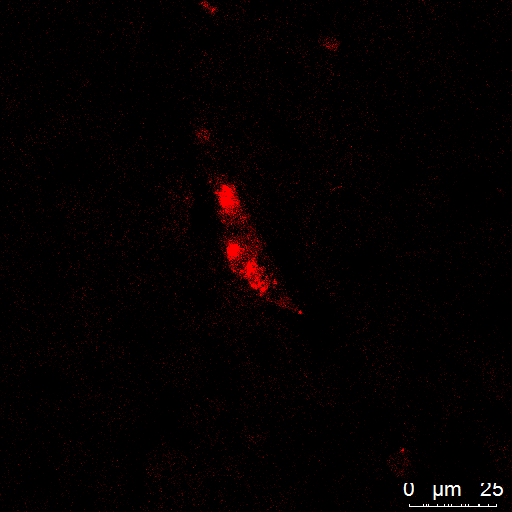

Supplement: Supplementary material — Original Images for Fig S1_Fig S9.zip [file IDRD_A_2585599_SM5400.zip › Original Image for Fig S9 DiD@PLTM left (NeuN-DiD).tif]

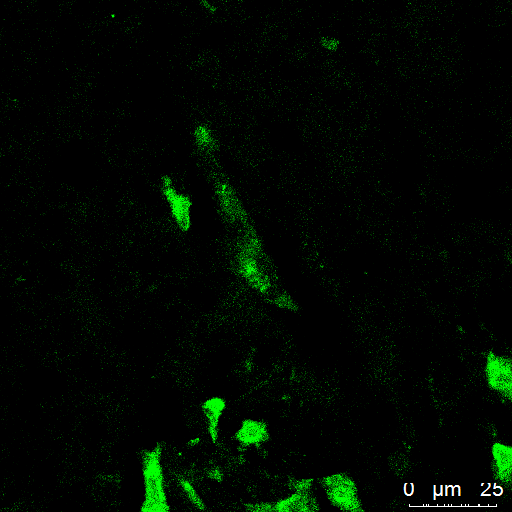

Supplement: Supplementary material — Original Images for Fig S1_Fig S9.zip [file IDRD_A_2585599_SM5400.zip › Original Image for Fig S9 DiD@PLTM left (NeuN-marker).tif]

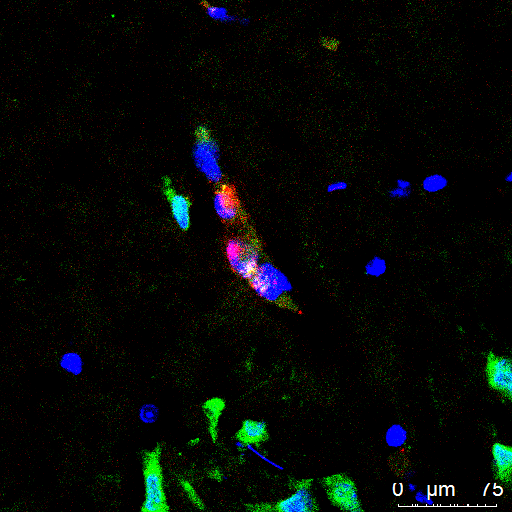

Supplement: Supplementary material — Original Images for Fig S1_Fig S9.zip [file IDRD_A_2585599_SM5400.zip › Original Image for Fig S9 DiD@PLTM left (NeuN-merged).tif]

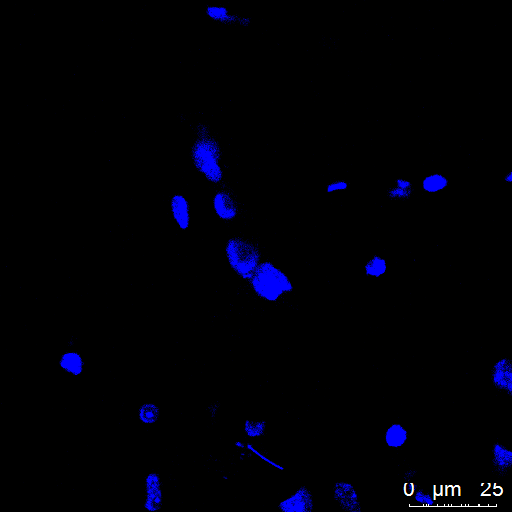

Supplement: Supplementary material — Original Images for Fig S1_Fig S9.zip [file IDRD_A_2585599_SM5400.zip › Original Image for Fig S9 DiD@PLTM left (NeuN-nucleus).tif]

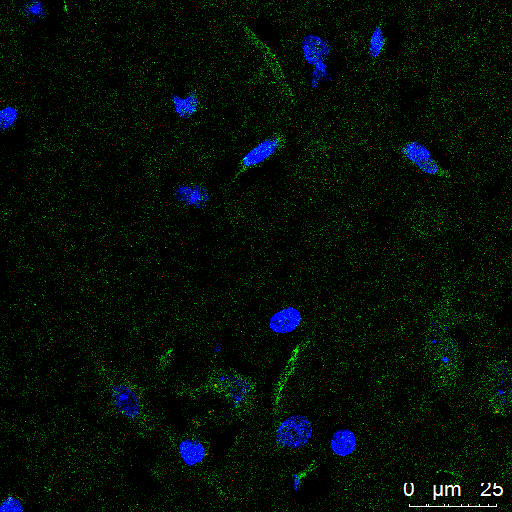

Supplement: Supplementary material — Original Images for Fig S1_Fig S9.zip [file IDRD_A_2585599_SM5400.zip › Original Image for Fig S9 DiD@PLTM right (CD31).tif]

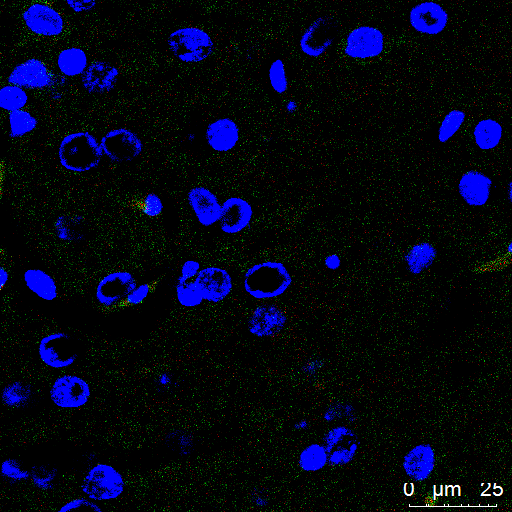

Supplement: Supplementary material — Original Images for Fig S1_Fig S9.zip [file IDRD_A_2585599_SM5400.zip › Original Image for Fig S9 DiD@PLTM right (GFAP).tif]

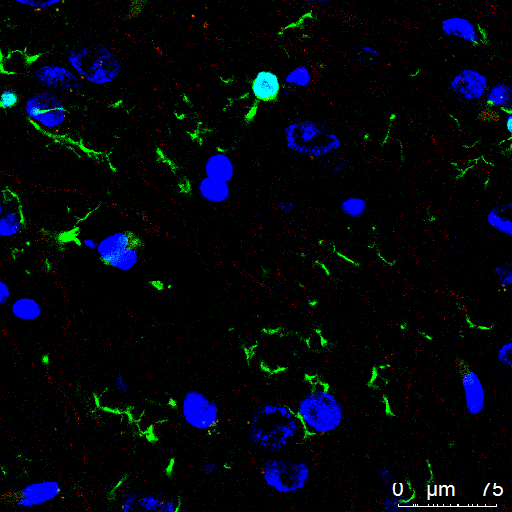

Supplement: Supplementary material — Original Images for Fig S1_Fig S9.zip [file IDRD_A_2585599_SM5400.zip › Original Image for Fig S9 DiD@PLTM right (Iba-1).tif]

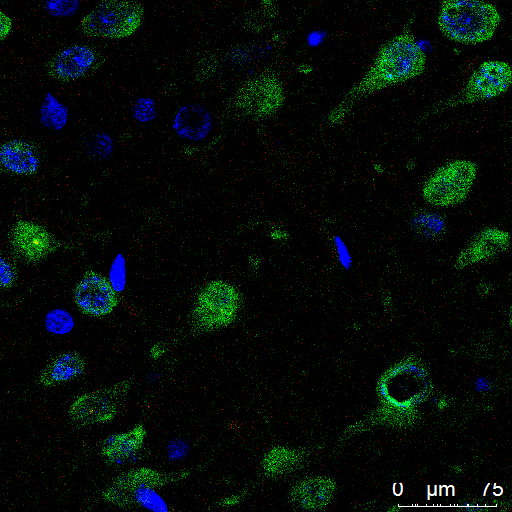

Supplement: Supplementary material — Original Images for Fig S1_Fig S9.zip [file IDRD_A_2585599_SM5400.zip › Original Image for Fig S9 DiD@PLTM right (NeuN).tif]

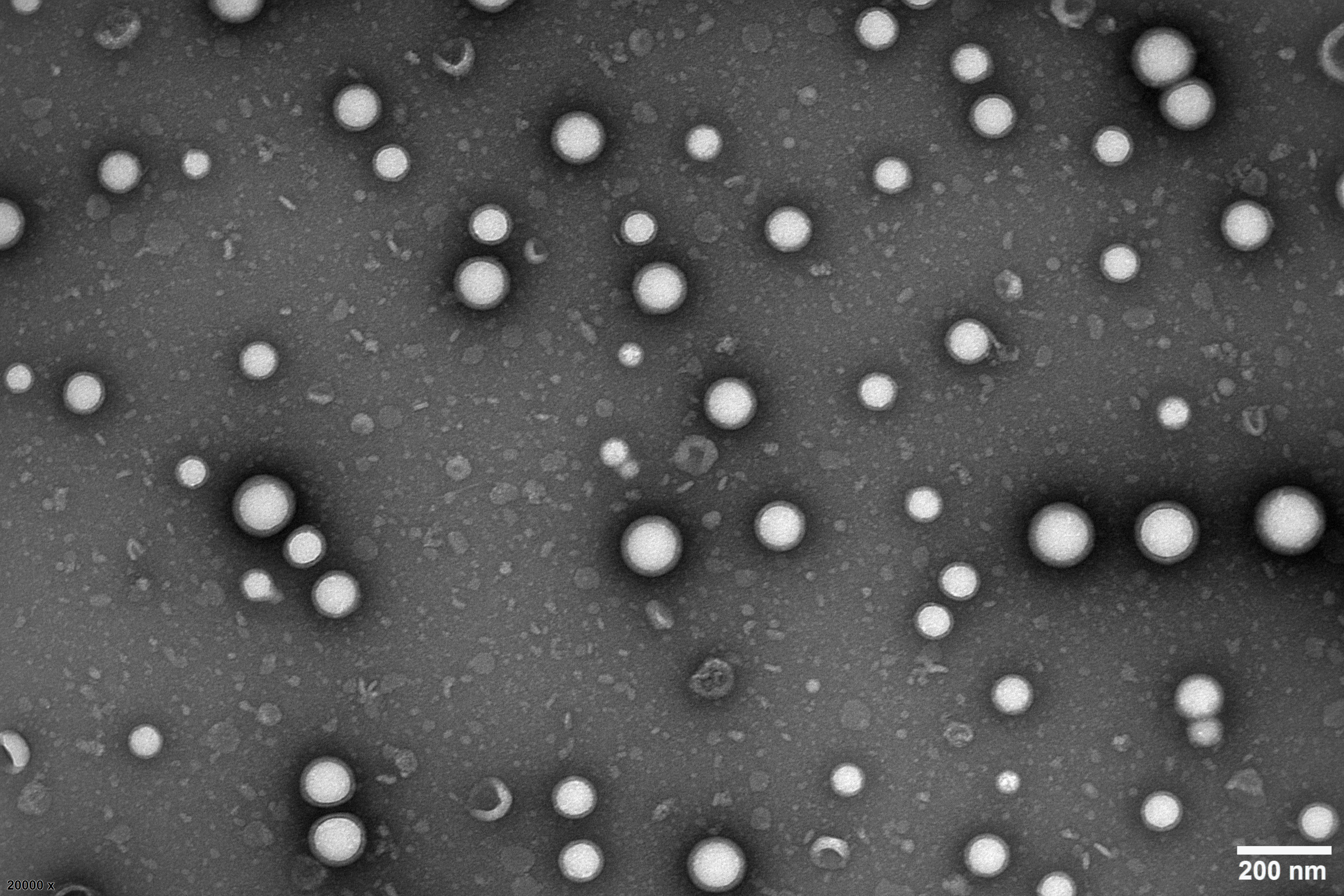

Supplement: Supplementary material — Original Images for Fig 1_Fig 2.zip [file IDRD_A_2585599_SM5401.zip › Original Image for Fig 1A (left).tif]

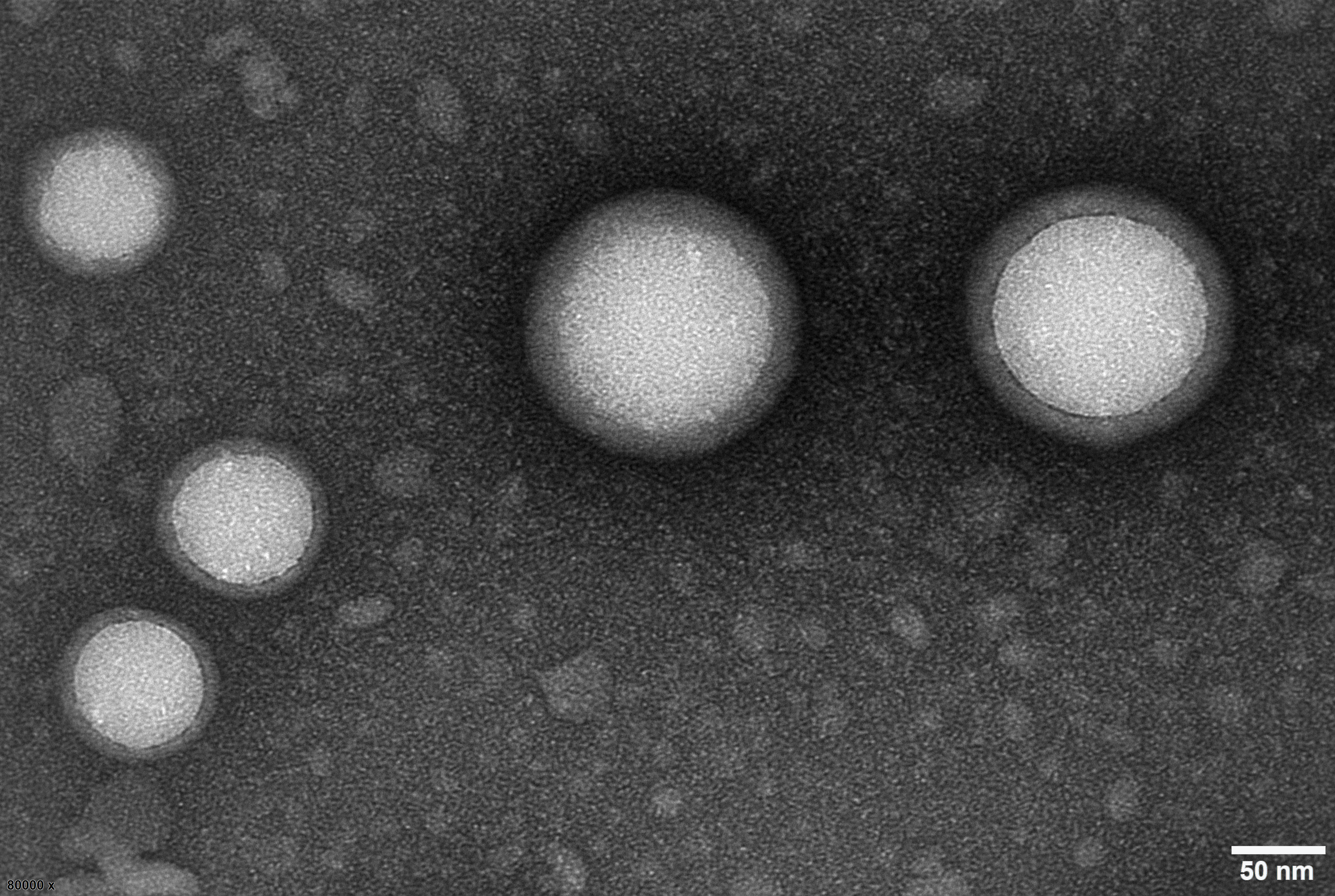

Supplement: Supplementary material — Original Images for Fig 1_Fig 2.zip [file IDRD_A_2585599_SM5401.zip › Original Image for Fig 1A (right).tif]

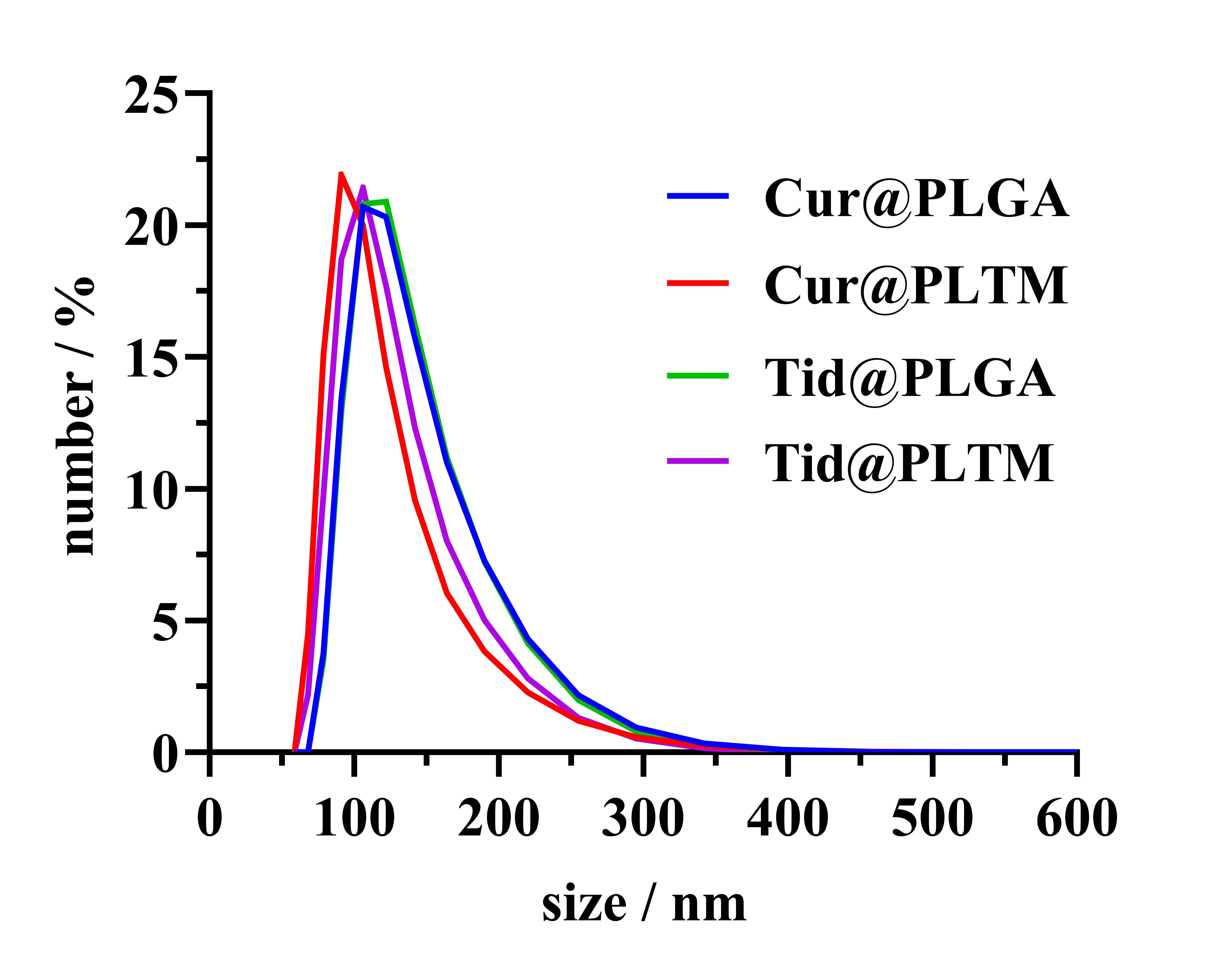

Supplement: Supplementary material — Original Images for Fig 1_Fig 2.zip [file IDRD_A_2585599_SM5401.zip › Original Image for Fig 1B.tif]

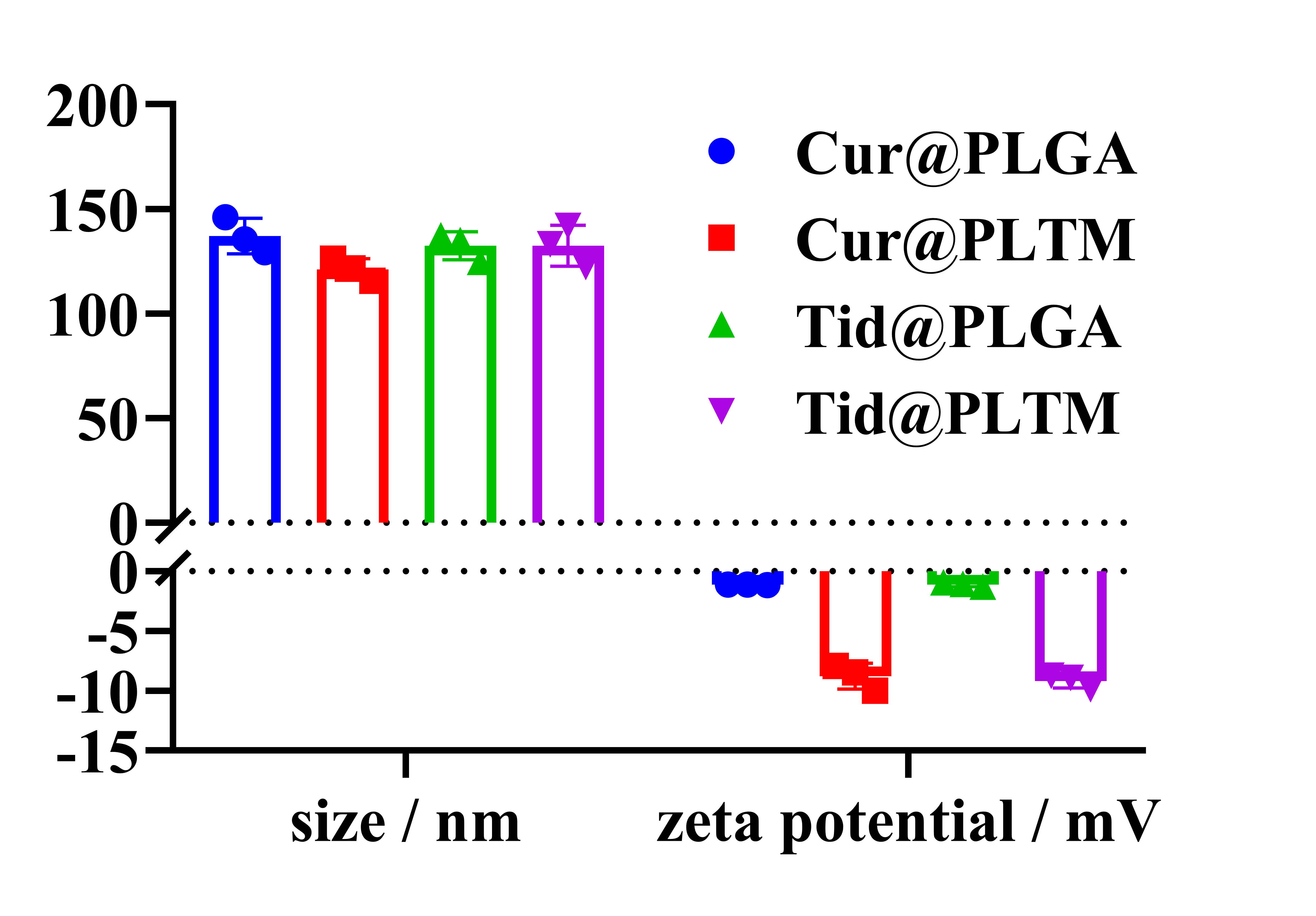

Supplement: Supplementary material — Original Images for Fig 1_Fig 2.zip [file IDRD_A_2585599_SM5401.zip › Original Image for Fig 1C.tif]

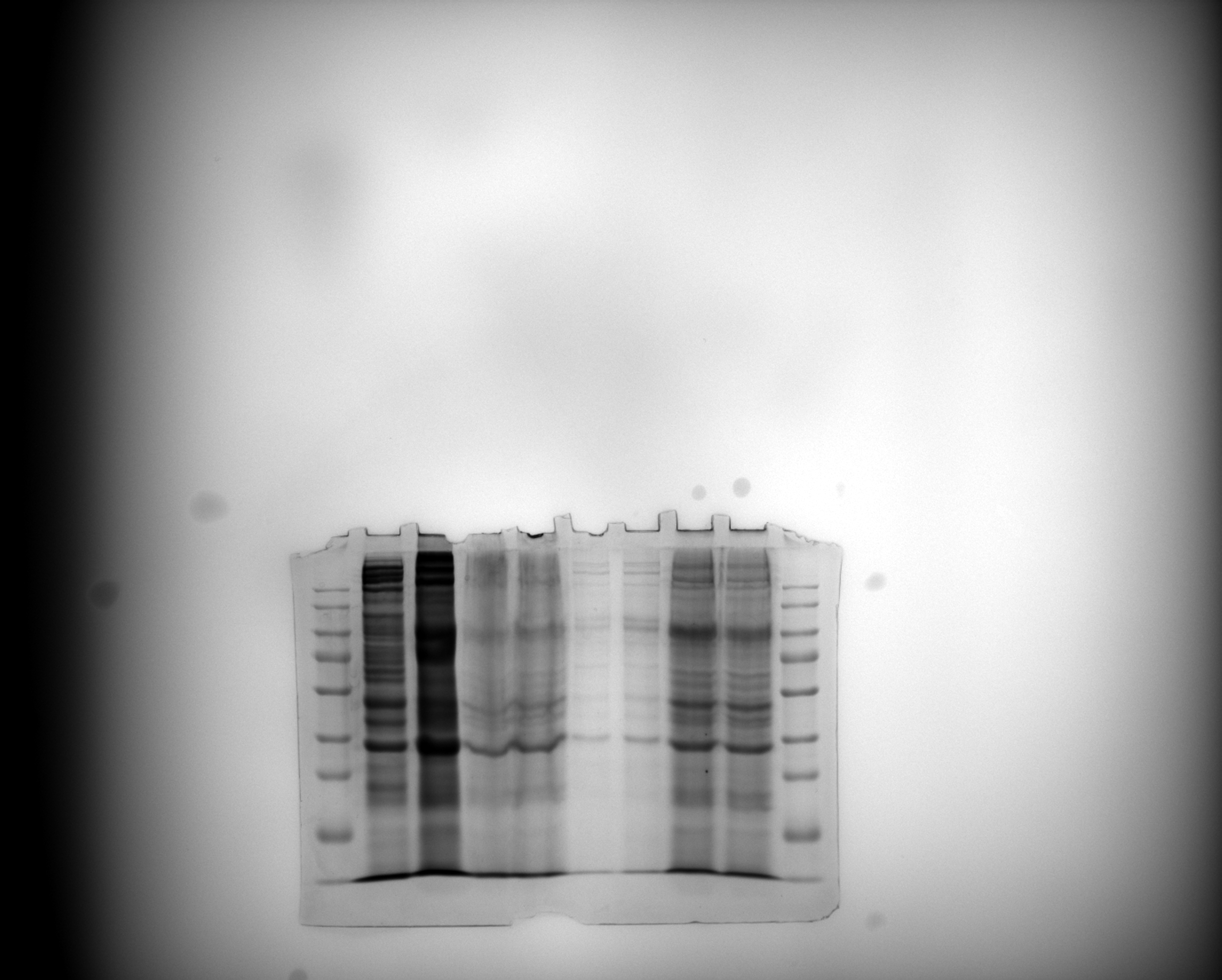

Supplement: Supplementary material — Original Images for Fig 1_Fig 2.zip [file IDRD_A_2585599_SM5401.zip › Original Image for Fig 1D.tif]

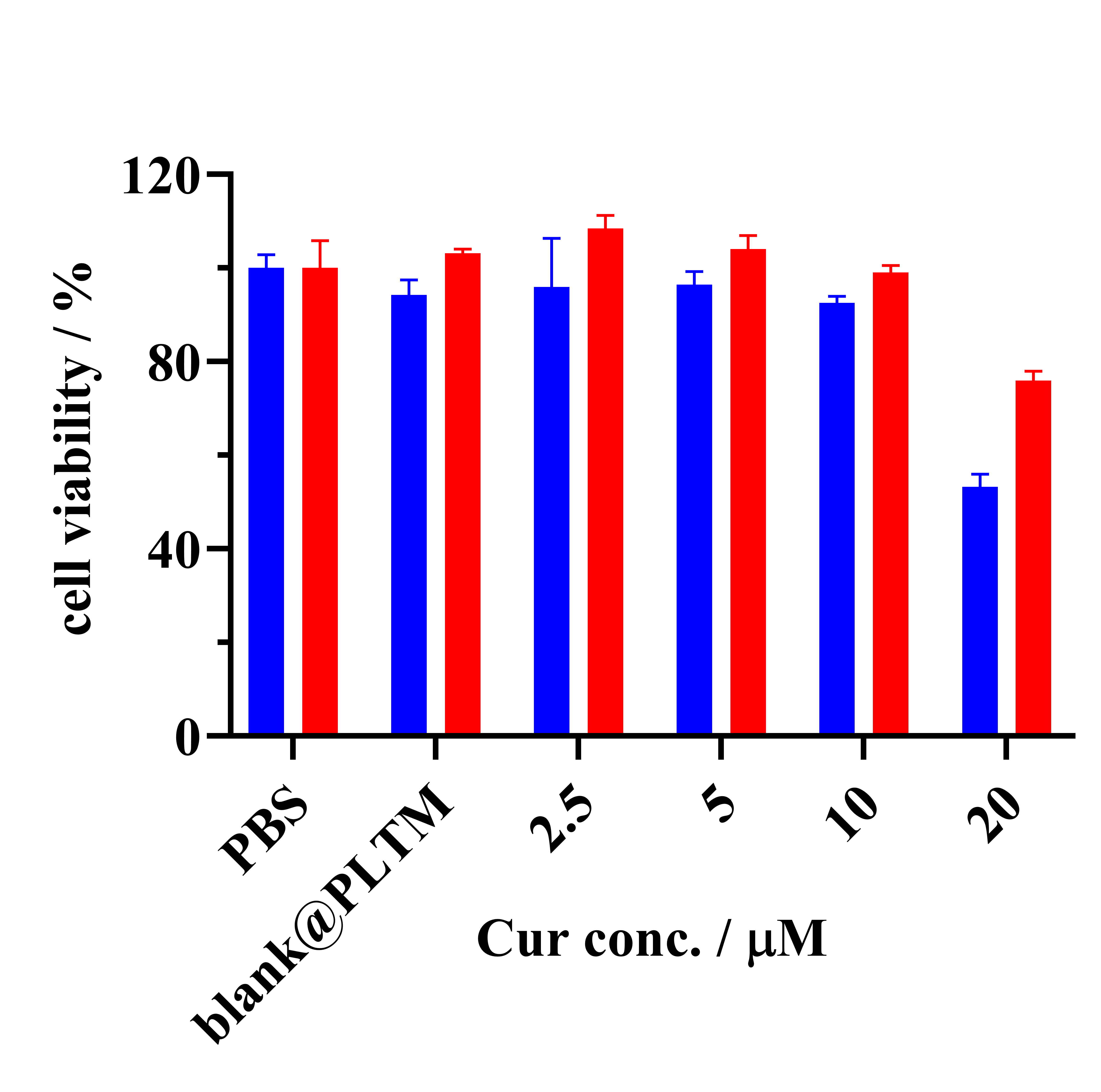

Supplement: Supplementary material — Original Images for Fig 1_Fig 2.zip [file IDRD_A_2585599_SM5401.zip › Original Image for Fig 2A (left).tif]

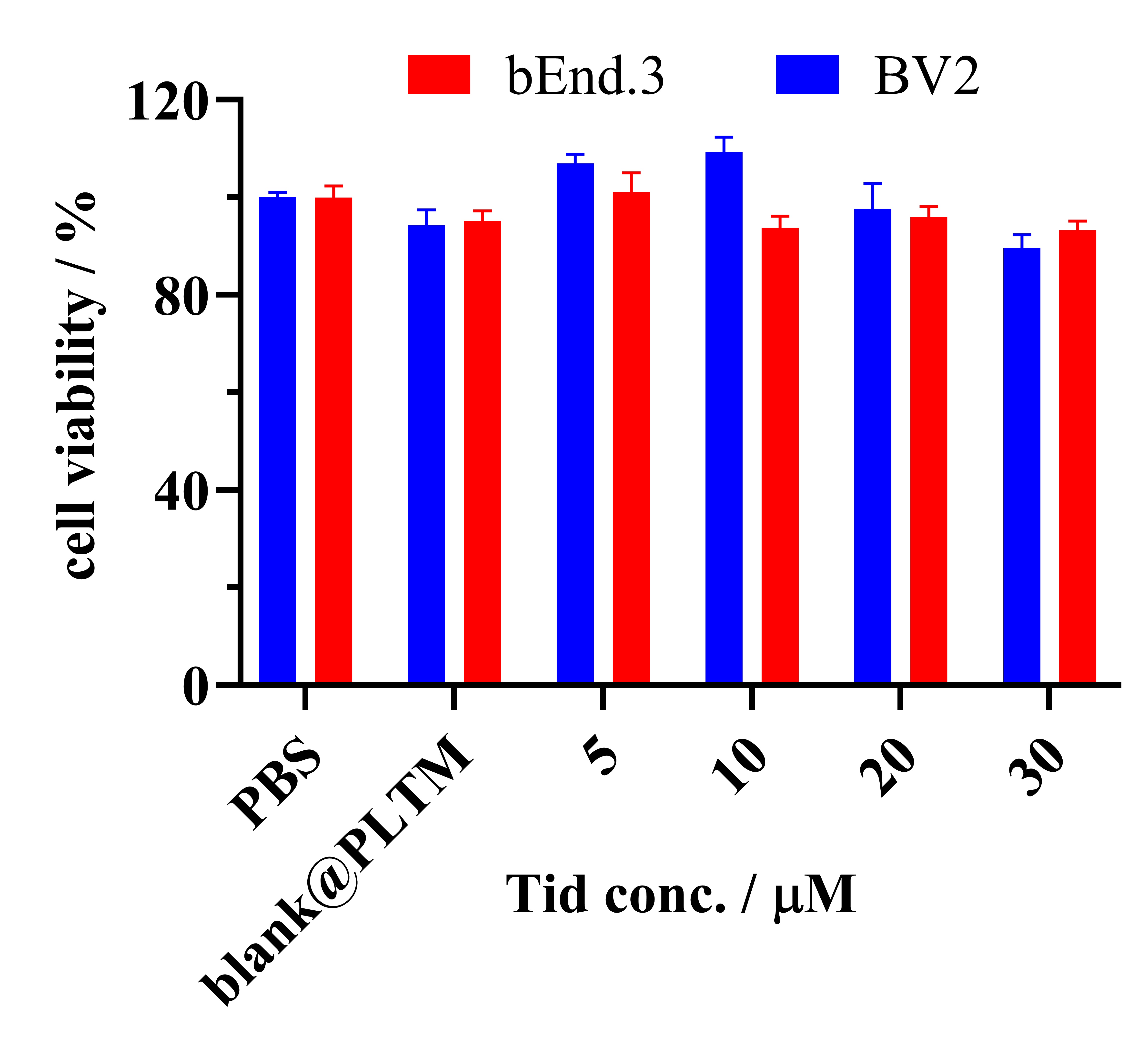

Supplement: Supplementary material — Original Images for Fig 1_Fig 2.zip [file IDRD_A_2585599_SM5401.zip › Original Image for Fig 2A (right).tif]

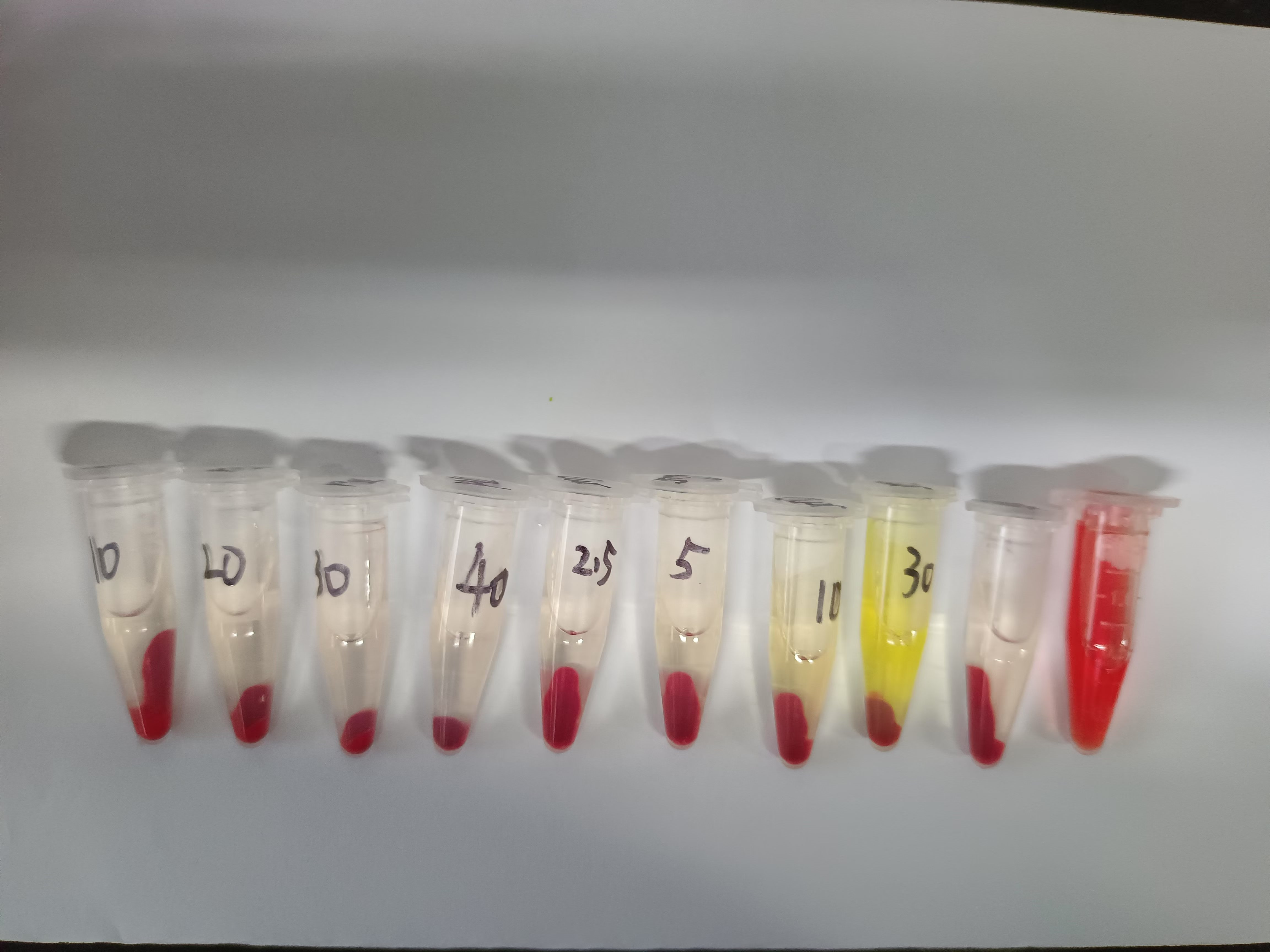

Supplement: Supplementary material — Original Images for Fig 1_Fig 2.zip [file IDRD_A_2585599_SM5401.zip › Original Image for Fig 2B.tif]

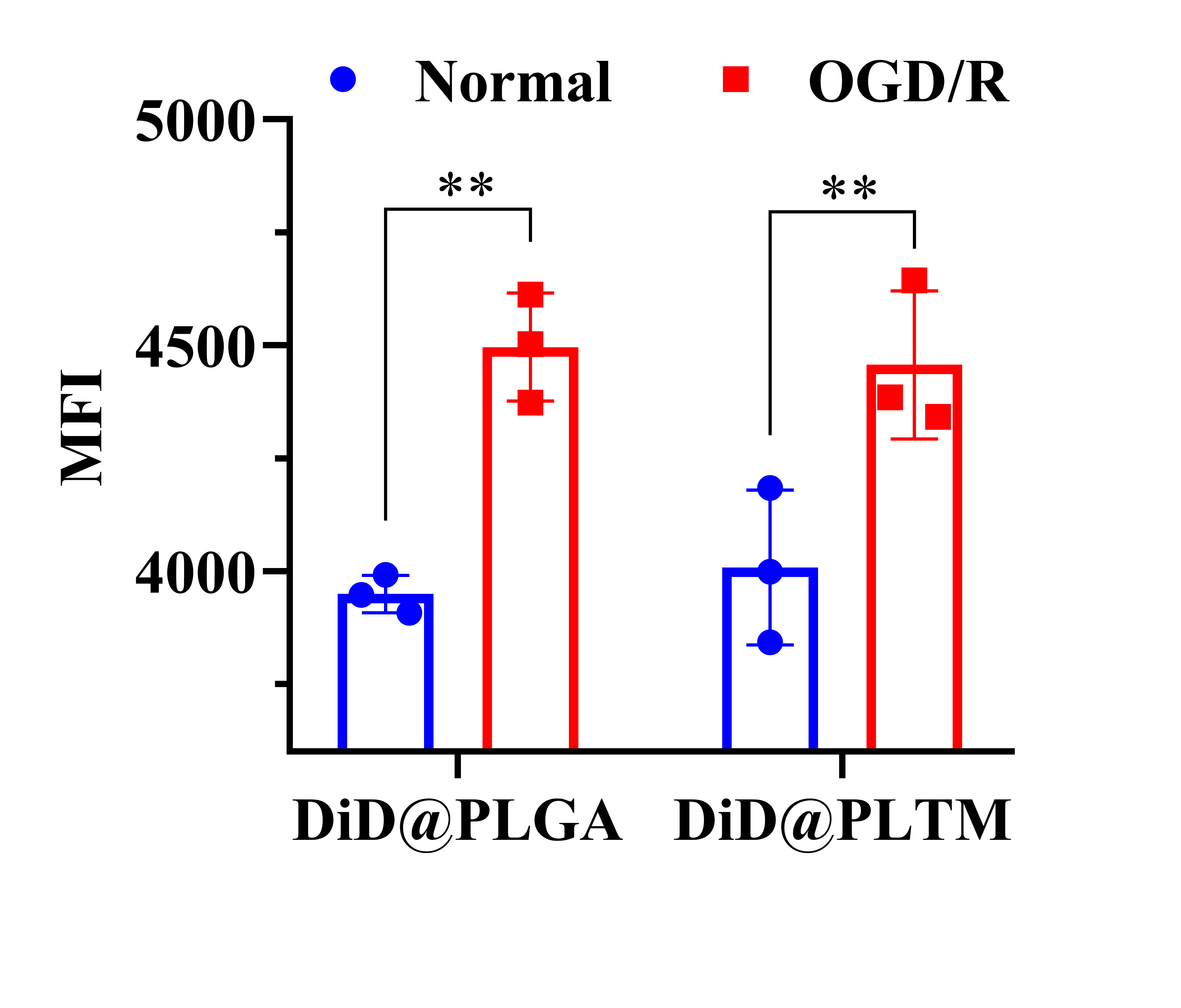

Supplement: Supplementary material — Original Images for Fig 1_Fig 2.zip [file IDRD_A_2585599_SM5401.zip › Original Image for Fig 2C.tif]

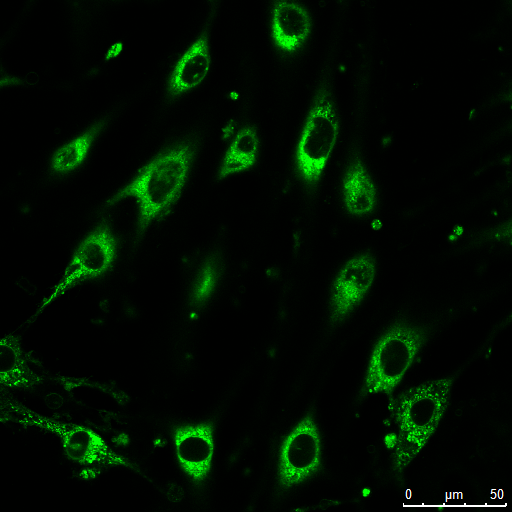

Supplement: Supplementary material — Original Images for Fig 1_Fig 2.zip [file IDRD_A_2585599_SM5401.zip › Original Image for Fig 2D bEnd.3 DiD@PLGA (cytoplasm).tif]

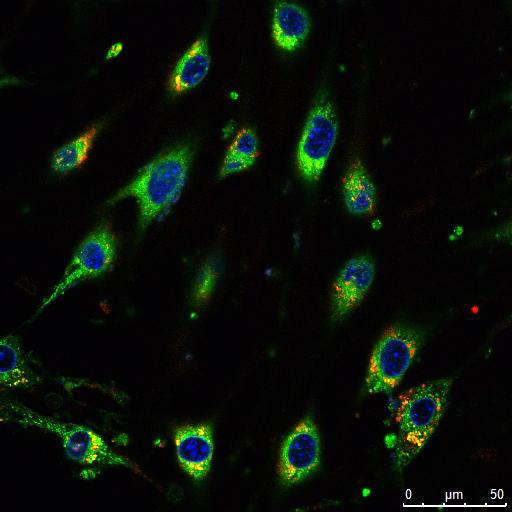

Supplement: Supplementary material — Original Images for Fig 1_Fig 2.zip [file IDRD_A_2585599_SM5401.zip › Original Image for Fig 2D bEnd.3 DiD@PLGA (merged).tif]

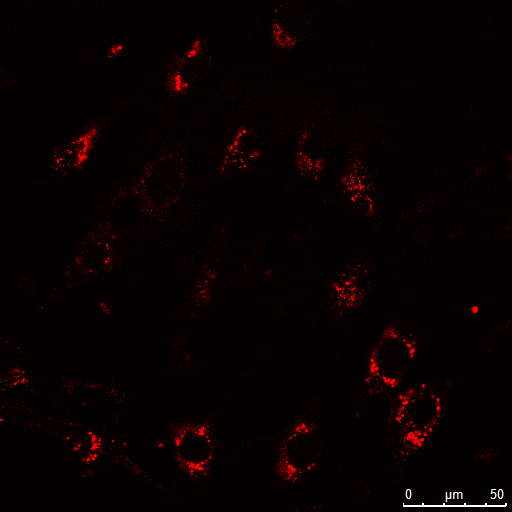

Supplement: Supplementary material — Original Images for Fig 1_Fig 2.zip [file IDRD_A_2585599_SM5401.zip › Original Image for Fig 2D bEnd.3 DiD@PLGA (nanoparticles).tif]

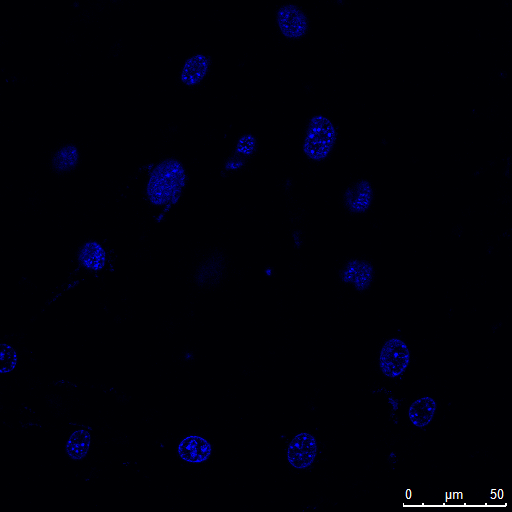

Supplement: Supplementary material — Original Images for Fig 1_Fig 2.zip [file IDRD_A_2585599_SM5401.zip › Original Image for Fig 2D bEnd.3 DiD@PLGA (nucleus).tif]

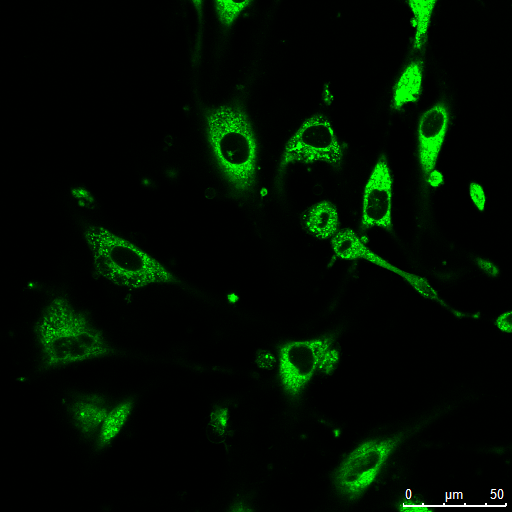

Supplement: Supplementary material — Original Images for Fig 1_Fig 2.zip [file IDRD_A_2585599_SM5401.zip › Original Image for Fig 2D bEnd.3 DiD@PLTM (cytoplasm).tif]

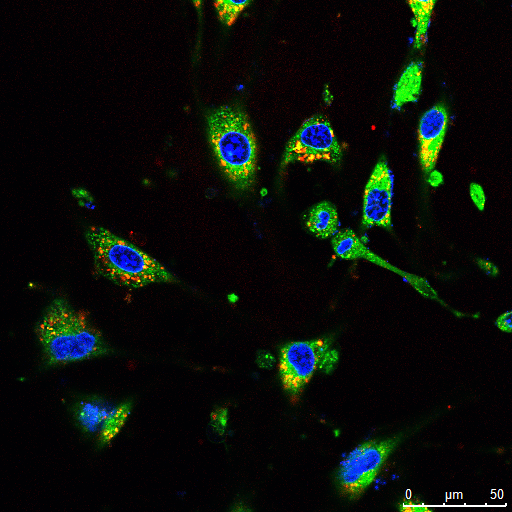

Supplement: Supplementary material — Original Images for Fig 1_Fig 2.zip [file IDRD_A_2585599_SM5401.zip › Original Image for Fig 2D bEnd.3 DiD@PLTM (merged).tif]

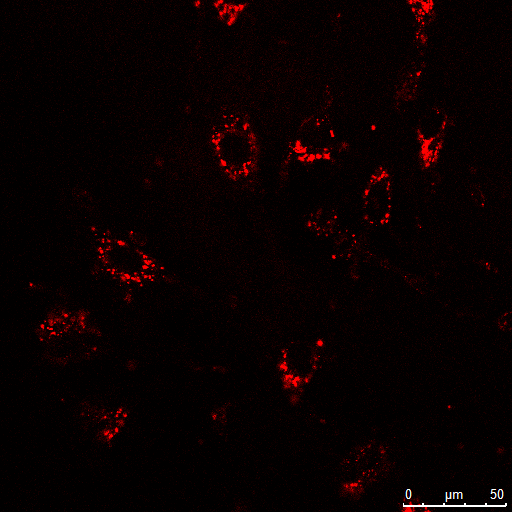

Supplement: Supplementary material — Original Images for Fig 1_Fig 2.zip [file IDRD_A_2585599_SM5401.zip › Original Image for Fig 2D bEnd.3 DiD@PLTM (nanoparticles).tif]

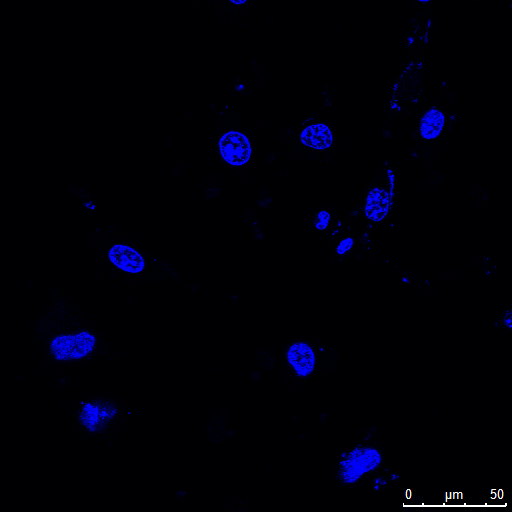

Supplement: Supplementary material — Original Images for Fig 1_Fig 2.zip [file IDRD_A_2585599_SM5401.zip › Original Image for Fig 2D bEnd.3 DiD@PLTM (nucleus).tif]

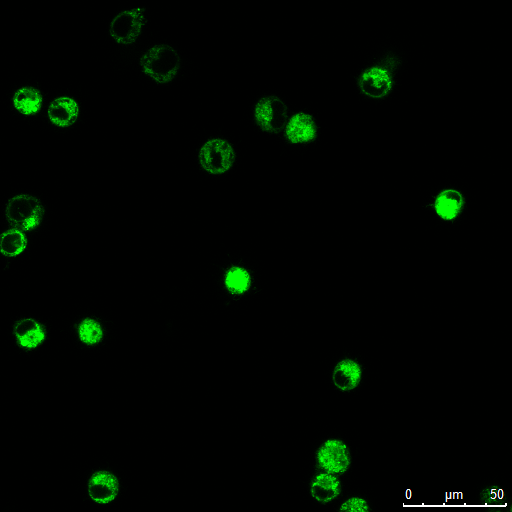

Supplement: Supplementary material — Original Images for Fig 1_Fig 2.zip [file IDRD_A_2585599_SM5401.zip › Original Image for Fig 2D BV2 DiD@PLGA (cytoplasm).tif]

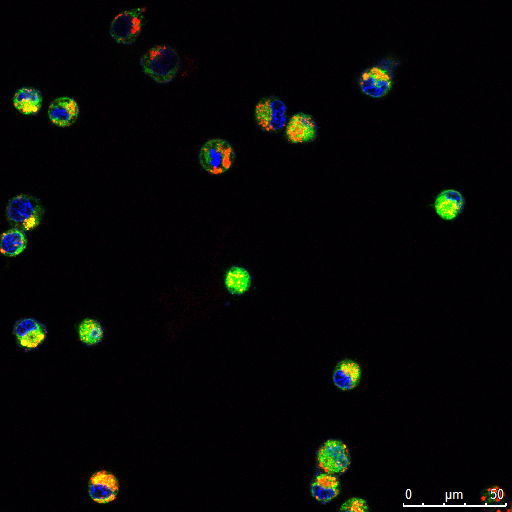

Supplement: Supplementary material — Original Images for Fig 1_Fig 2.zip [file IDRD_A_2585599_SM5401.zip › Original Image for Fig 2D BV2 DiD@PLGA (merged).tif]

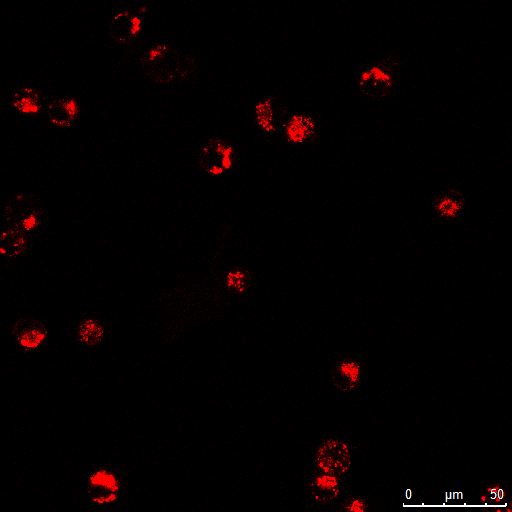

Supplement: Supplementary material — Original Images for Fig 1_Fig 2.zip [file IDRD_A_2585599_SM5401.zip › Original Image for Fig 2D BV2 DiD@PLGA (nanoparticles).tif]

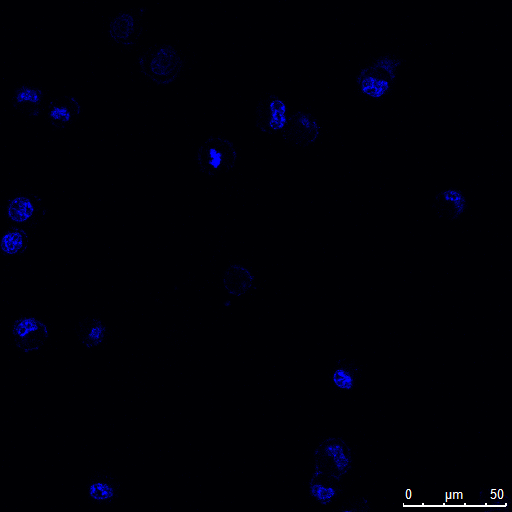

Supplement: Supplementary material — Original Images for Fig 1_Fig 2.zip [file IDRD_A_2585599_SM5401.zip › Original Image for Fig 2D BV2 DiD@PLGA (nucleus).tif]

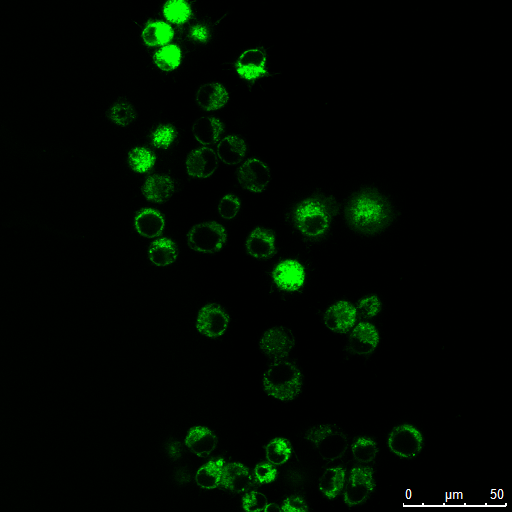

Supplement: Supplementary material — Original Images for Fig 1_Fig 2.zip [file IDRD_A_2585599_SM5401.zip › Original Image for Fig 2D BV2 DiD@PLTM (cytoplasm).tif]

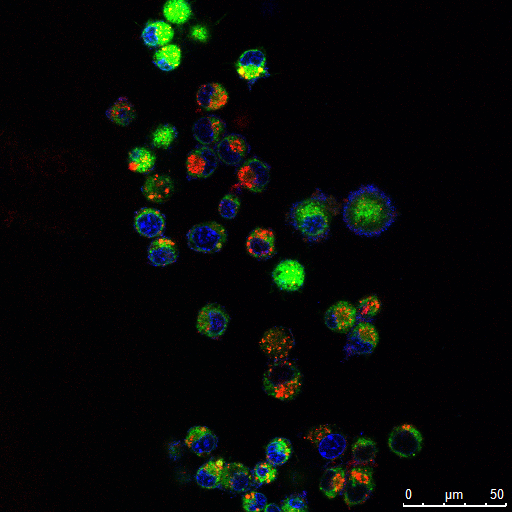

Supplement: Supplementary material — Original Images for Fig 1_Fig 2.zip [file IDRD_A_2585599_SM5401.zip › Original Image for Fig 2D BV2 DiD@PLTM (merged).tif]

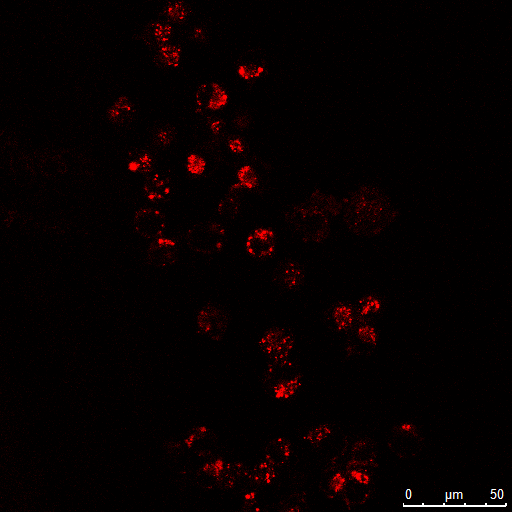

Supplement: Supplementary material — Original Images for Fig 1_Fig 2.zip [file IDRD_A_2585599_SM5401.zip › Original Image for Fig 2D BV2 DiD@PLTM (nanoparticles).tif]

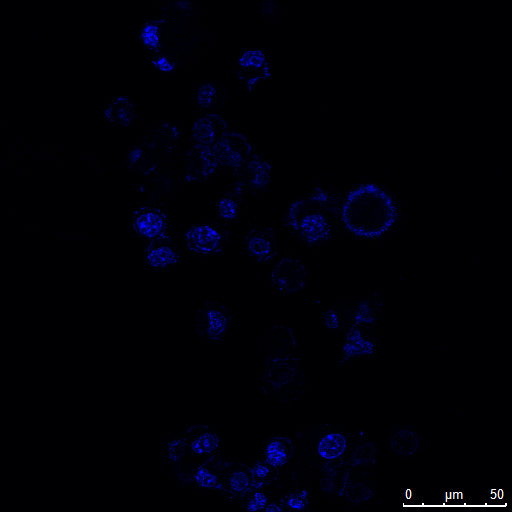

Supplement: Supplementary material — Original Images for Fig 1_Fig 2.zip [file IDRD_A_2585599_SM5401.zip › Original Image for Fig 2D BV2 DiD@PLTM (nucleus).tif]

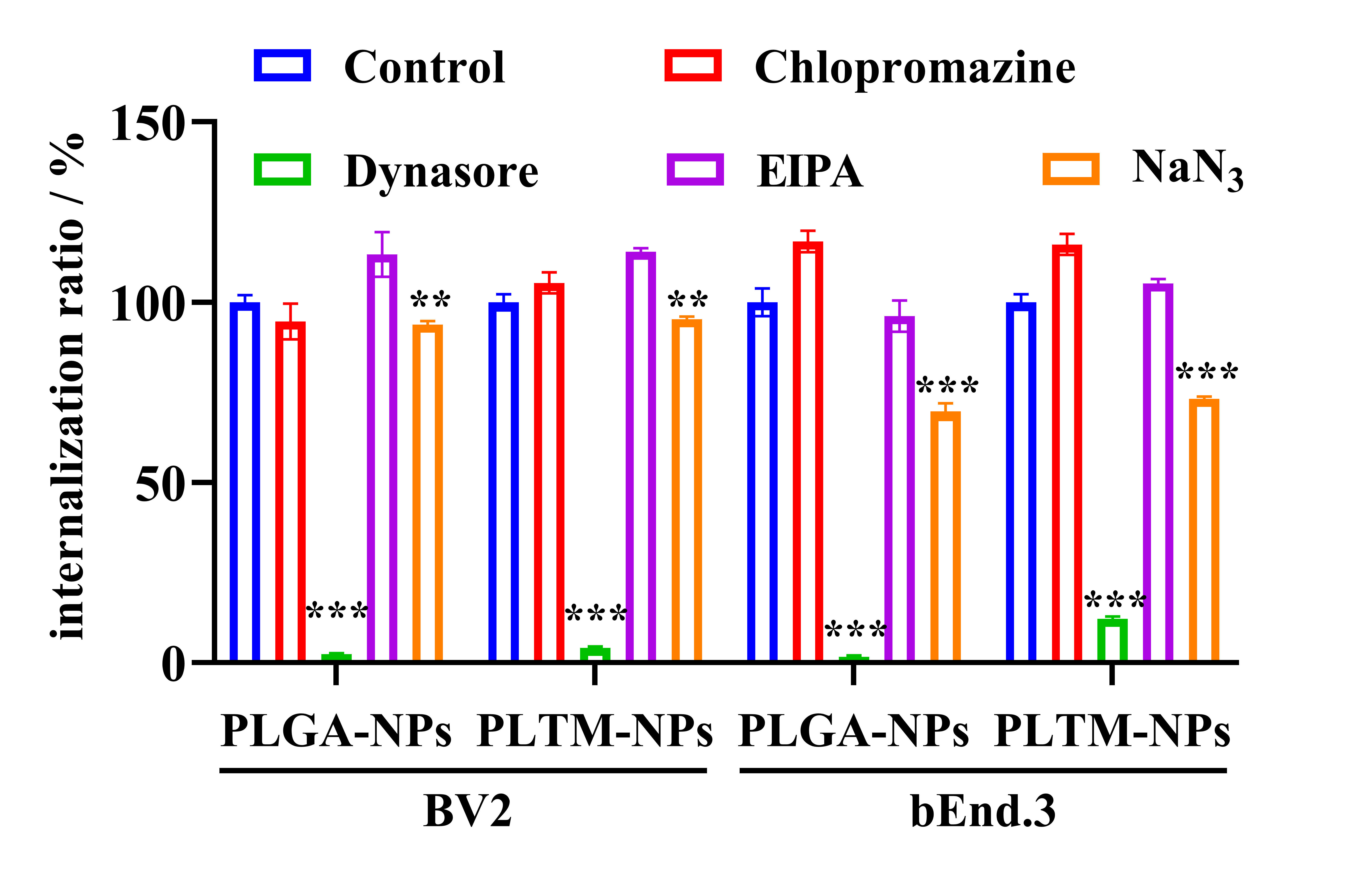

Supplement: Supplementary material — Original Images for Fig 1_Fig 2.zip [file IDRD_A_2585599_SM5401.zip › Original Image for Fig 2E.tif]

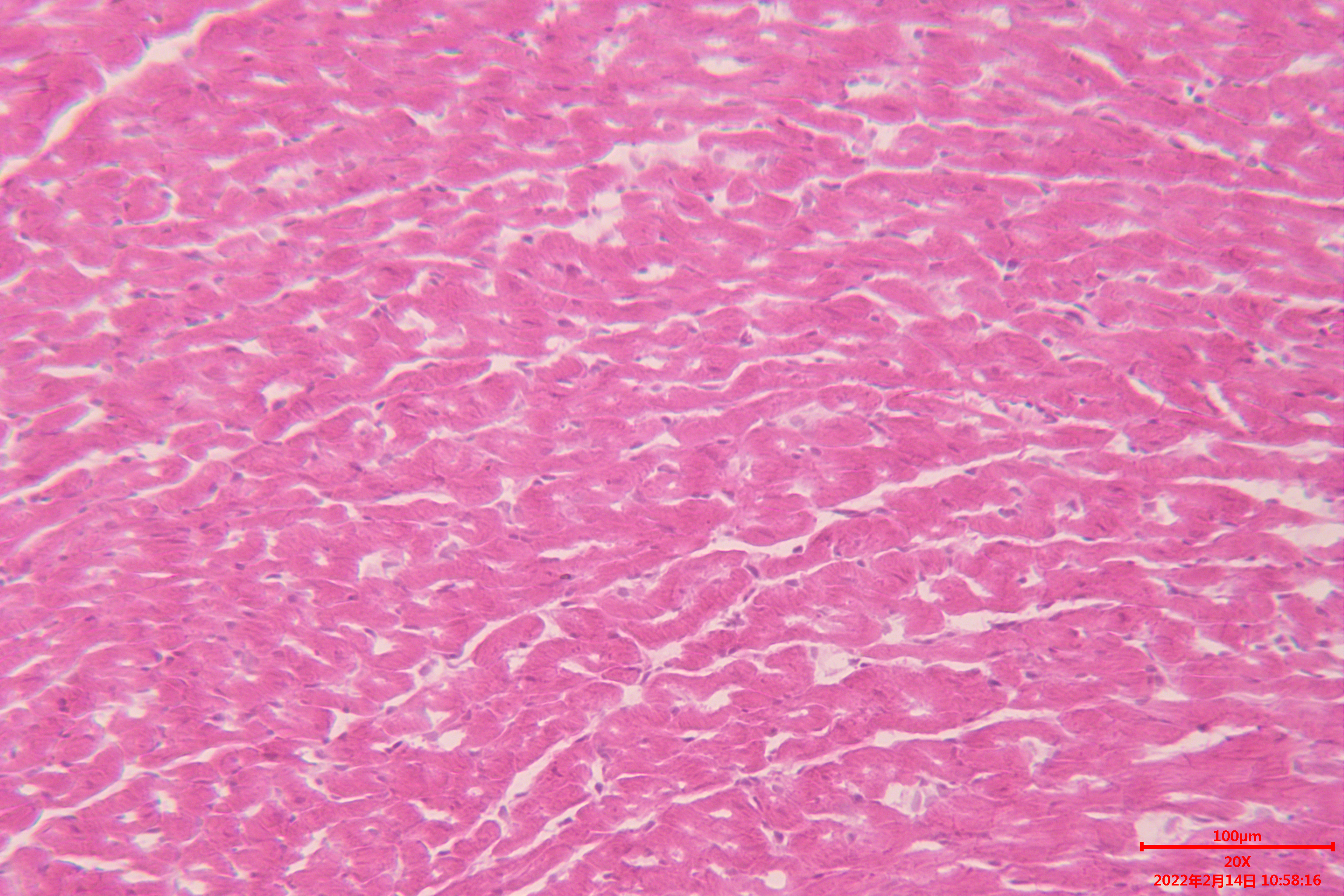

Supplement: Supplementary material — Original Images for Fig S10_2.zip [file IDRD_A_2585599_SM5402.zip › Original Image for Fig S10 G3 (heart).tif]

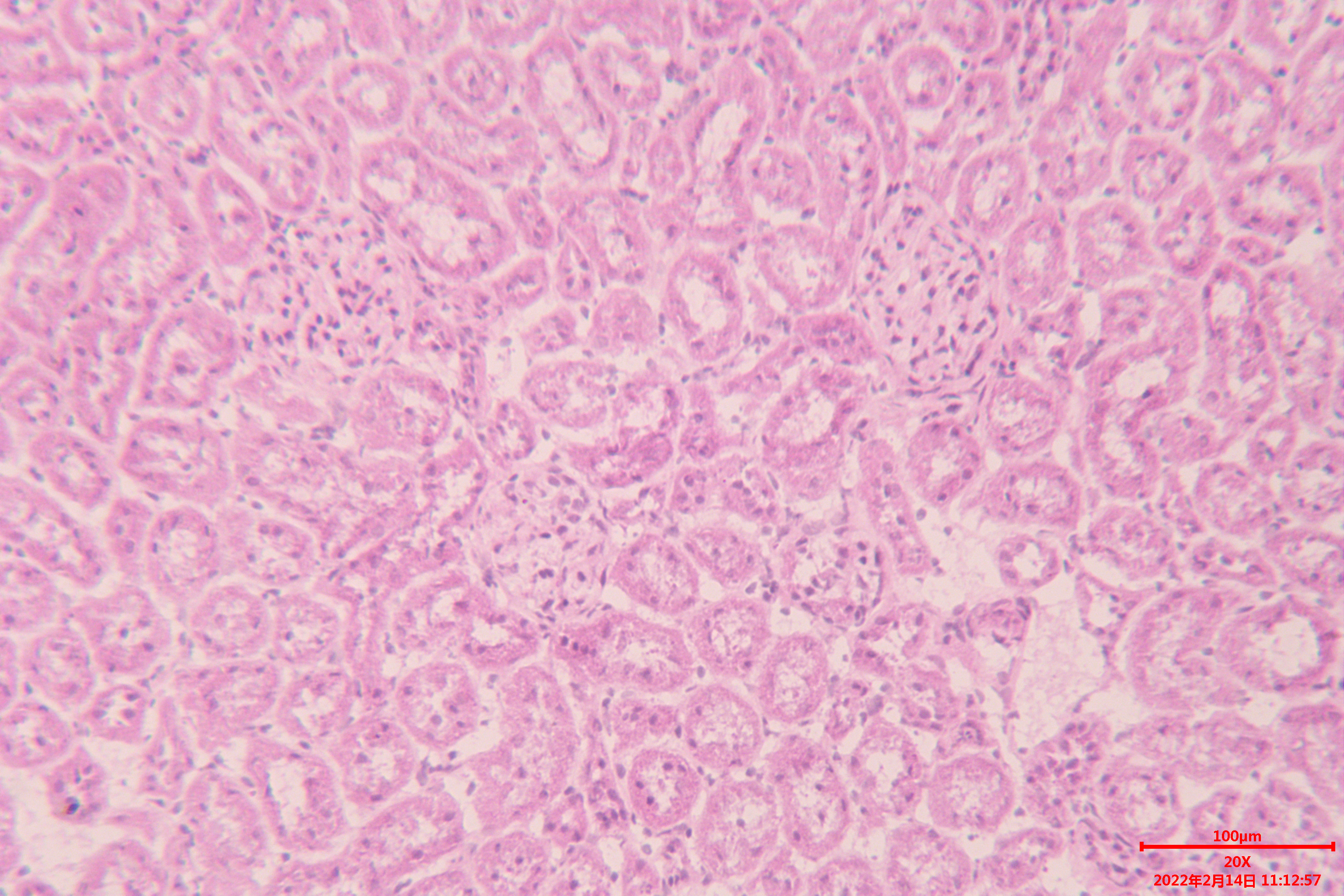

Supplement: Supplementary material — Original Images for Fig S10_2.zip [file IDRD_A_2585599_SM5402.zip › Original Image for Fig S10 G3 (kidney).tif]

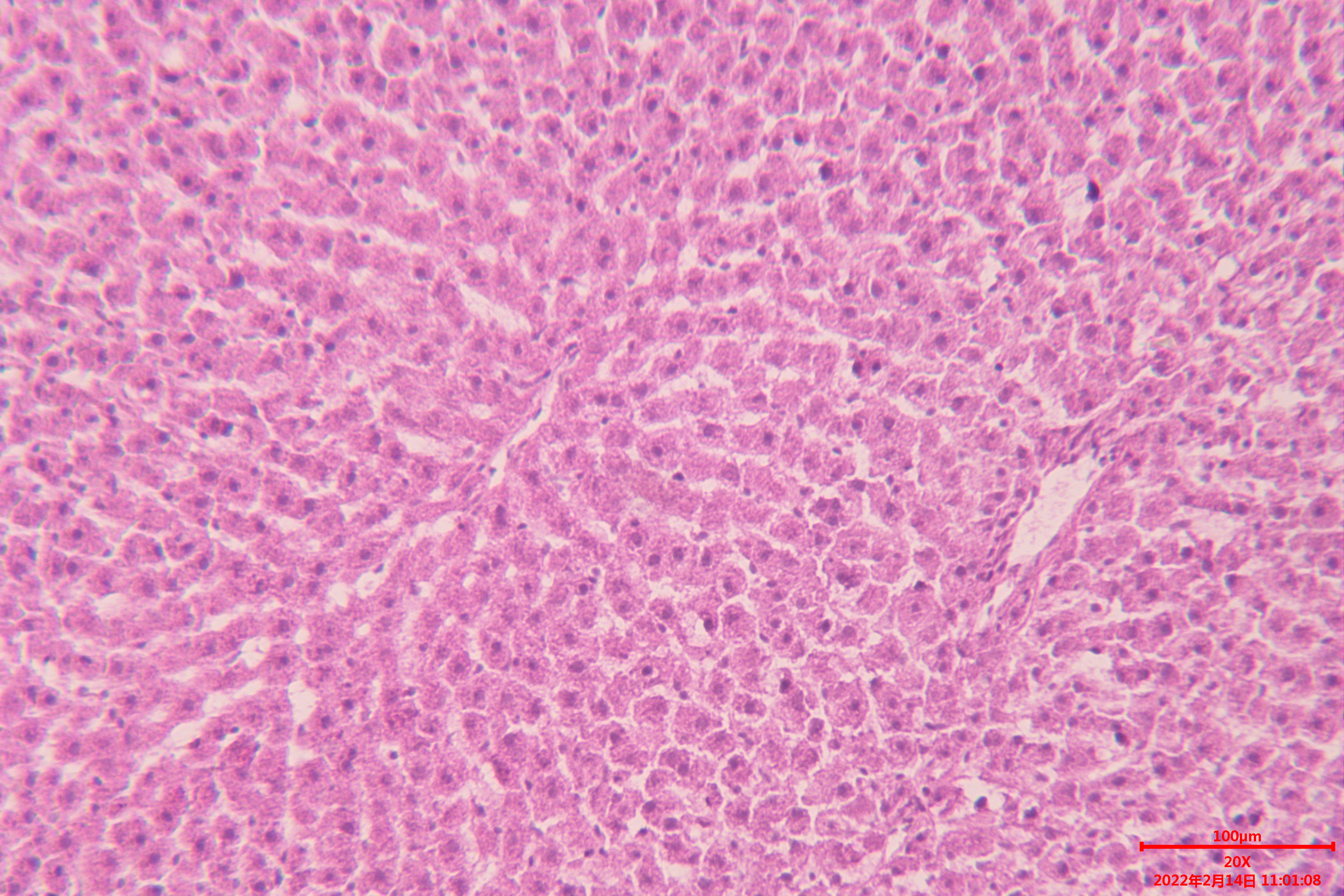

Supplement: Supplementary material — Original Images for Fig S10_2.zip [file IDRD_A_2585599_SM5402.zip › Original Image for Fig S10 G3 (liver).tif]

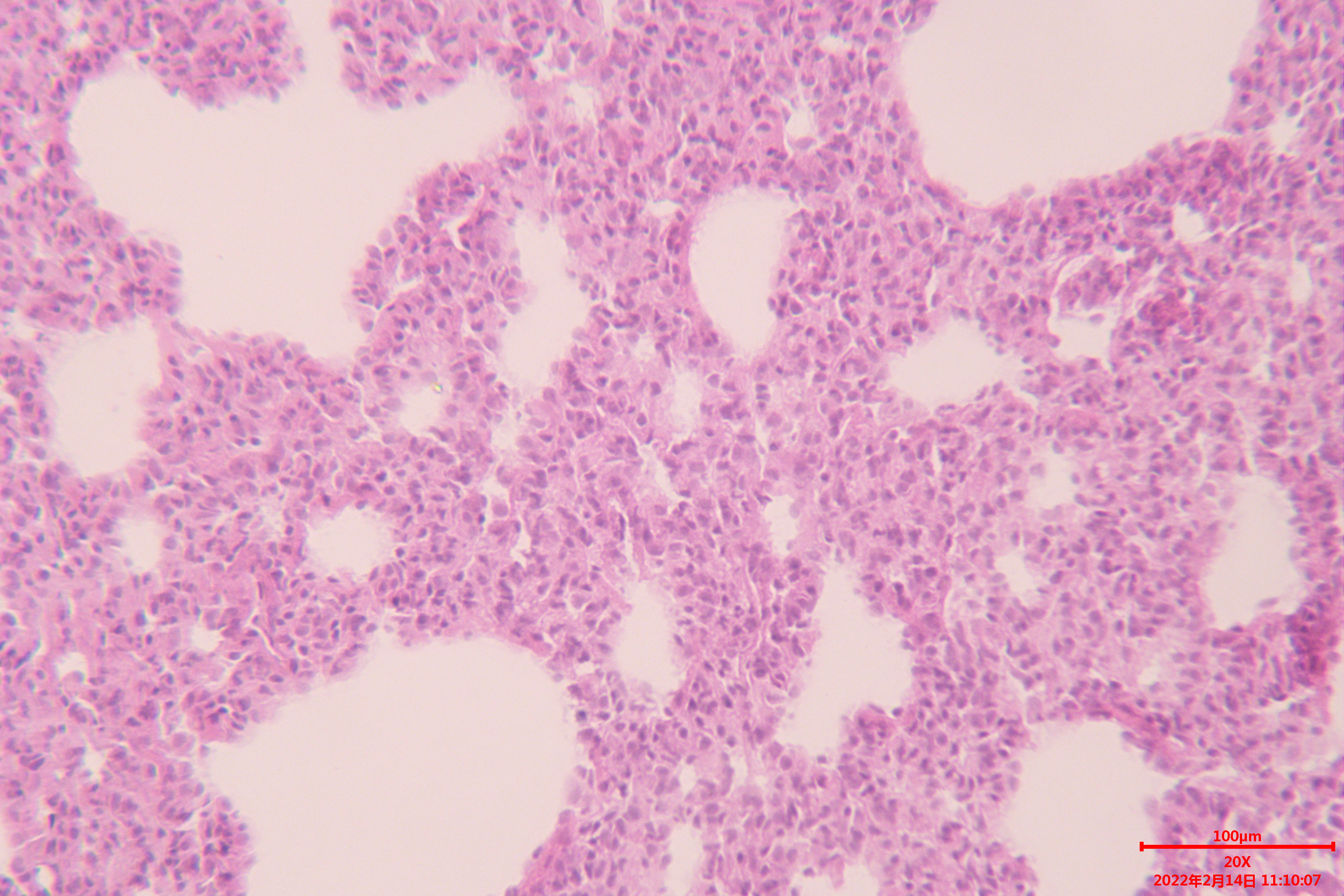

Supplement: Supplementary material — Original Images for Fig S10_2.zip [file IDRD_A_2585599_SM5402.zip › Original Image for Fig S10 G3 (lung).tif]
