# Supplementary figures and images for: Targeted strategy by curcumin and tideglusib biomimetic nano-systems alleviates oxidative stress and inflammation under ischemic stroke (part 2 of 3)
Source: Drug Deliv. 2025 Nov 25;32(1):2585599. doi: 10.1080/10717544.2025.2585599 (PMC12667299; doi:10.1080/10717544.2025.2585599)

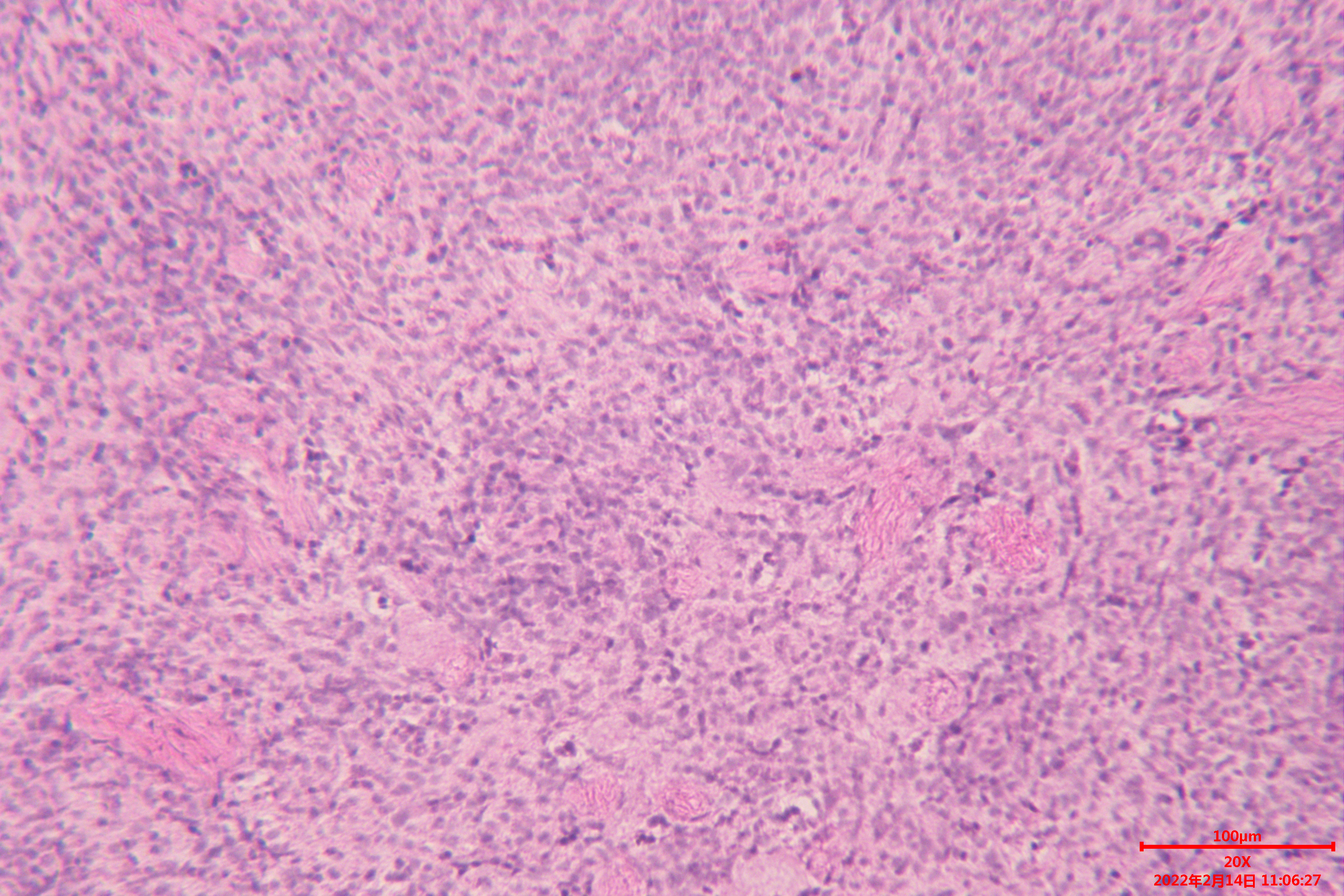

Supplement: Supplementary material — Original Images for Fig S10_2.zip [file IDRD_A_2585599_SM5402.zip › Original Image for Fig S10 G3 (spleen).tif]

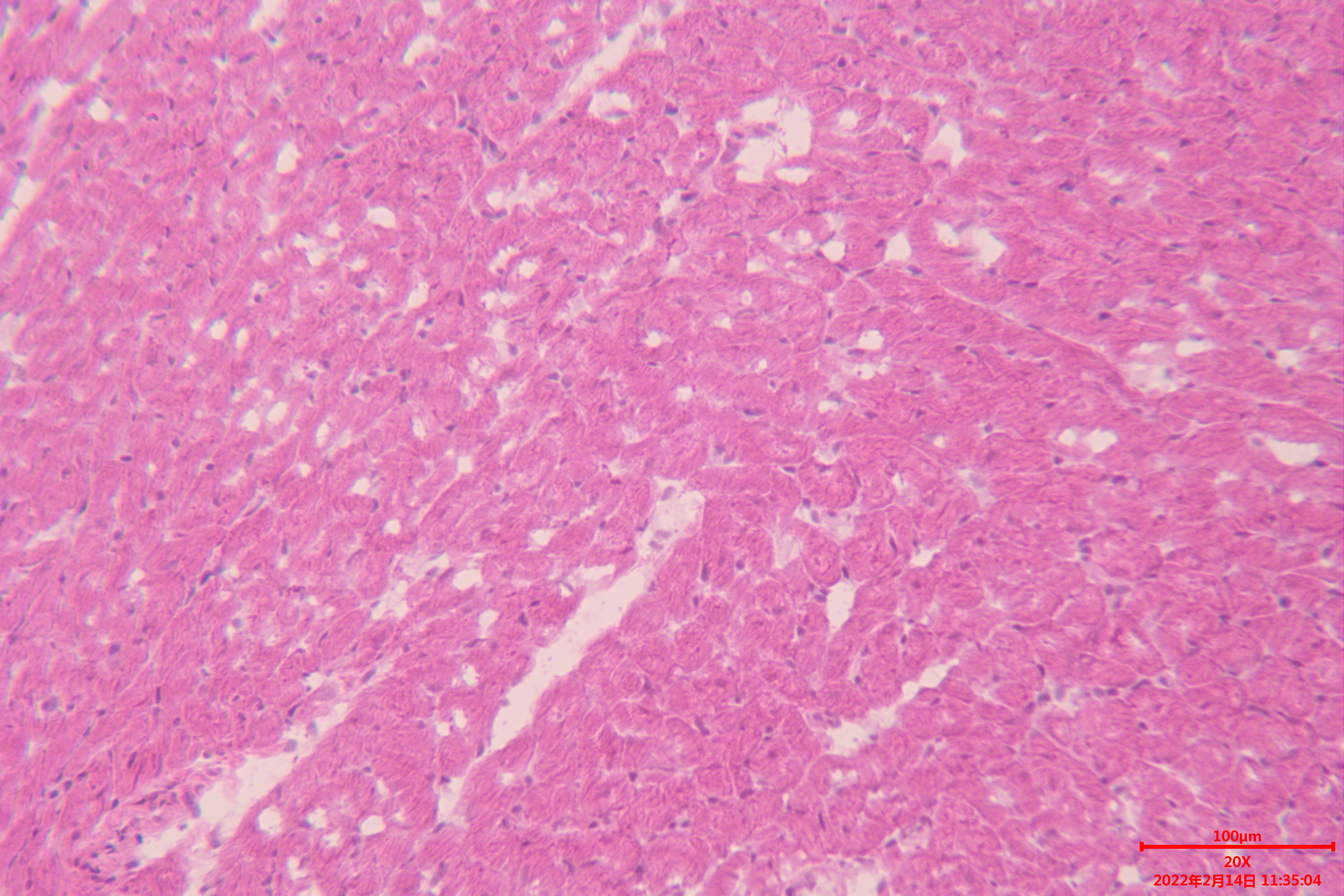

Supplement: Supplementary material — Original Images for Fig S10_2.zip [file IDRD_A_2585599_SM5402.zip › Original Image for Fig S10 G4 (heart).tif]

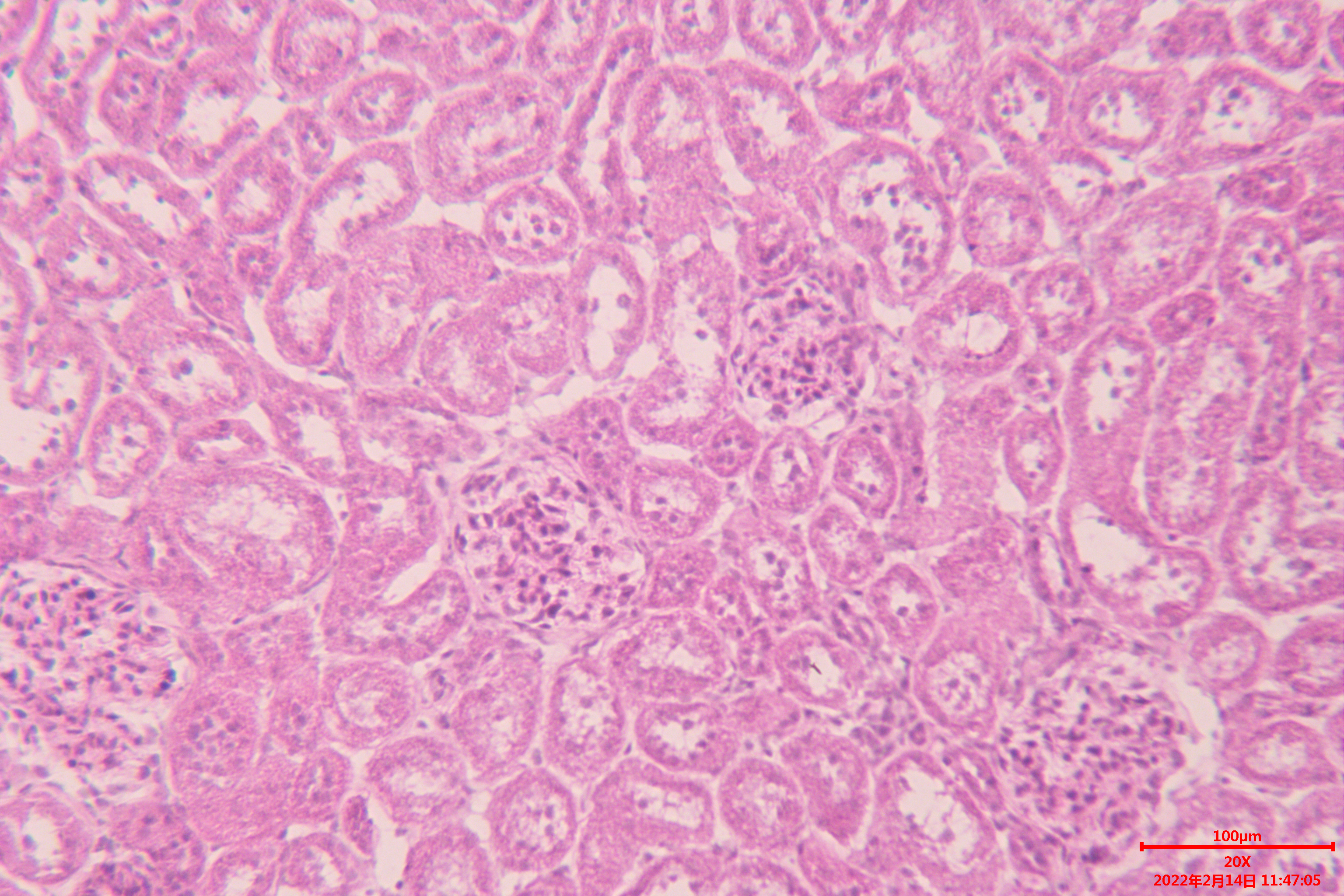

Supplement: Supplementary material — Original Images for Fig S10_2.zip [file IDRD_A_2585599_SM5402.zip › Original Image for Fig S10 G4 (kidney).tif]

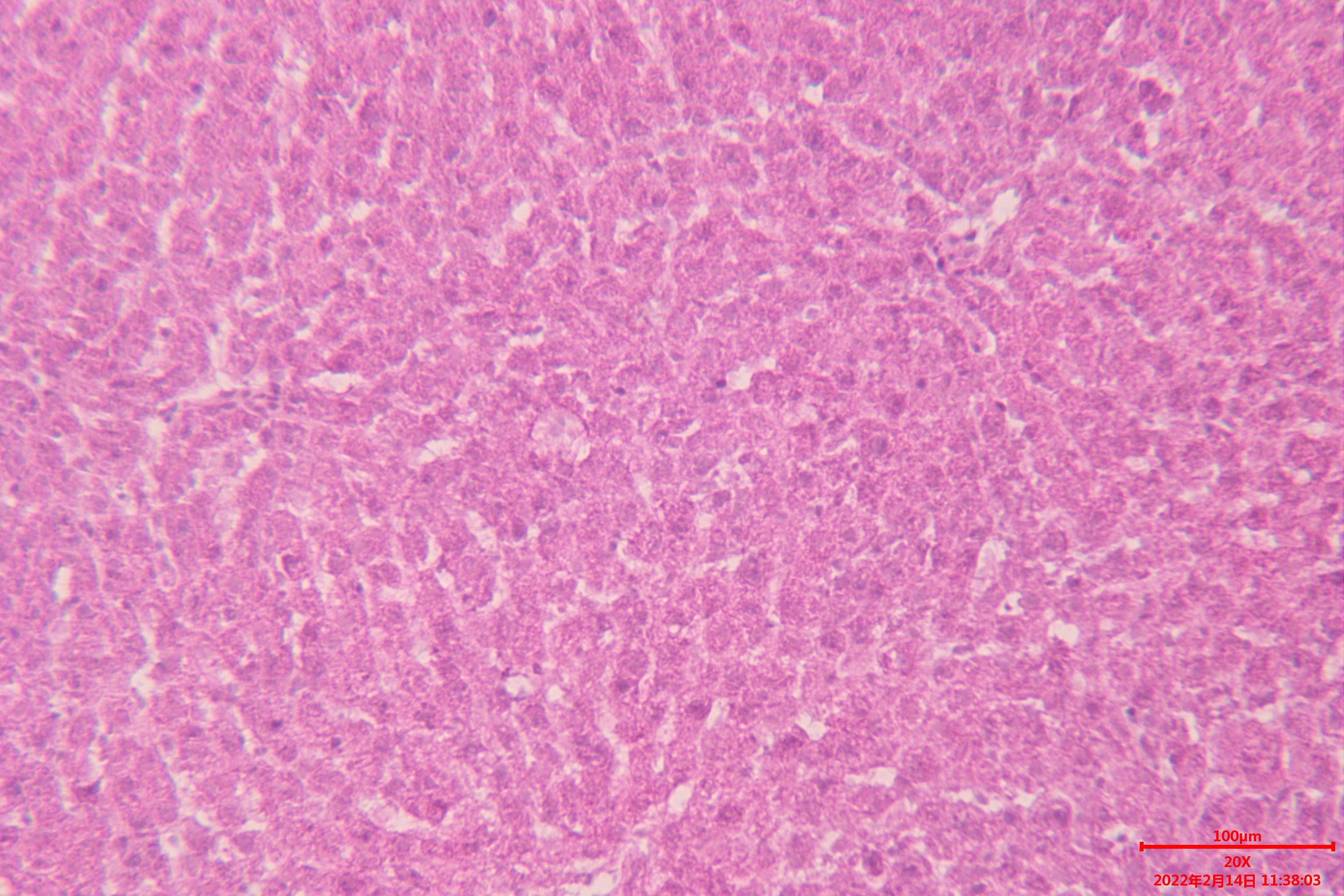

Supplement: Supplementary material — Original Images for Fig S10_2.zip [file IDRD_A_2585599_SM5402.zip › Original Image for Fig S10 G4 (liver).tif]

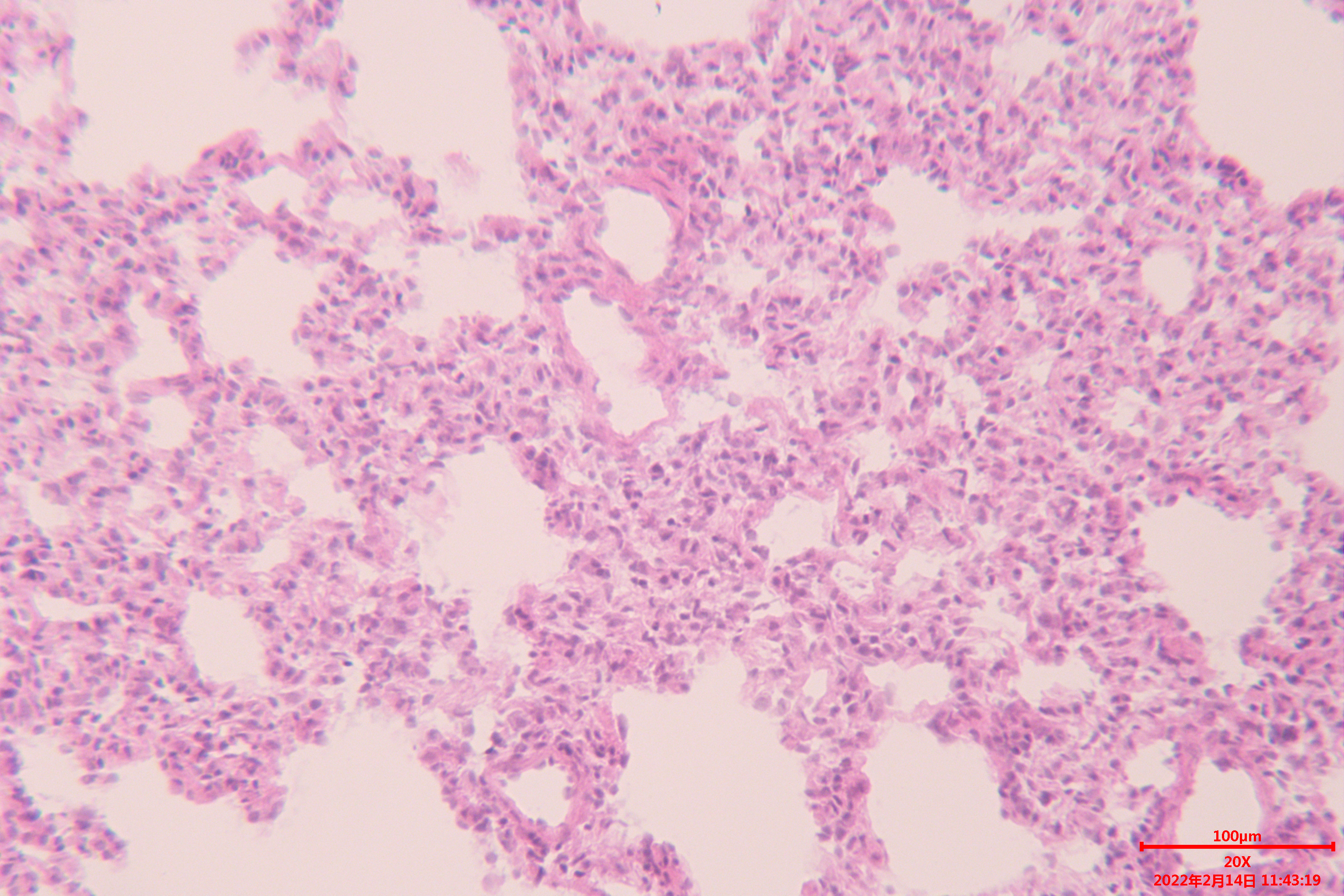

Supplement: Supplementary material — Original Images for Fig S10_2.zip [file IDRD_A_2585599_SM5402.zip › Original Image for Fig S10 G4 (lung).tif]

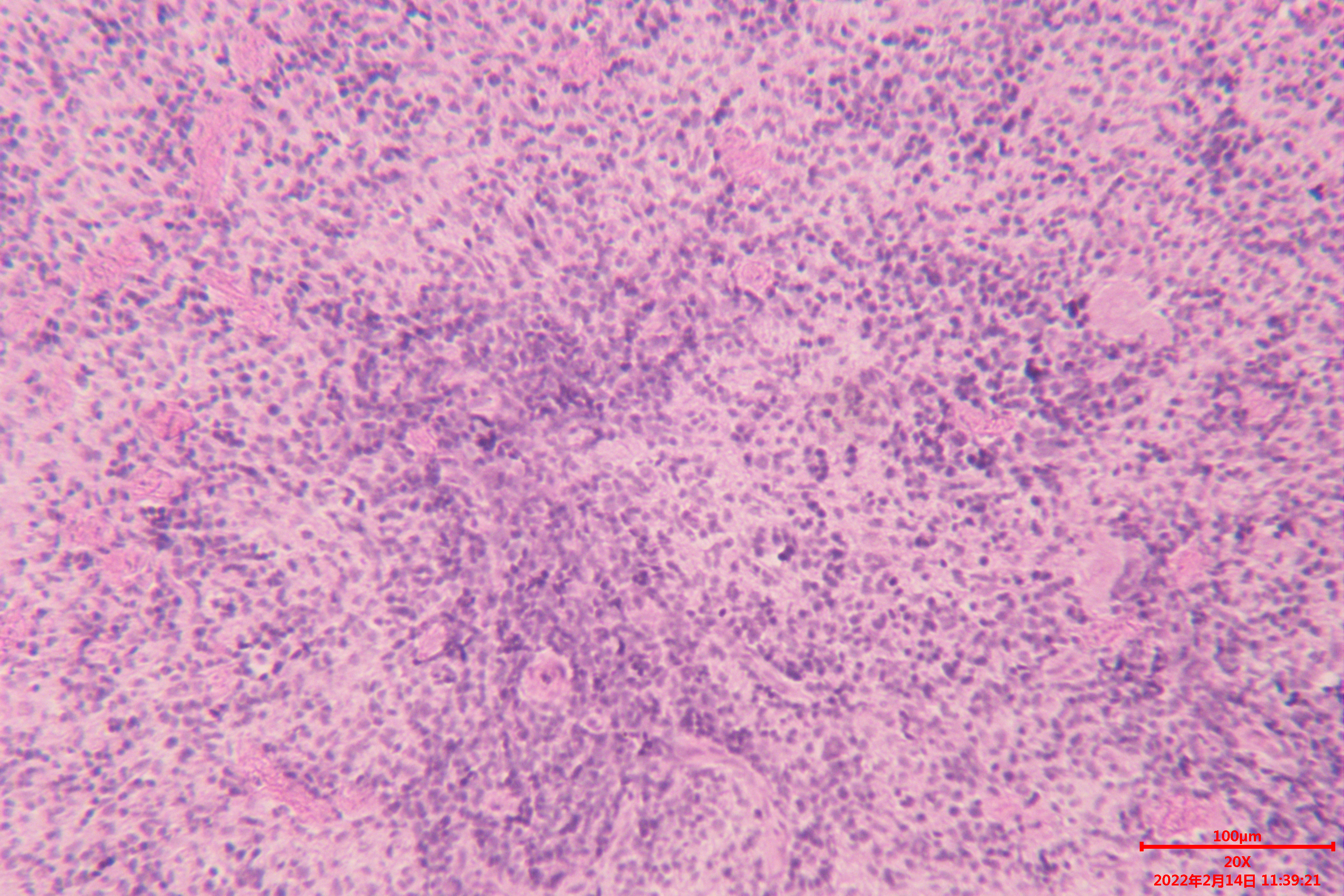

Supplement: Supplementary material — Original Images for Fig S10_2.zip [file IDRD_A_2585599_SM5402.zip › Original Image for Fig S10 G4 (spleen).tif]

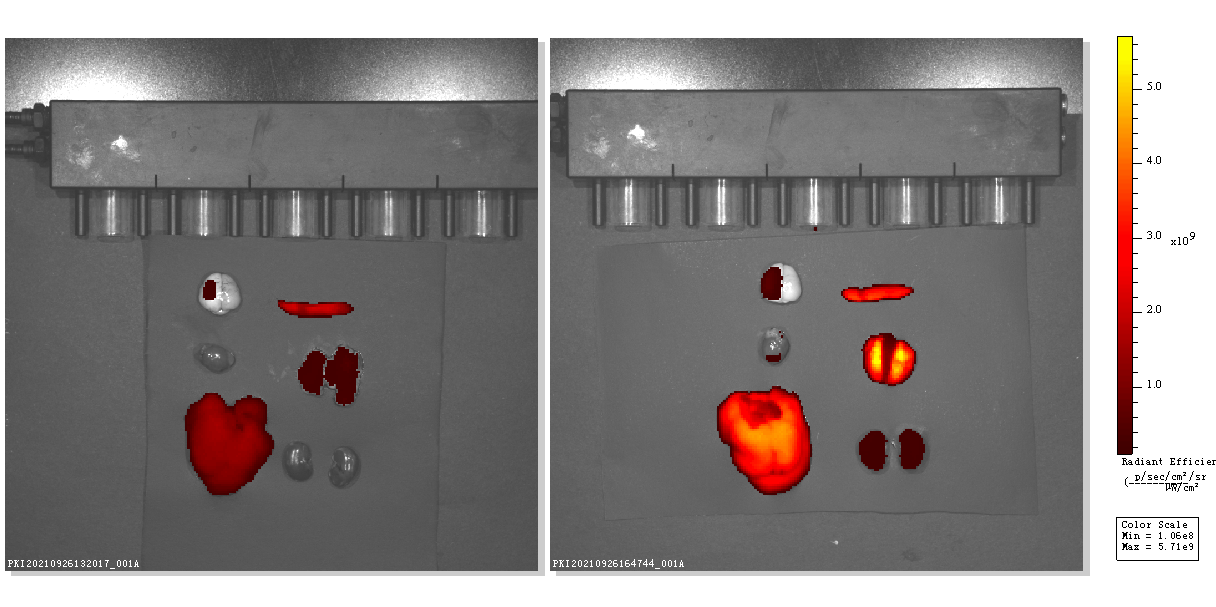

Supplement: Supplementary material — Original Images for Fig 5.zip [file IDRD_A_2585599_SM5403.zip › Original Image for Fig 5A.tif]

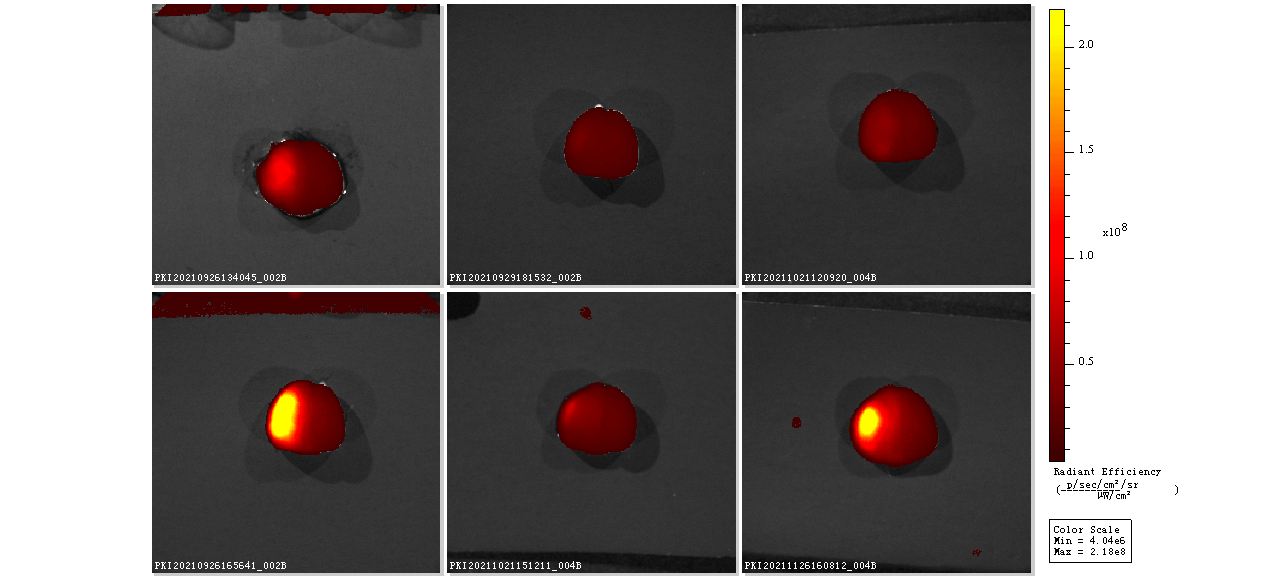

Supplement: Supplementary material — Original Images for Fig 5.zip [file IDRD_A_2585599_SM5403.zip › Original Image for Fig 5B.tif]

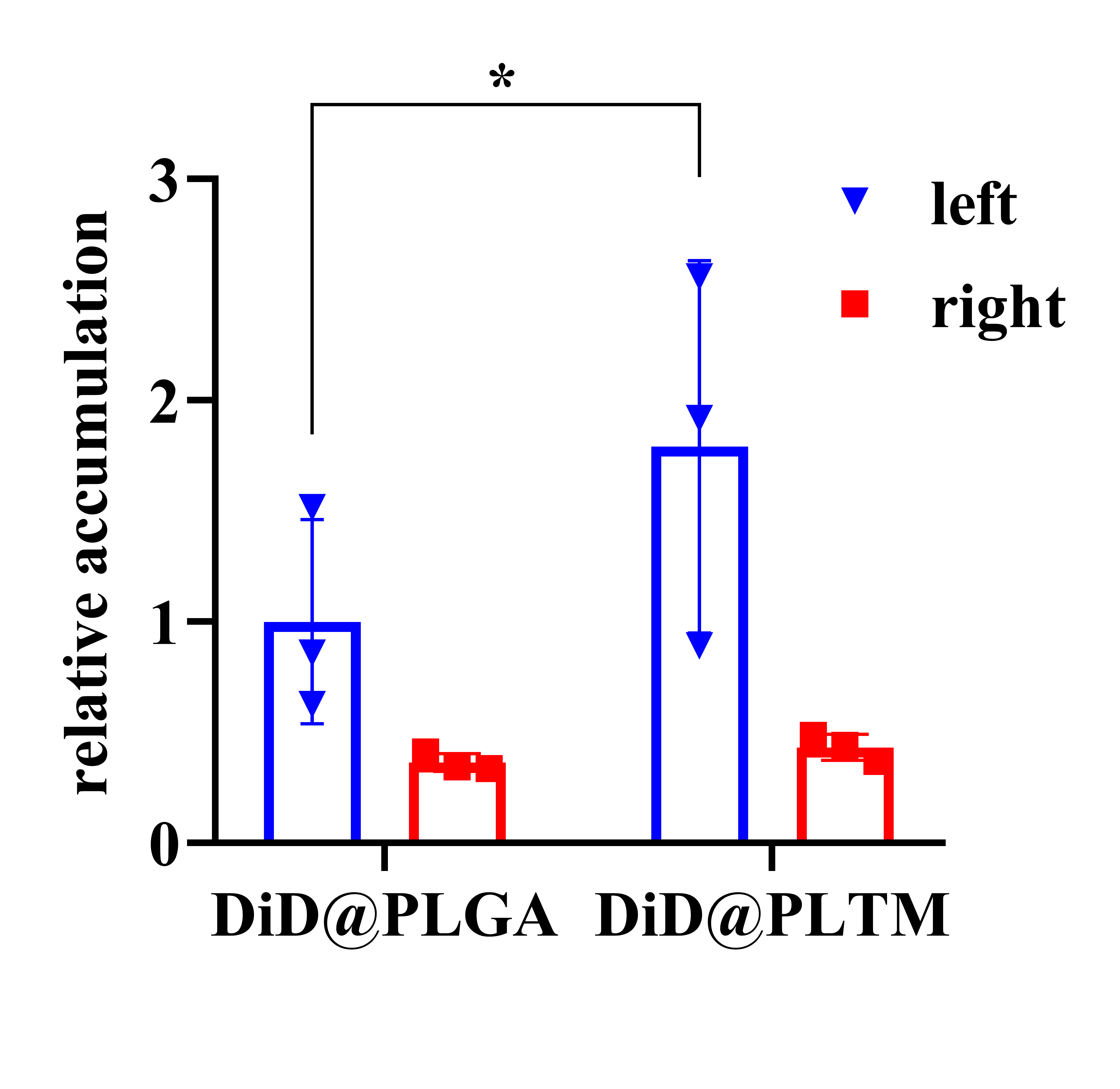

Supplement: Supplementary material — Original Images for Fig 5.zip [file IDRD_A_2585599_SM5403.zip › Original Image for Fig 5C.tif]

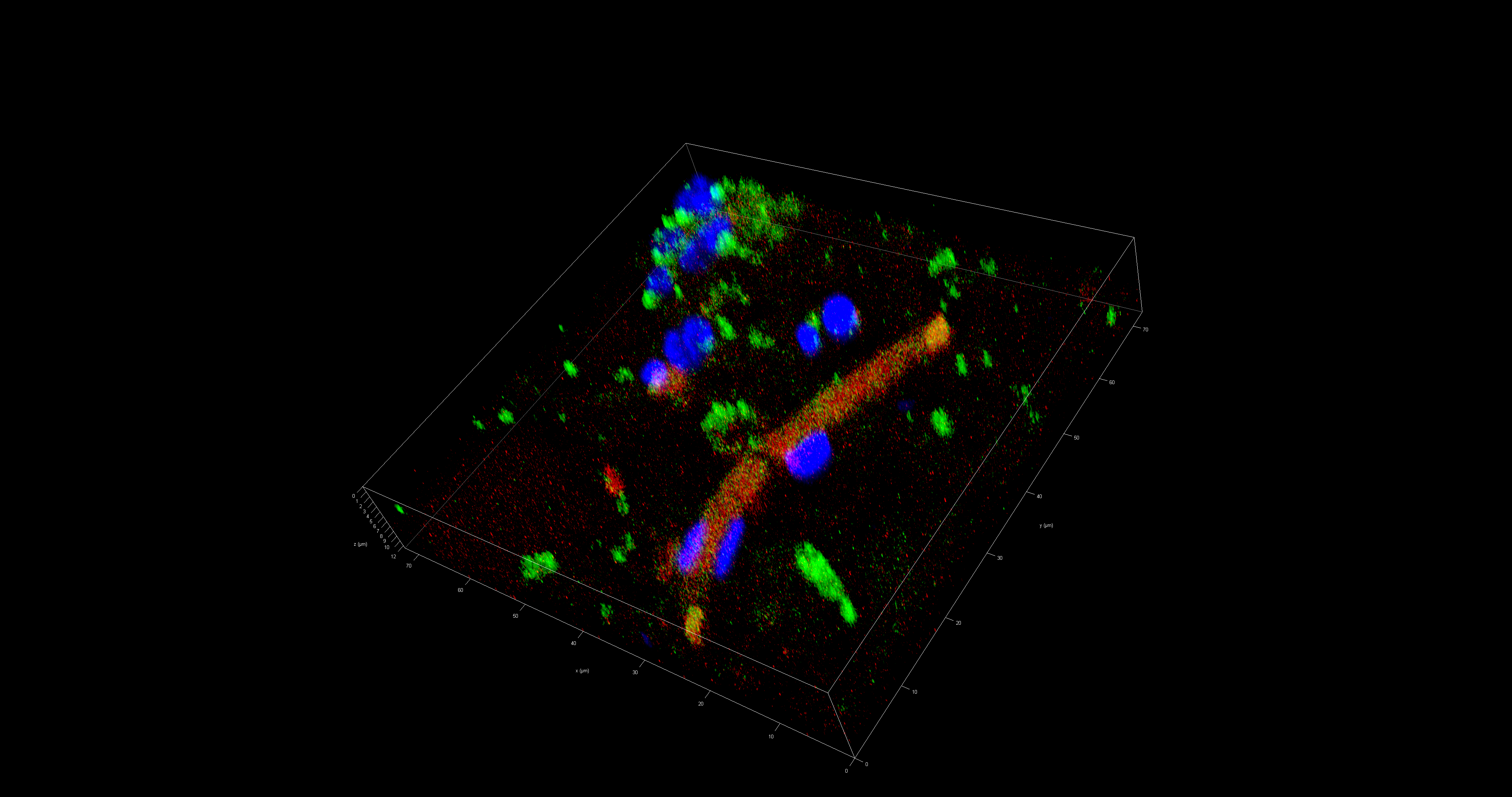

Supplement: Supplementary material — Original Images for Fig 5.zip [file IDRD_A_2585599_SM5403.zip › Original Image for Fig 5D CD31 DiD@PLGA.tif]

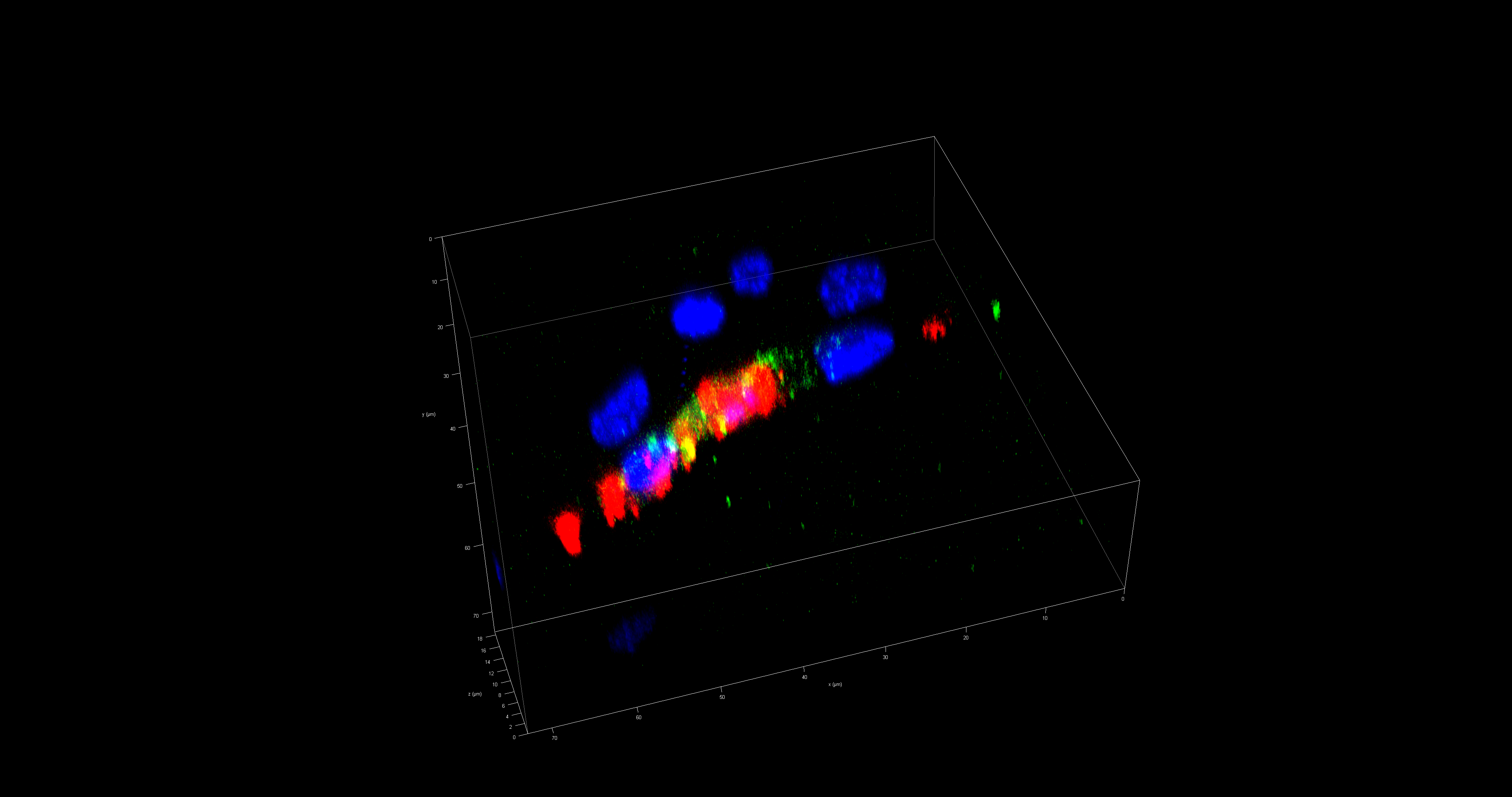

Supplement: Supplementary material — Original Images for Fig 5.zip [file IDRD_A_2585599_SM5403.zip › Original Image for Fig 5D CD31 DiD@PLTM.tif]

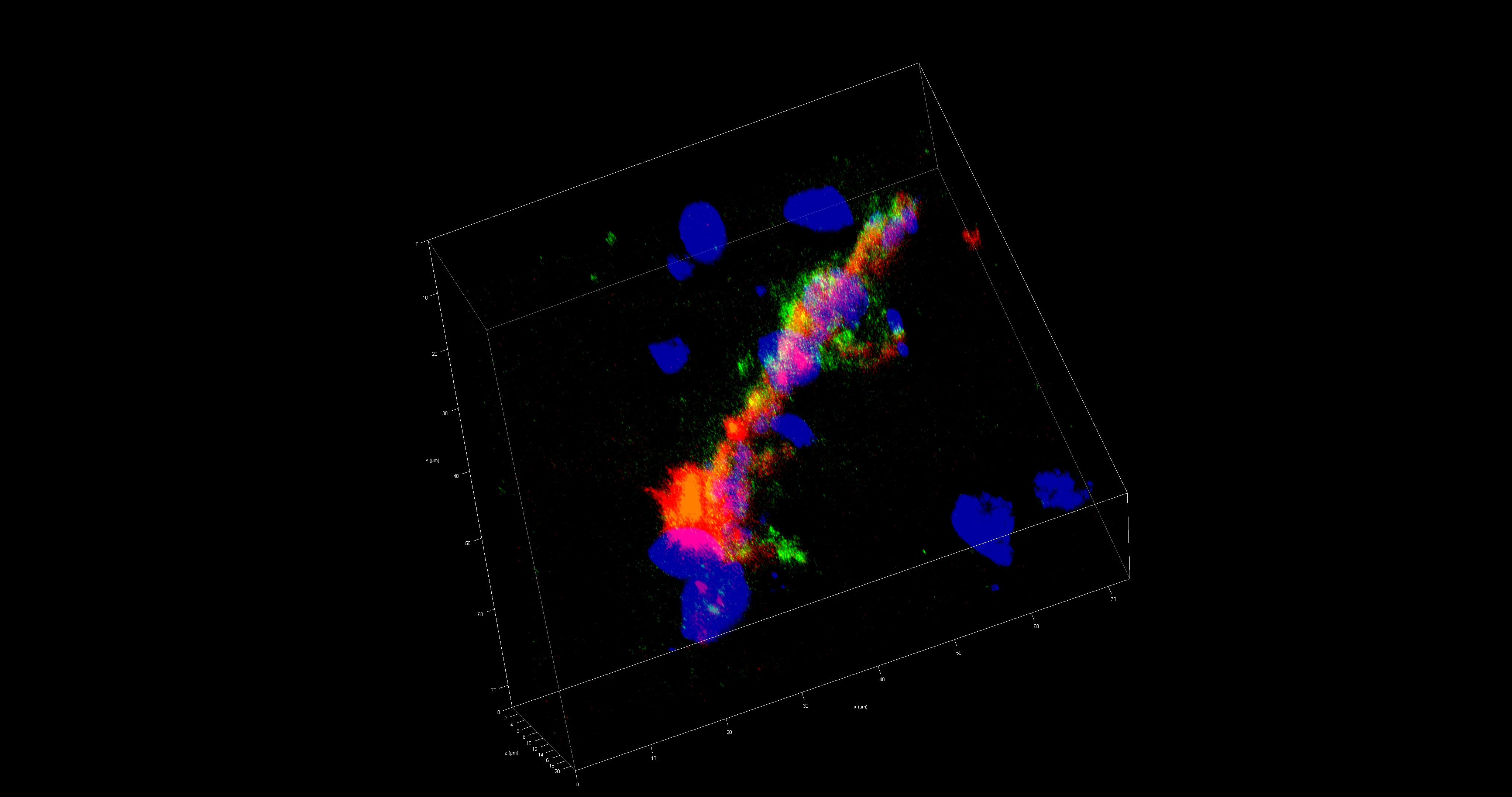

Supplement: Supplementary material — Original Images for Fig 5.zip [file IDRD_A_2585599_SM5403.zip › Original Image for Fig 5D GFAP DiD@PLGA.tif]

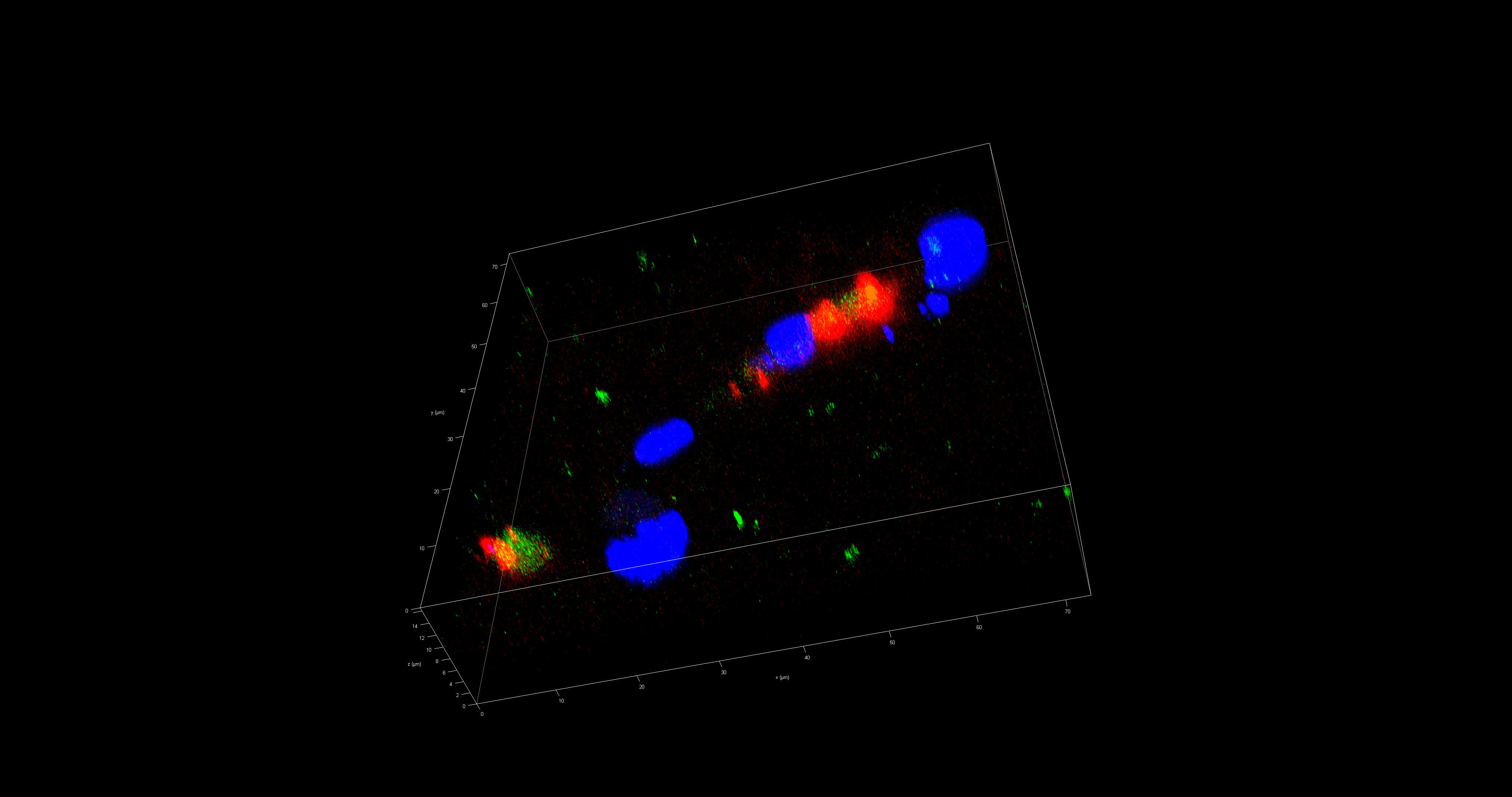

Supplement: Supplementary material — Original Images for Fig 5.zip [file IDRD_A_2585599_SM5403.zip › Original Image for Fig 5D GFAP DiD@PLTM.tif]

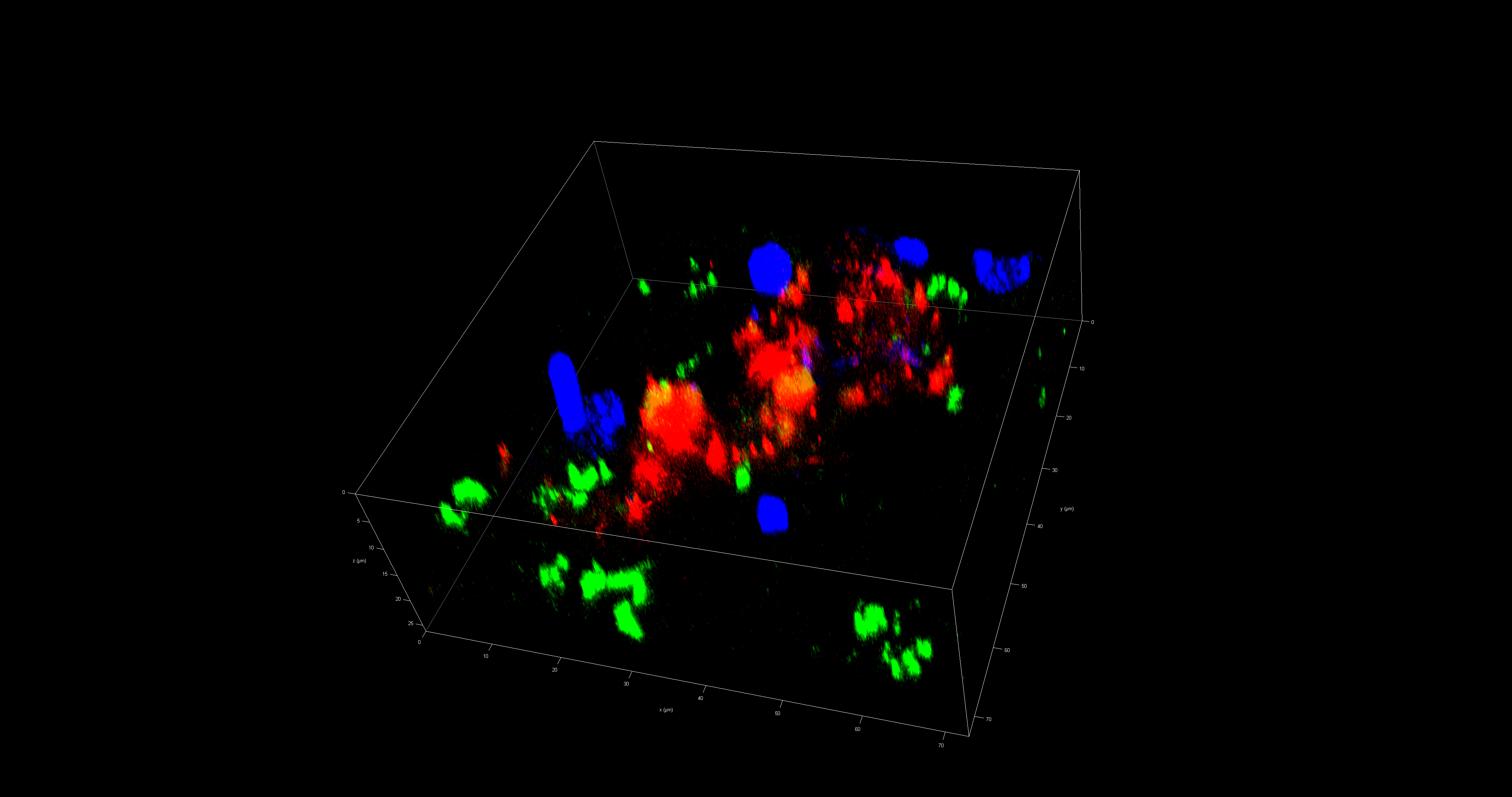

Supplement: Supplementary material — Original Images for Fig 5.zip [file IDRD_A_2585599_SM5403.zip › Original Image for Fig 5D Iba-1 DiD@PLGA.tif]

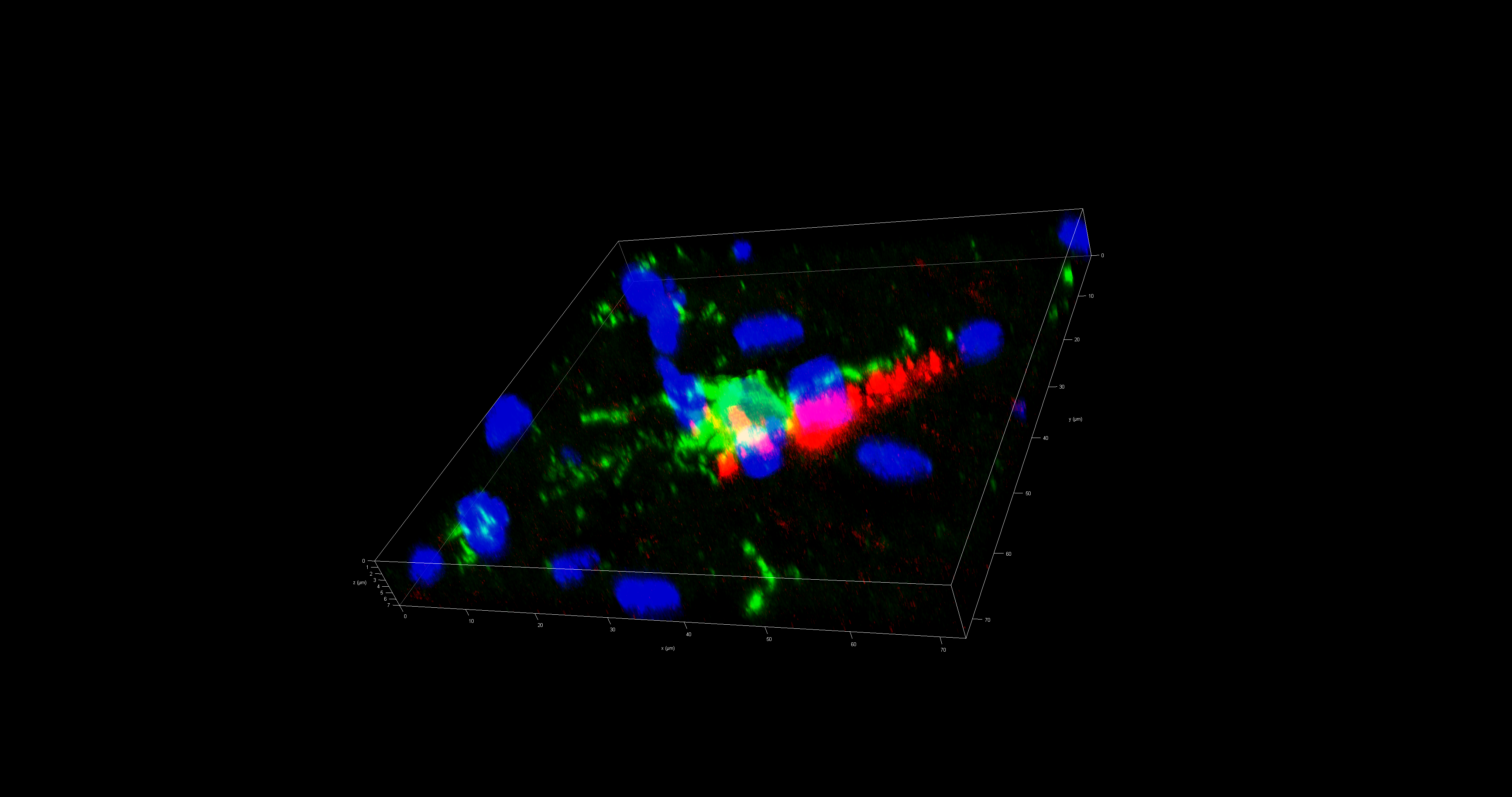

Supplement: Supplementary material — Original Images for Fig 5.zip [file IDRD_A_2585599_SM5403.zip › Original Image for Fig 5D Iba-1 DiD@PLTM.tif]

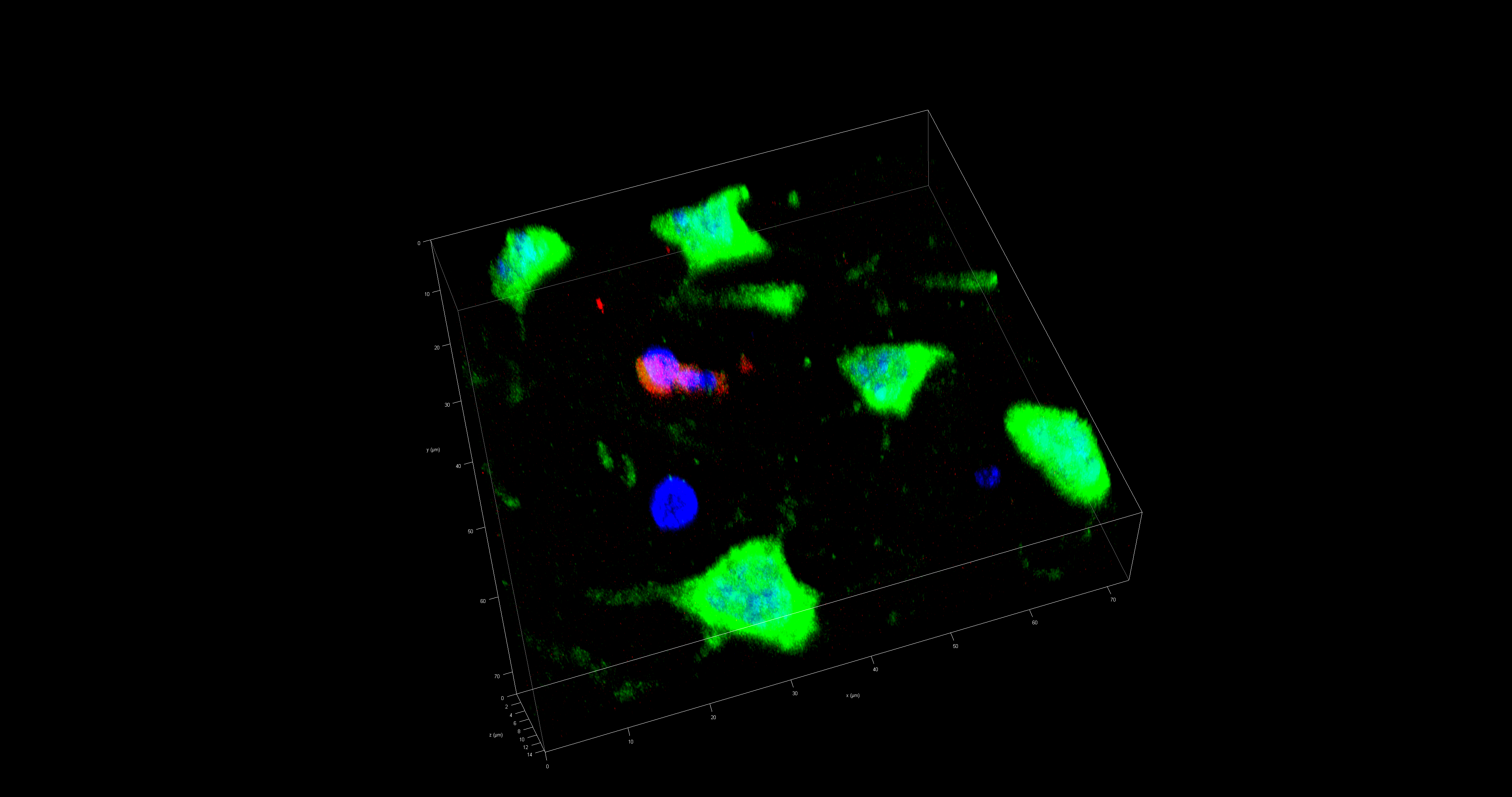

Supplement: Supplementary material — Original Images for Fig 5.zip [file IDRD_A_2585599_SM5403.zip › Original Image for Fig 5D NeuN DiD@PLGA.tif]

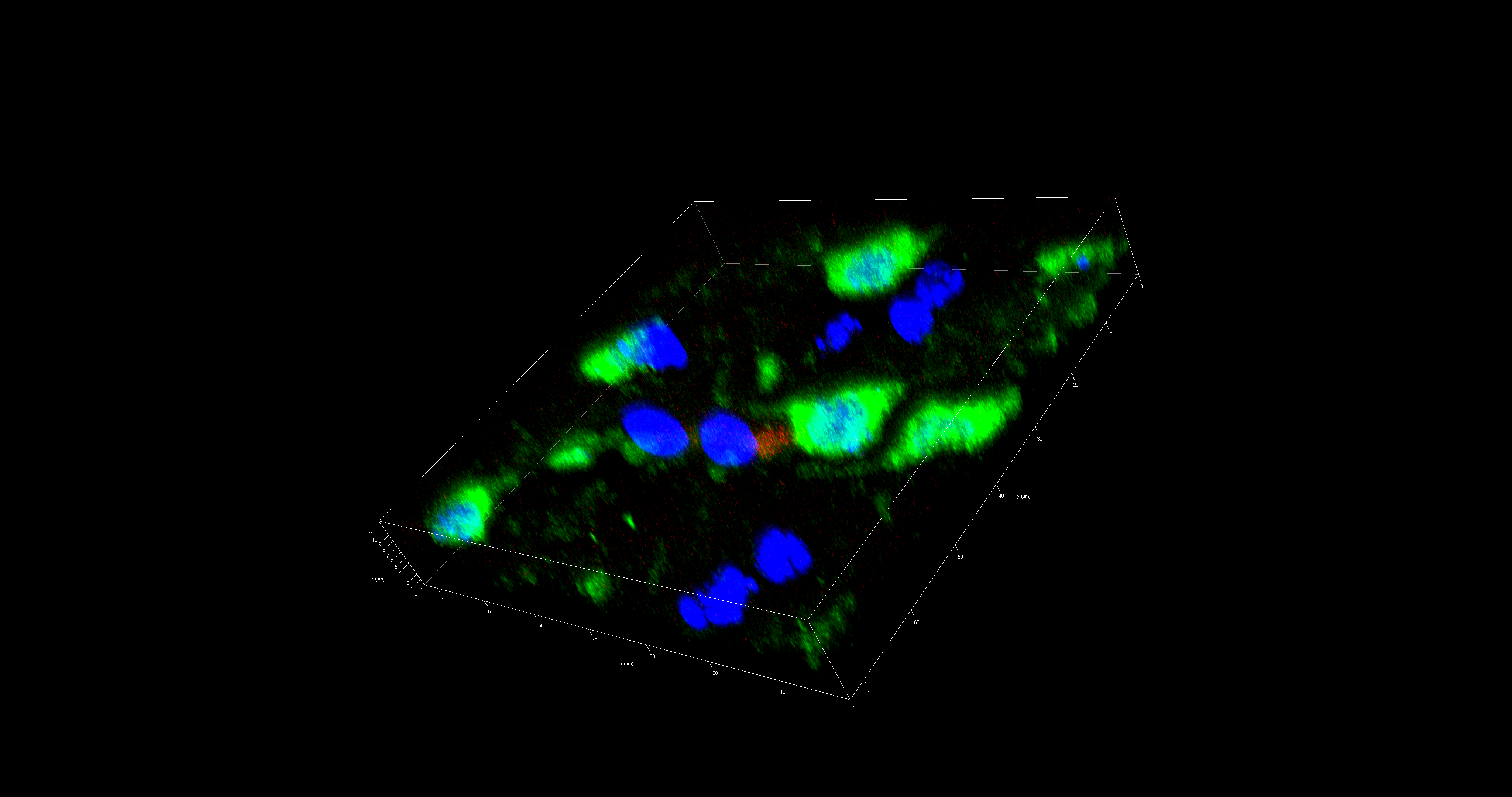

Supplement: Supplementary material — Original Images for Fig 5.zip [file IDRD_A_2585599_SM5403.zip › Original Image for Fig 5D NeuN DiD@PLTM.tif]

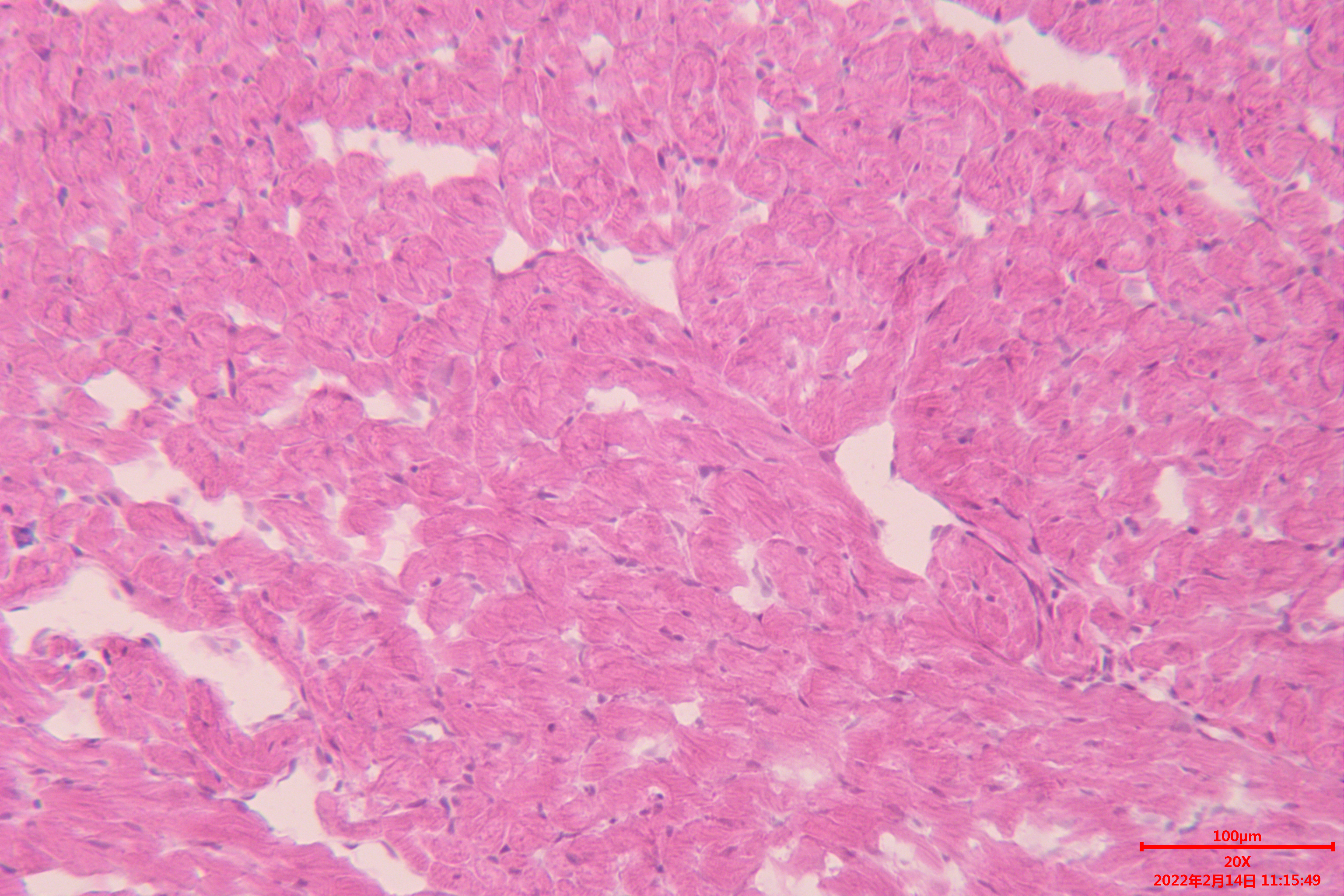

Supplement: Supplementary material — Original Images for Fig S10_3.zip [file IDRD_A_2585599_SM5404.zip › Original Image for Fig S10 G5 (heart).tif]

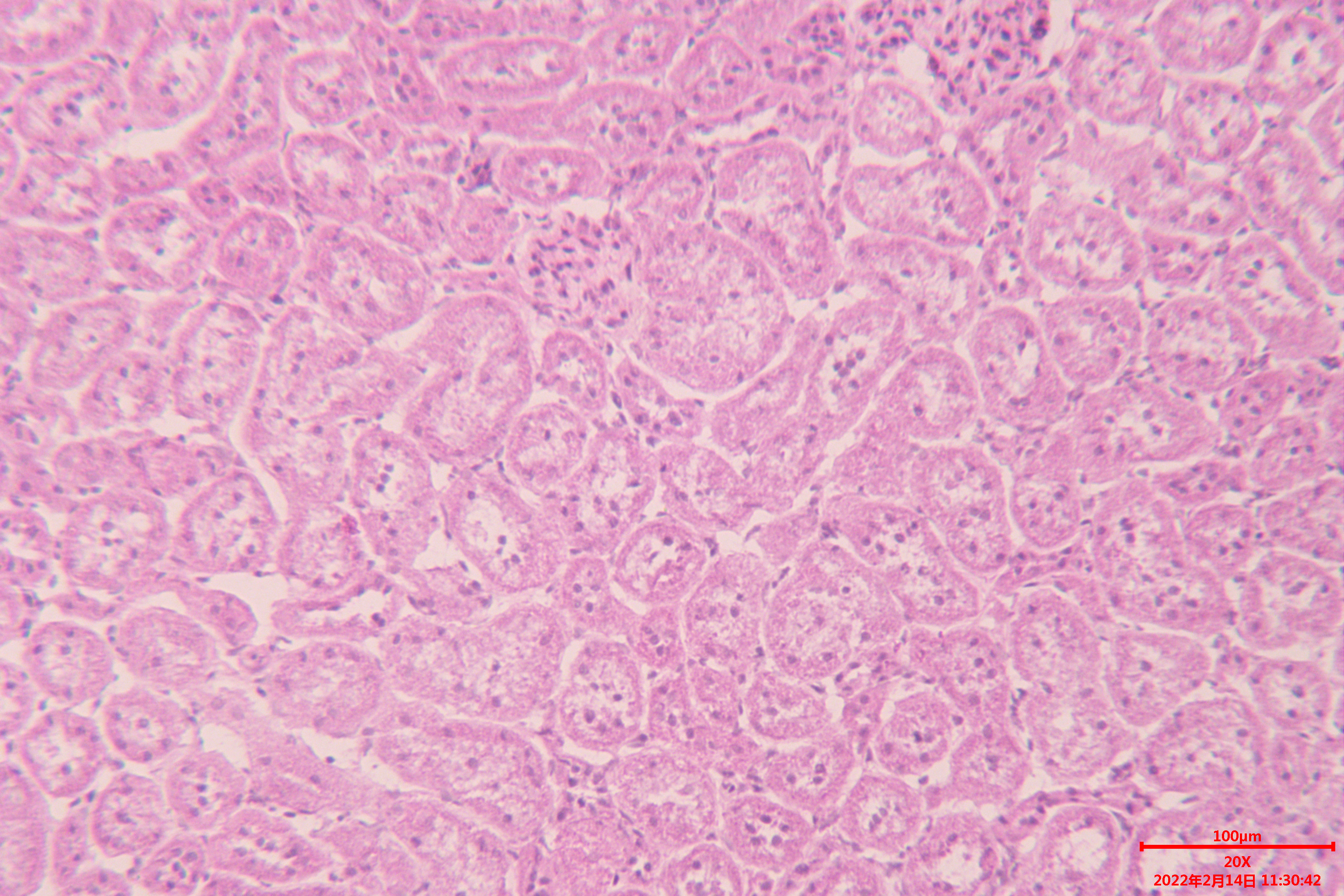

Supplement: Supplementary material — Original Images for Fig S10_3.zip [file IDRD_A_2585599_SM5404.zip › Original Image for Fig S10 G5 (kidney).tif]

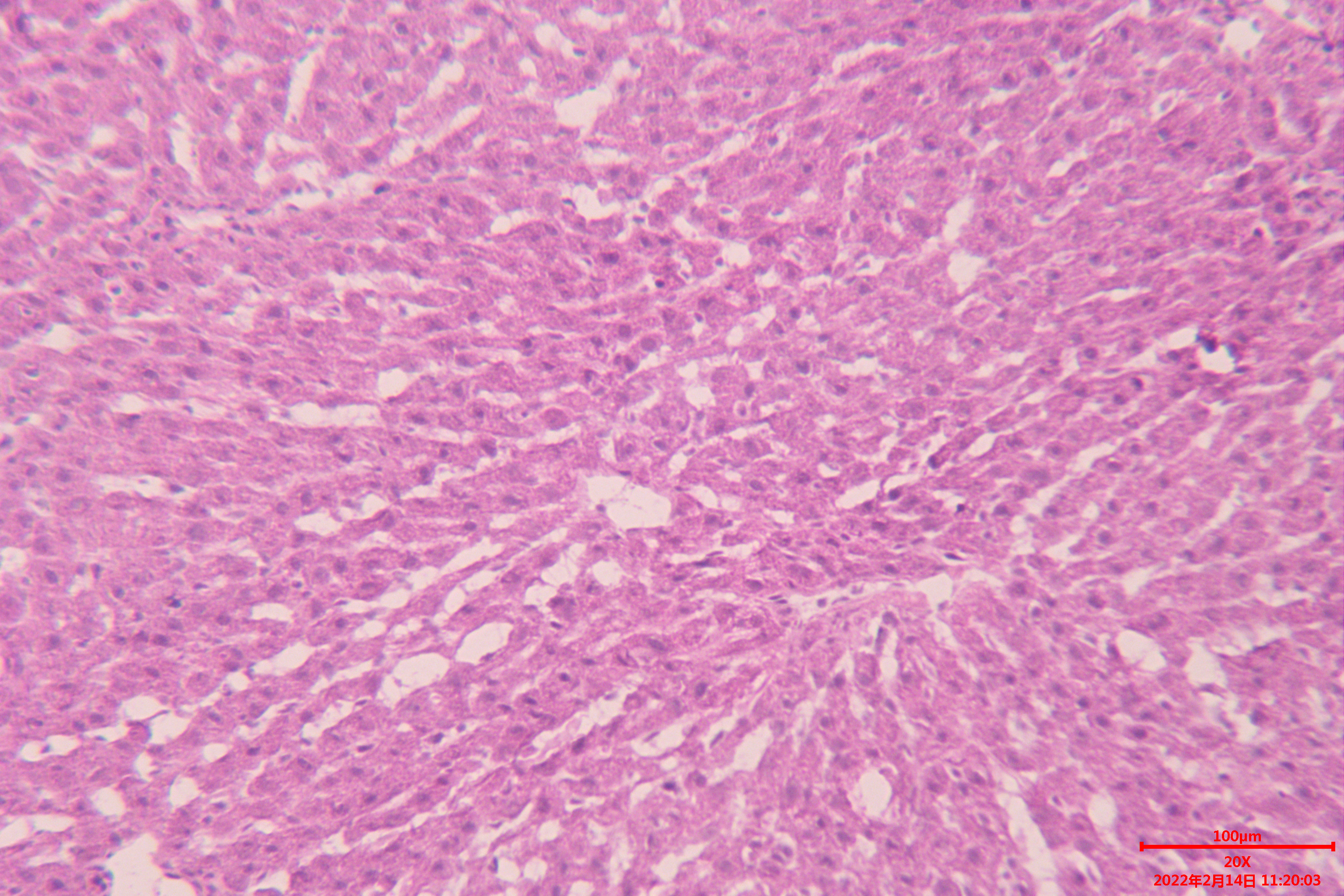

Supplement: Supplementary material — Original Images for Fig S10_3.zip [file IDRD_A_2585599_SM5404.zip › Original Image for Fig S10 G5 (liver).tif]

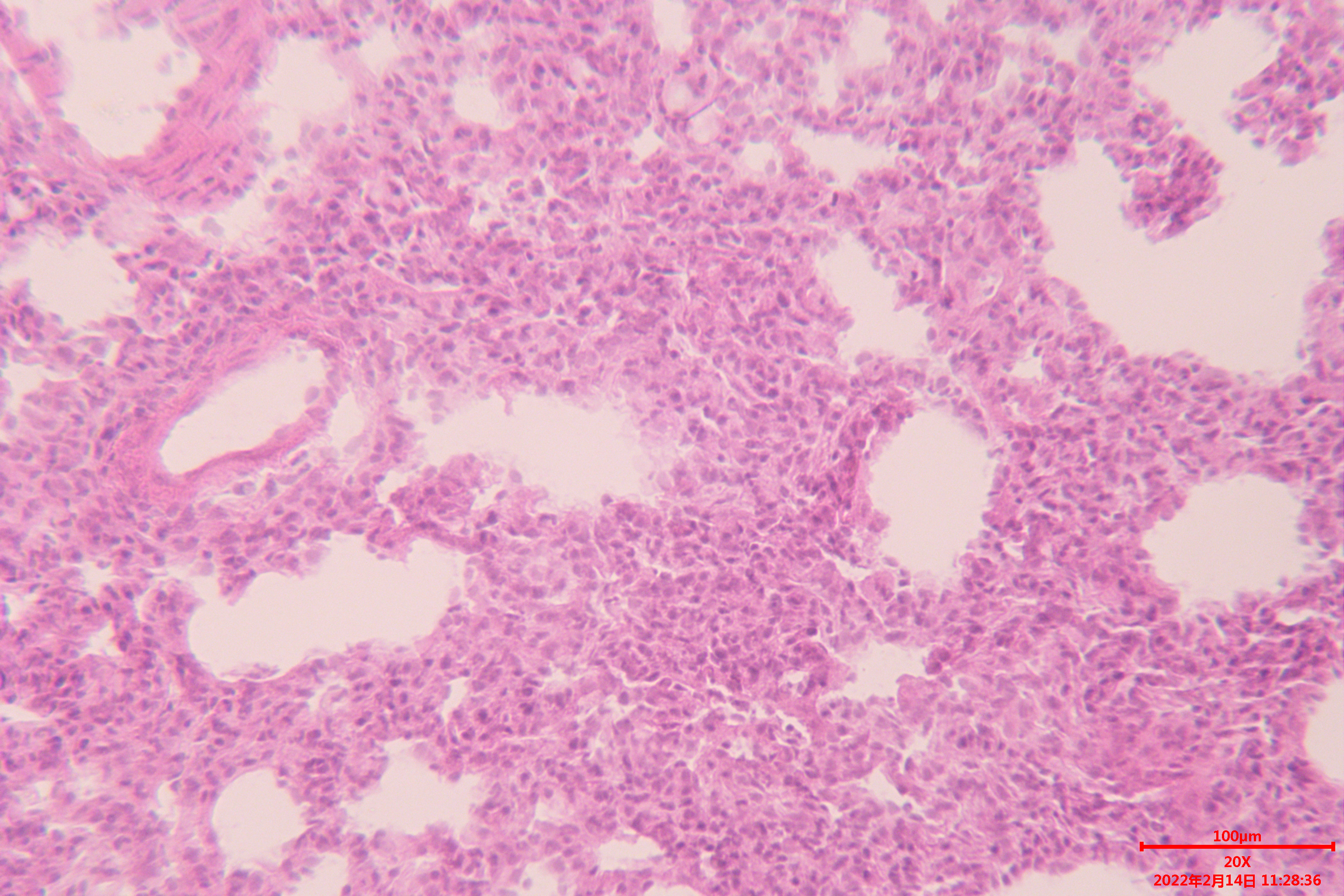

Supplement: Supplementary material — Original Images for Fig S10_3.zip [file IDRD_A_2585599_SM5404.zip › Original Image for Fig S10 G5 (lung).tif]

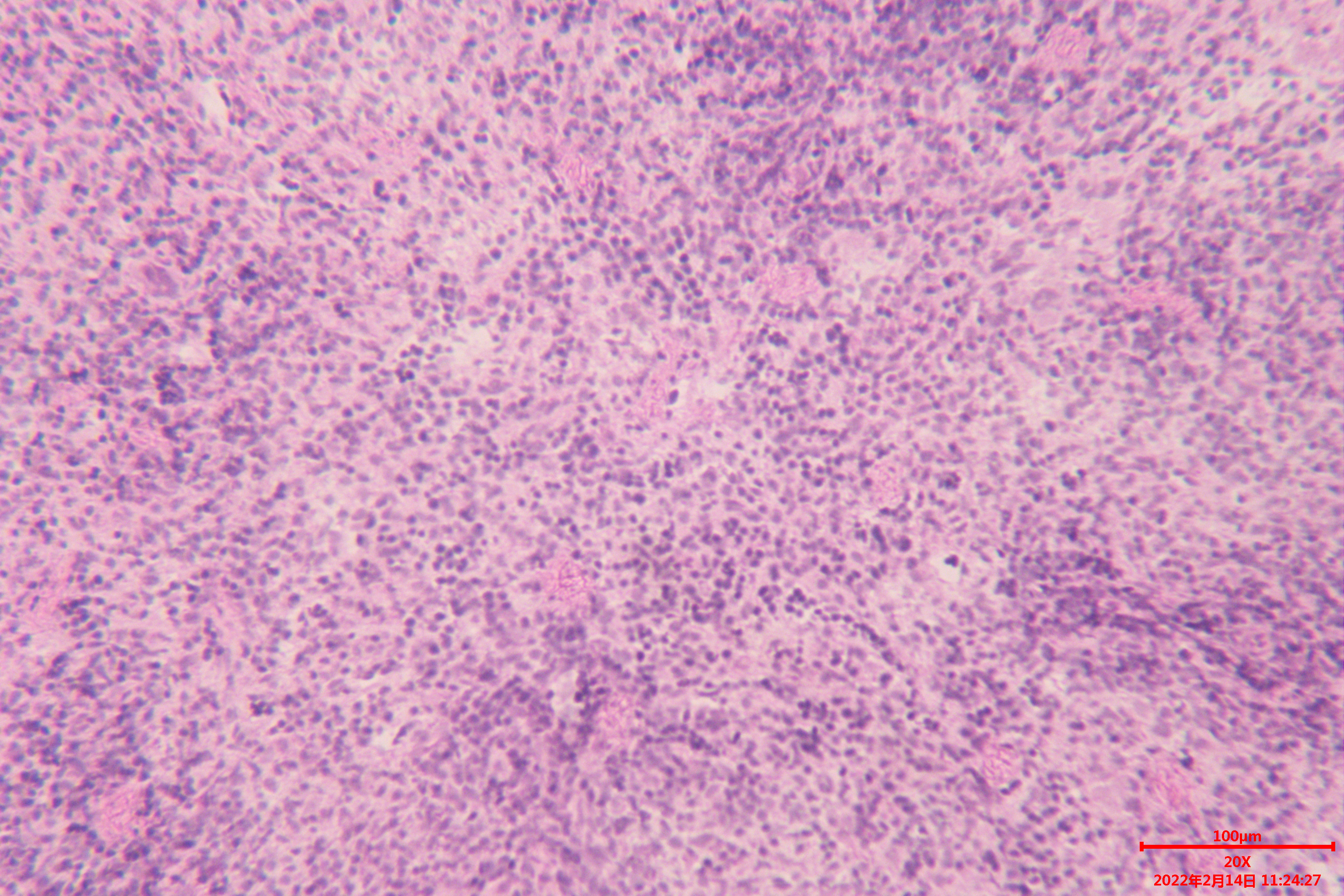

Supplement: Supplementary material — Original Images for Fig S10_3.zip [file IDRD_A_2585599_SM5404.zip › Original Image for Fig S10 G5 (spleen).tif]

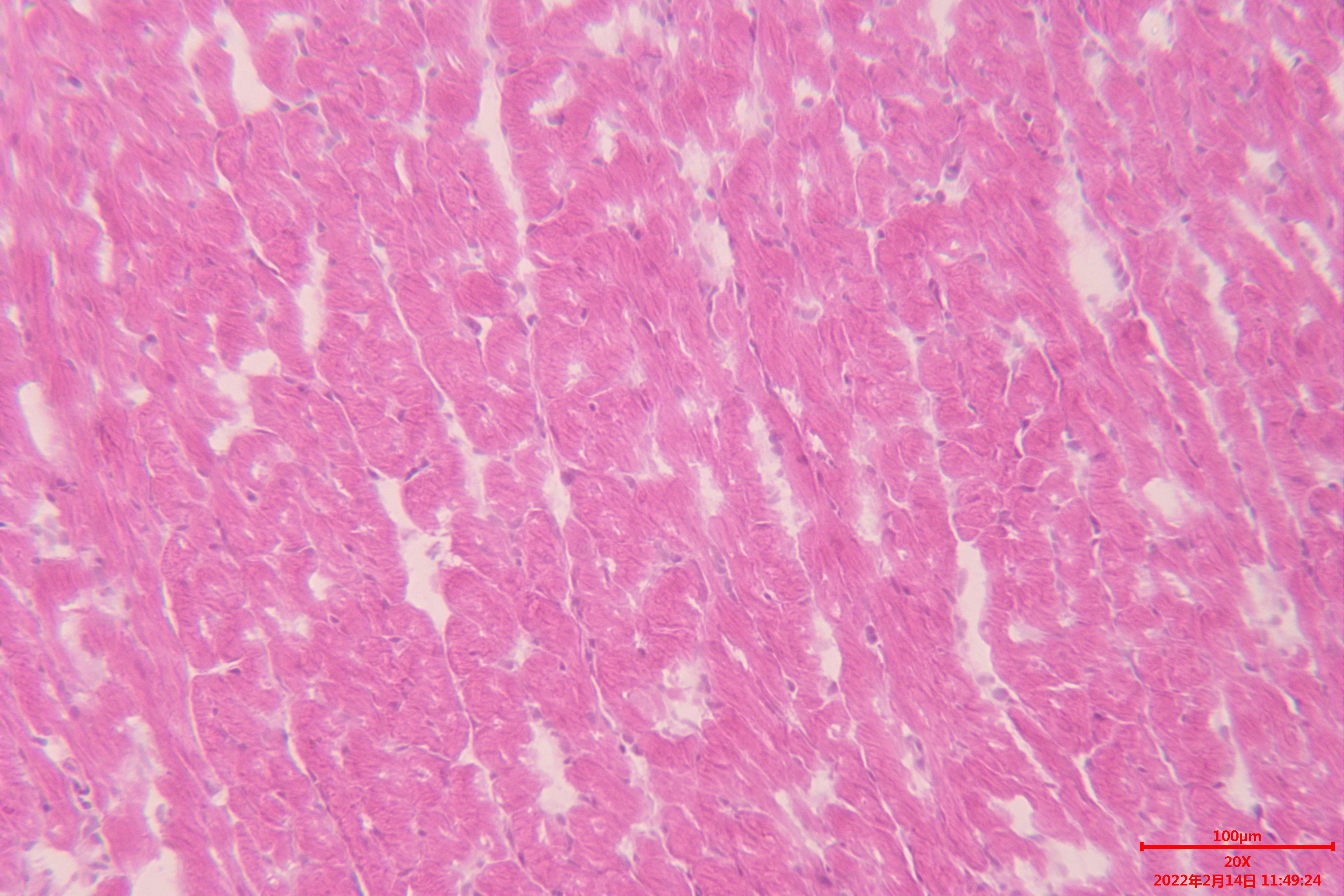

Supplement: Supplementary material — Original Images for Fig S10_3.zip [file IDRD_A_2585599_SM5404.zip › Original Image for Fig S10 G6 (heart).tif]

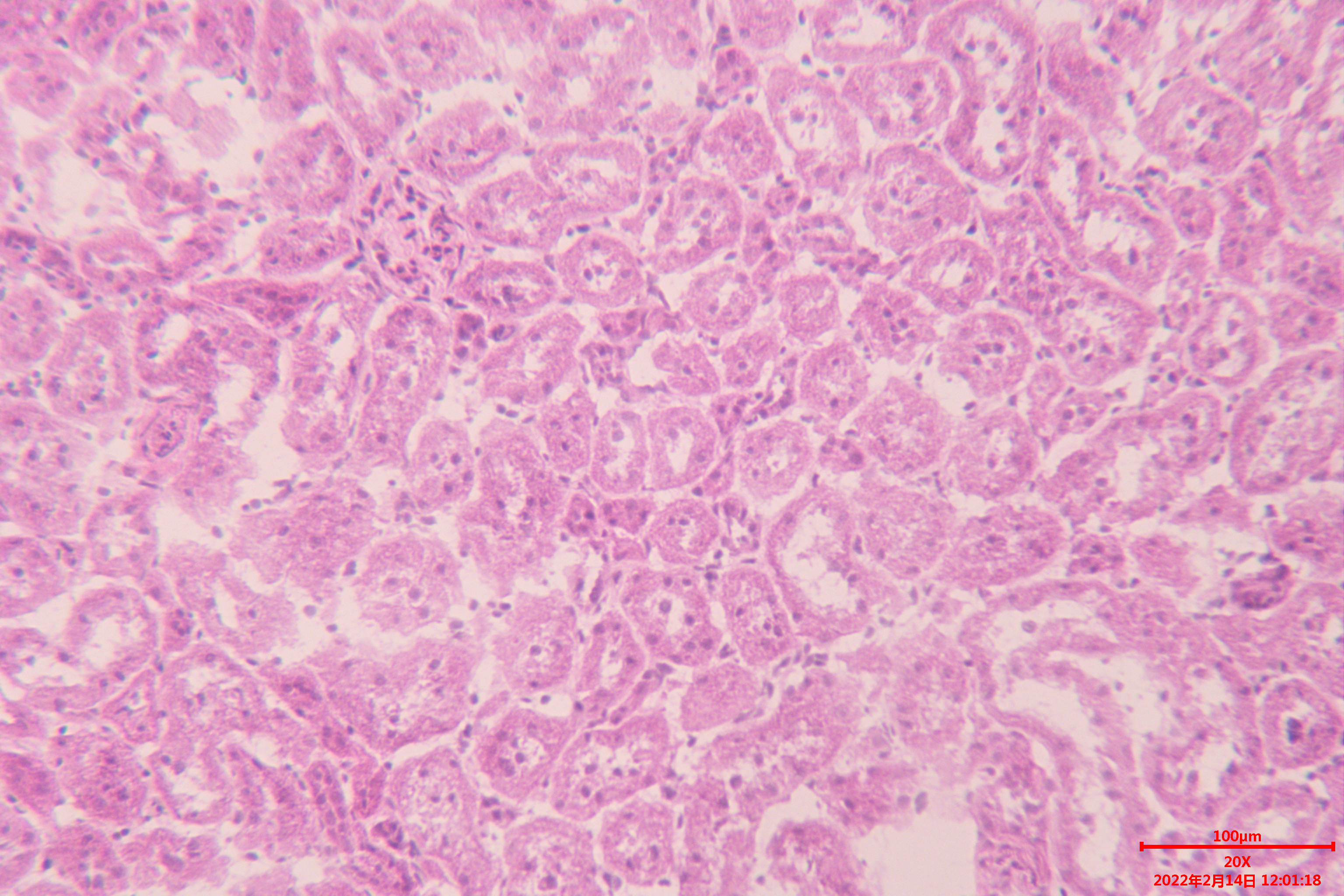

Supplement: Supplementary material — Original Images for Fig S10_3.zip [file IDRD_A_2585599_SM5404.zip › Original Image for Fig S10 G6 (kidney).tif]

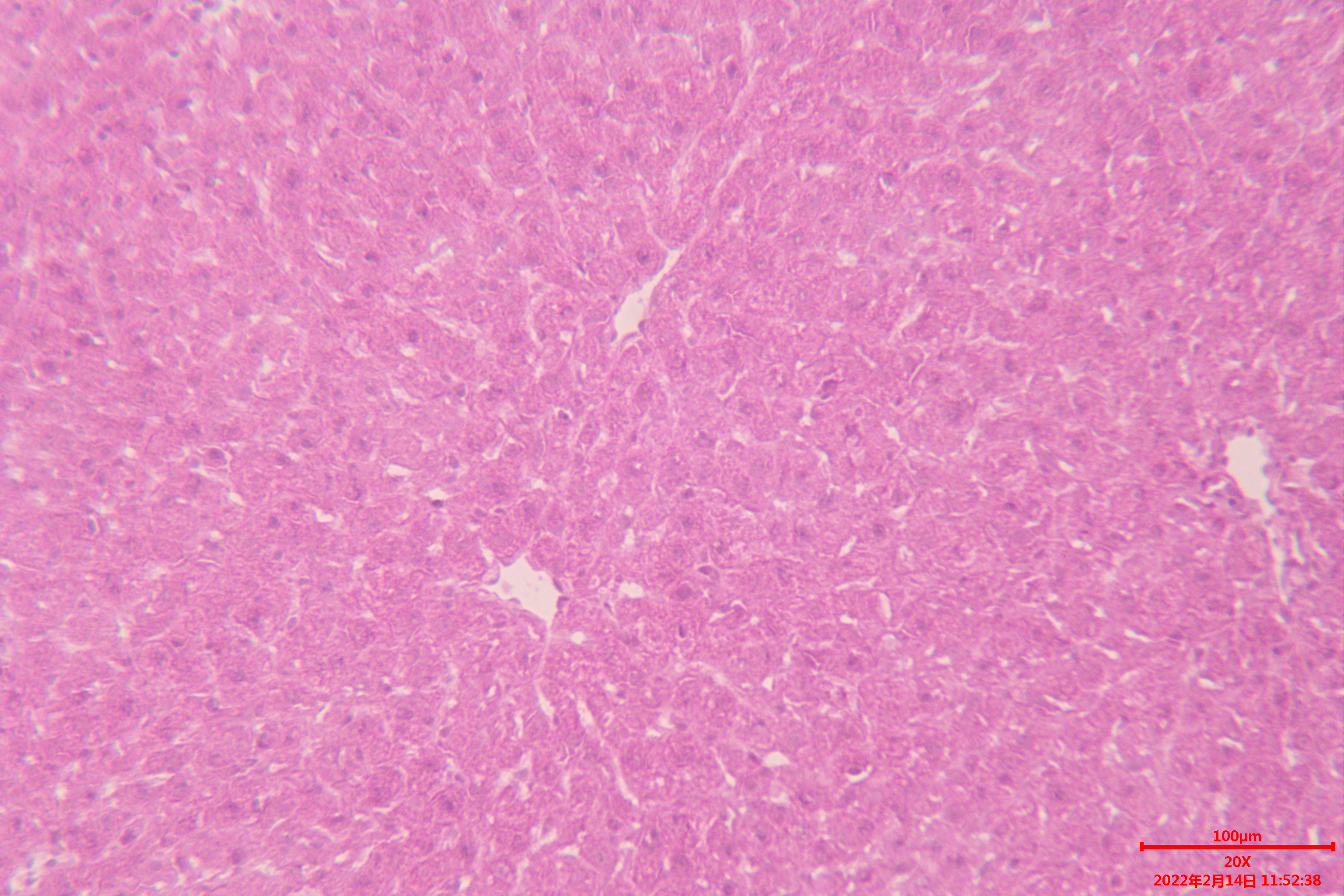

Supplement: Supplementary material — Original Images for Fig S10_3.zip [file IDRD_A_2585599_SM5404.zip › Original Image for Fig S10 G6 (liver).tif]

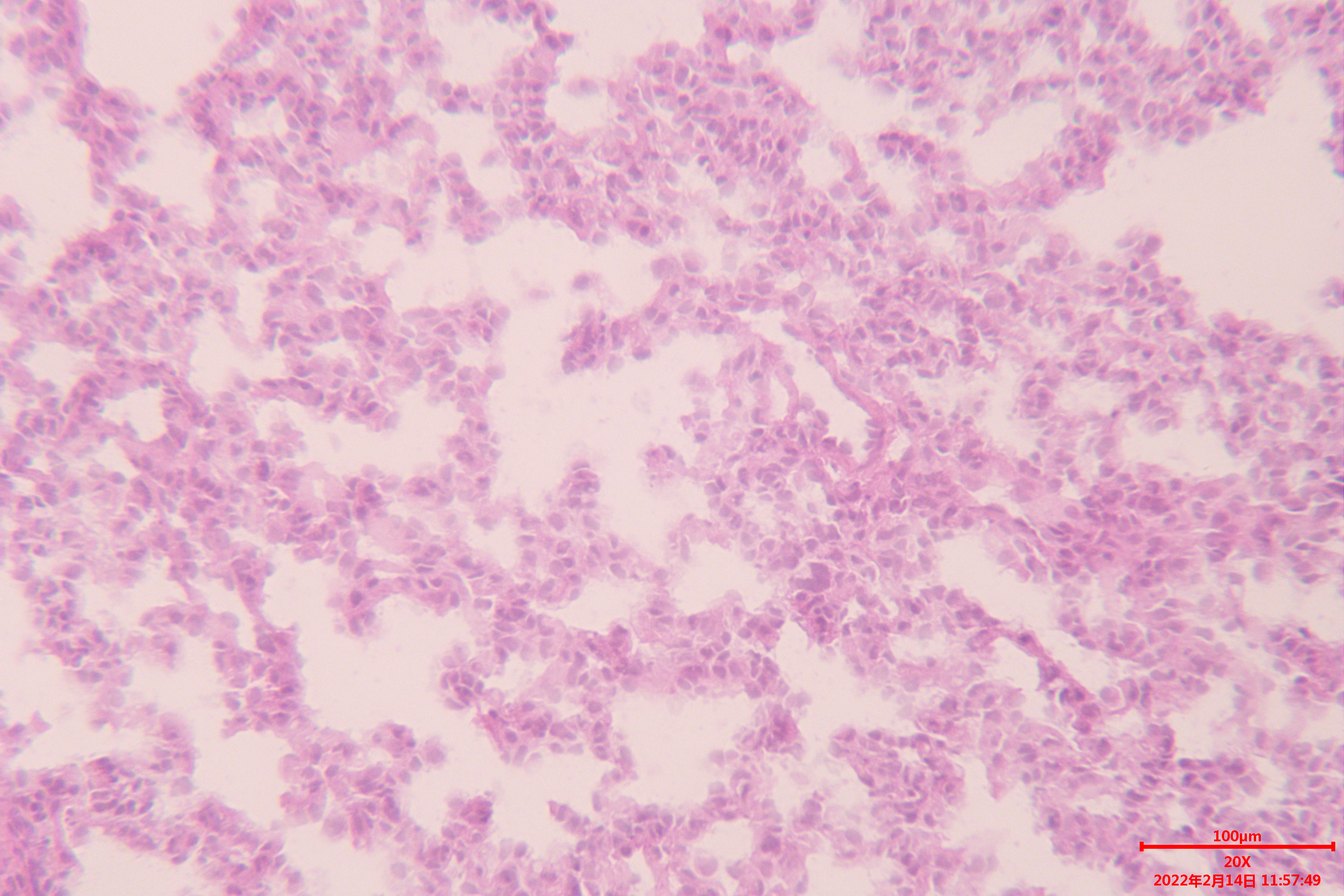

Supplement: Supplementary material — Original Images for Fig S10_3.zip [file IDRD_A_2585599_SM5404.zip › Original Image for Fig S10 G6 (lung).tif]

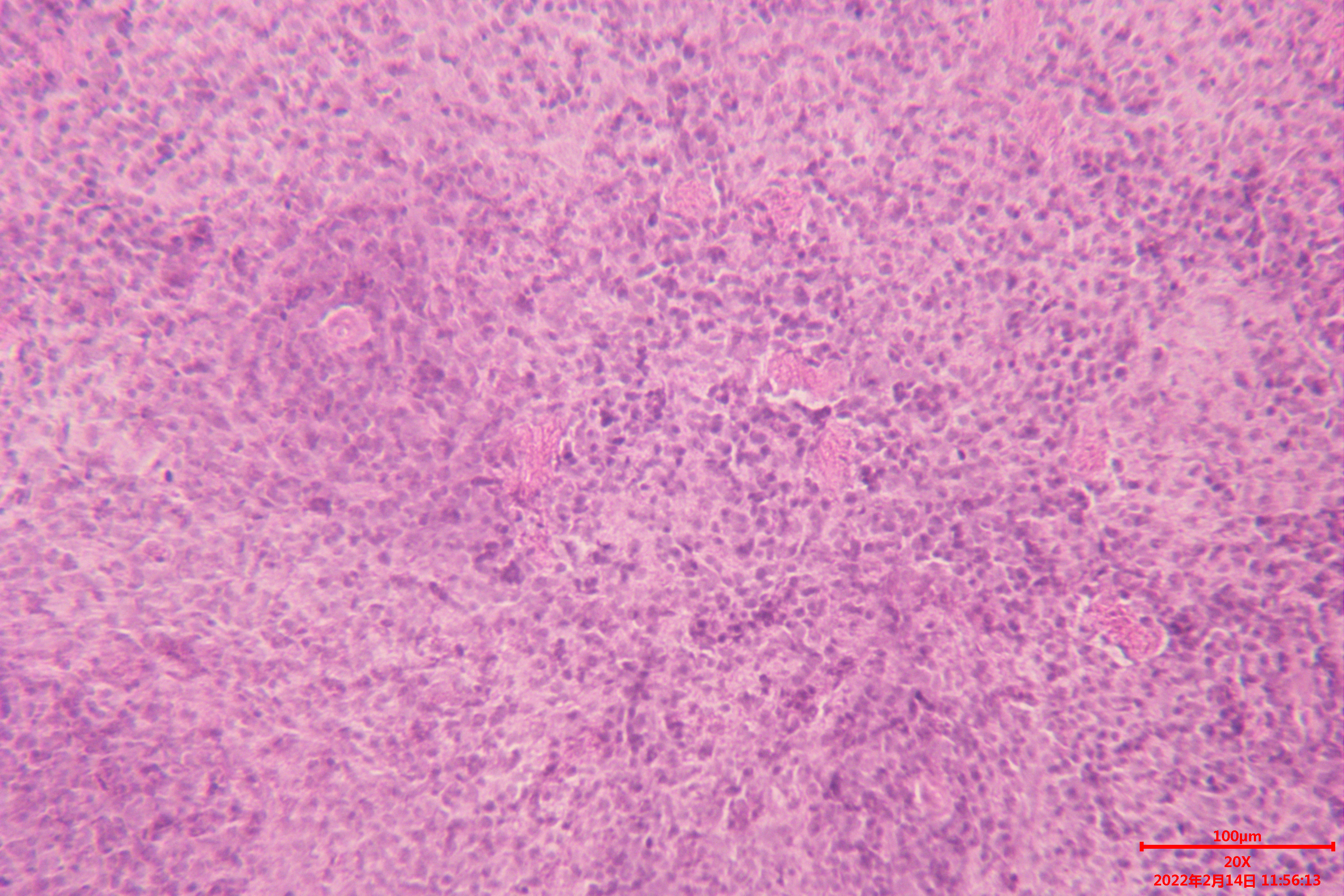

Supplement: Supplementary material — Original Images for Fig S10_3.zip [file IDRD_A_2585599_SM5404.zip › Original Image for Fig S10 G6 (spleen).tif]

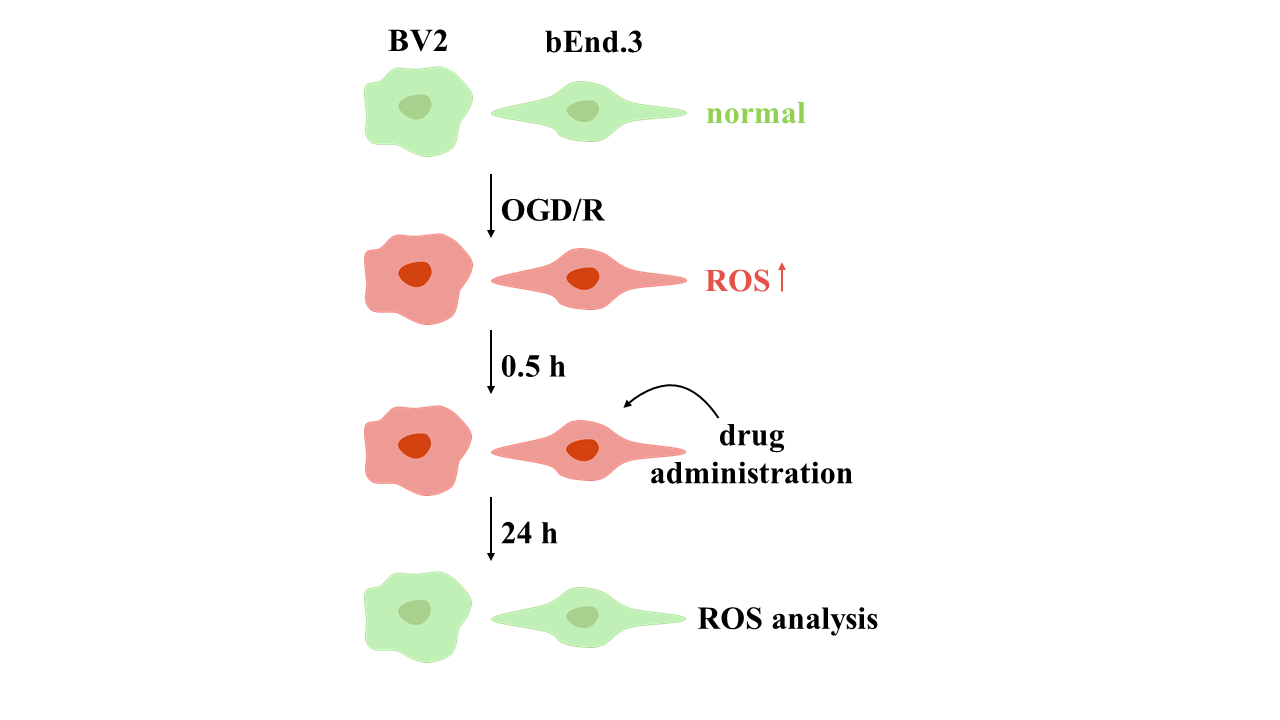

Supplement: Supplementary material — Original Images for Fig 3_Fig 4.zip [file IDRD_A_2585599_SM5405.zip › Original Image for Fig 3A.tif]

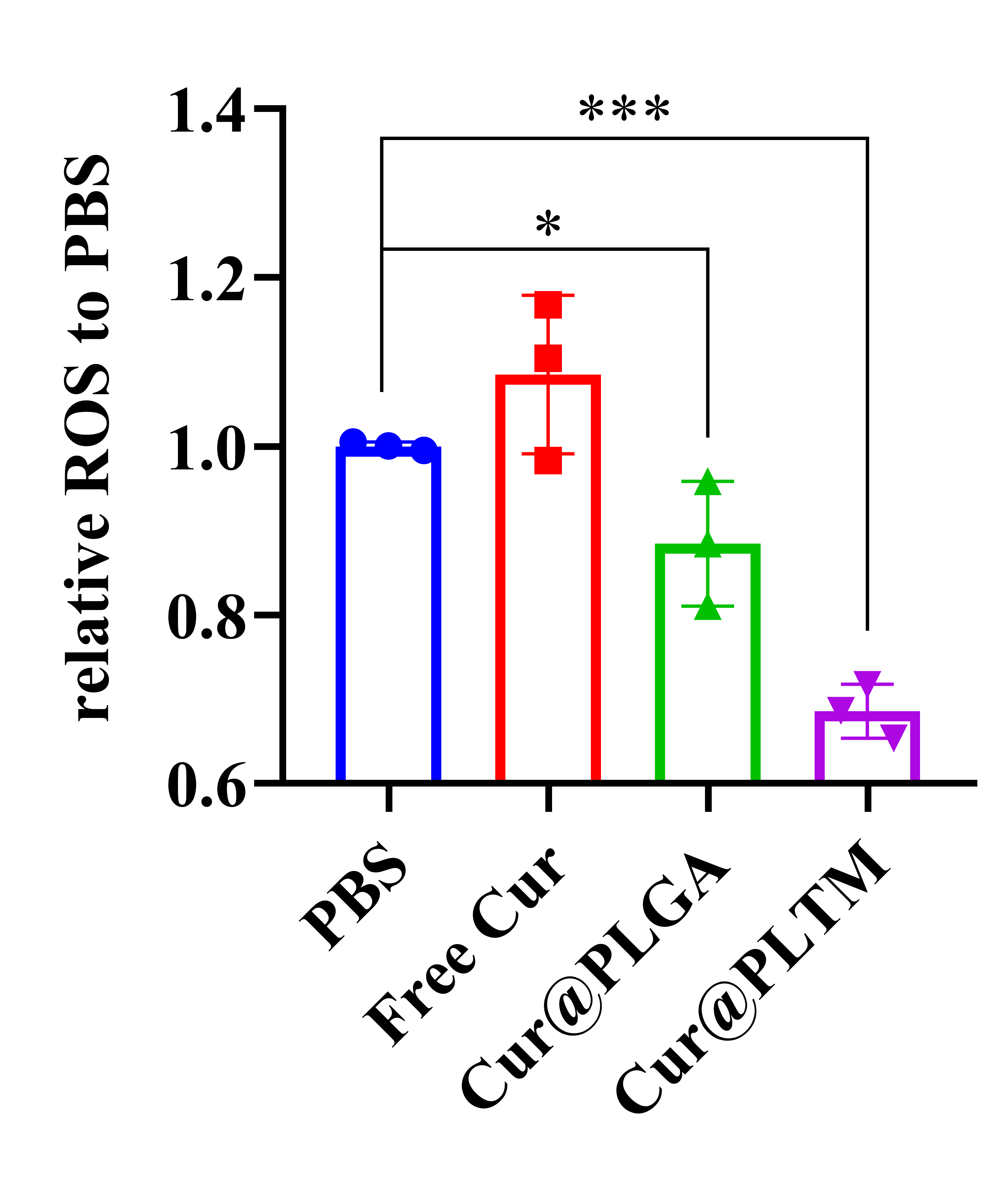

Supplement: Supplementary material — Original Images for Fig 3_Fig 4.zip [file IDRD_A_2585599_SM5405.zip › Original Image for Fig 3B.tif]

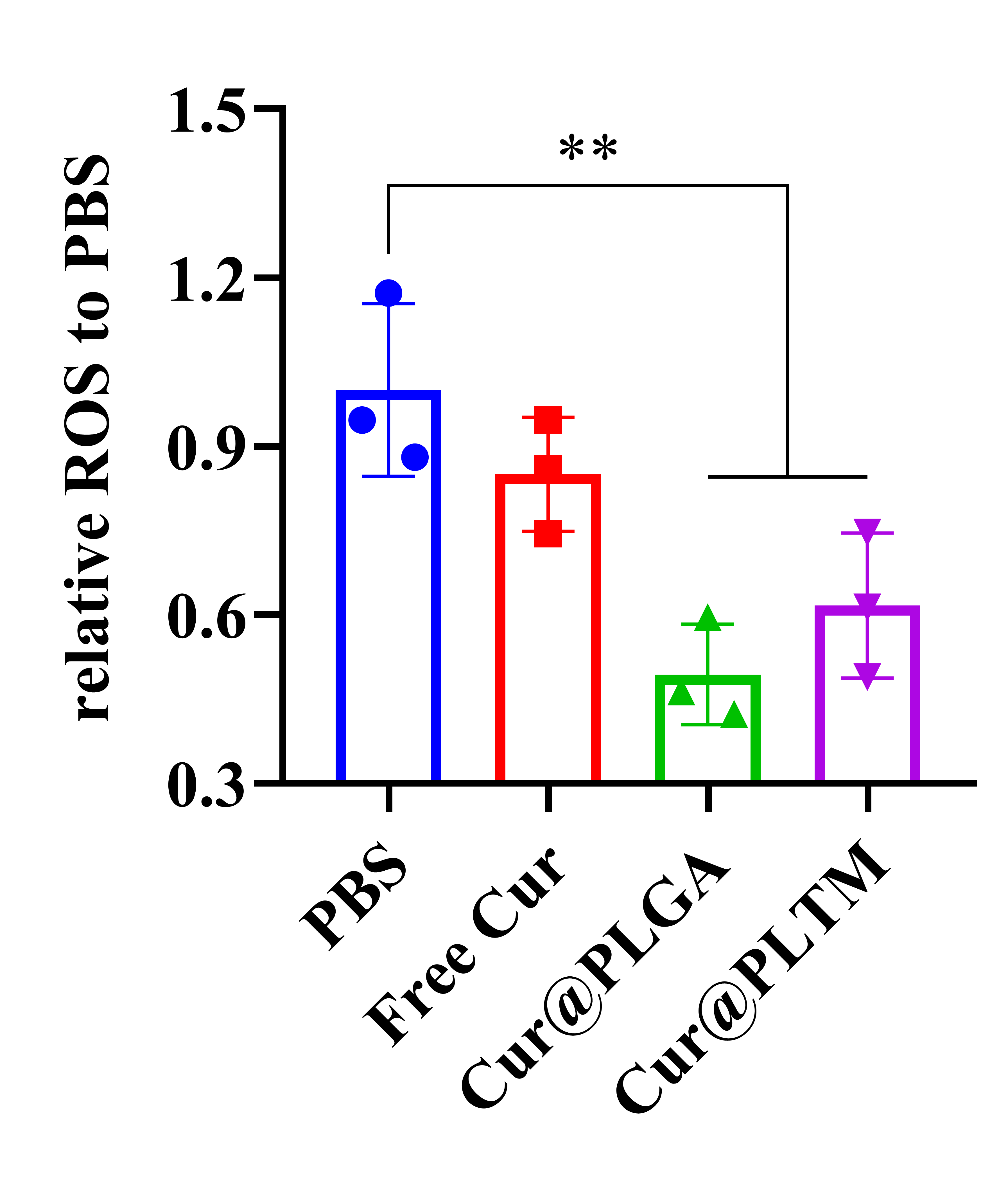

Supplement: Supplementary material — Original Images for Fig 3_Fig 4.zip [file IDRD_A_2585599_SM5405.zip › Original Image for Fig 3C.tif]

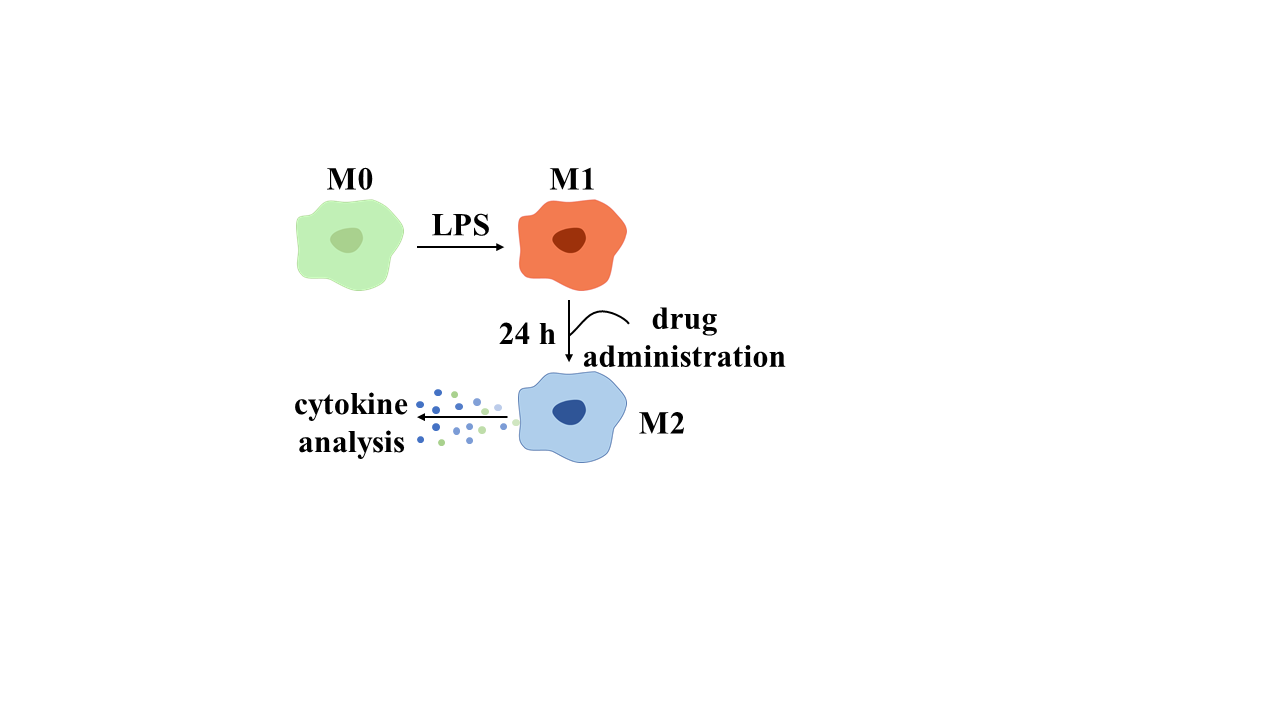

Supplement: Supplementary material — Original Images for Fig 3_Fig 4.zip [file IDRD_A_2585599_SM5405.zip › Original Image for Fig 3D.tif]

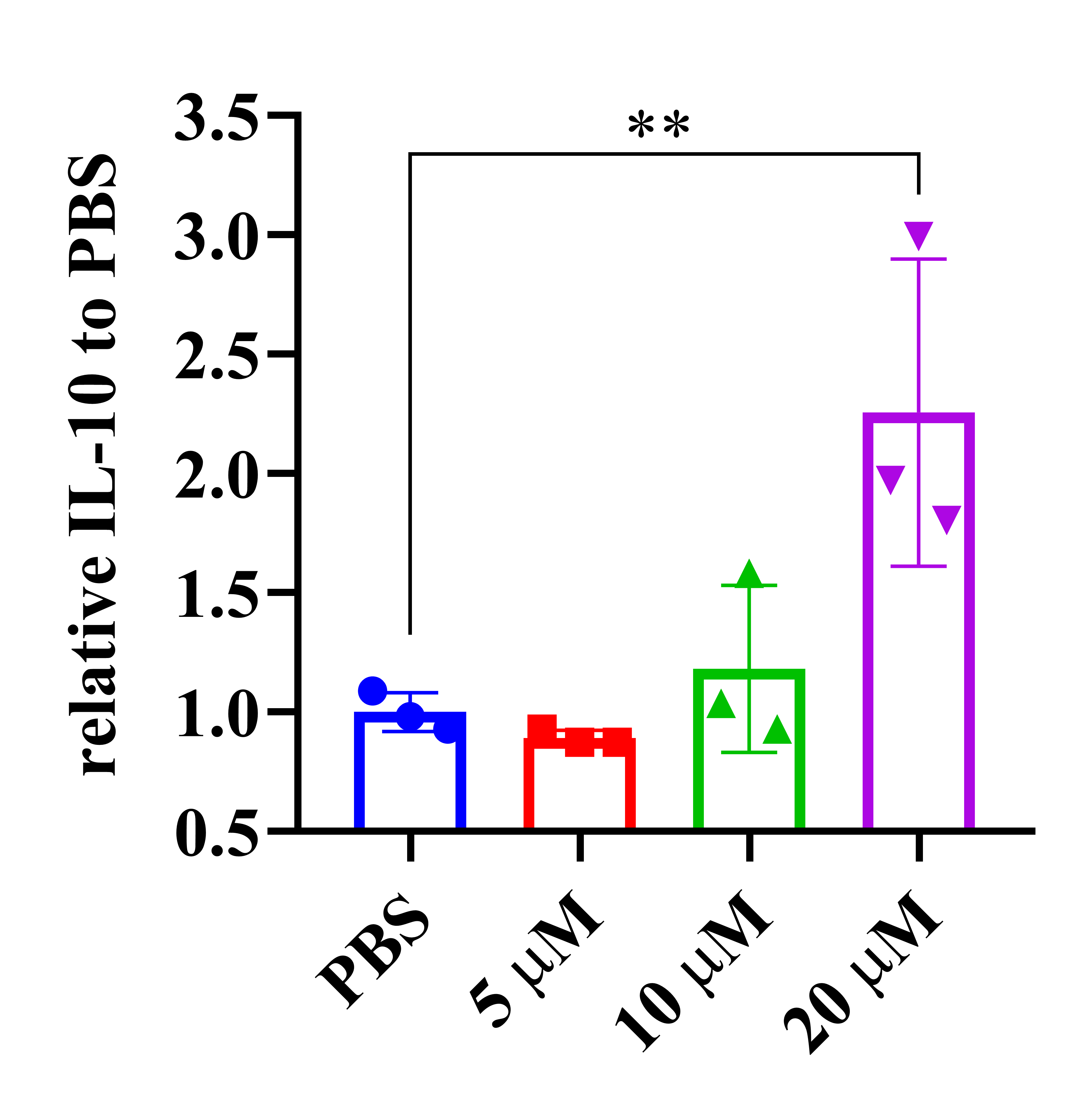

Supplement: Supplementary material — Original Images for Fig 3_Fig 4.zip [file IDRD_A_2585599_SM5405.zip › Original Image for Fig 3E (IL-10).tif]

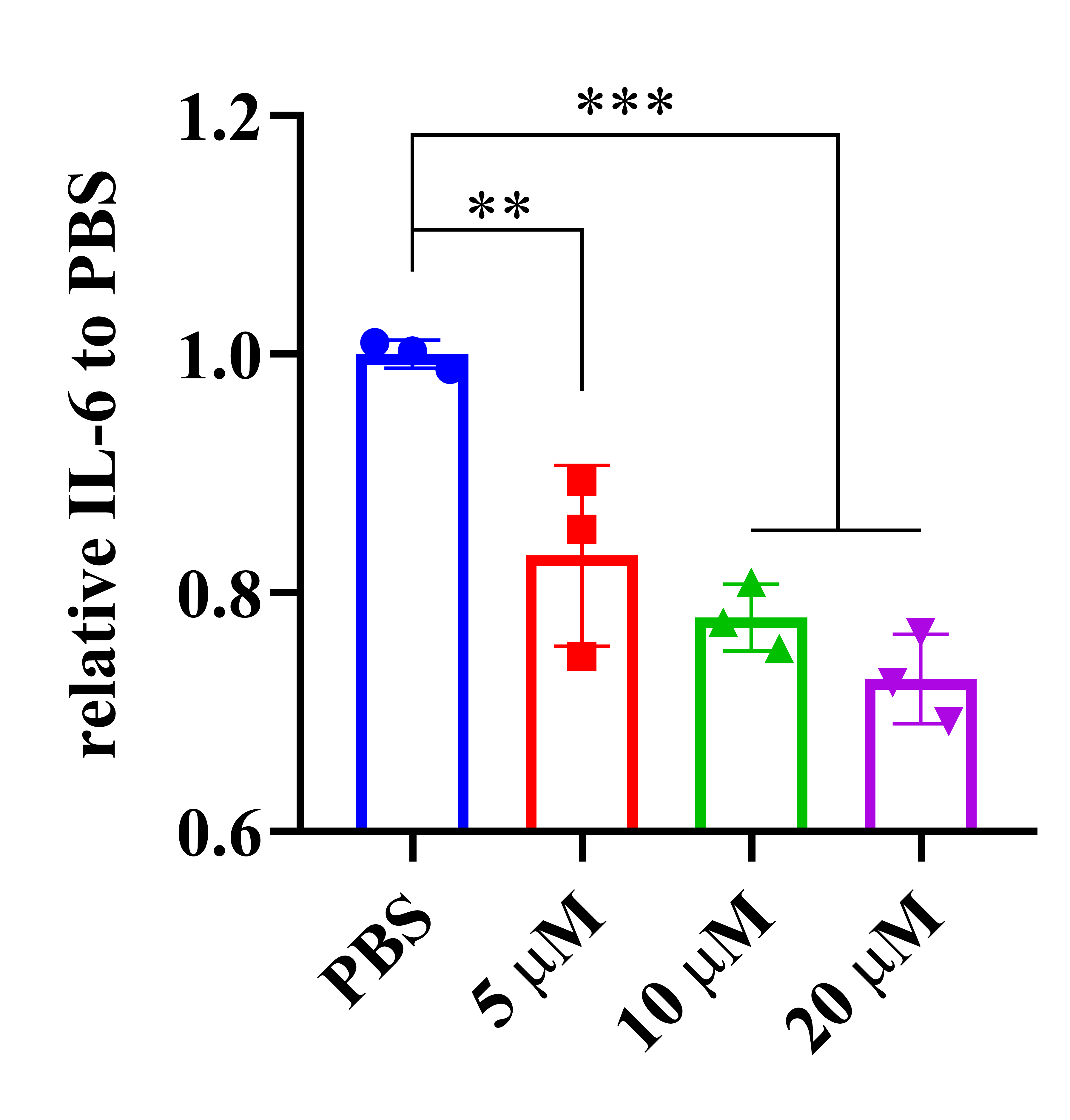

Supplement: Supplementary material — Original Images for Fig 3_Fig 4.zip [file IDRD_A_2585599_SM5405.zip › Original Image for Fig 3E (IL-6).tif]

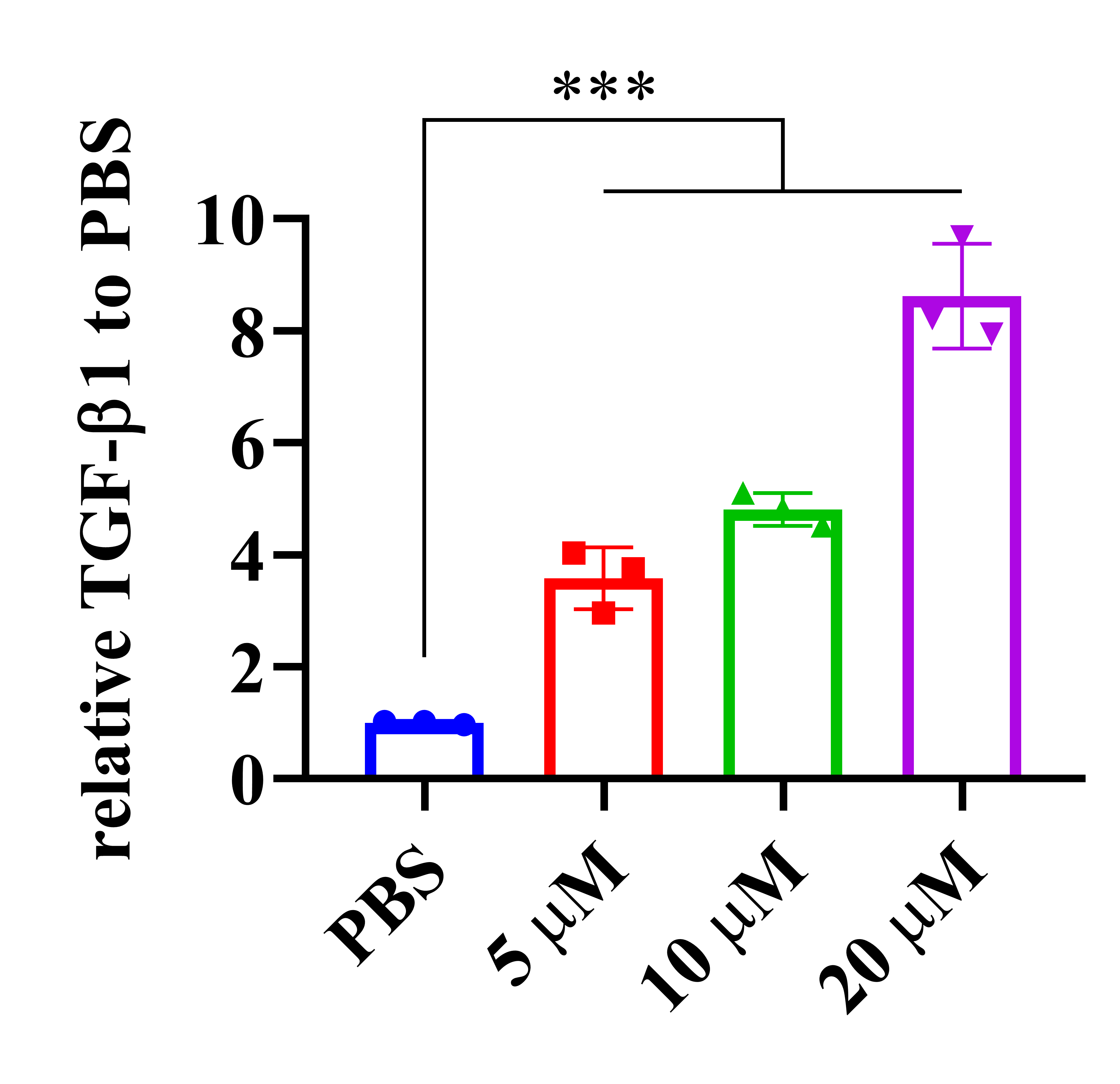

Supplement: Supplementary material — Original Images for Fig 3_Fig 4.zip [file IDRD_A_2585599_SM5405.zip › Original Image for Fig 3E (TGF-β1).tif]

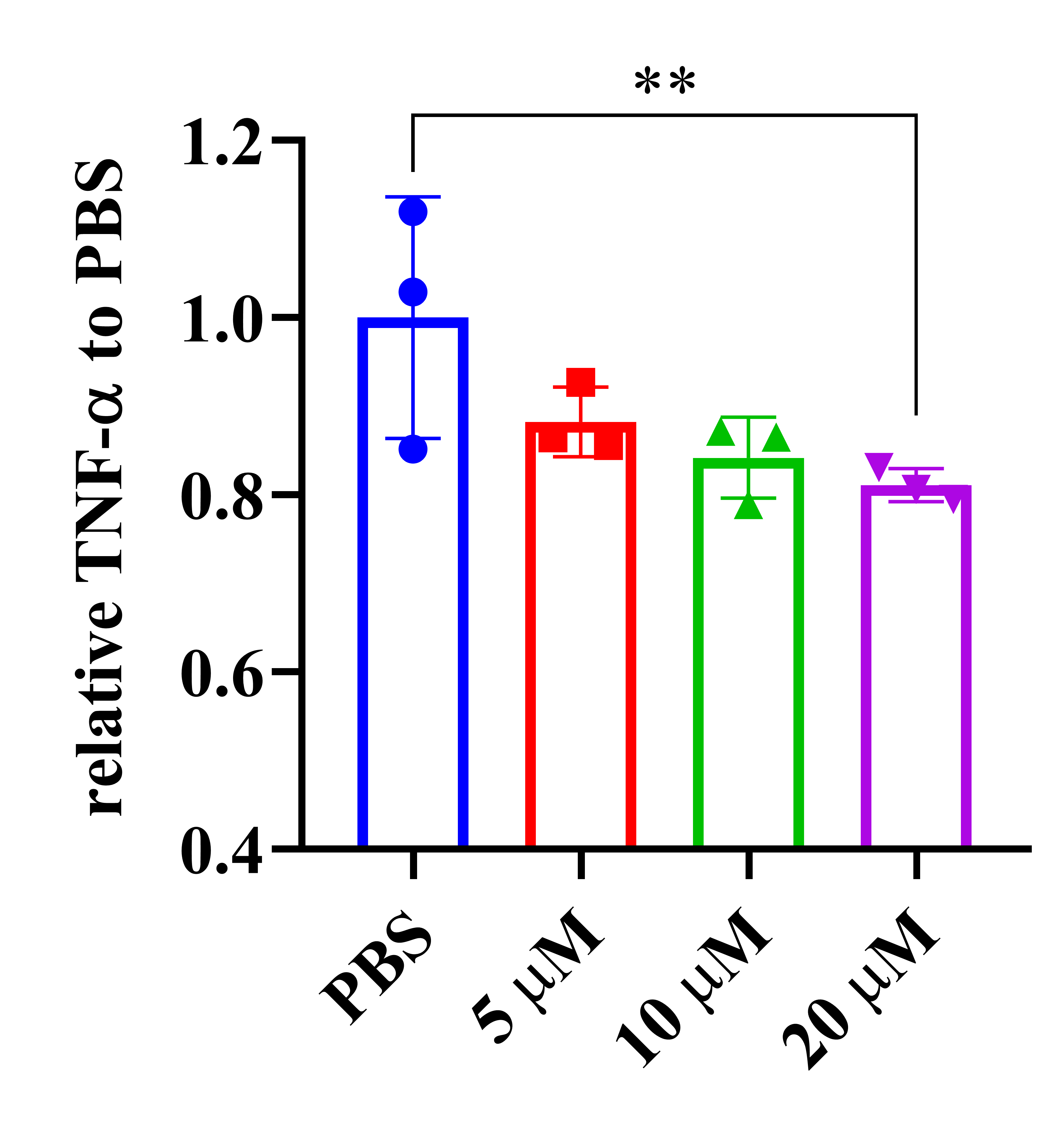

Supplement: Supplementary material — Original Images for Fig 3_Fig 4.zip [file IDRD_A_2585599_SM5405.zip › Original Image for Fig 3E (TNF-α).tif]

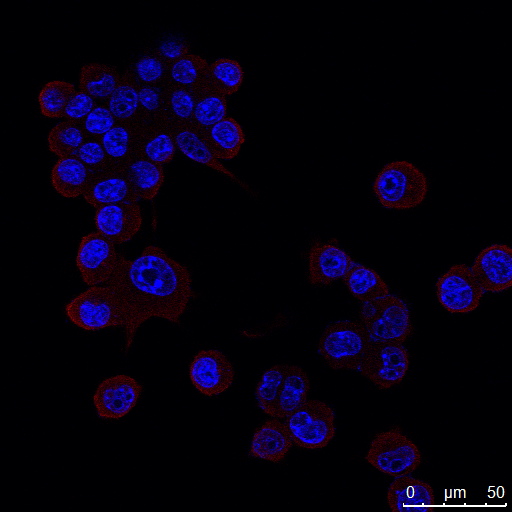

Supplement: Supplementary material — Original Images for Fig 3_Fig 4.zip [file IDRD_A_2585599_SM5405.zip › Original Image for Fig 3F (IL-4).tif]

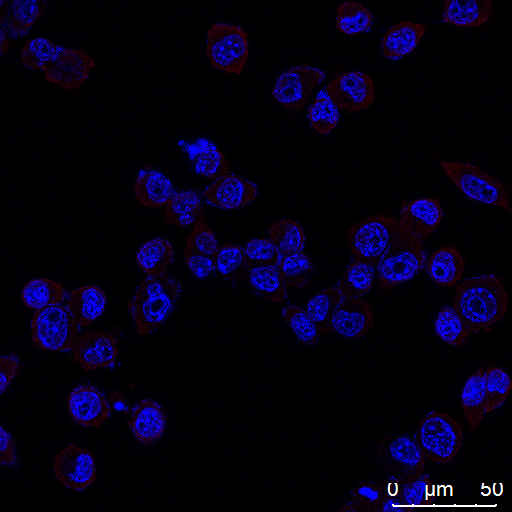

Supplement: Supplementary material — Original Images for Fig 3_Fig 4.zip [file IDRD_A_2585599_SM5405.zip › Original Image for Fig 3F (PBS).tif]

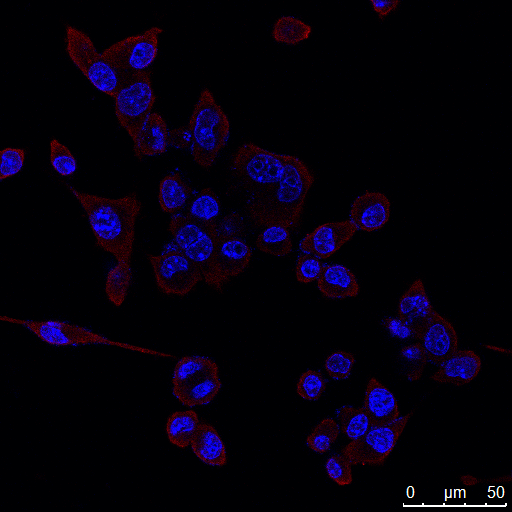

Supplement: Supplementary material — Original Images for Fig 3_Fig 4.zip [file IDRD_A_2585599_SM5405.zip › Original Image for Fig 3F (Tid@PLTM).tif]

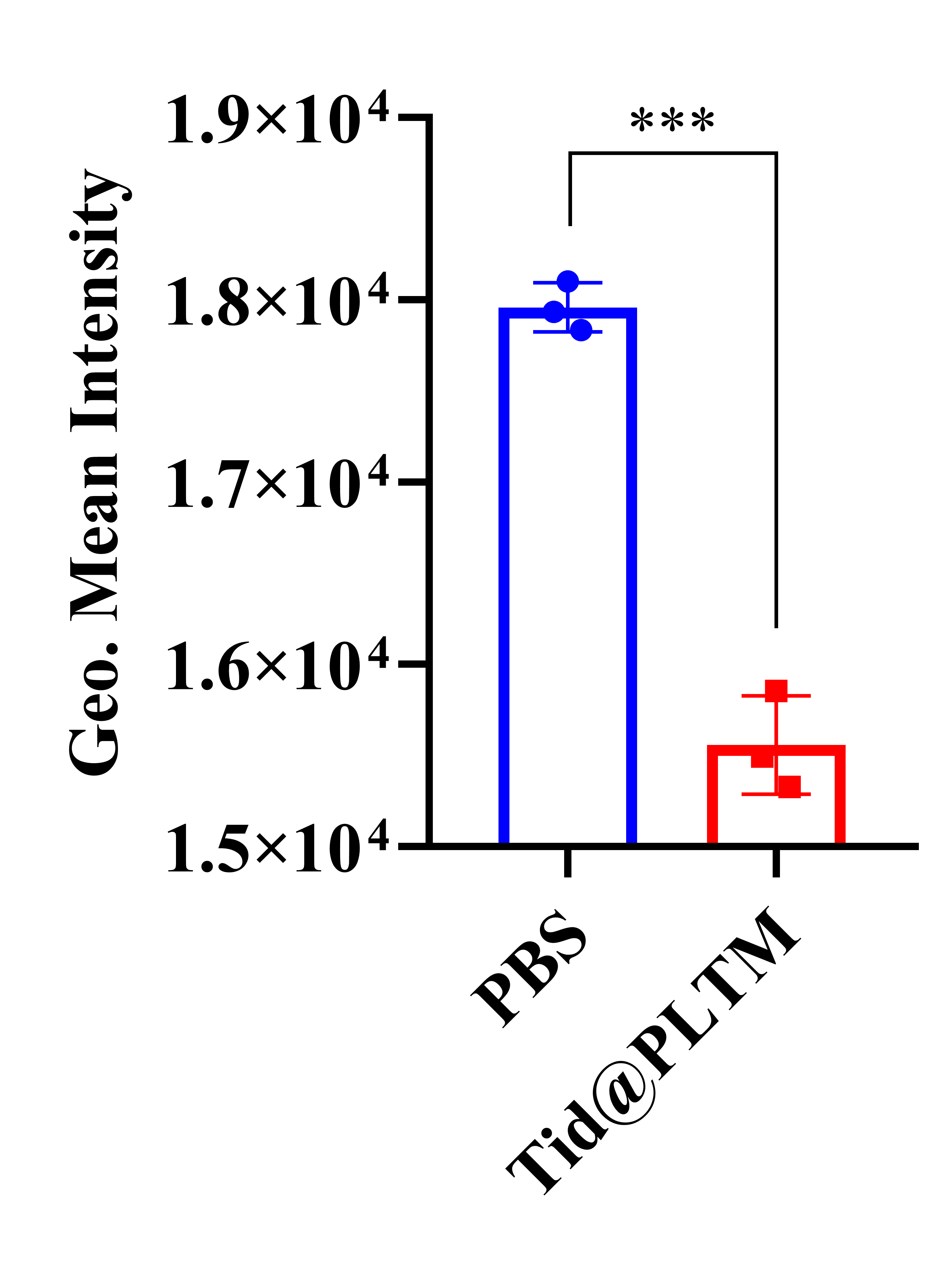

Supplement: Supplementary material — Original Images for Fig 3_Fig 4.zip [file IDRD_A_2585599_SM5405.zip › Original Image for Fig 3G.tif]

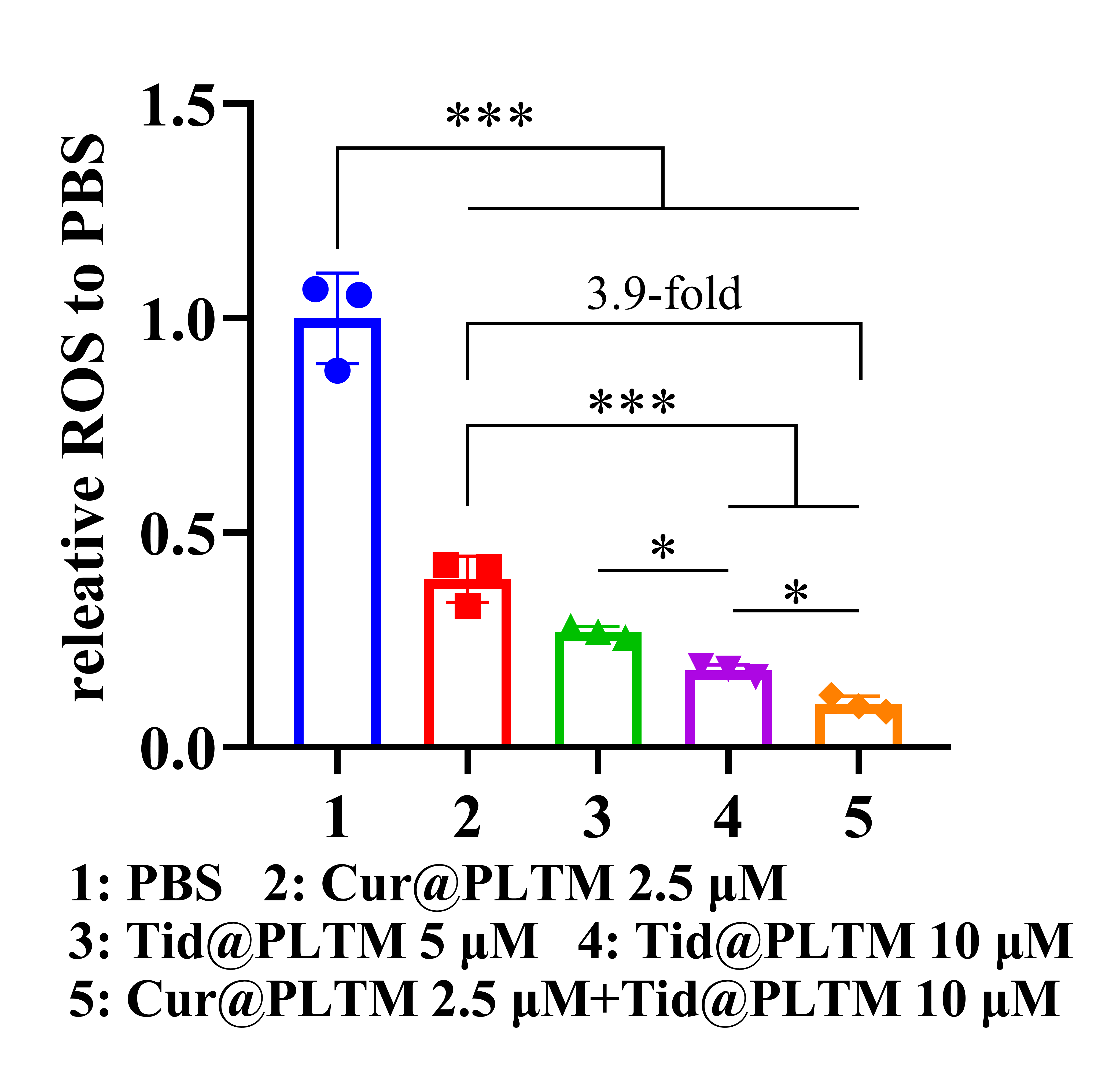

Supplement: Supplementary material — Original Images for Fig 3_Fig 4.zip [file IDRD_A_2585599_SM5405.zip › Original Image for Fig 3H.tif]

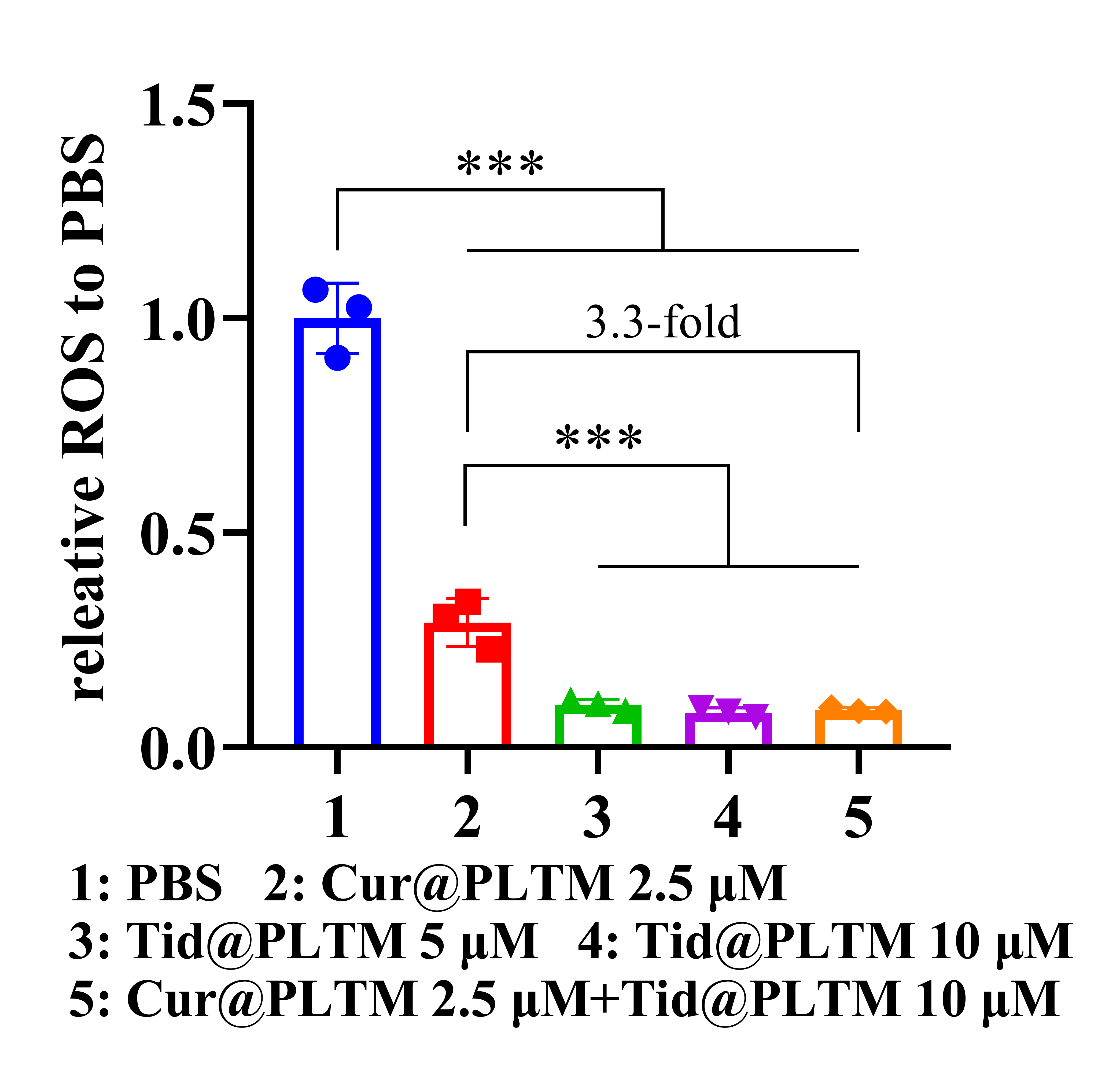

Supplement: Supplementary material — Original Images for Fig 3_Fig 4.zip [file IDRD_A_2585599_SM5405.zip › Original Image for Fig 3I.tif]

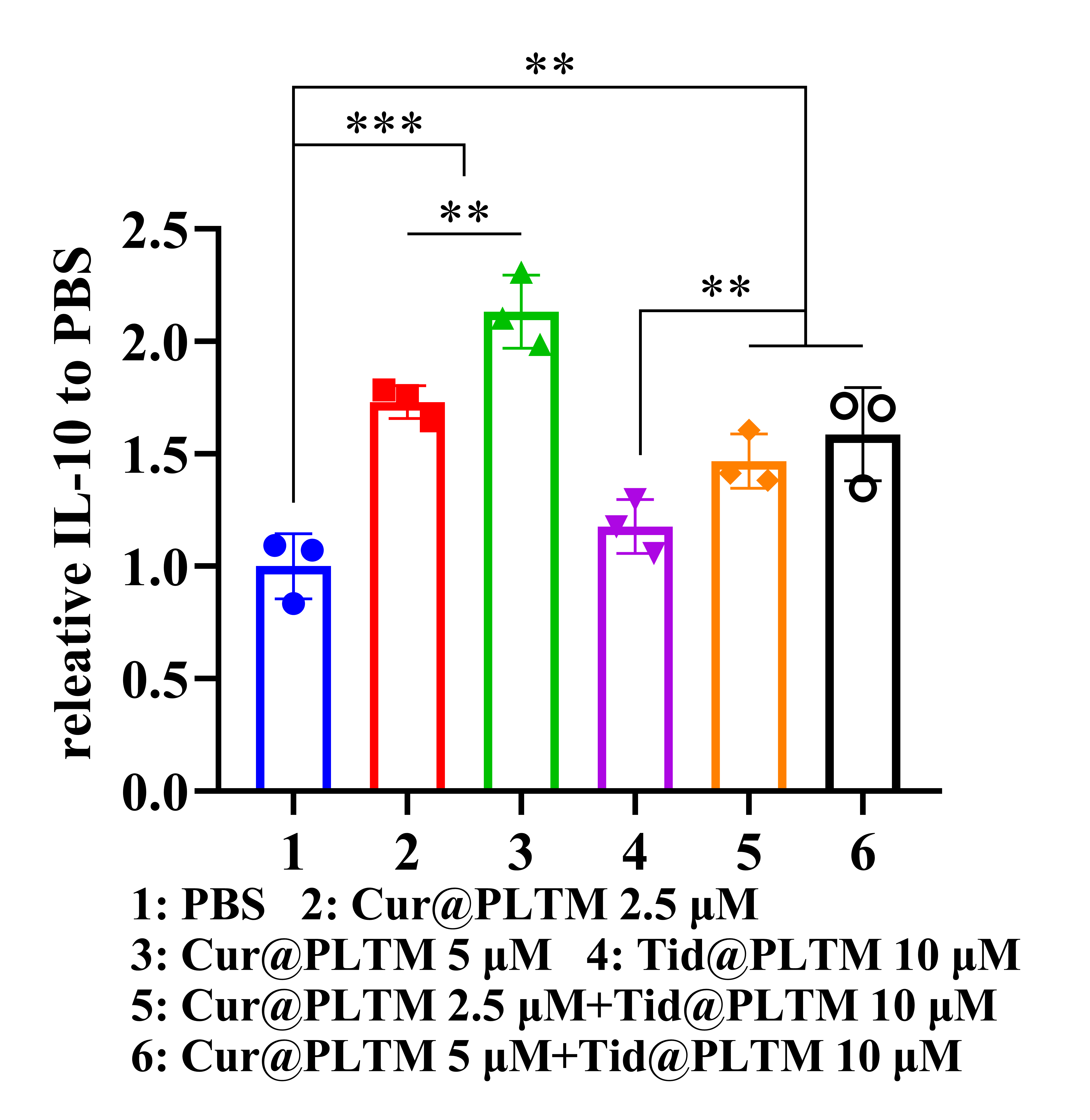

Supplement: Supplementary material — Original Images for Fig 3_Fig 4.zip [file IDRD_A_2585599_SM5405.zip › Original Image for Fig 3J.tif]

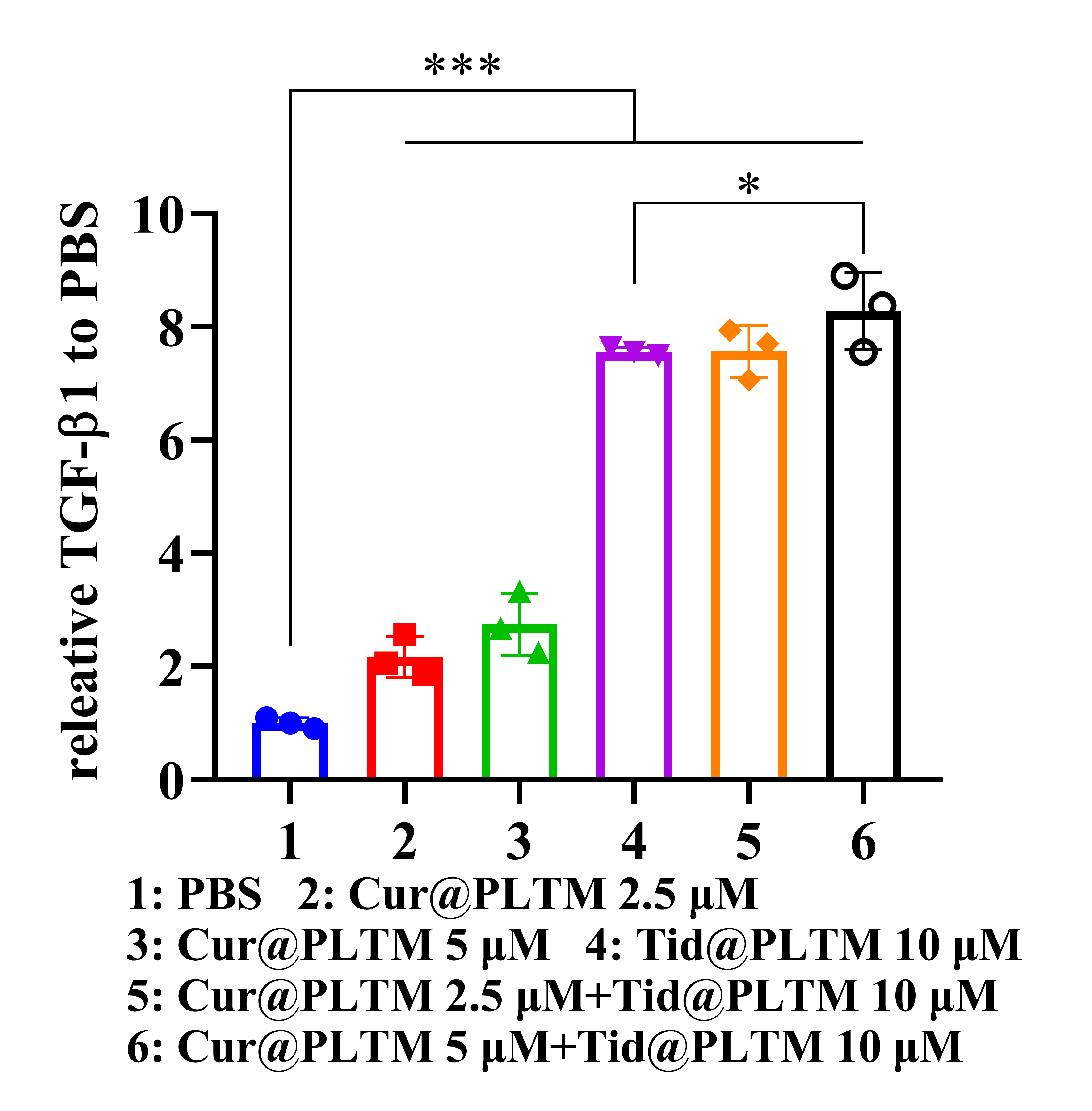

Supplement: Supplementary material — Original Images for Fig 3_Fig 4.zip [file IDRD_A_2585599_SM5405.zip › Original Image for Fig 3K.tif]

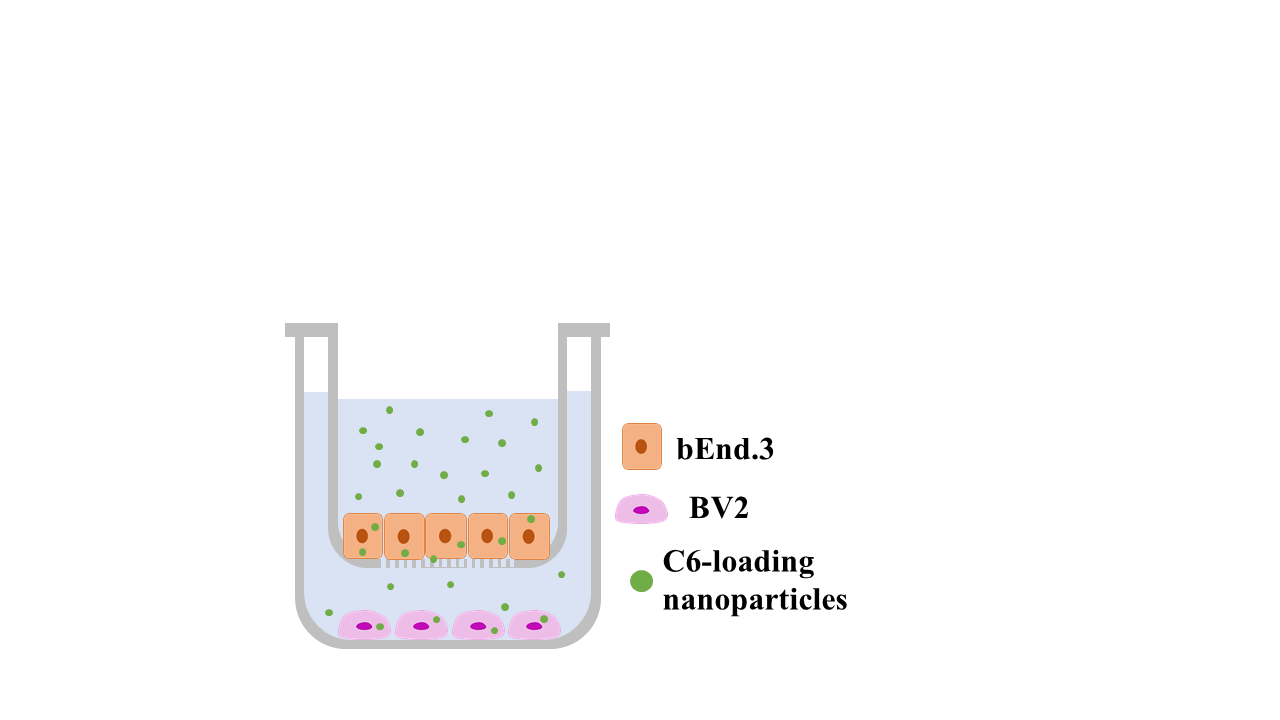

Supplement: Supplementary material — Original Images for Fig 3_Fig 4.zip [file IDRD_A_2585599_SM5405.zip › Original Image for Fig 4A.tif]

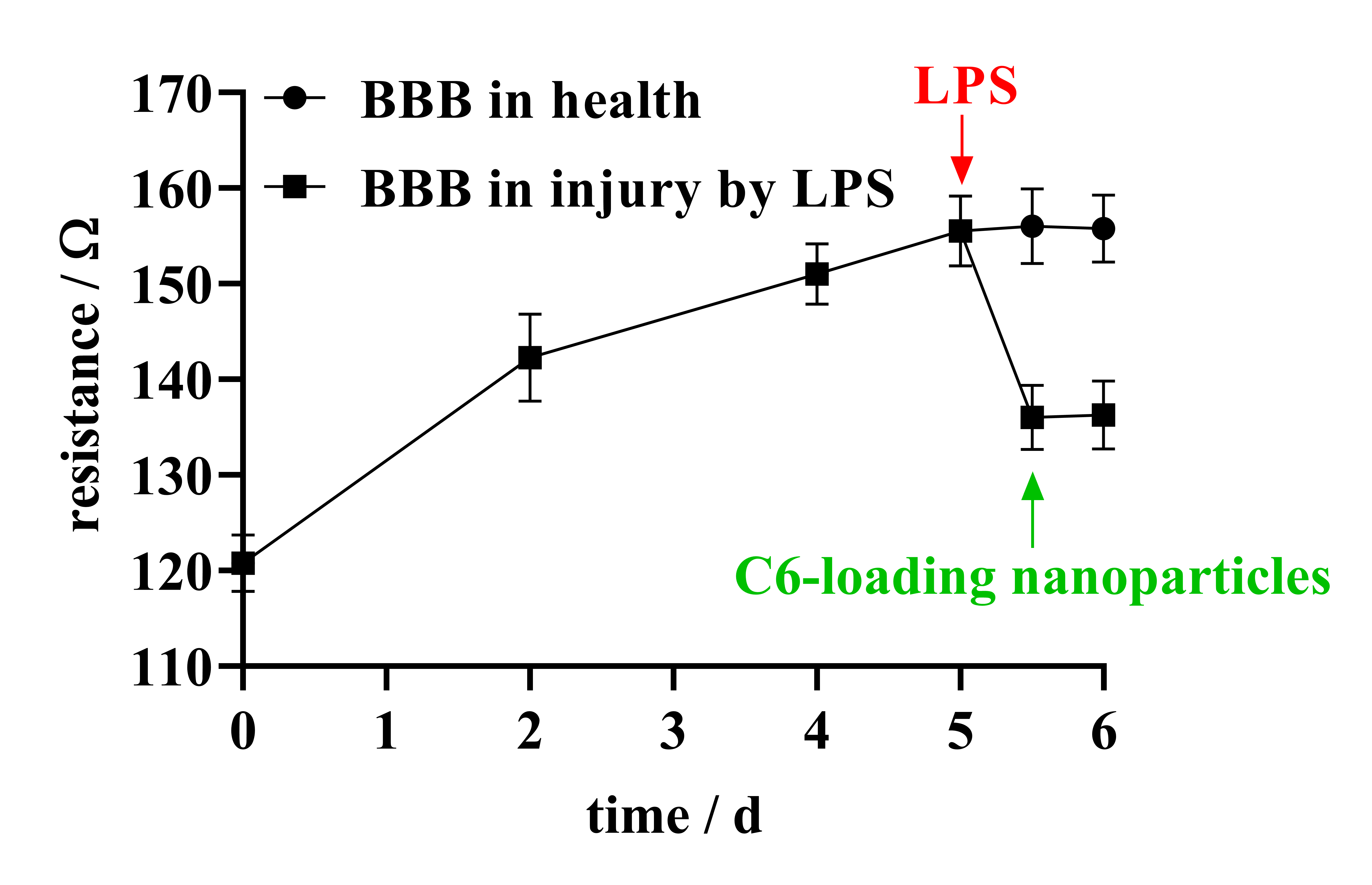

Supplement: Supplementary material — Original Images for Fig 3_Fig 4.zip [file IDRD_A_2585599_SM5405.zip › Original Image for Fig 4B.tif]

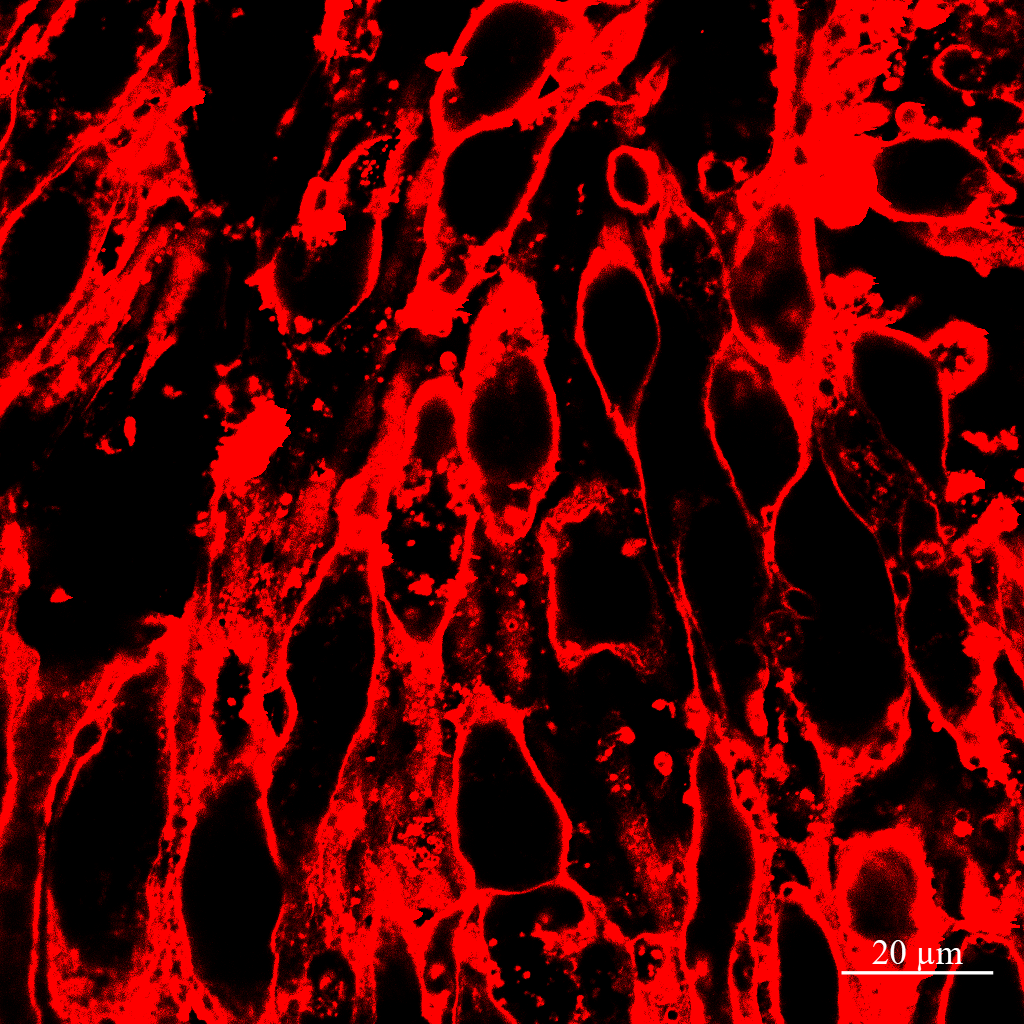

Supplement: Supplementary material — Original Images for Fig 3_Fig 4.zip [file IDRD_A_2585599_SM5405.zip › Original Image for Fig 4C Healthy C6@PLGA (cytomembrane).tif]

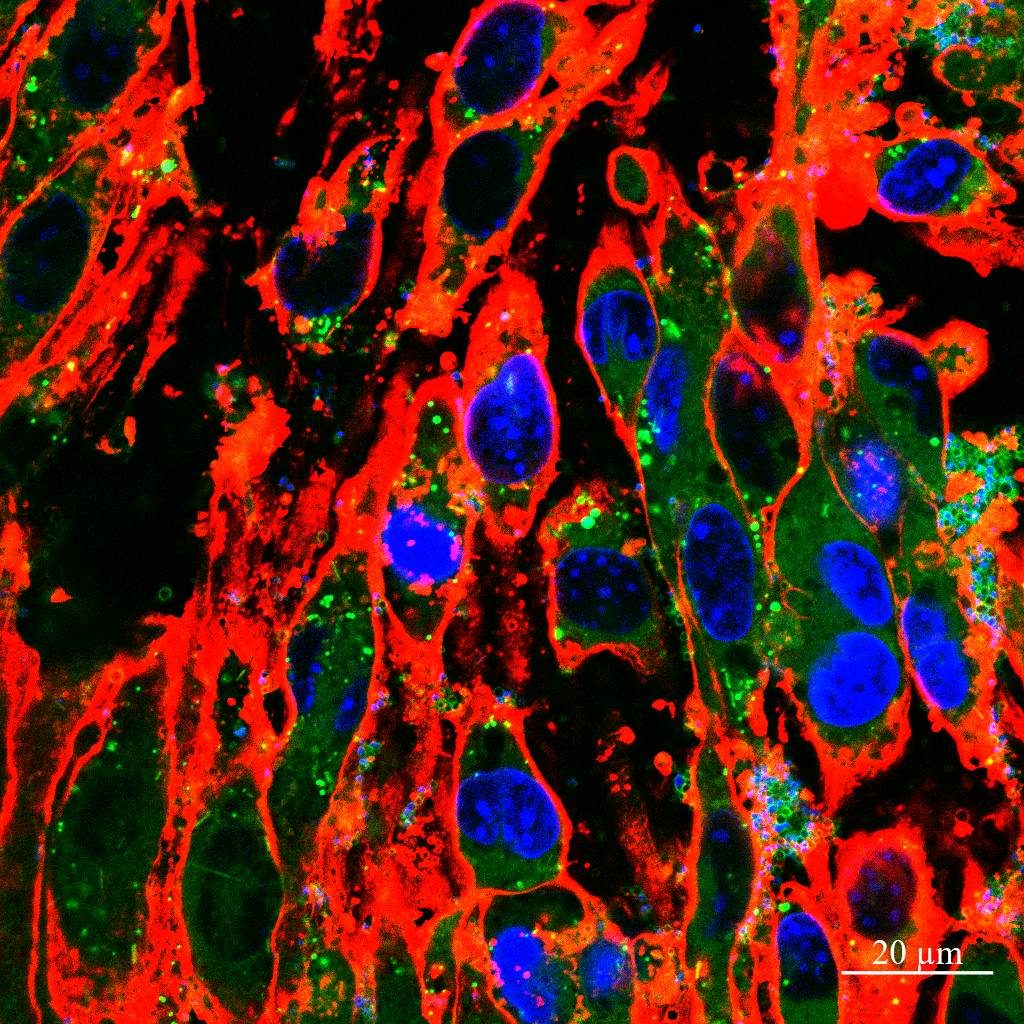

Supplement: Supplementary material — Original Images for Fig 3_Fig 4.zip [file IDRD_A_2585599_SM5405.zip › Original Image for Fig 4C Healthy C6@PLGA (merged).tif]

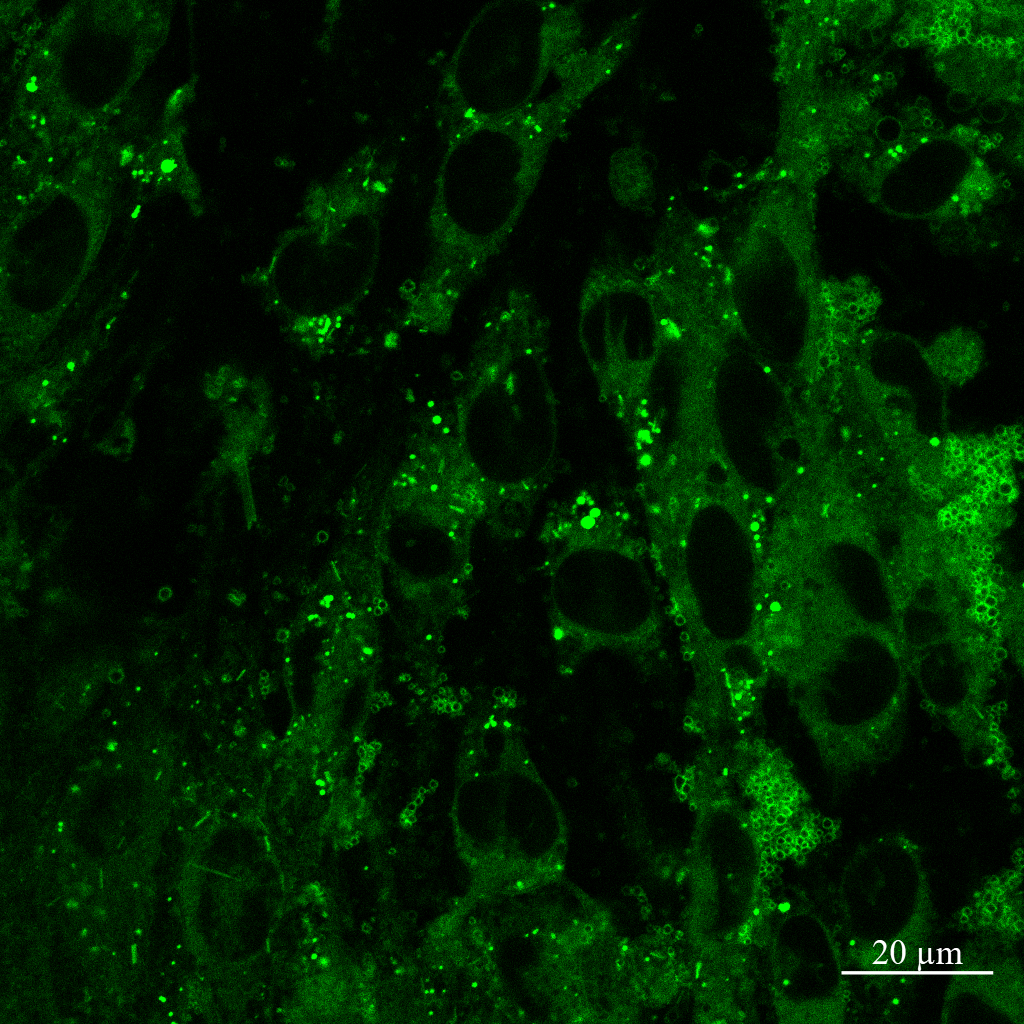

Supplement: Supplementary material — Original Images for Fig 3_Fig 4.zip [file IDRD_A_2585599_SM5405.zip › Original Image for Fig 4C Healthy C6@PLGA (nanoparticles).tif]

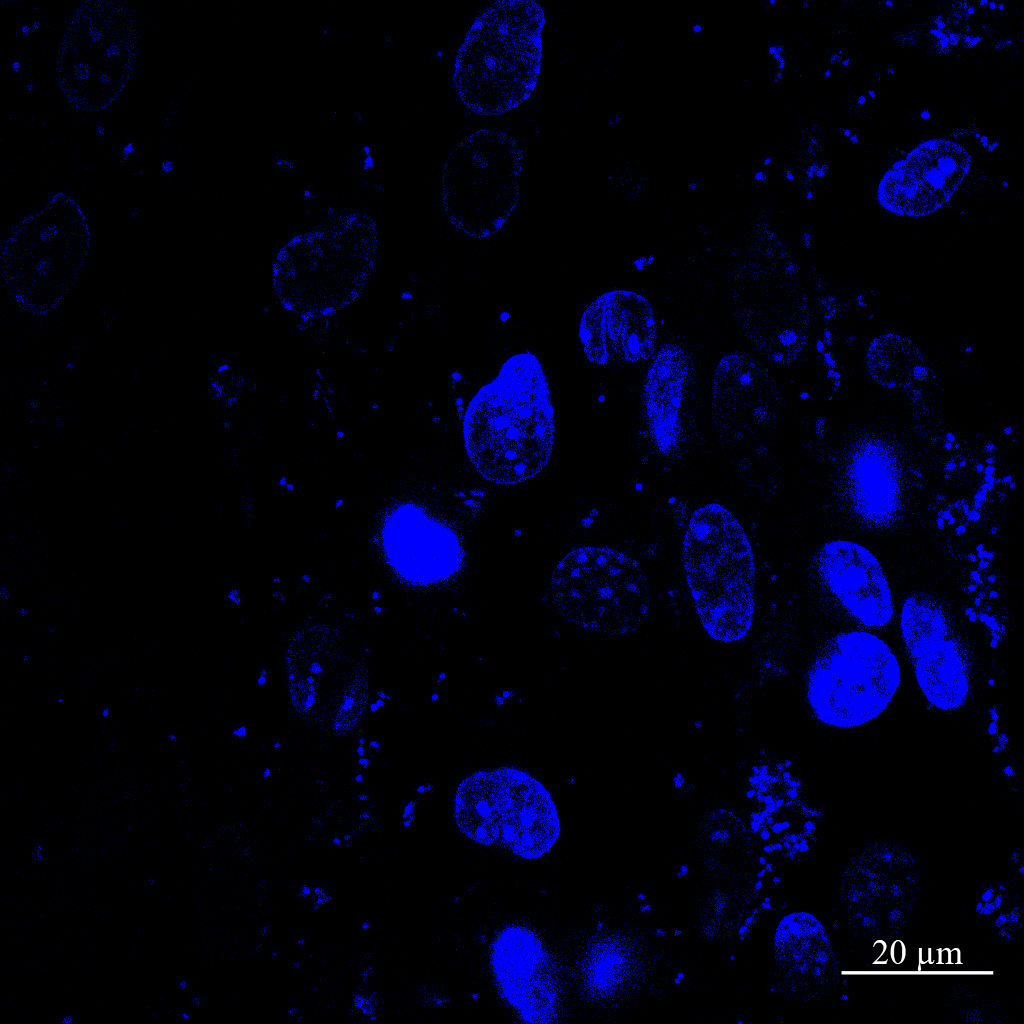

Supplement: Supplementary material — Original Images for Fig 3_Fig 4.zip [file IDRD_A_2585599_SM5405.zip › Original Image for Fig 4C Healthy C6@PLGA (nucleus).tif]

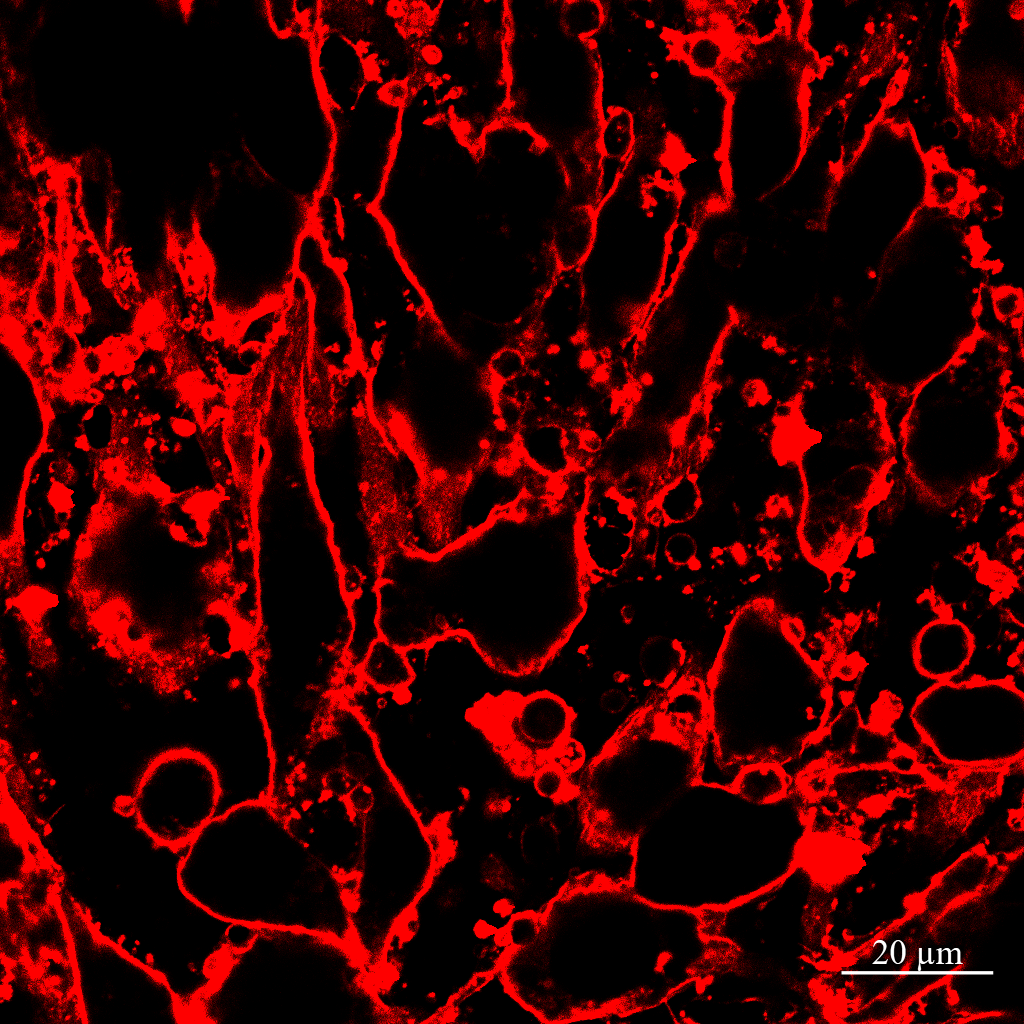

Supplement: Supplementary material — Original Images for Fig 3_Fig 4.zip [file IDRD_A_2585599_SM5405.zip › Original Image for Fig 4C Healthy C6@PLTM (cytomembrane).tif]

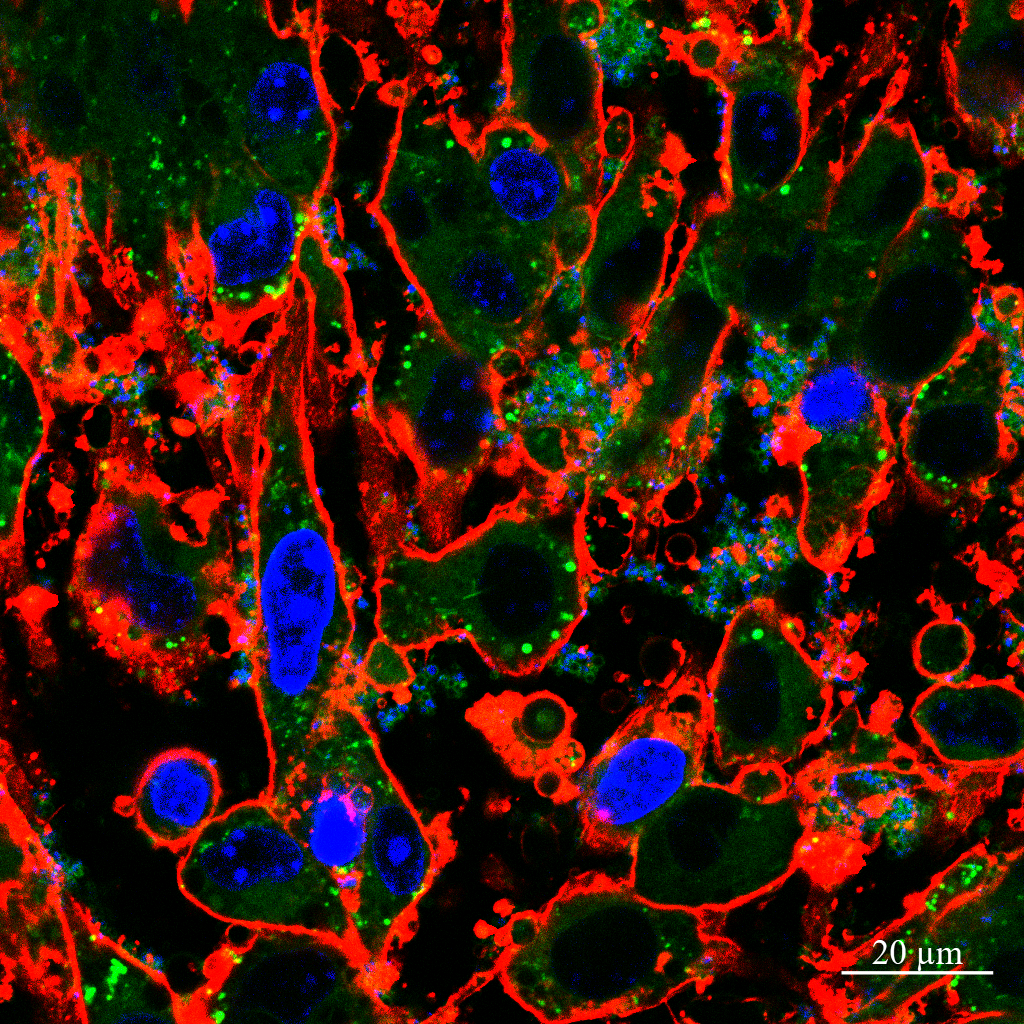

Supplement: Supplementary material — Original Images for Fig 3_Fig 4.zip [file IDRD_A_2585599_SM5405.zip › Original Image for Fig 4C Healthy C6@PLTM (merged).tif]

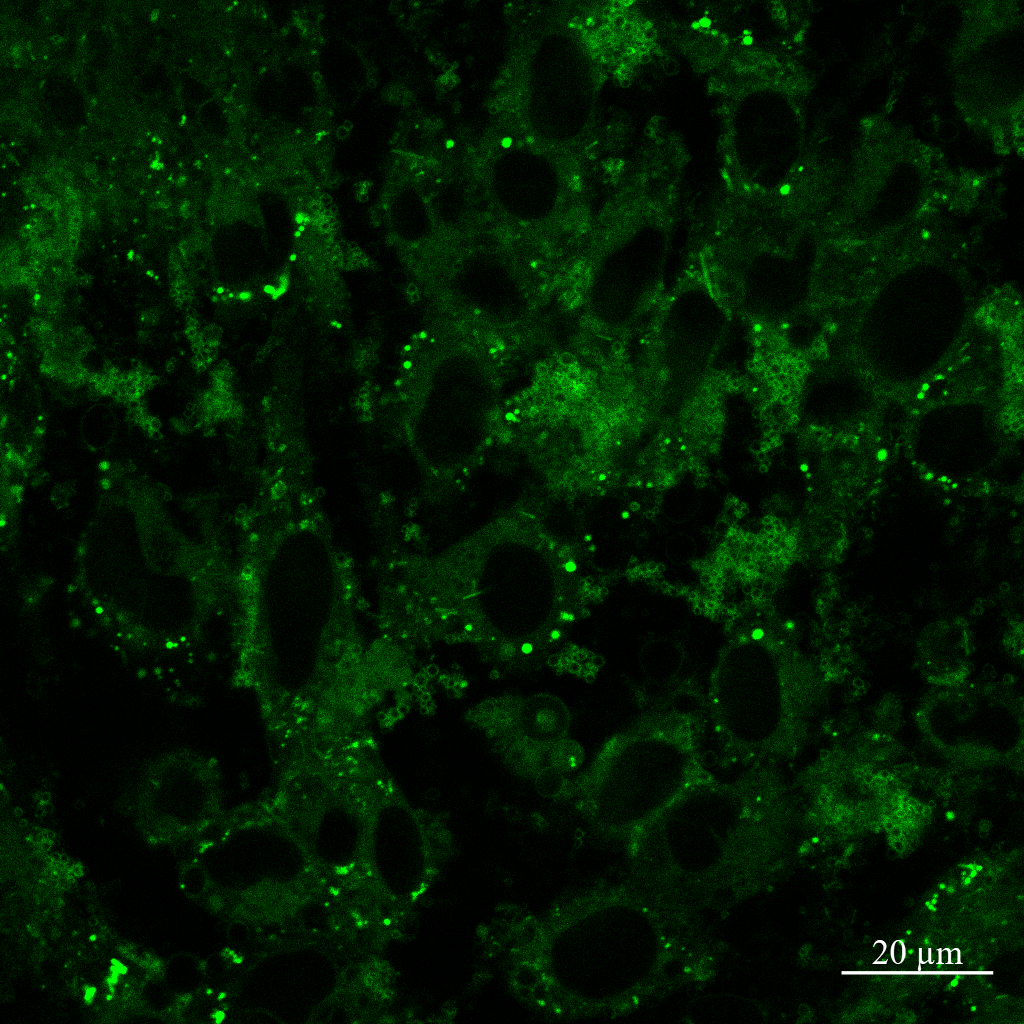

Supplement: Supplementary material — Original Images for Fig 3_Fig 4.zip [file IDRD_A_2585599_SM5405.zip › Original Image for Fig 4C Healthy C6@PLTM (nanoparticles).tif]

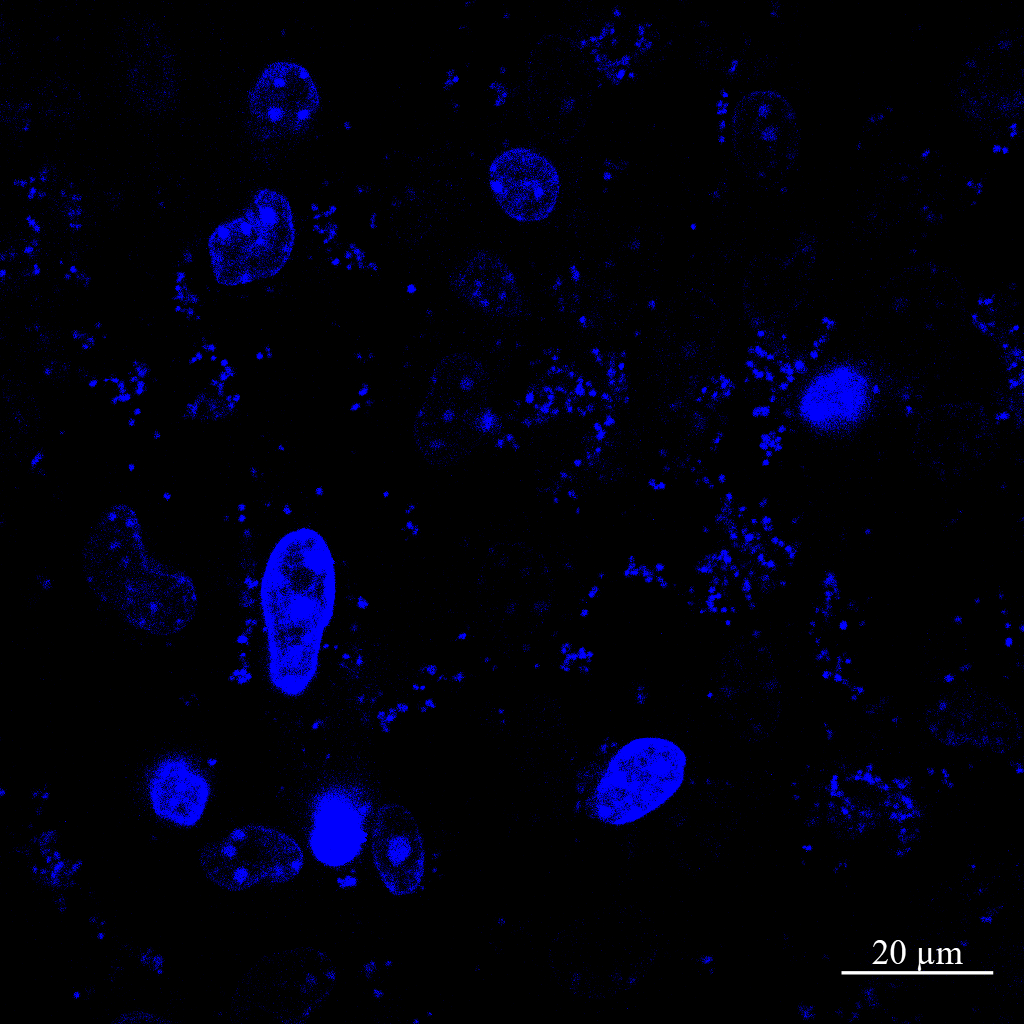

Supplement: Supplementary material — Original Images for Fig 3_Fig 4.zip [file IDRD_A_2585599_SM5405.zip › Original Image for Fig 4C Healthy C6@PLTM (nucleus).tif]

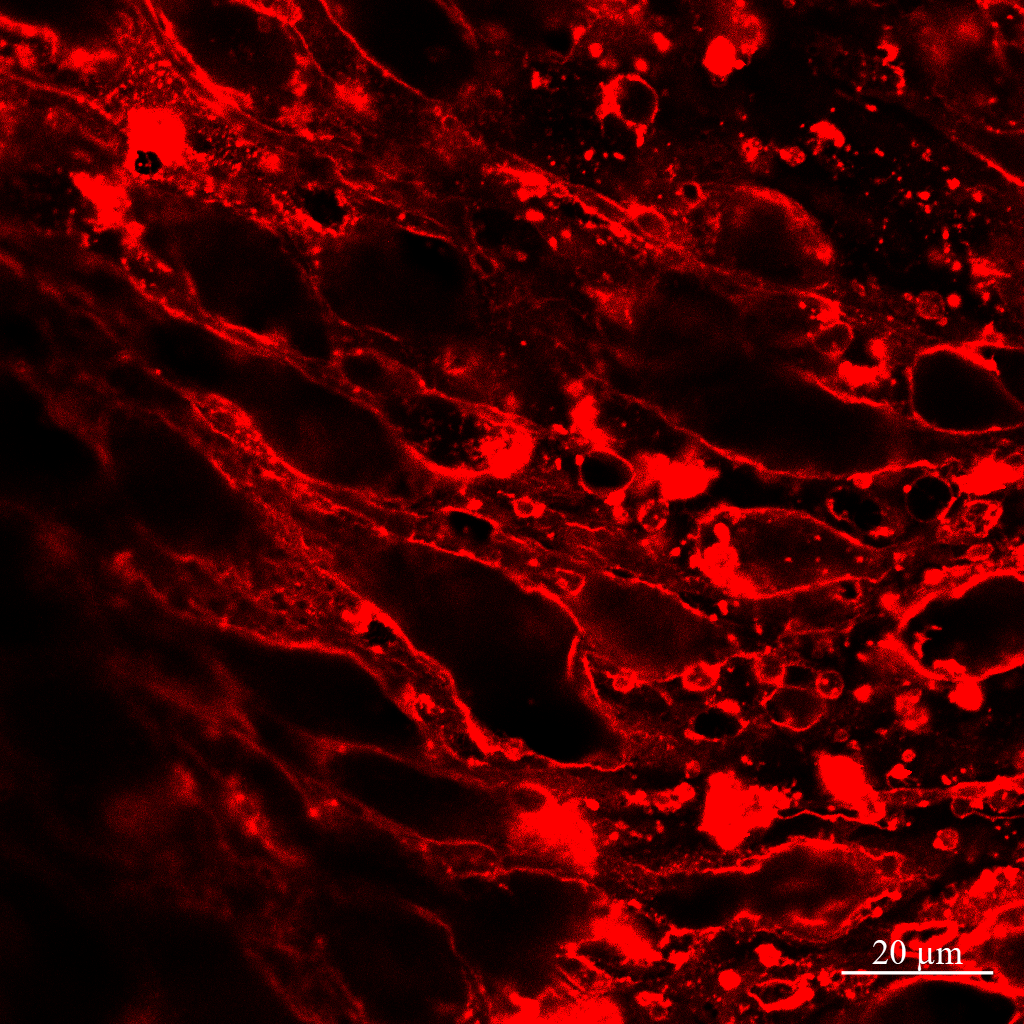

Supplement: Supplementary material — Original Images for Fig 3_Fig 4.zip [file IDRD_A_2585599_SM5405.zip › Original Image for Fig 4C Injured C6@PLGA (cytomembrane).tif]

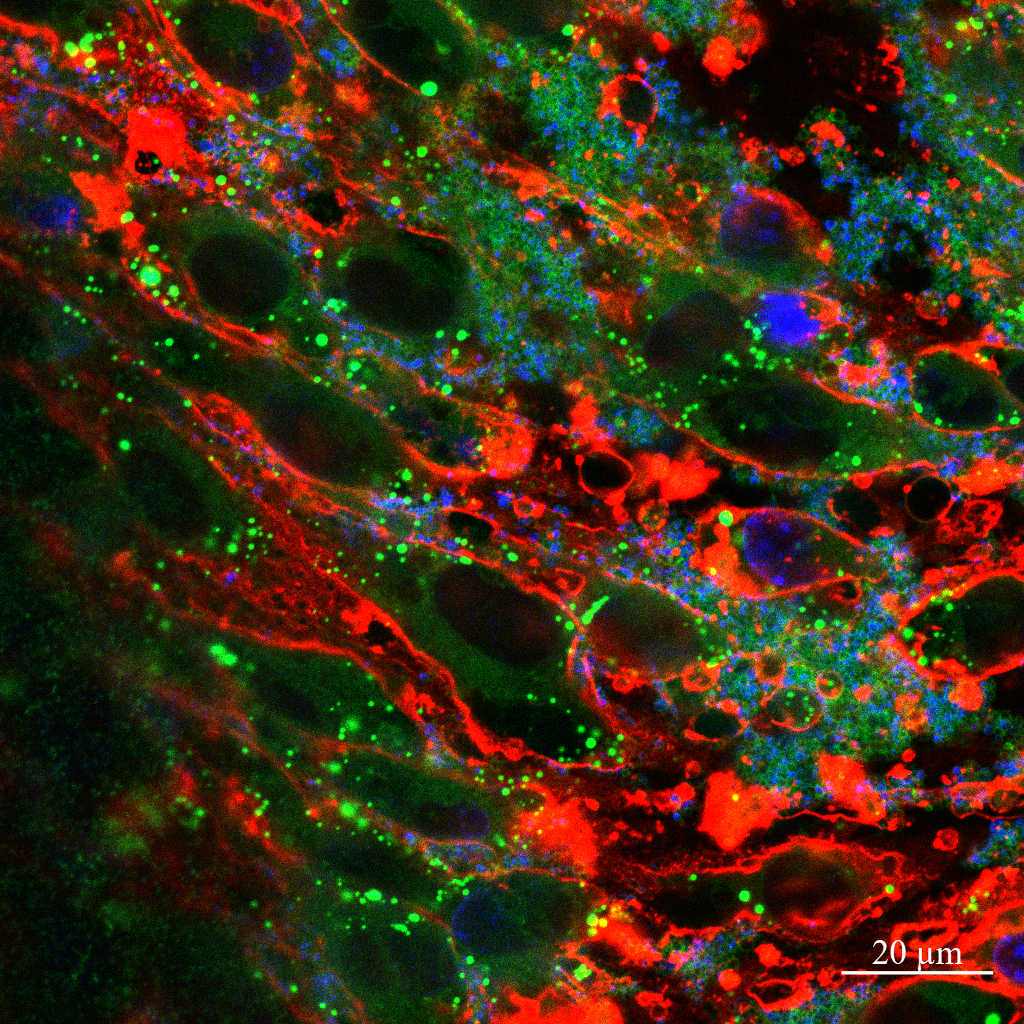

Supplement: Supplementary material — Original Images for Fig 3_Fig 4.zip [file IDRD_A_2585599_SM5405.zip › Original Image for Fig 4C Injured C6@PLGA (merged).tif]

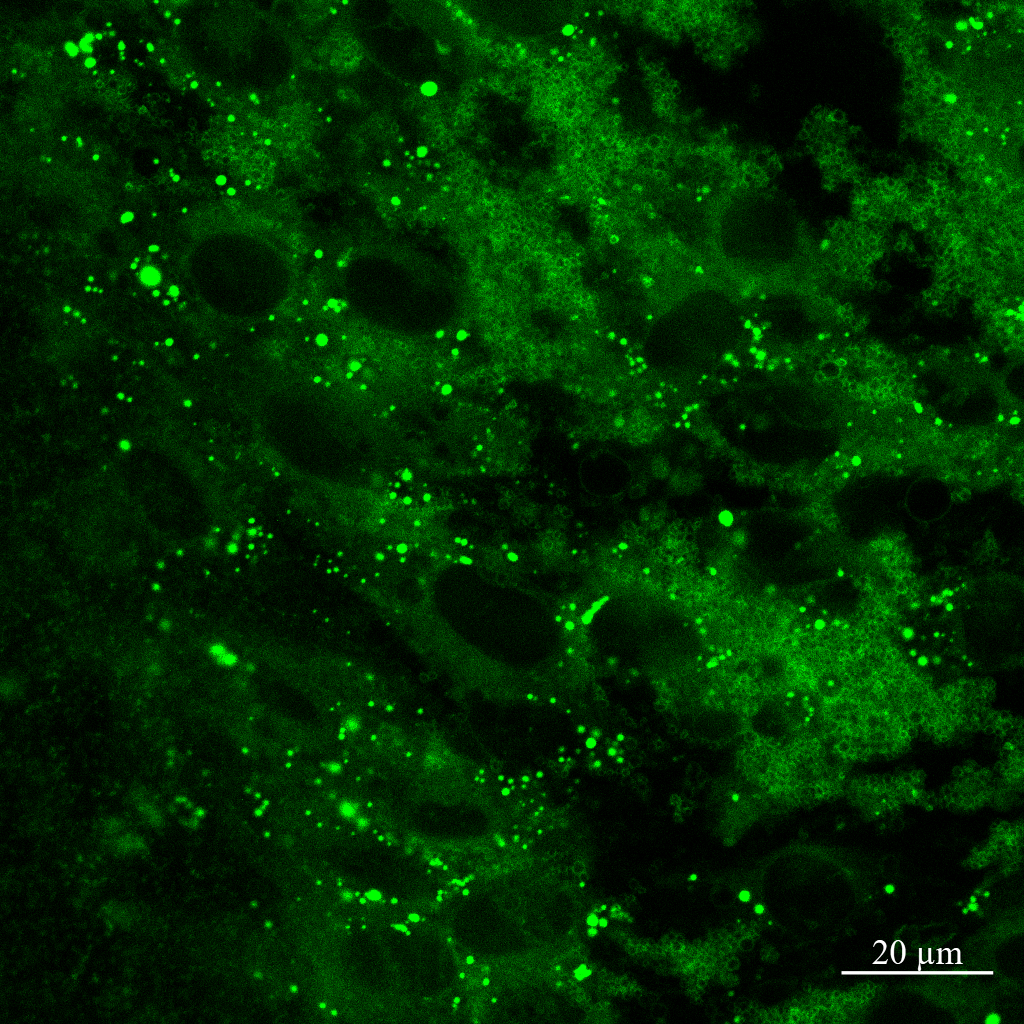

Supplement: Supplementary material — Original Images for Fig 3_Fig 4.zip [file IDRD_A_2585599_SM5405.zip › Original Image for Fig 4C Injured C6@PLGA (nanoparticles).tif]

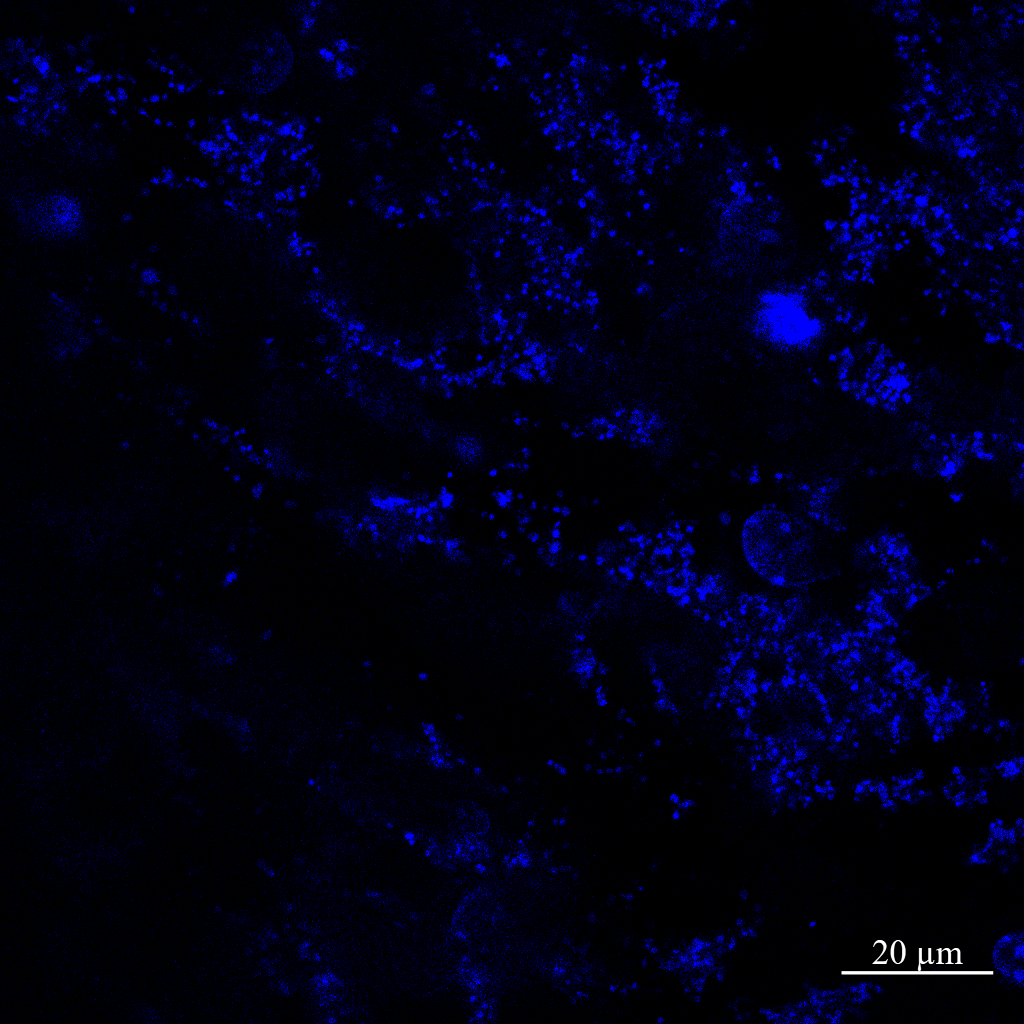

Supplement: Supplementary material — Original Images for Fig 3_Fig 4.zip [file IDRD_A_2585599_SM5405.zip › Original Image for Fig 4C Injured C6@PLGA (nucleus).tif]

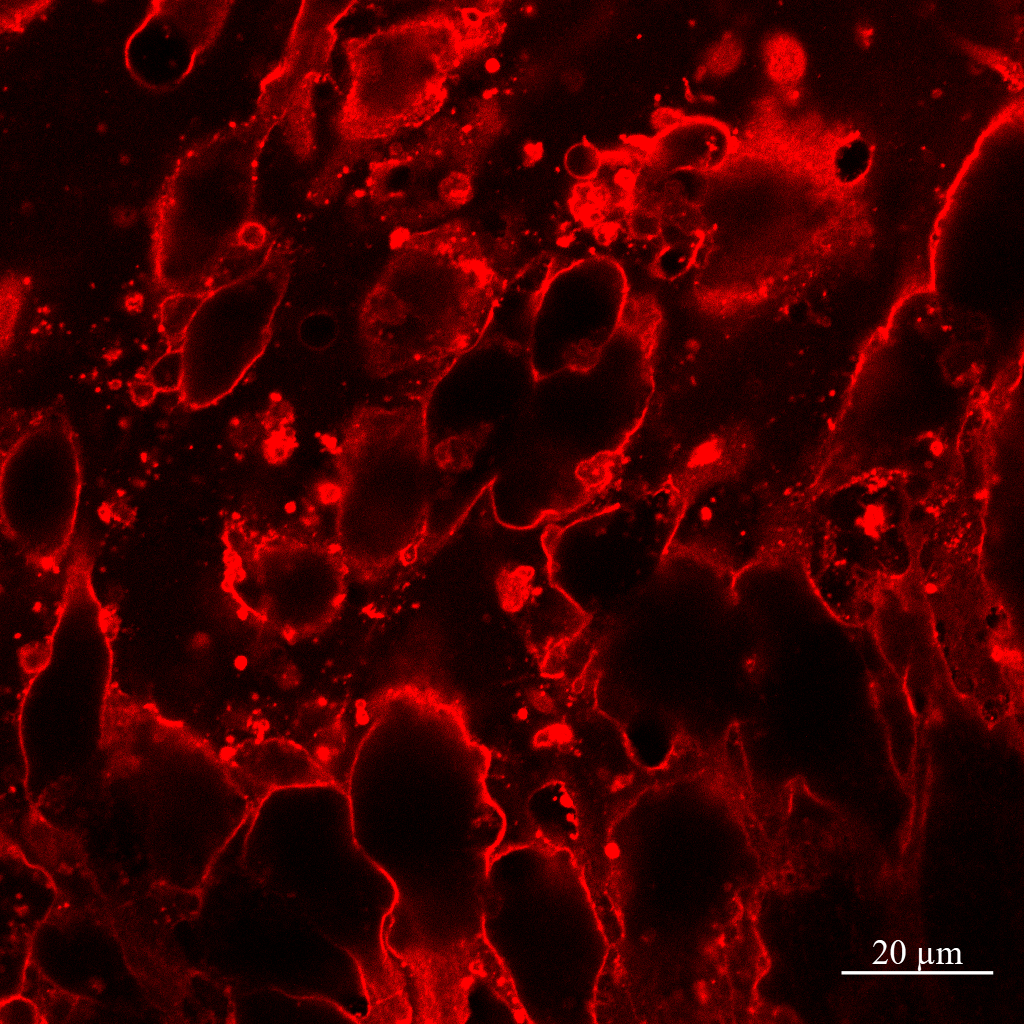

Supplement: Supplementary material — Original Images for Fig 3_Fig 4.zip [file IDRD_A_2585599_SM5405.zip › Original Image for Fig 4C Injured C6@PLTM (cytomembrane).tif]

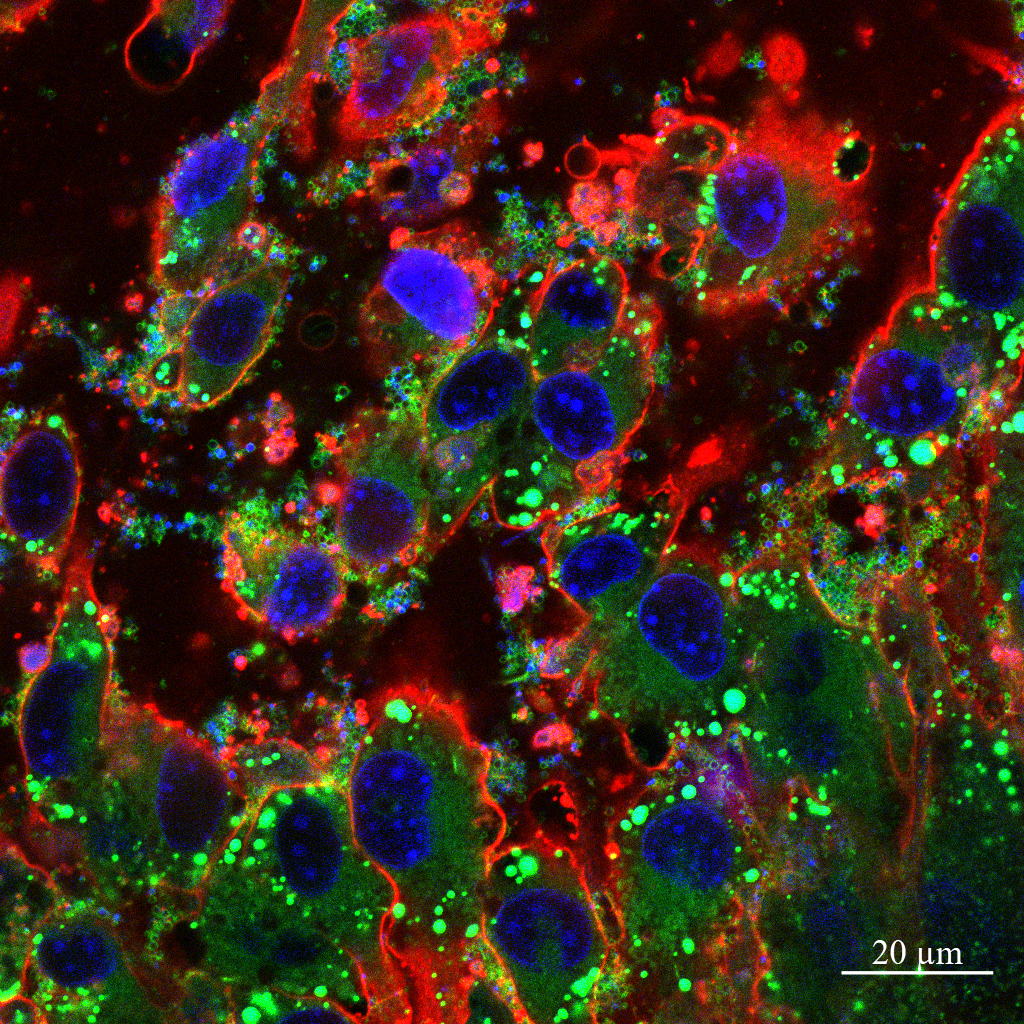

Supplement: Supplementary material — Original Images for Fig 3_Fig 4.zip [file IDRD_A_2585599_SM5405.zip › Original Image for Fig 4C Injured C6@PLTM (merged).tif]

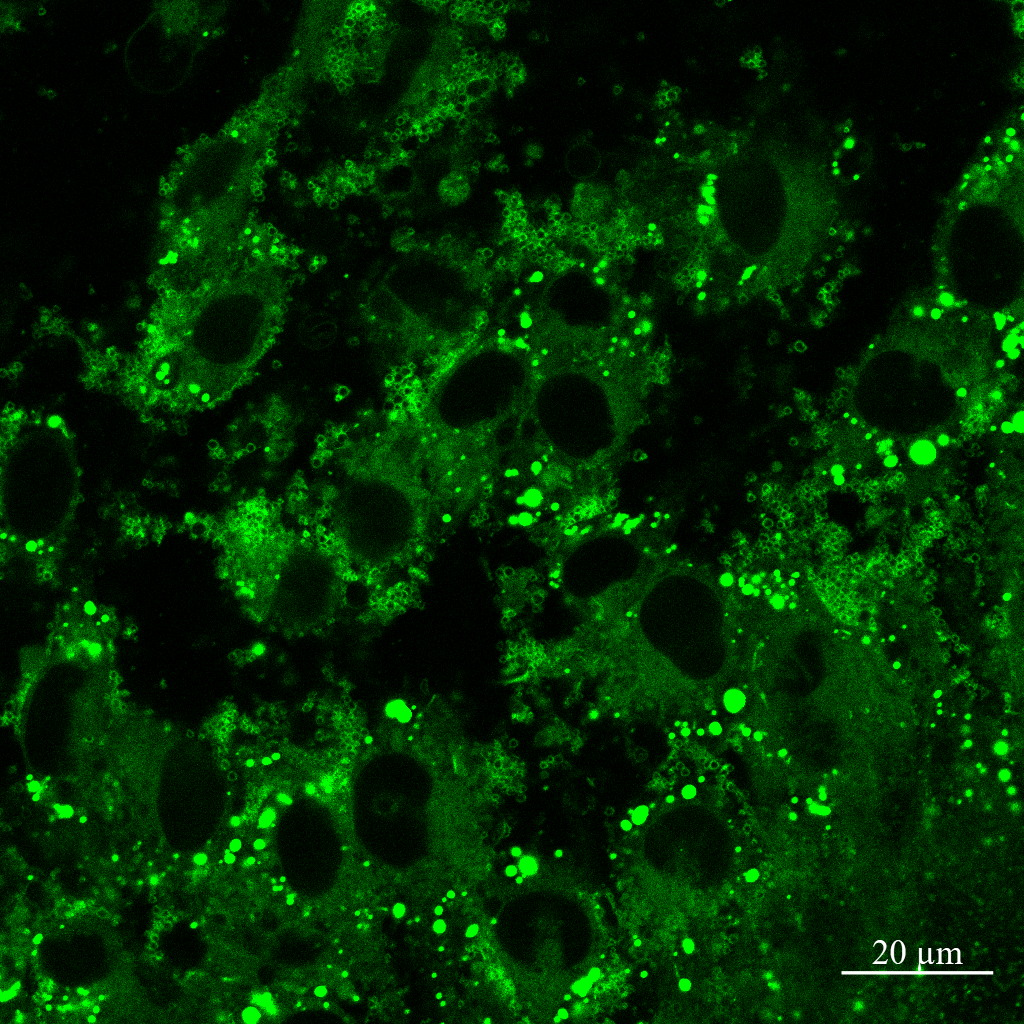

Supplement: Supplementary material — Original Images for Fig 3_Fig 4.zip [file IDRD_A_2585599_SM5405.zip › Original Image for Fig 4C Injured C6@PLTM (nanoparticles).tif]

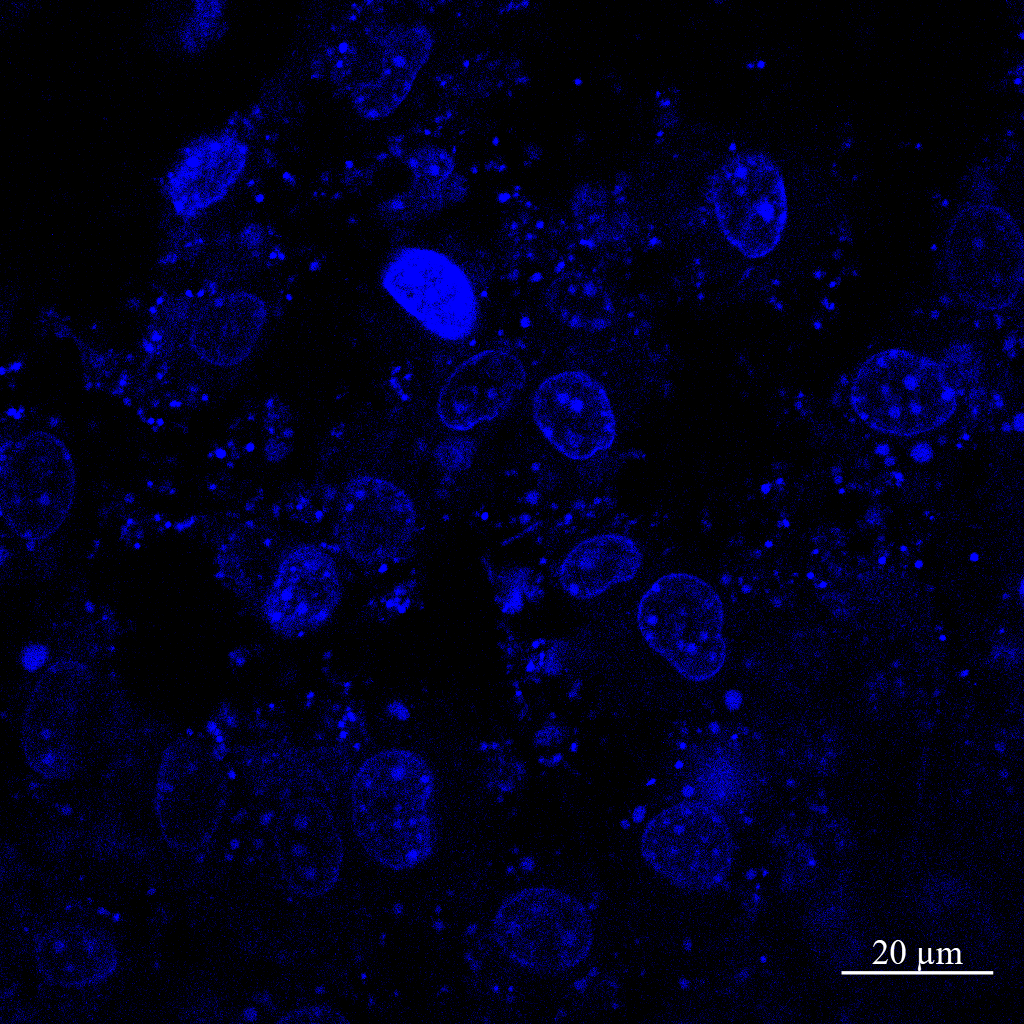

Supplement: Supplementary material — Original Images for Fig 3_Fig 4.zip [file IDRD_A_2585599_SM5405.zip › Original Image for Fig 4C Injured C6@PLTM (nucleus).tif]

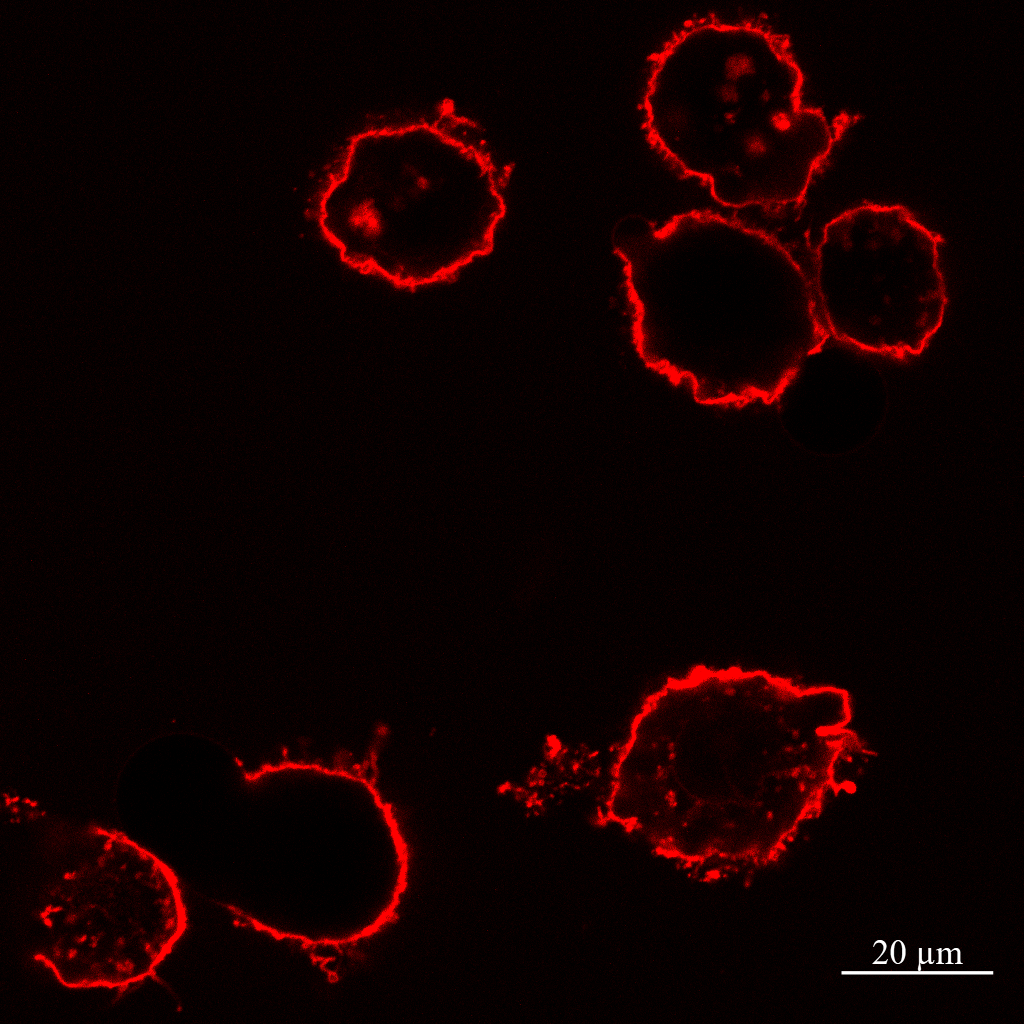

Supplement: Supplementary material — Original Images for Fig 3_Fig 4.zip [file IDRD_A_2585599_SM5405.zip › Original Image for Fig 4D Healthy C6@PLGA (cytomembrane).tif]

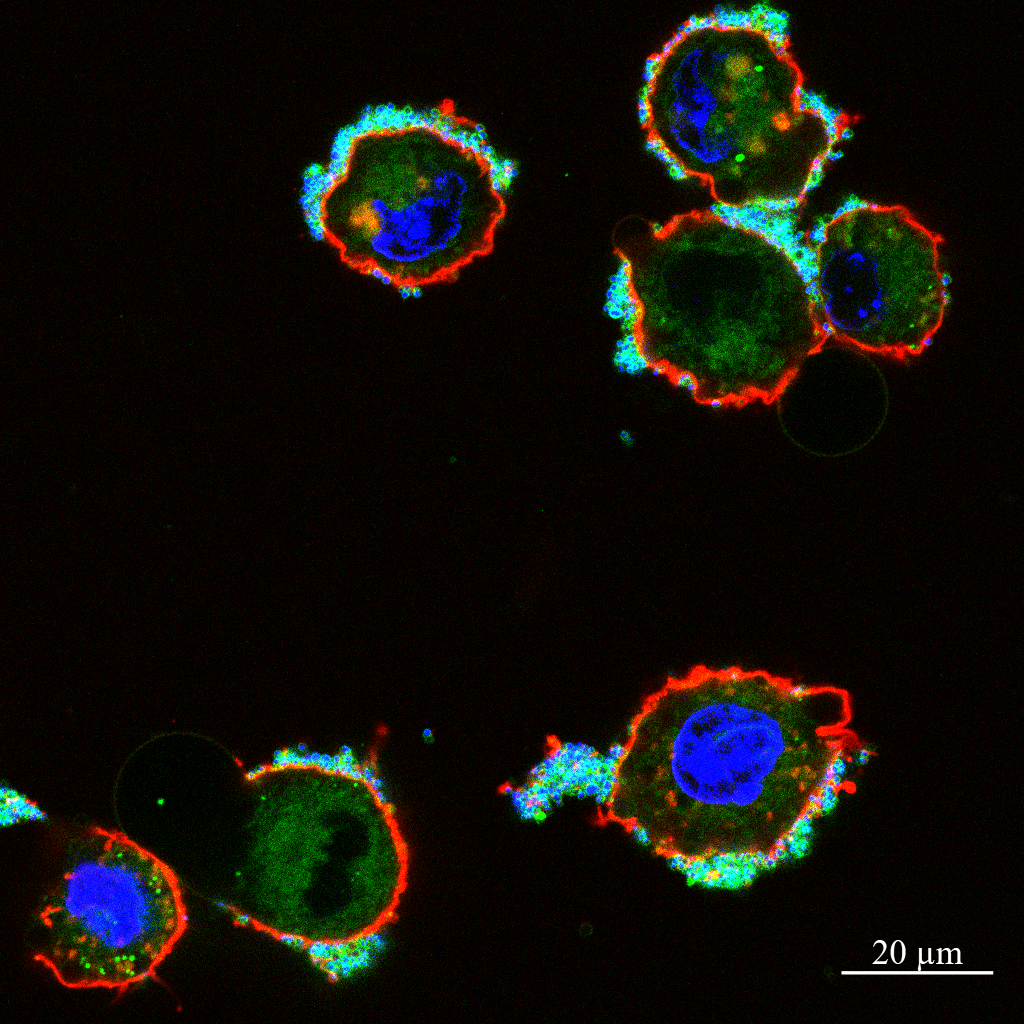

Supplement: Supplementary material — Original Images for Fig 3_Fig 4.zip [file IDRD_A_2585599_SM5405.zip › Original Image for Fig 4D Healthy C6@PLGA (merged).tif]

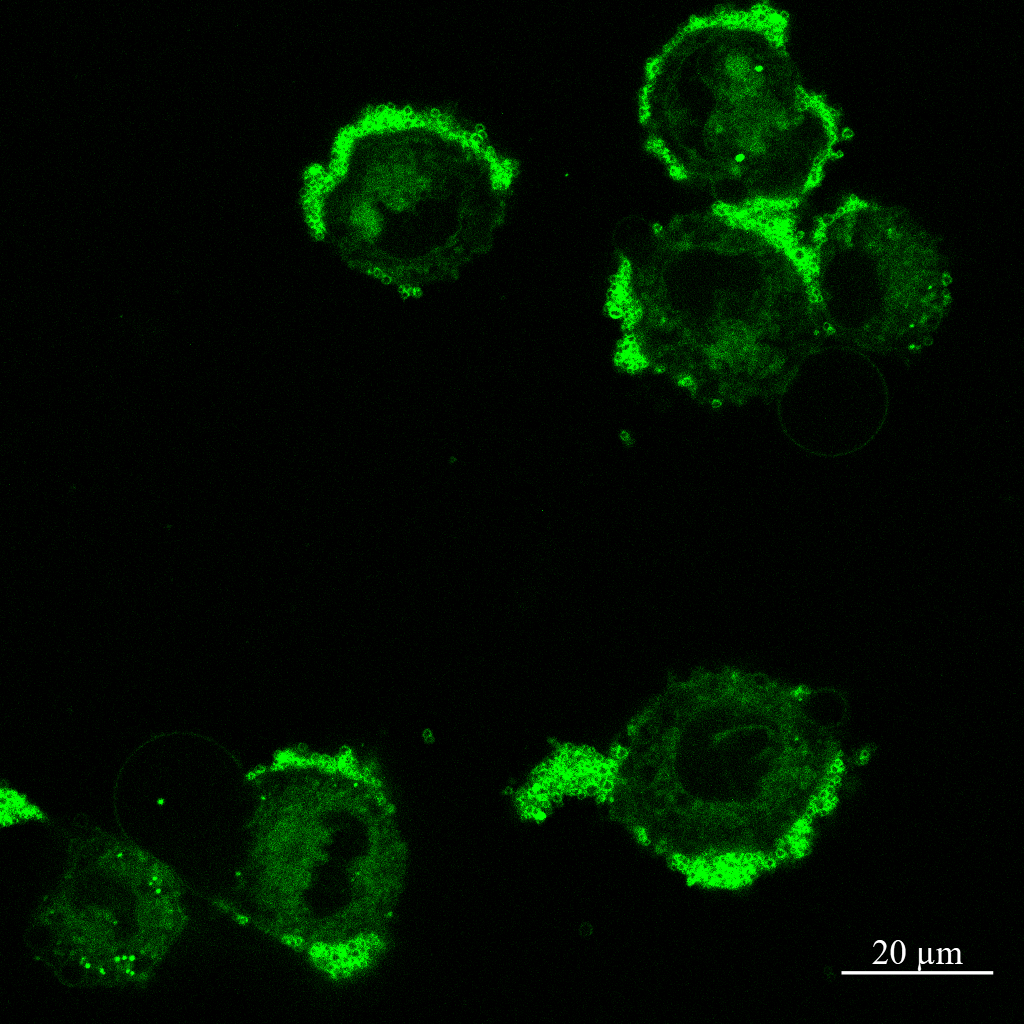

Supplement: Supplementary material — Original Images for Fig 3_Fig 4.zip [file IDRD_A_2585599_SM5405.zip › Original Image for Fig 4D Healthy C6@PLGA (nanoparticles).tif]

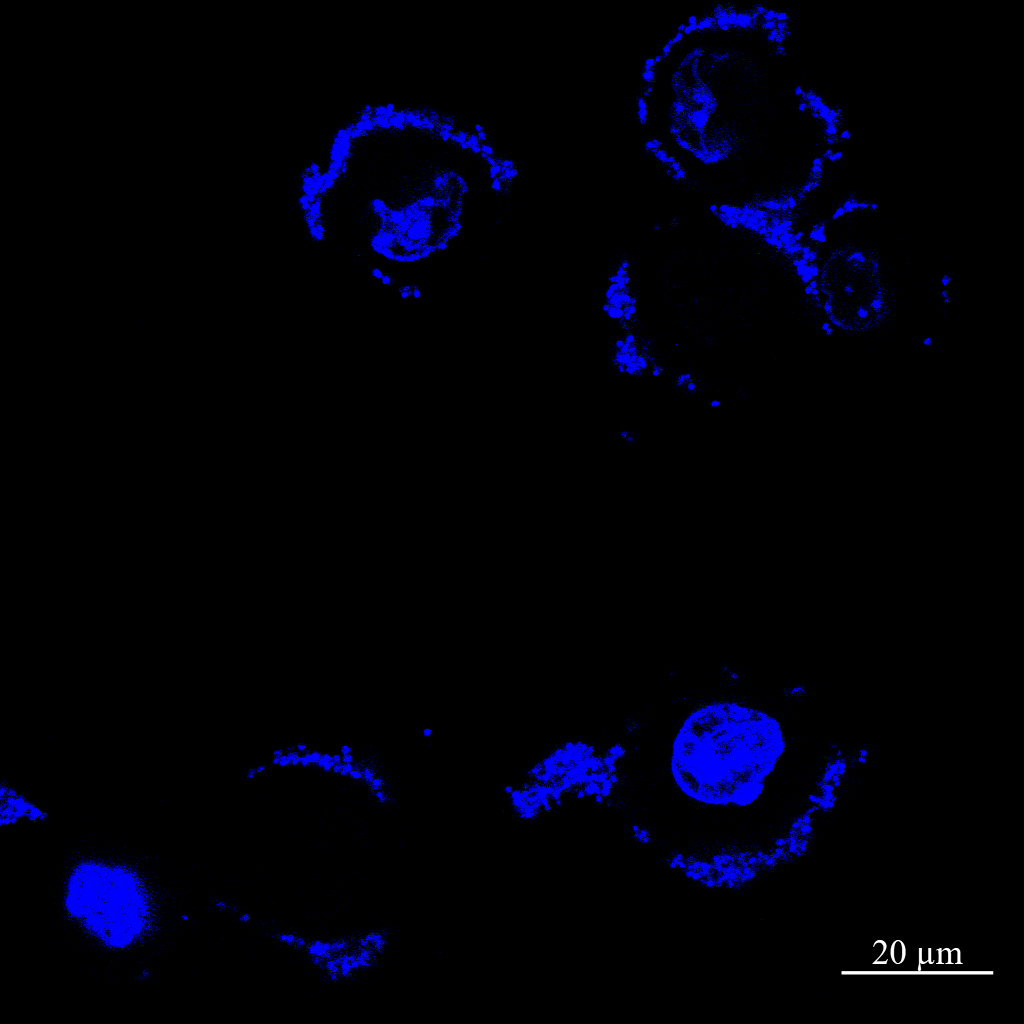

Supplement: Supplementary material — Original Images for Fig 3_Fig 4.zip [file IDRD_A_2585599_SM5405.zip › Original Image for Fig 4D Healthy C6@PLGA (nucleus).tif]

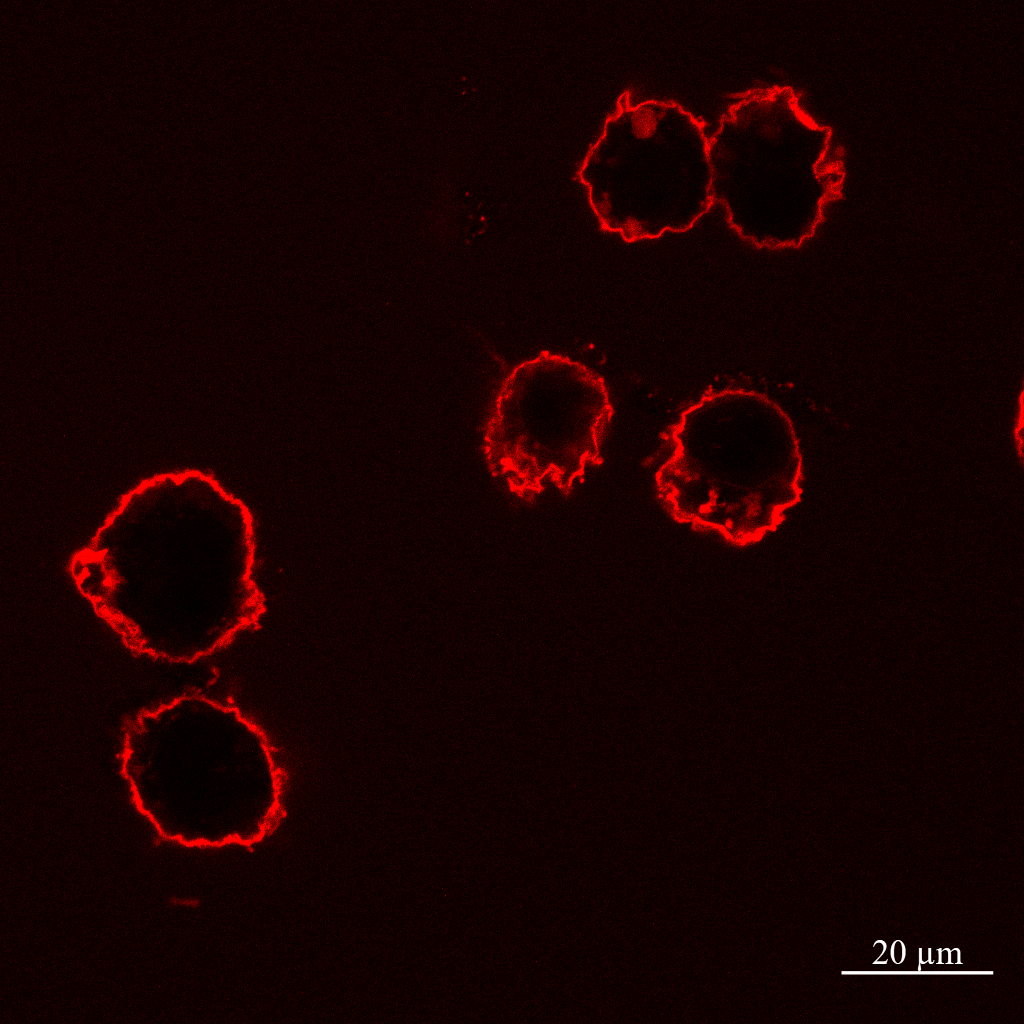

Supplement: Supplementary material — Original Images for Fig 3_Fig 4.zip [file IDRD_A_2585599_SM5405.zip › Original Image for Fig 4D Healthy C6@PLTM (cytomembrane).tif]

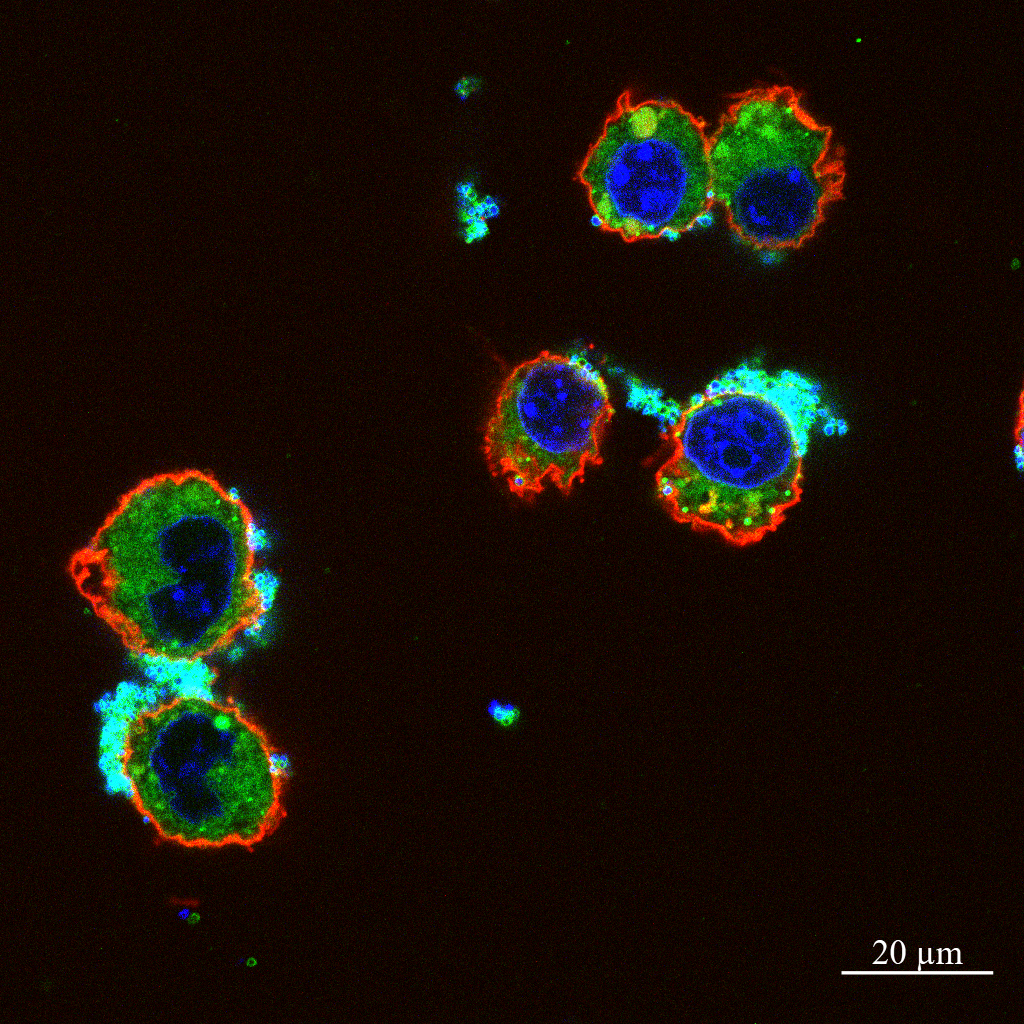

Supplement: Supplementary material — Original Images for Fig 3_Fig 4.zip [file IDRD_A_2585599_SM5405.zip › Original Image for Fig 4D Healthy C6@PLTM (merged).tif]

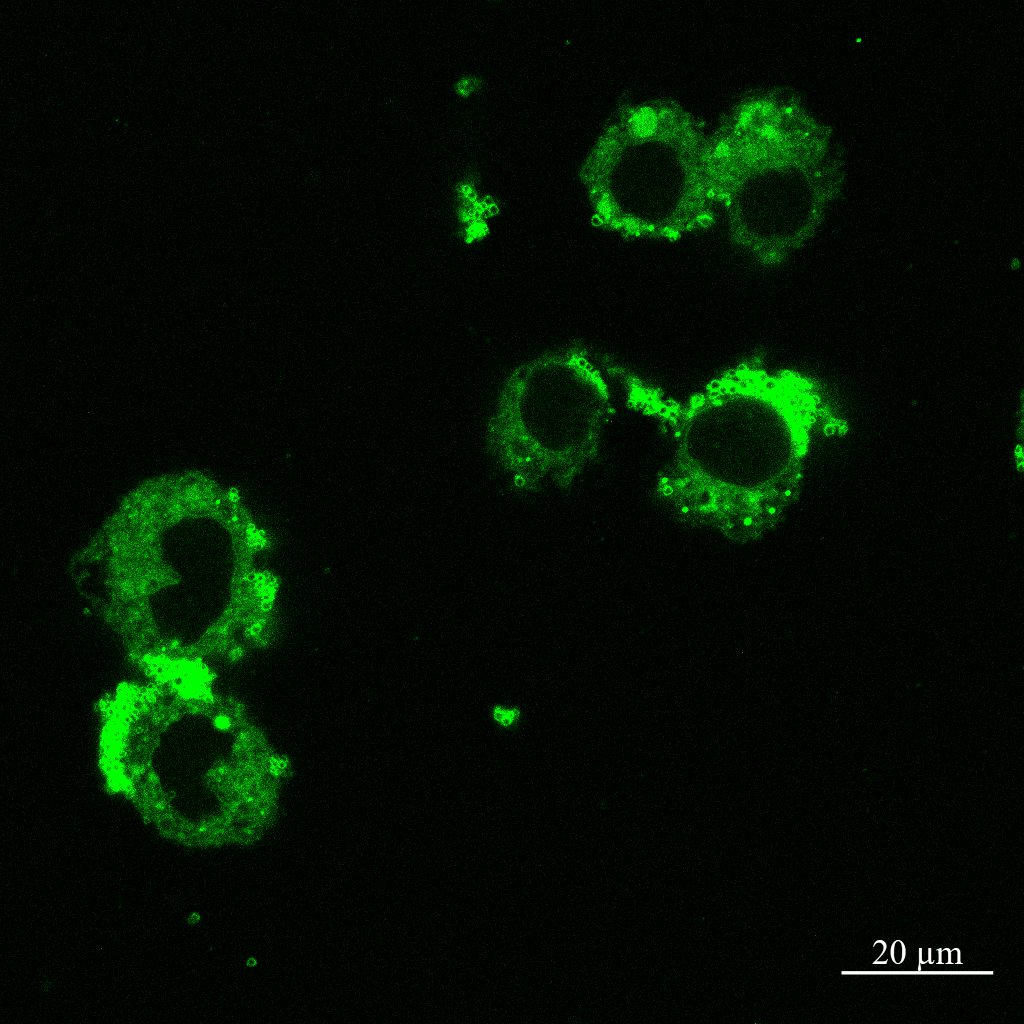

Supplement: Supplementary material — Original Images for Fig 3_Fig 4.zip [file IDRD_A_2585599_SM5405.zip › Original Image for Fig 4D Healthy C6@PLTM (nanoparticles).tif]

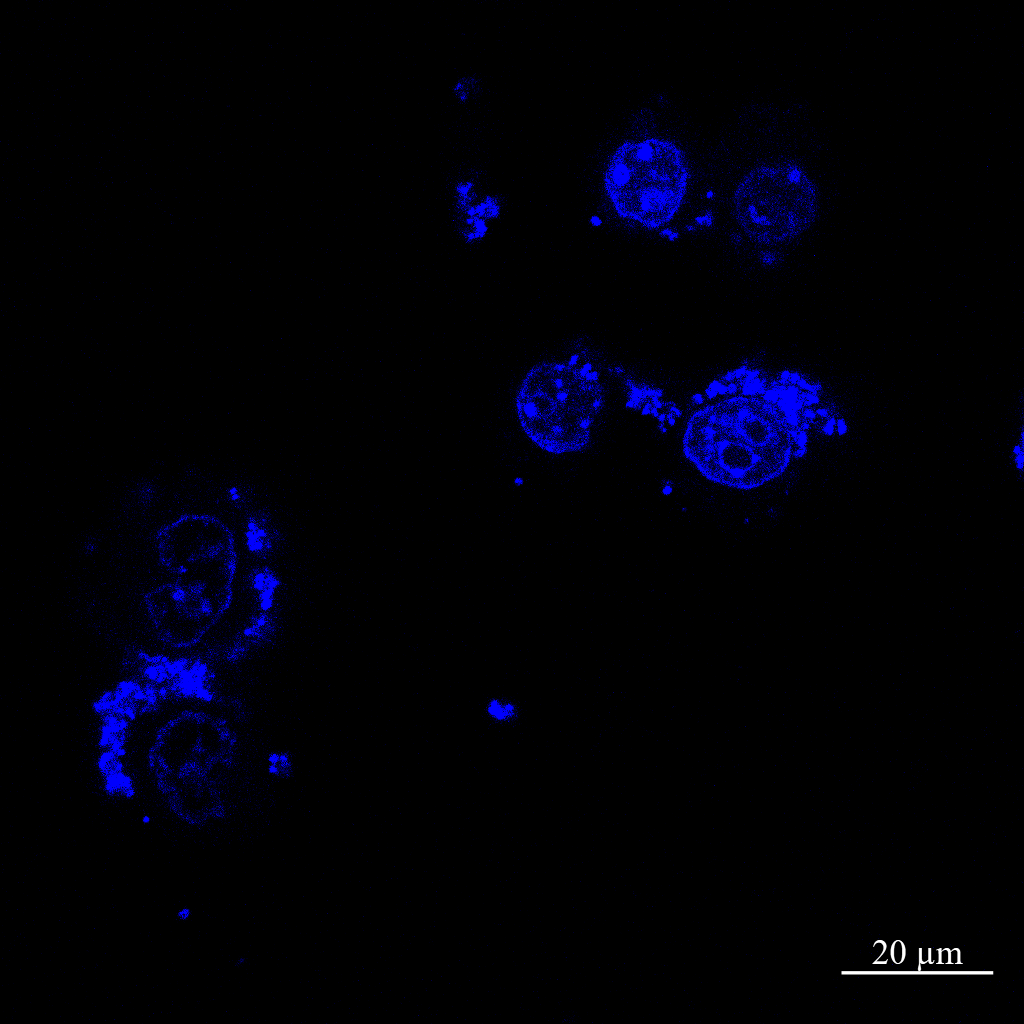

Supplement: Supplementary material — Original Images for Fig 3_Fig 4.zip [file IDRD_A_2585599_SM5405.zip › Original Image for Fig 4D Healthy C6@PLTM (nucleus).tif]

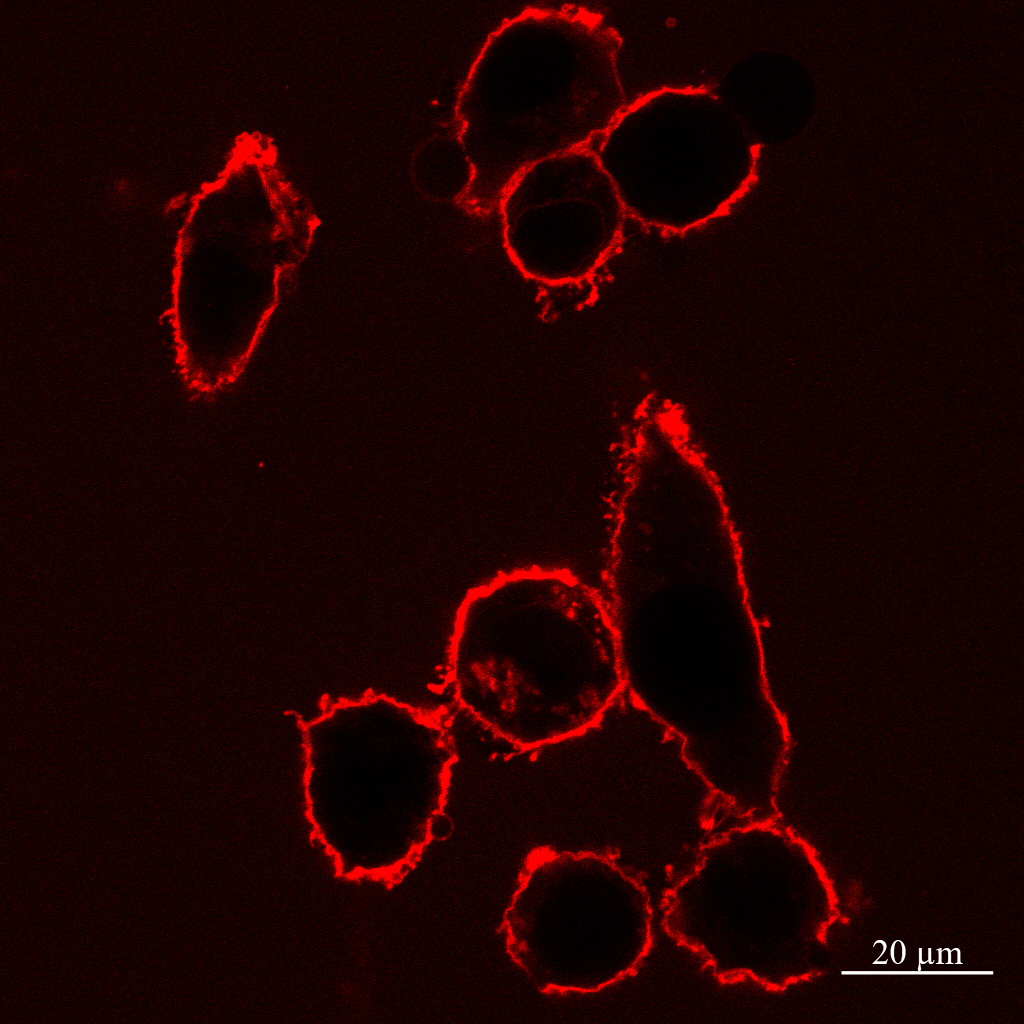

Supplement: Supplementary material — Original Images for Fig 3_Fig 4.zip [file IDRD_A_2585599_SM5405.zip › Original Image for Fig 4D Injured C6@PLGA (cytomembrane).tif]

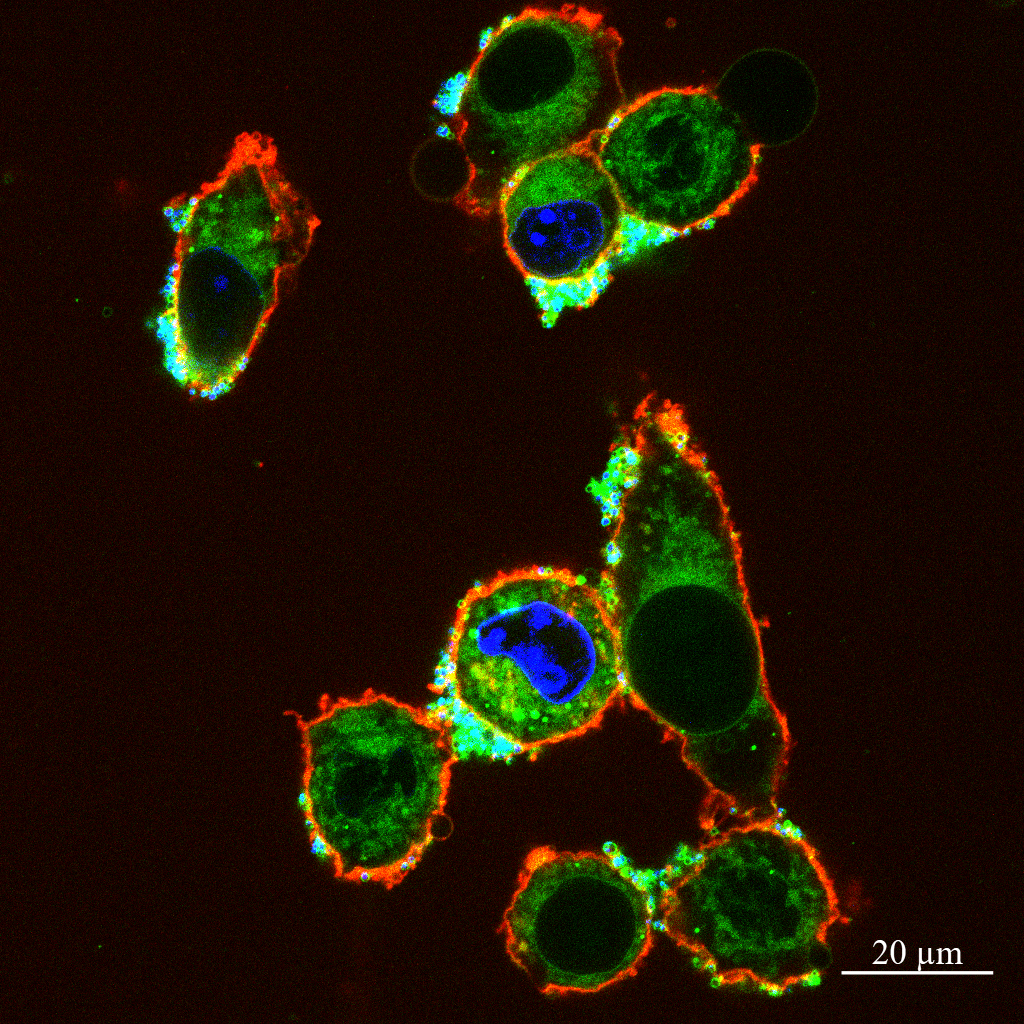

Supplement: Supplementary material — Original Images for Fig 3_Fig 4.zip [file IDRD_A_2585599_SM5405.zip › Original Image for Fig 4D Injured C6@PLGA (merged).tif]

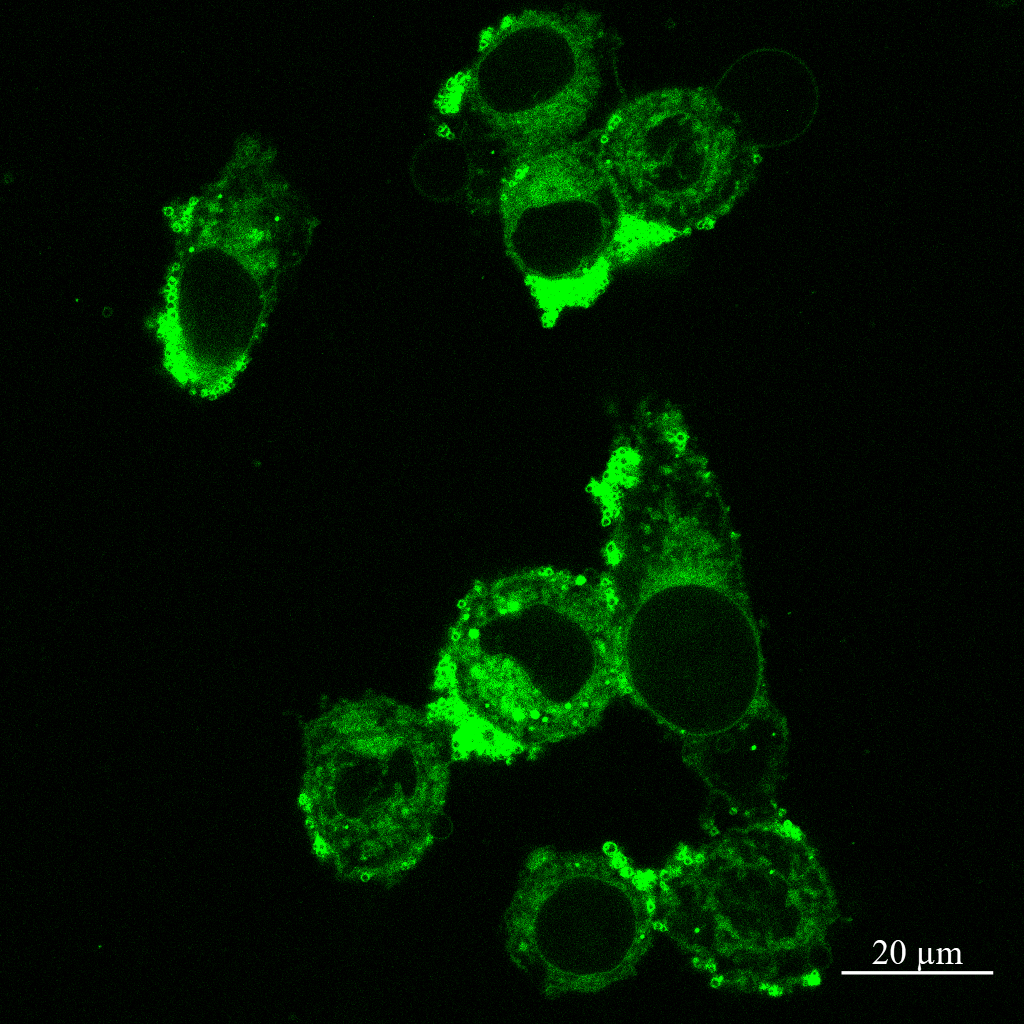

Supplement: Supplementary material — Original Images for Fig 3_Fig 4.zip [file IDRD_A_2585599_SM5405.zip › Original Image for Fig 4D Injured C6@PLGA (nanoparticles).tif]

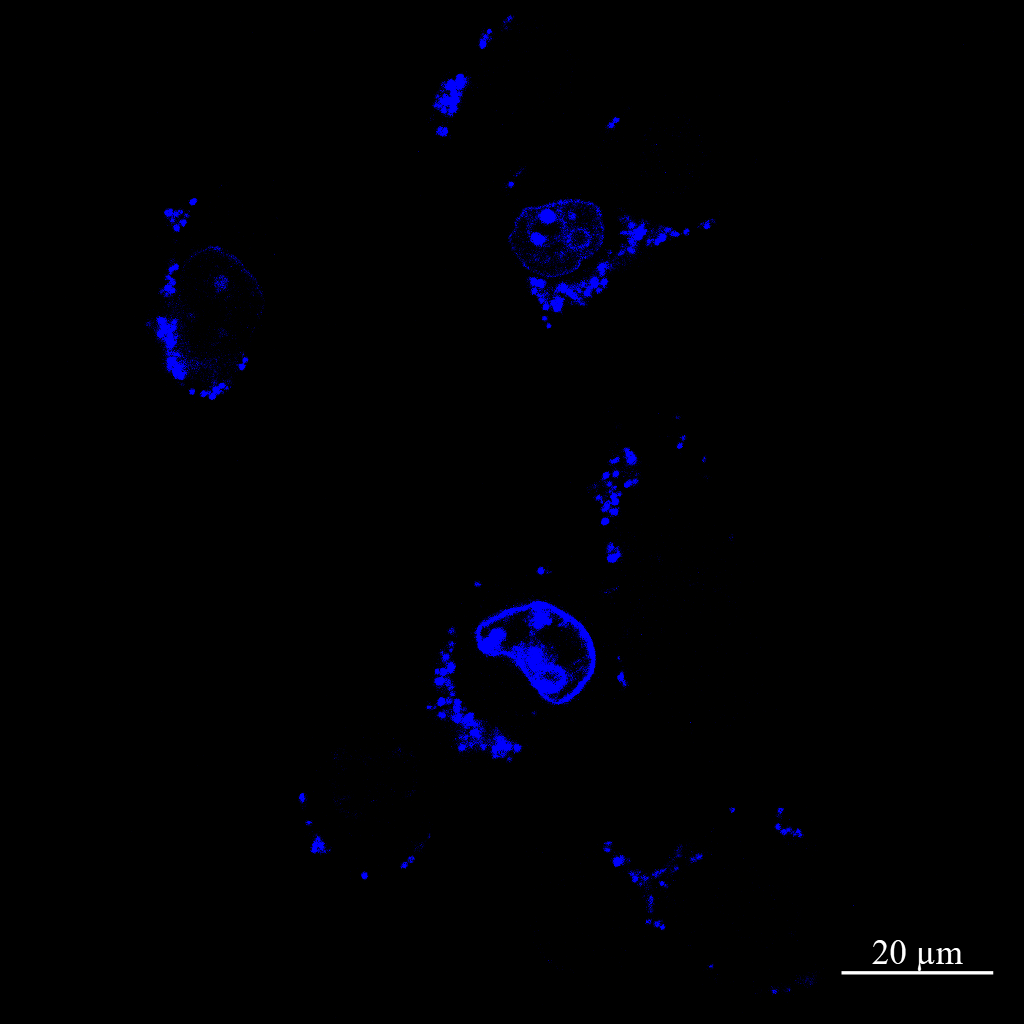

Supplement: Supplementary material — Original Images for Fig 3_Fig 4.zip [file IDRD_A_2585599_SM5405.zip › Original Image for Fig 4D Injured C6@PLGA (nucleus).tif]

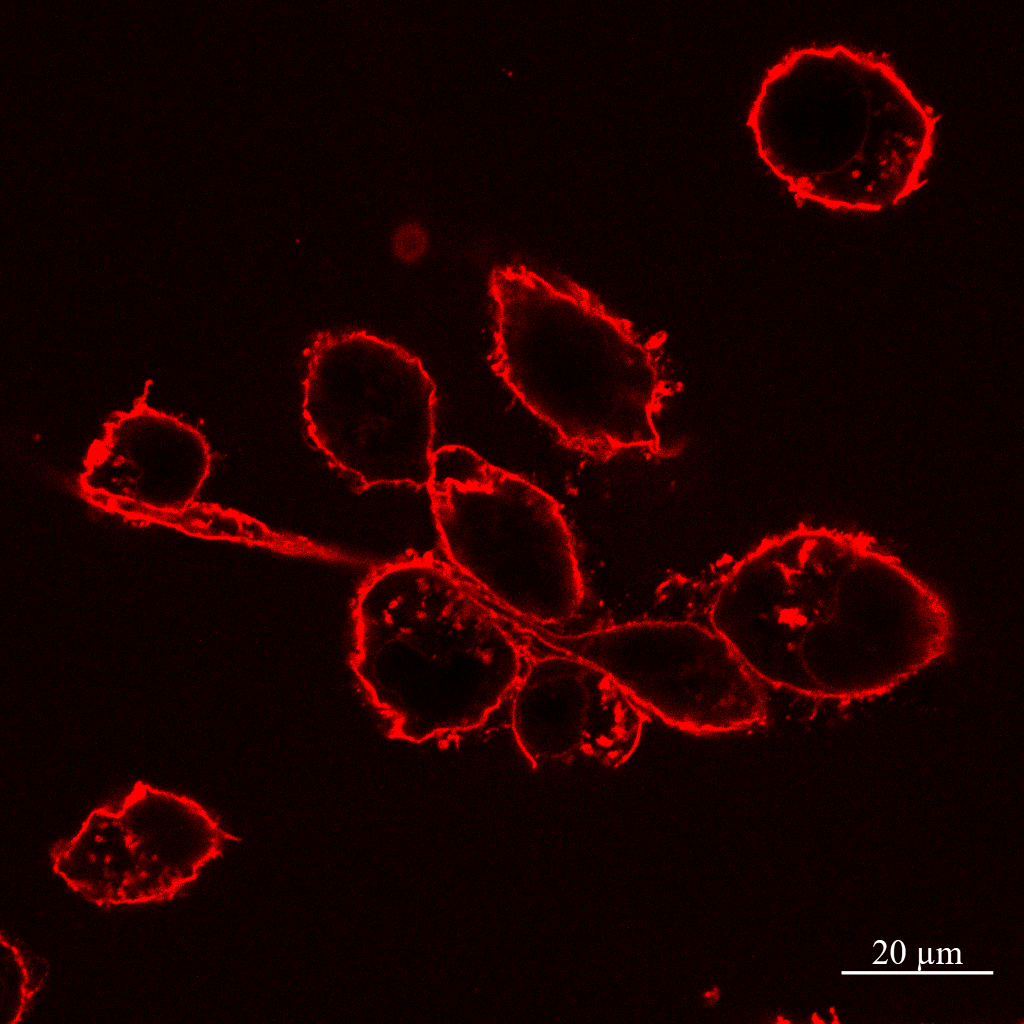

Supplement: Supplementary material — Original Images for Fig 3_Fig 4.zip [file IDRD_A_2585599_SM5405.zip › Original Image for Fig 4D Injured C6@PLTM (cytomembrane).tif]

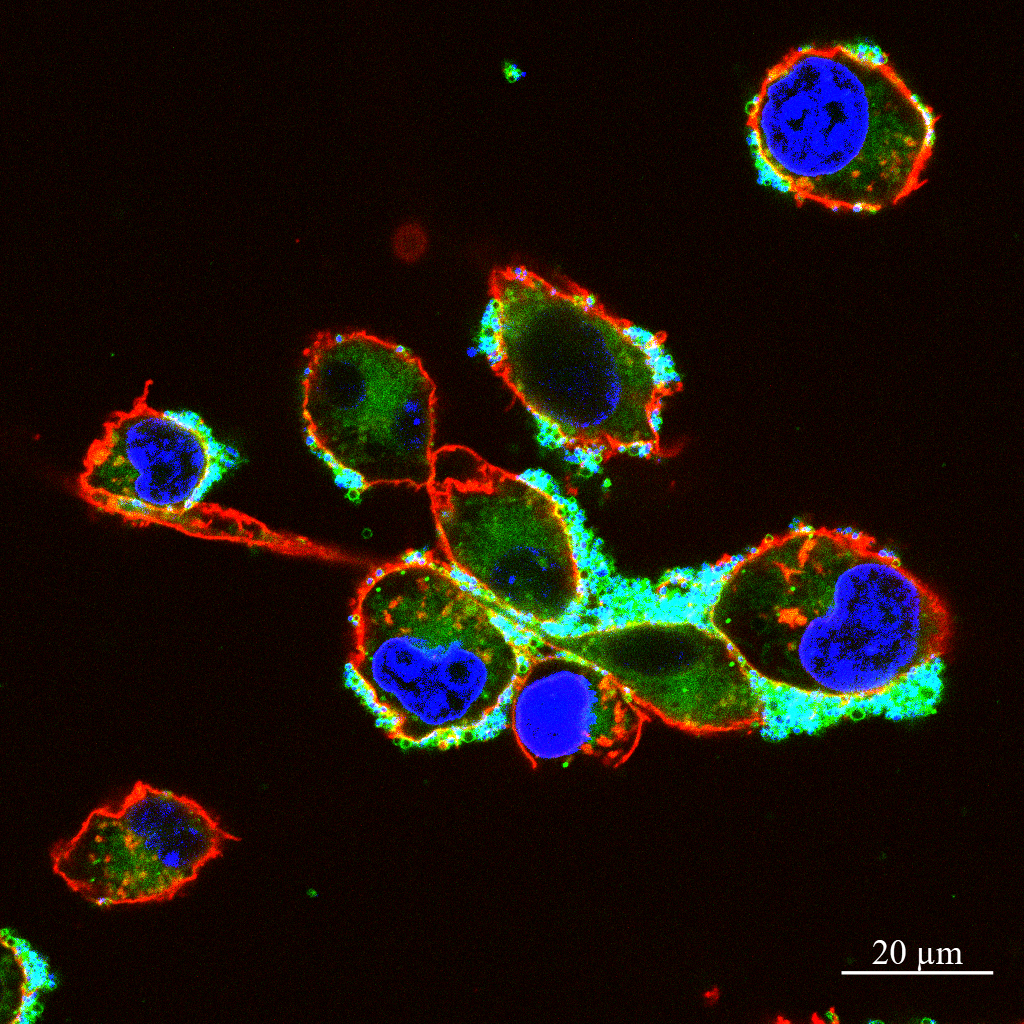

Supplement: Supplementary material — Original Images for Fig 3_Fig 4.zip [file IDRD_A_2585599_SM5405.zip › Original Image for Fig 4D Injured C6@PLTM (merged).tif]

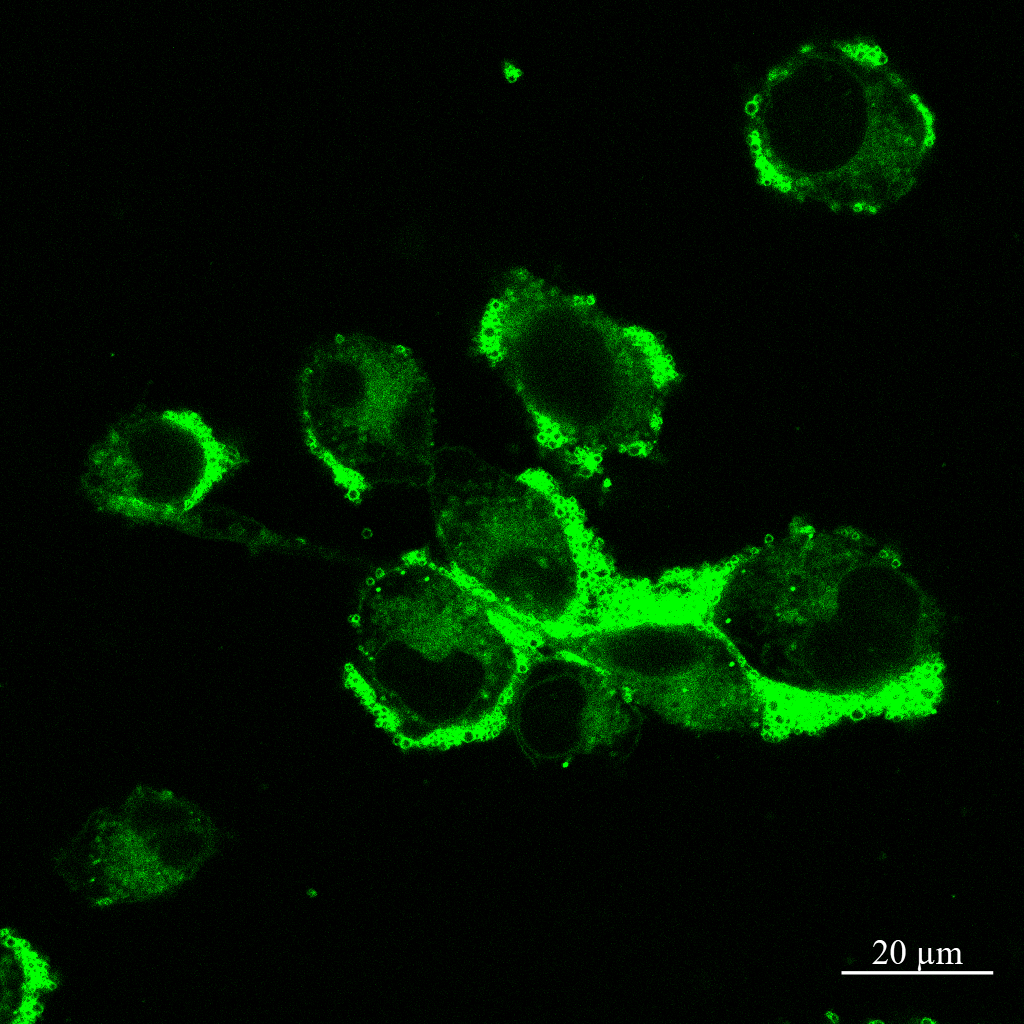

Supplement: Supplementary material — Original Images for Fig 3_Fig 4.zip [file IDRD_A_2585599_SM5405.zip › Original Image for Fig 4D Injured C6@PLTM (nanoparticles).tif]

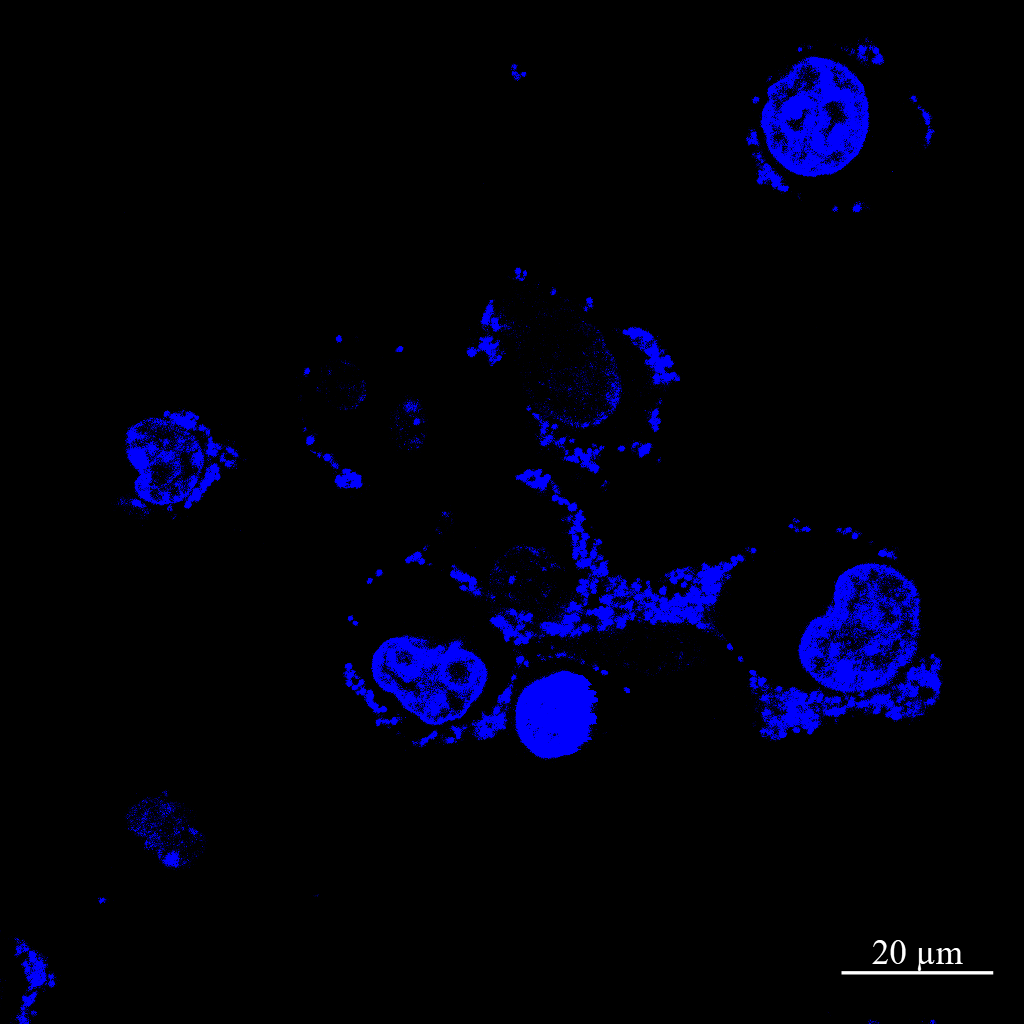

Supplement: Supplementary material — Original Images for Fig 3_Fig 4.zip [file IDRD_A_2585599_SM5405.zip › Original Image for Fig 4D Injured C6@PLTM (nucleus).tif]

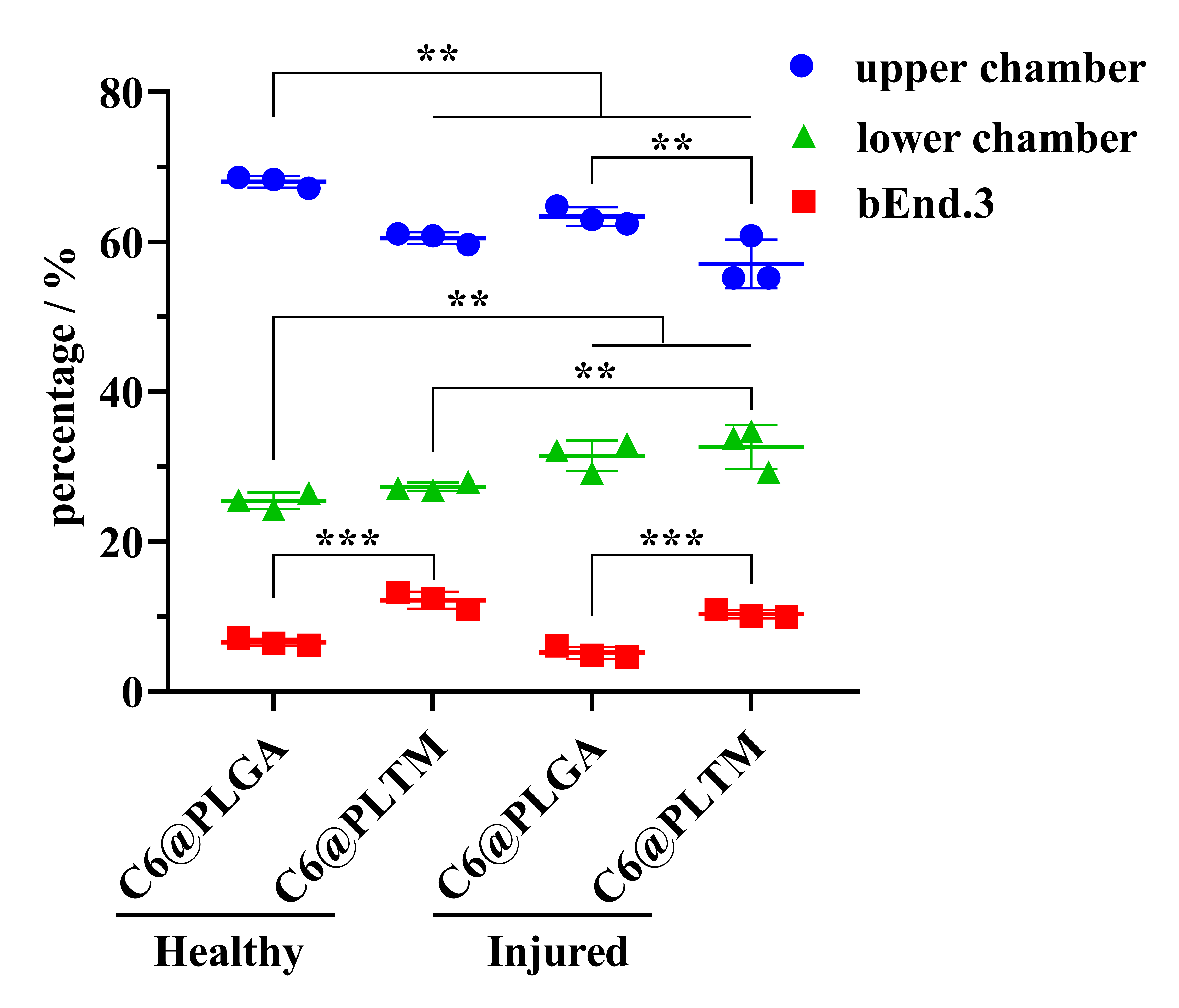

Supplement: Supplementary material — Original Images for Fig 3_Fig 4.zip [file IDRD_A_2585599_SM5405.zip › Original Image for Fig 4E.tif]

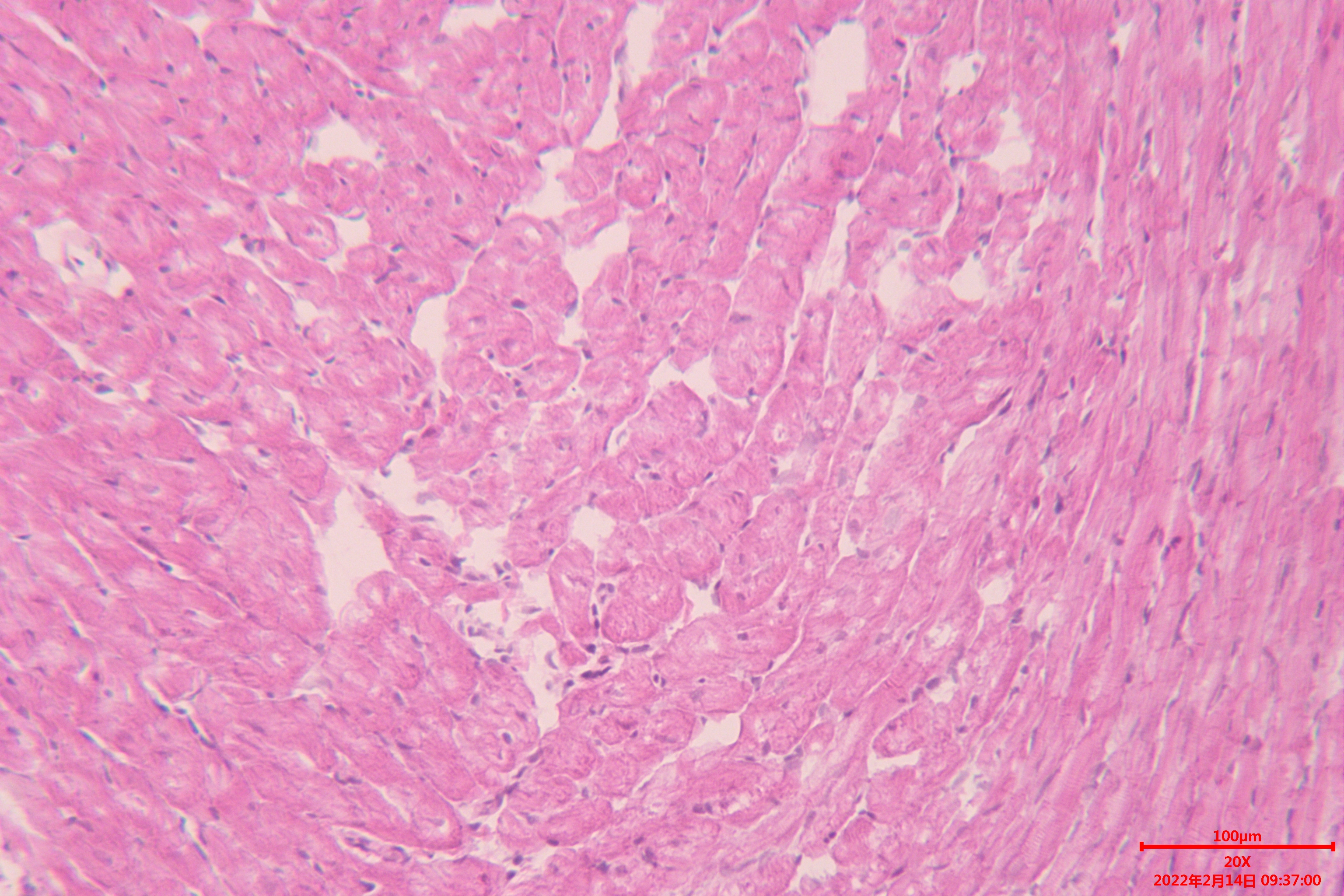

Supplement: Supplementary material — Original Images for Fig S10_1.zip [file IDRD_A_2585599_SM5406.zip › Original Image for Fig S10 G1 (heart).tif]

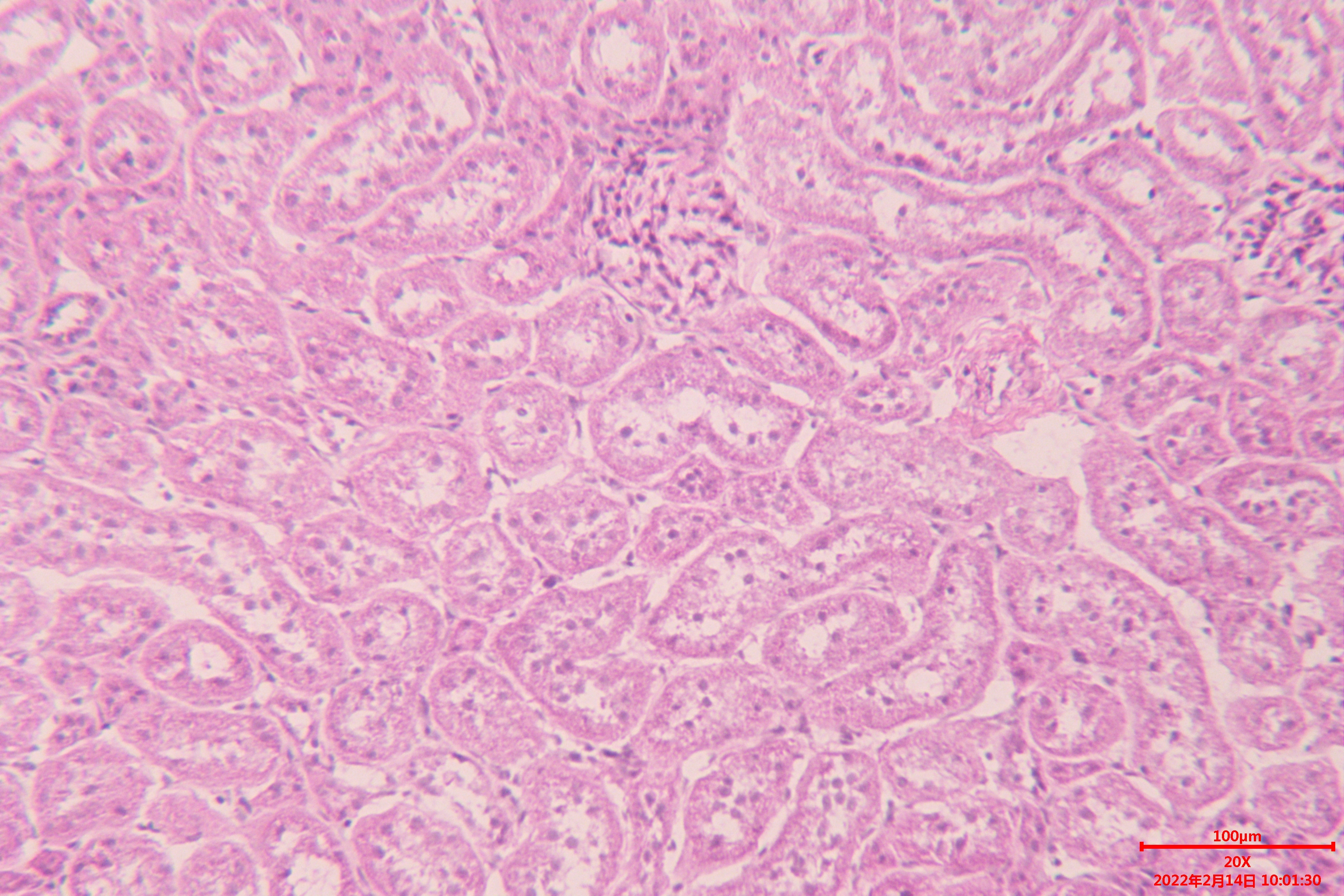

Supplement: Supplementary material — Original Images for Fig S10_1.zip [file IDRD_A_2585599_SM5406.zip › Original Image for Fig S10 G1 (kidney).tif]

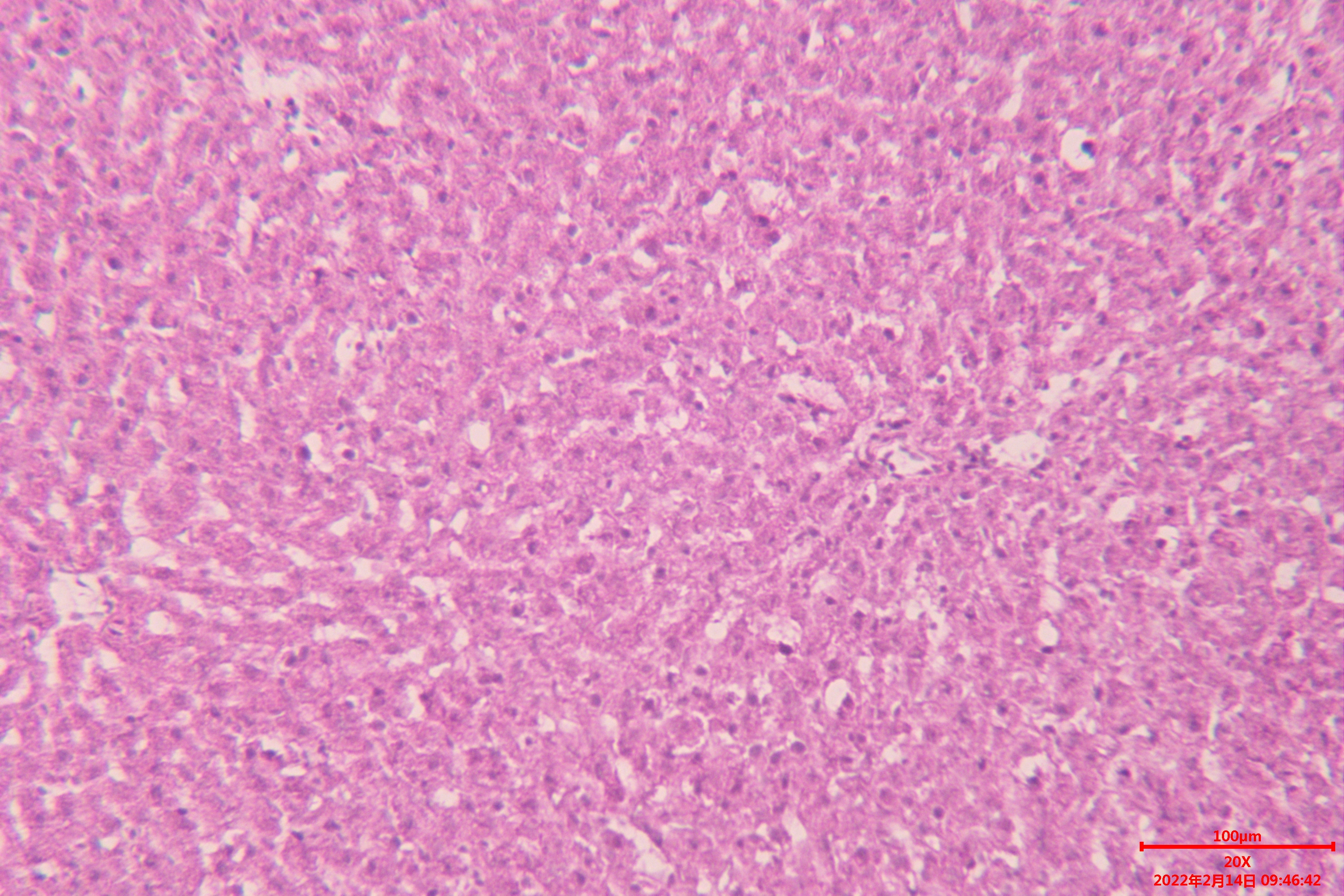

Supplement: Supplementary material — Original Images for Fig S10_1.zip [file IDRD_A_2585599_SM5406.zip › Original Image for Fig S10 G1 (liver).tif]

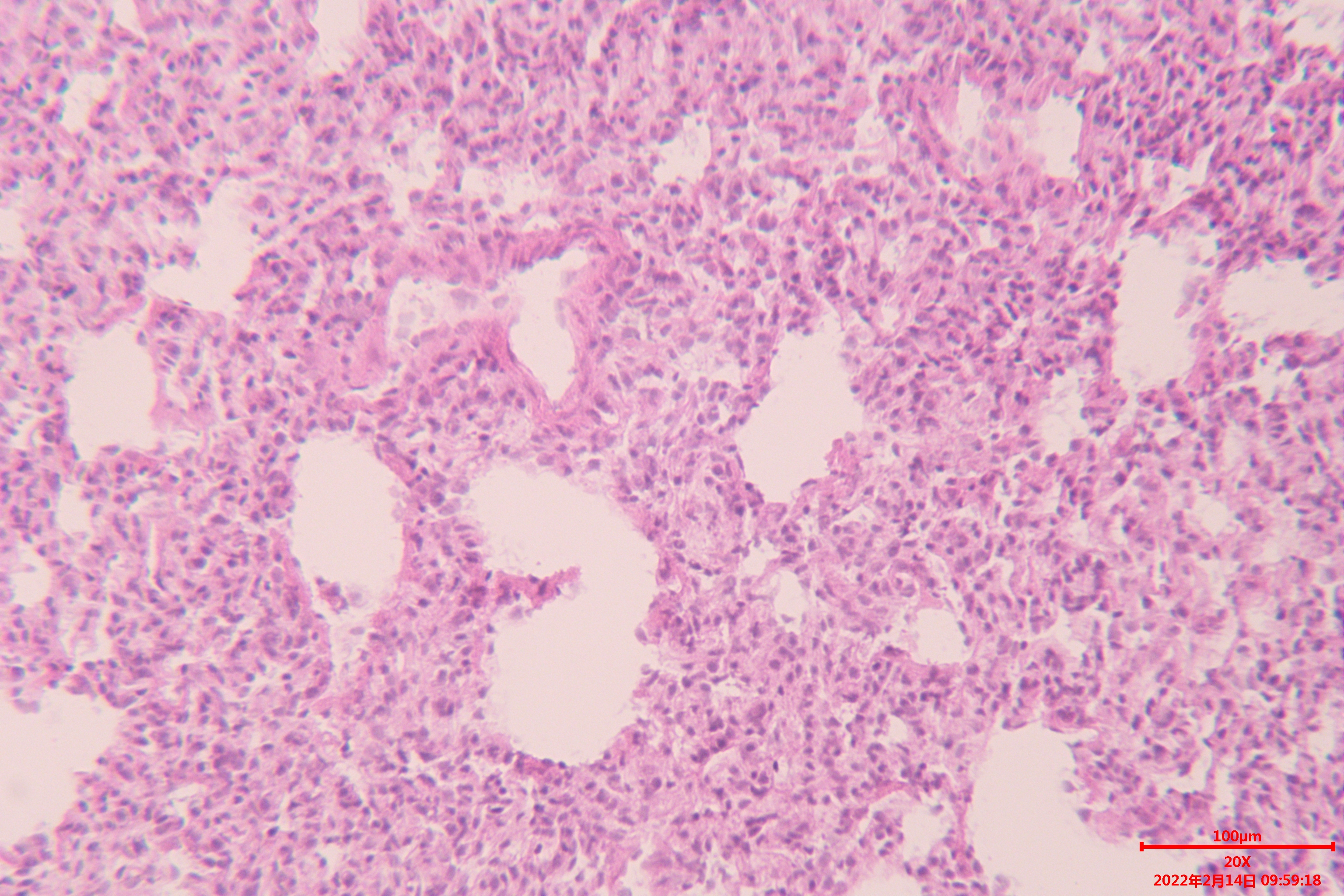

Supplement: Supplementary material — Original Images for Fig S10_1.zip [file IDRD_A_2585599_SM5406.zip › Original Image for Fig S10 G1 (lung).tif]

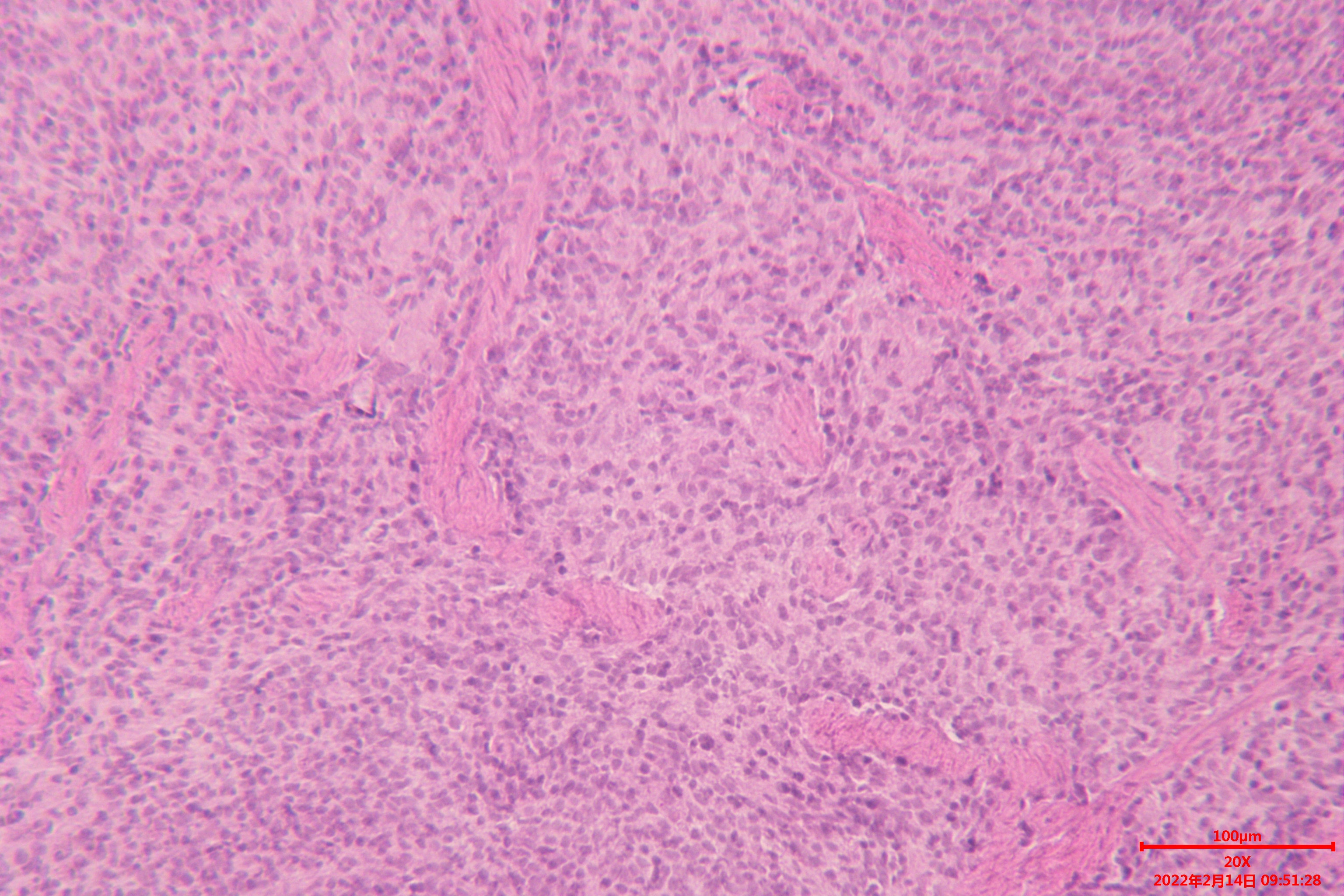

Supplement: Supplementary material — Original Images for Fig S10_1.zip [file IDRD_A_2585599_SM5406.zip › Original Image for Fig S10 G1 (spleen).tif]

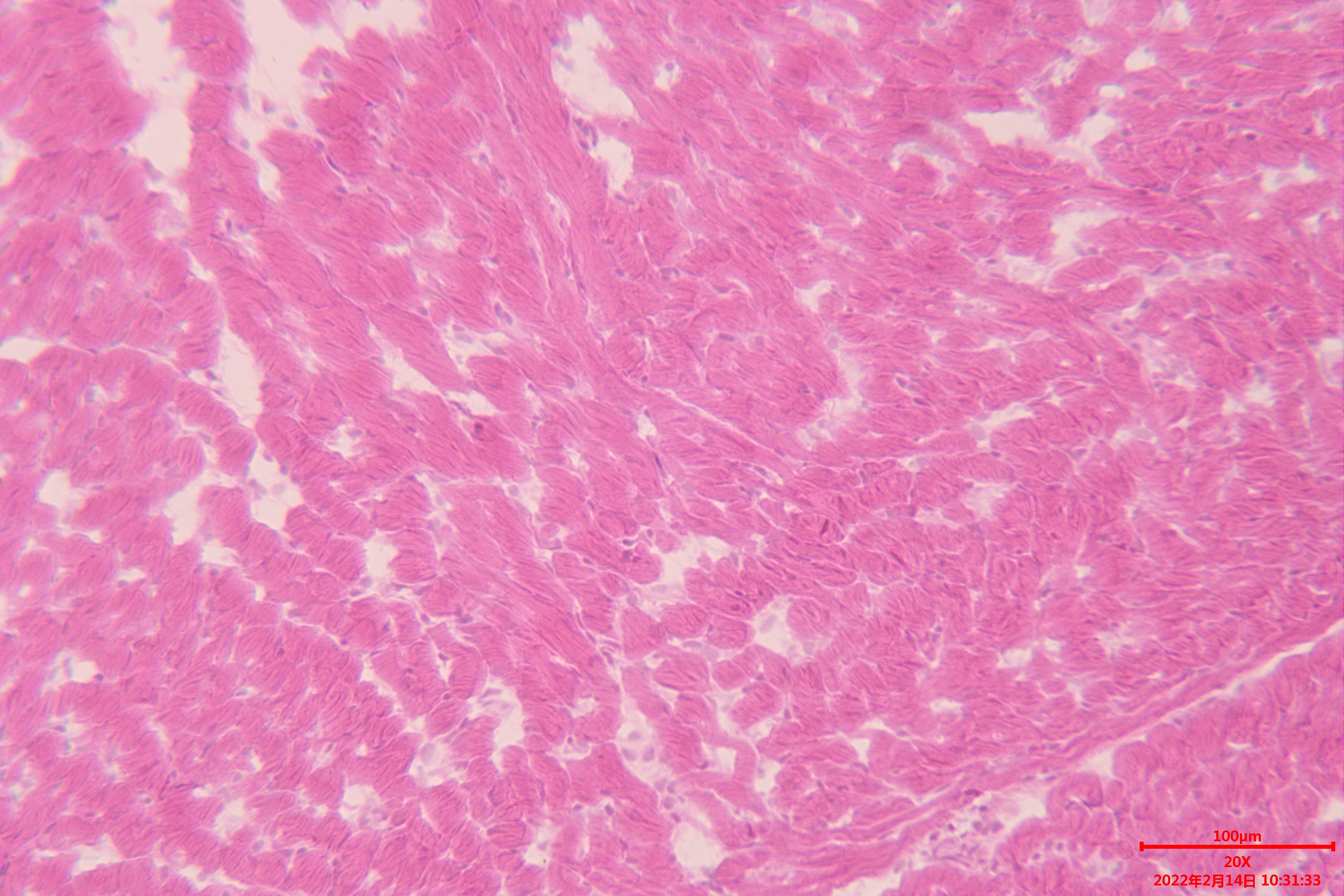

Supplement: Supplementary material — Original Images for Fig S10_1.zip [file IDRD_A_2585599_SM5406.zip › Original Image for Fig S10 G2 (heart).tif]

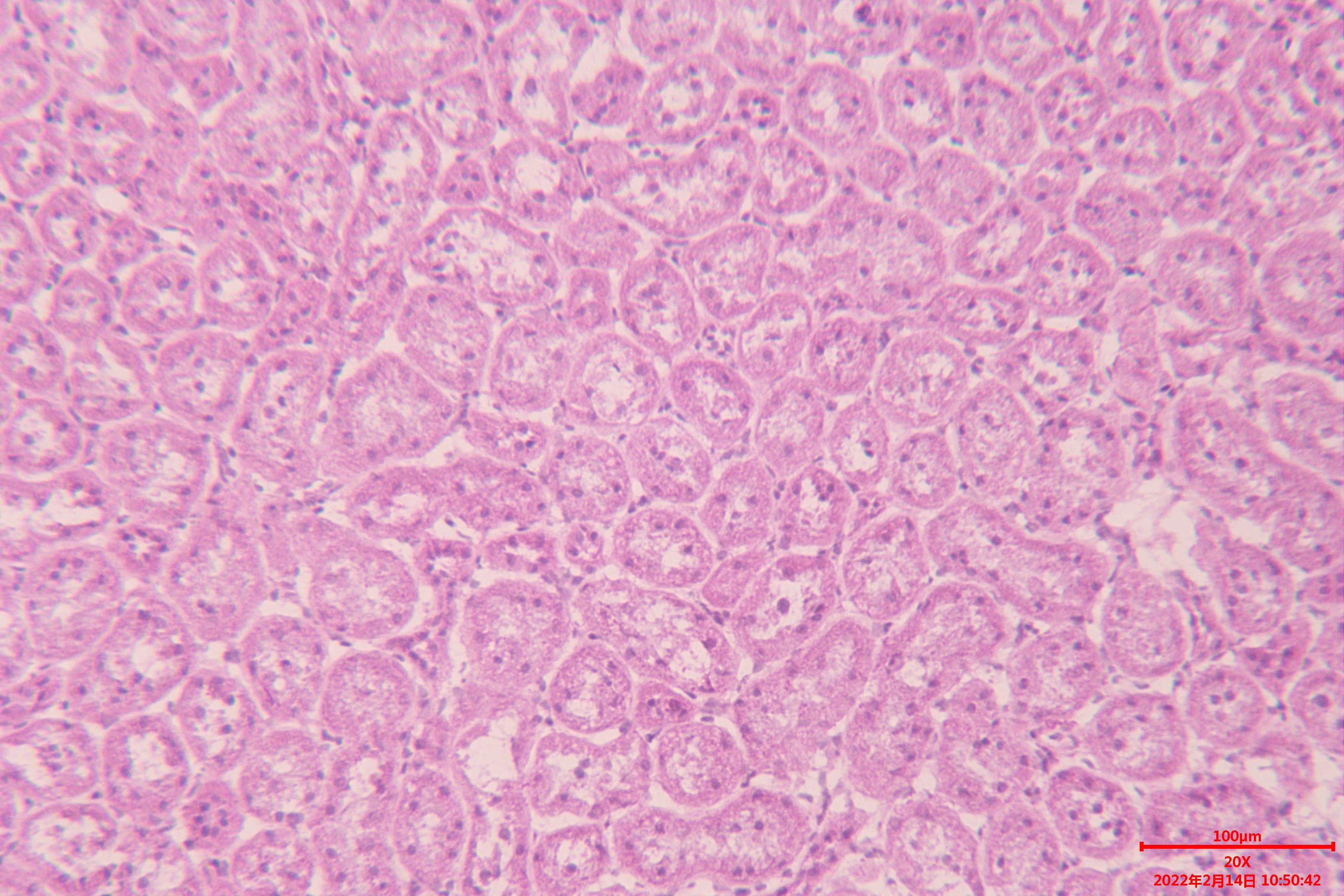

Supplement: Supplementary material — Original Images for Fig S10_1.zip [file IDRD_A_2585599_SM5406.zip › Original Image for Fig S10 G2 (kidney).tif]

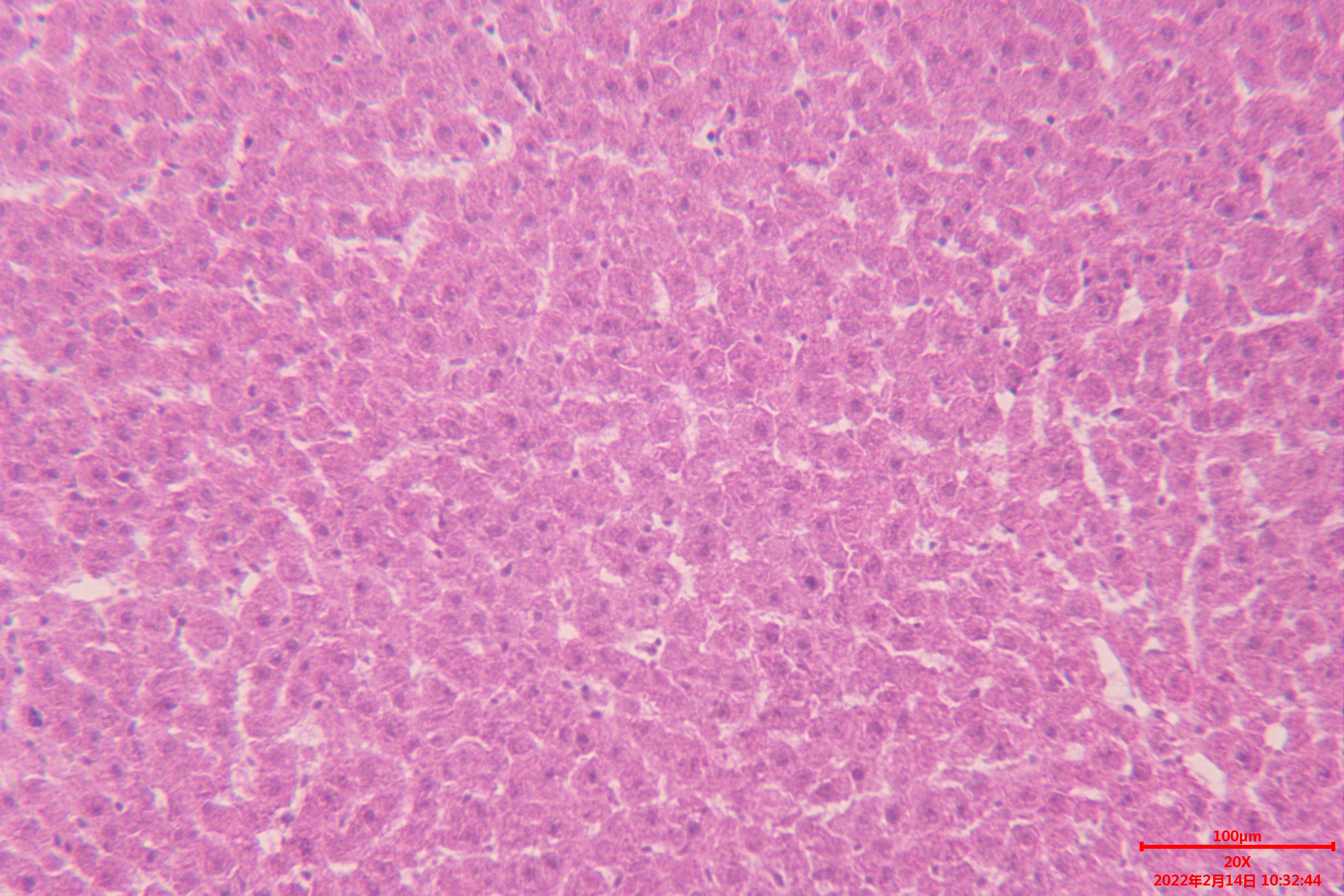

Supplement: Supplementary material — Original Images for Fig S10_1.zip [file IDRD_A_2585599_SM5406.zip › Original Image for Fig S10 G2 (liver).tif]

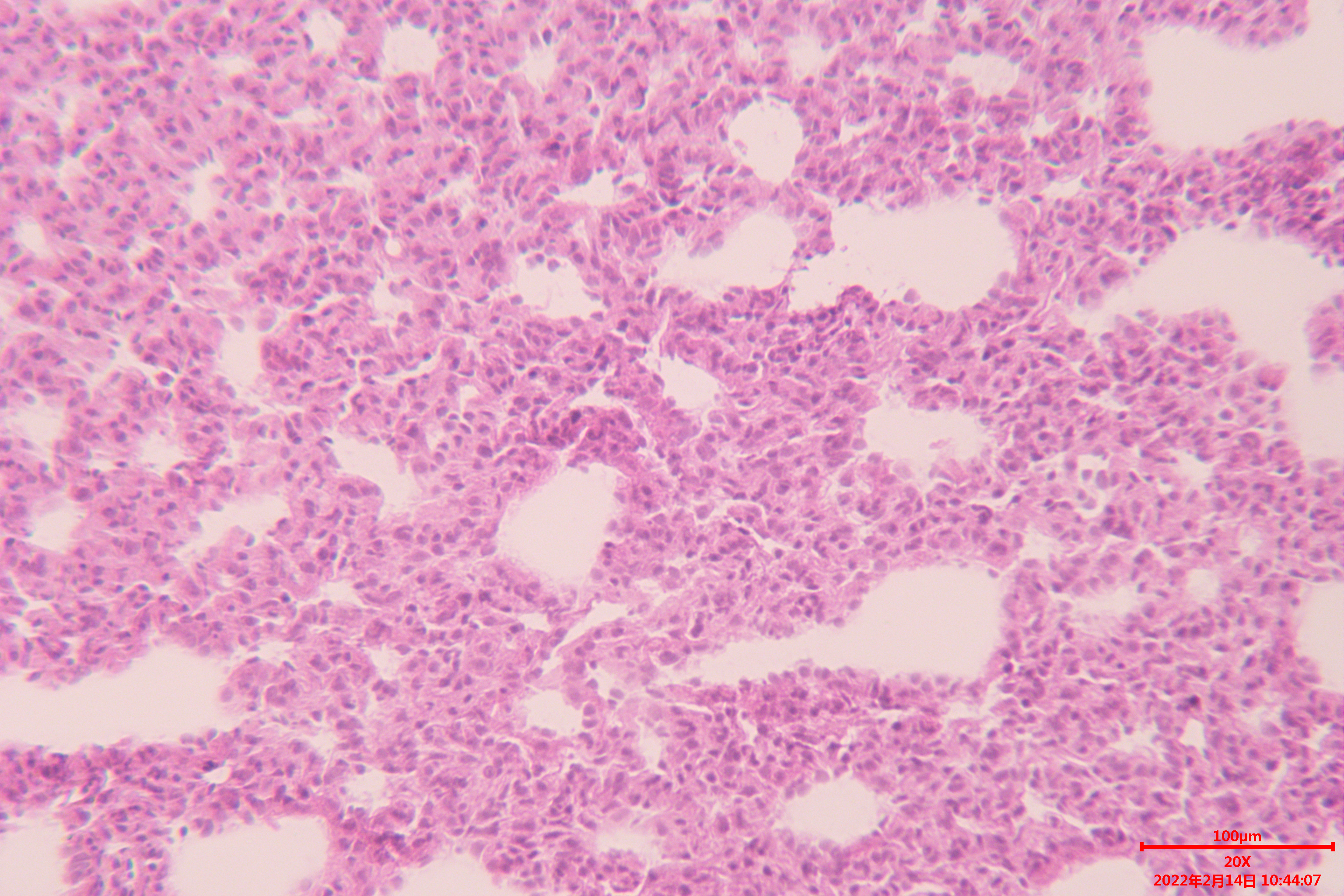

Supplement: Supplementary material — Original Images for Fig S10_1.zip [file IDRD_A_2585599_SM5406.zip › Original Image for Fig S10 G2 (lung).tif]

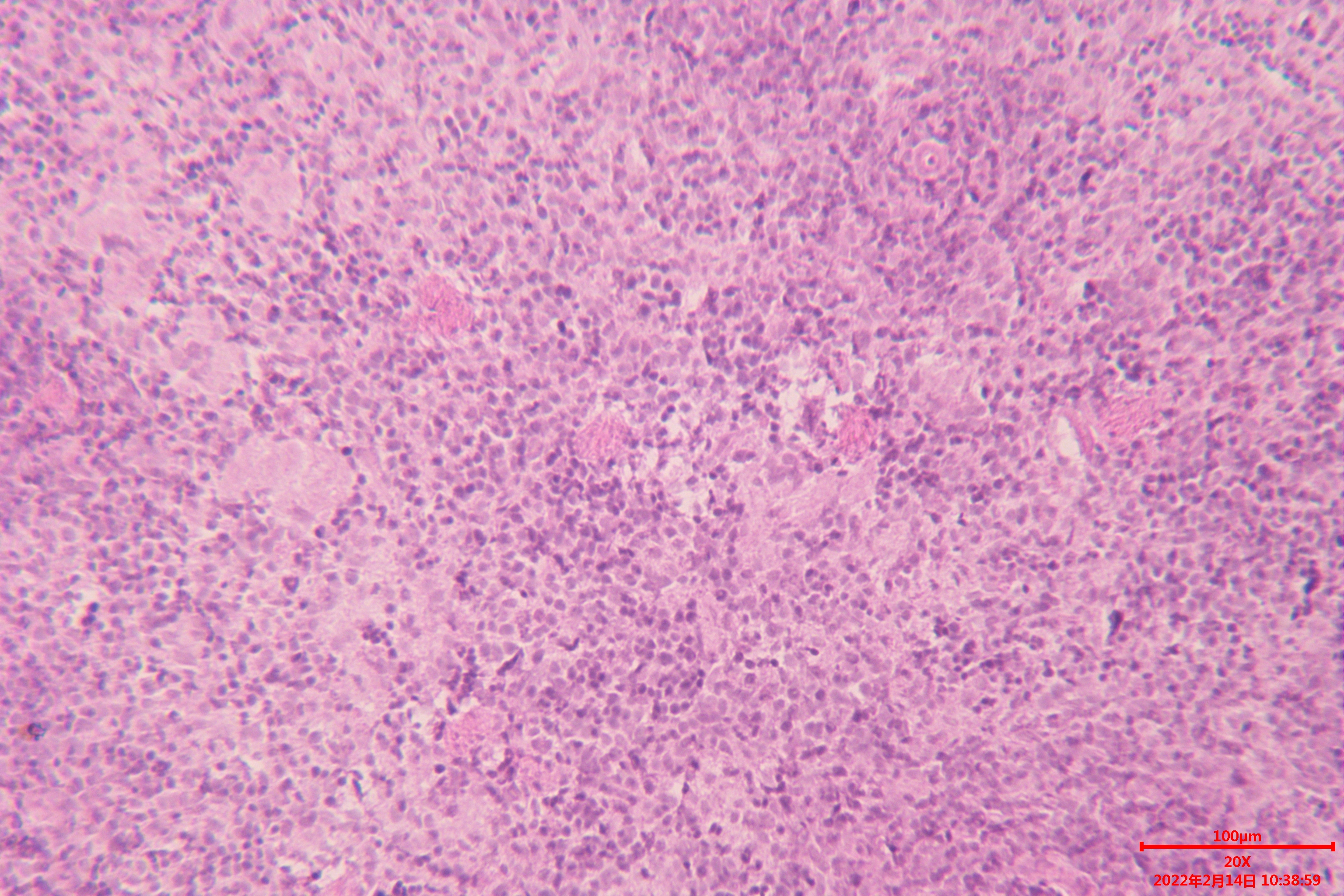

Supplement: Supplementary material — Original Images for Fig S10_1.zip [file IDRD_A_2585599_SM5406.zip › Original Image for Fig S10 G2 (spleen).tif]

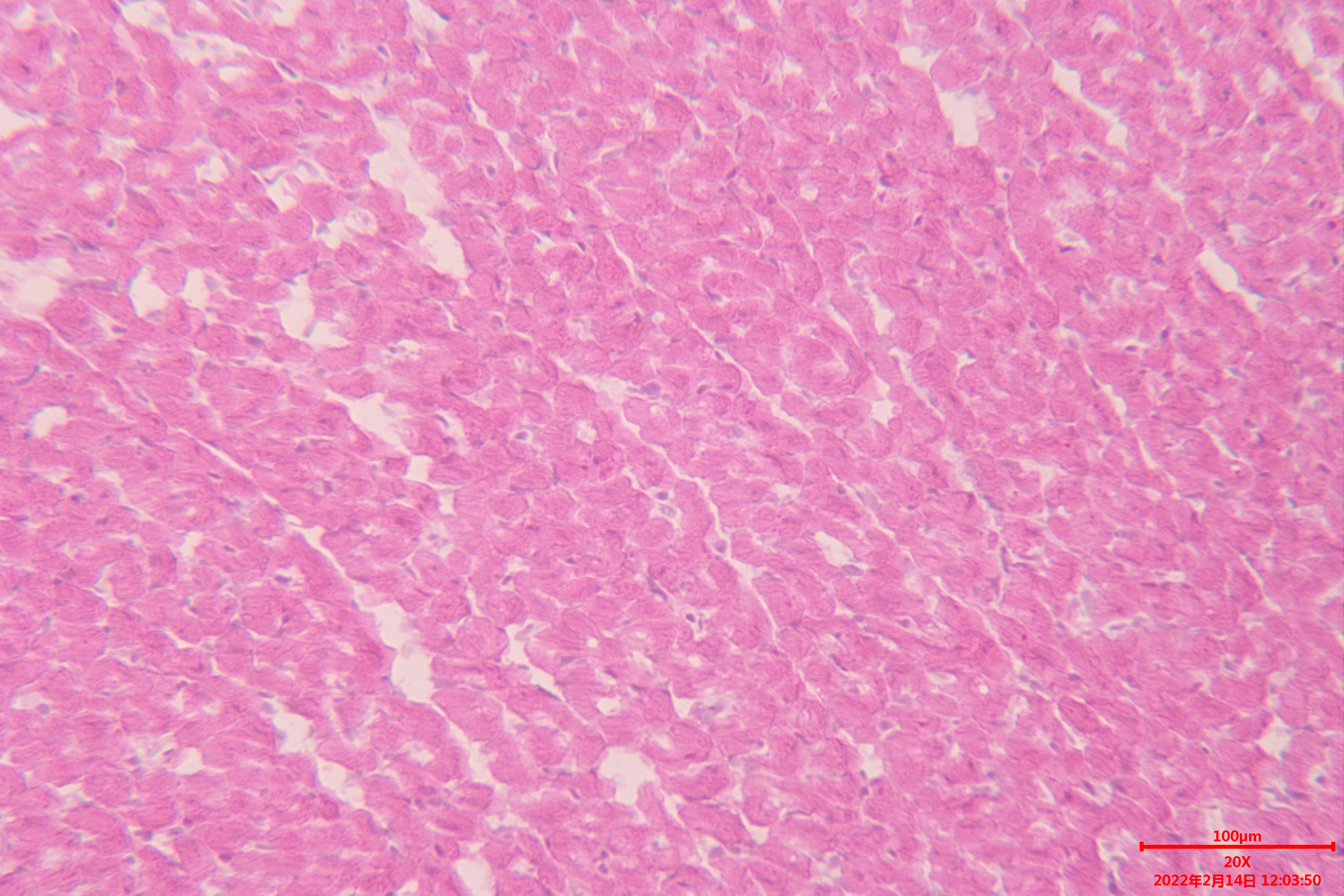

Supplement: Supplementary material — Original Images for Fig S10_4.zip [file IDRD_A_2585599_SM5407.zip › Original Image for Fig S10 G7 (heart).tif]

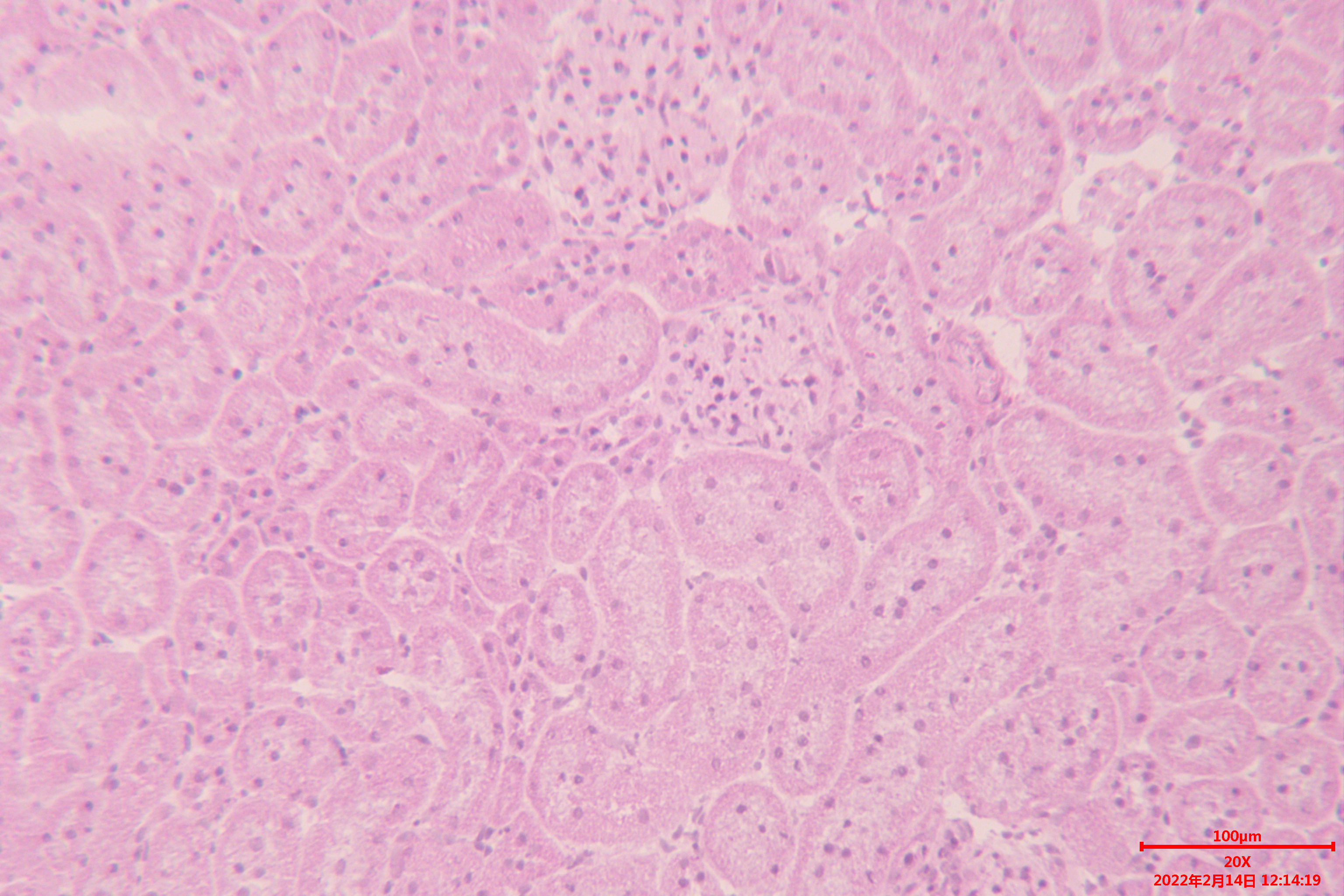

Supplement: Supplementary material — Original Images for Fig S10_4.zip [file IDRD_A_2585599_SM5407.zip › Original Image for Fig S10 G7 (kidney).tif]

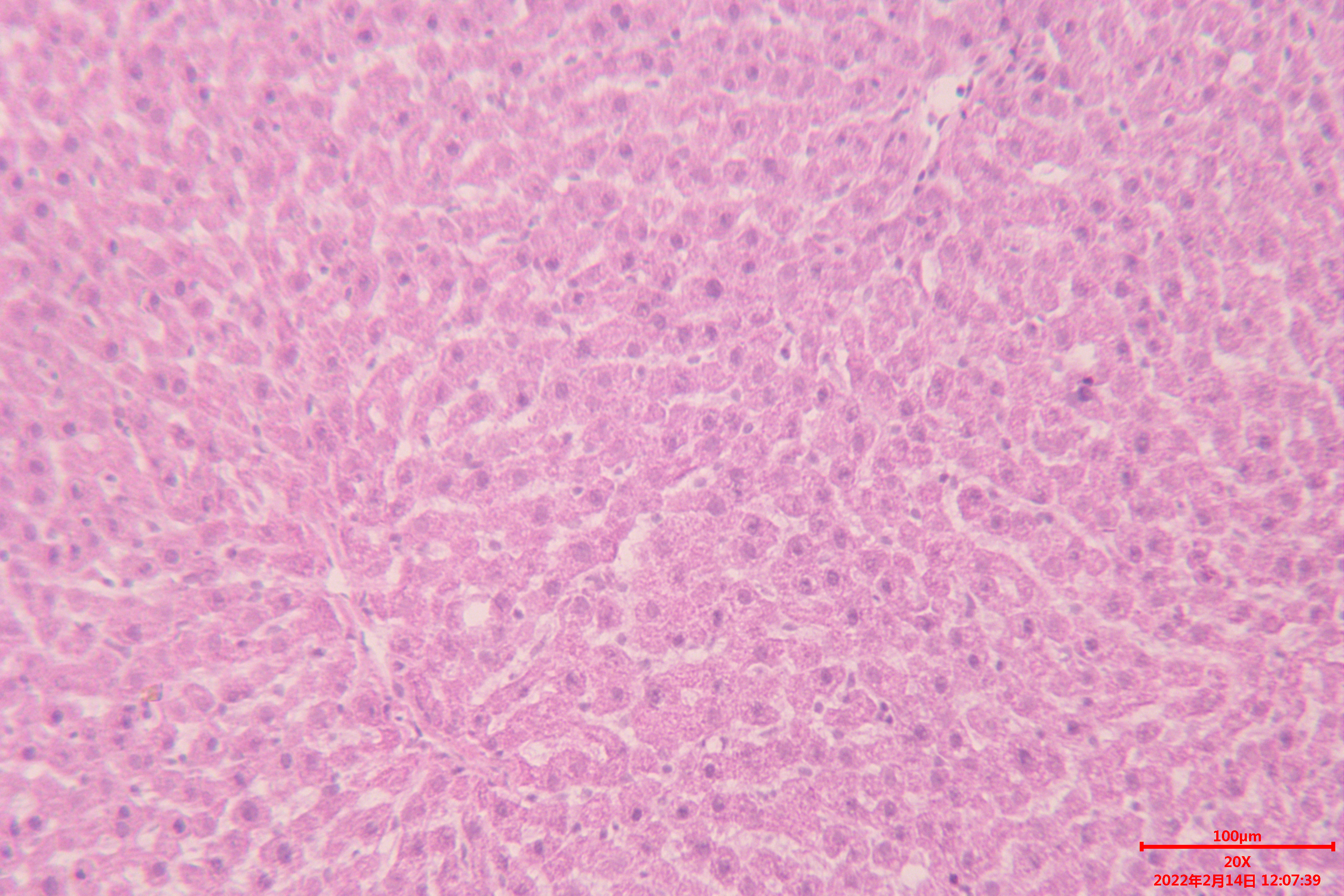

Supplement: Supplementary material — Original Images for Fig S10_4.zip [file IDRD_A_2585599_SM5407.zip › Original Image for Fig S10 G7 (liver).tif]

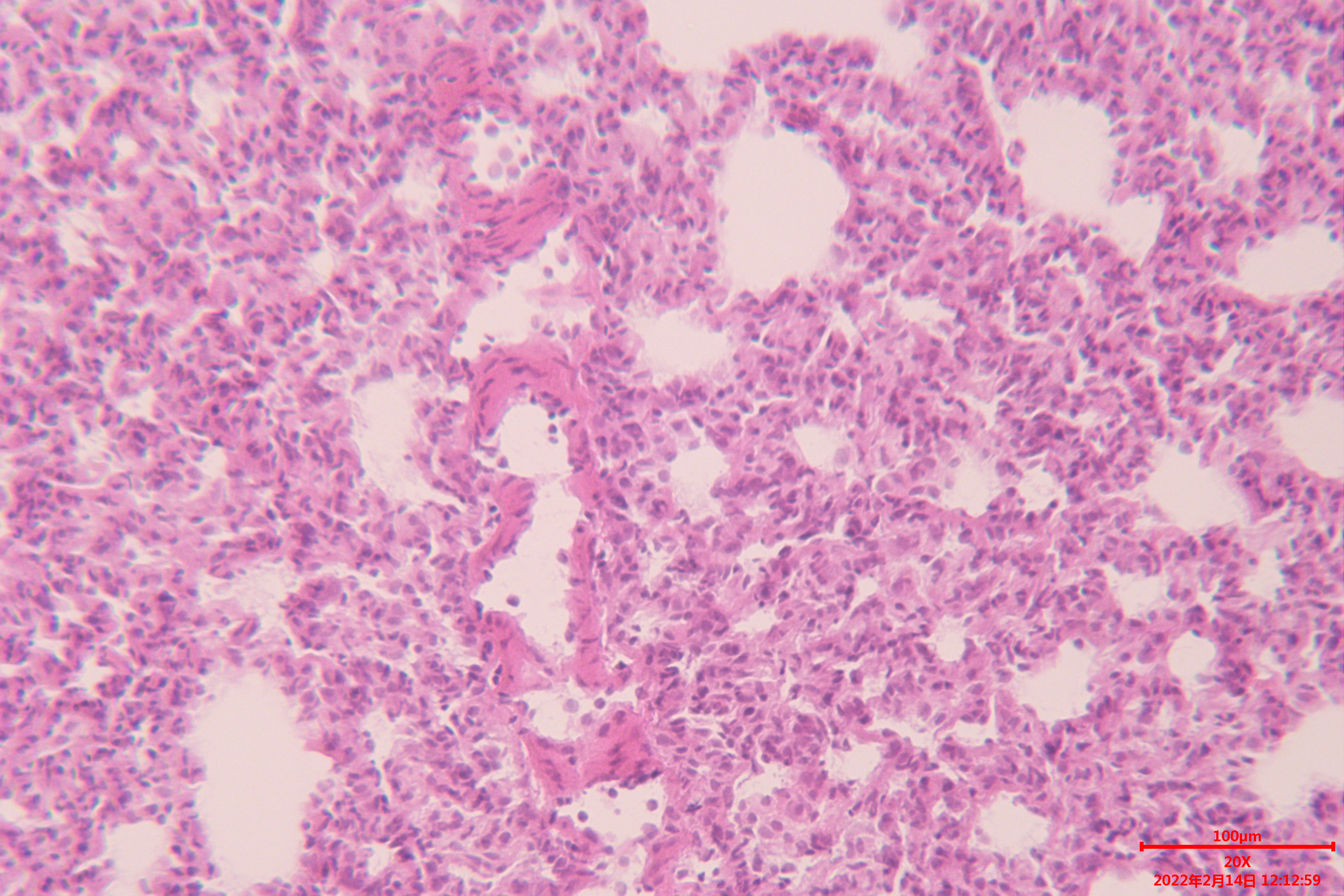

Supplement: Supplementary material — Original Images for Fig S10_4.zip [file IDRD_A_2585599_SM5407.zip › Original Image for Fig S10 G7 (lung).tif]

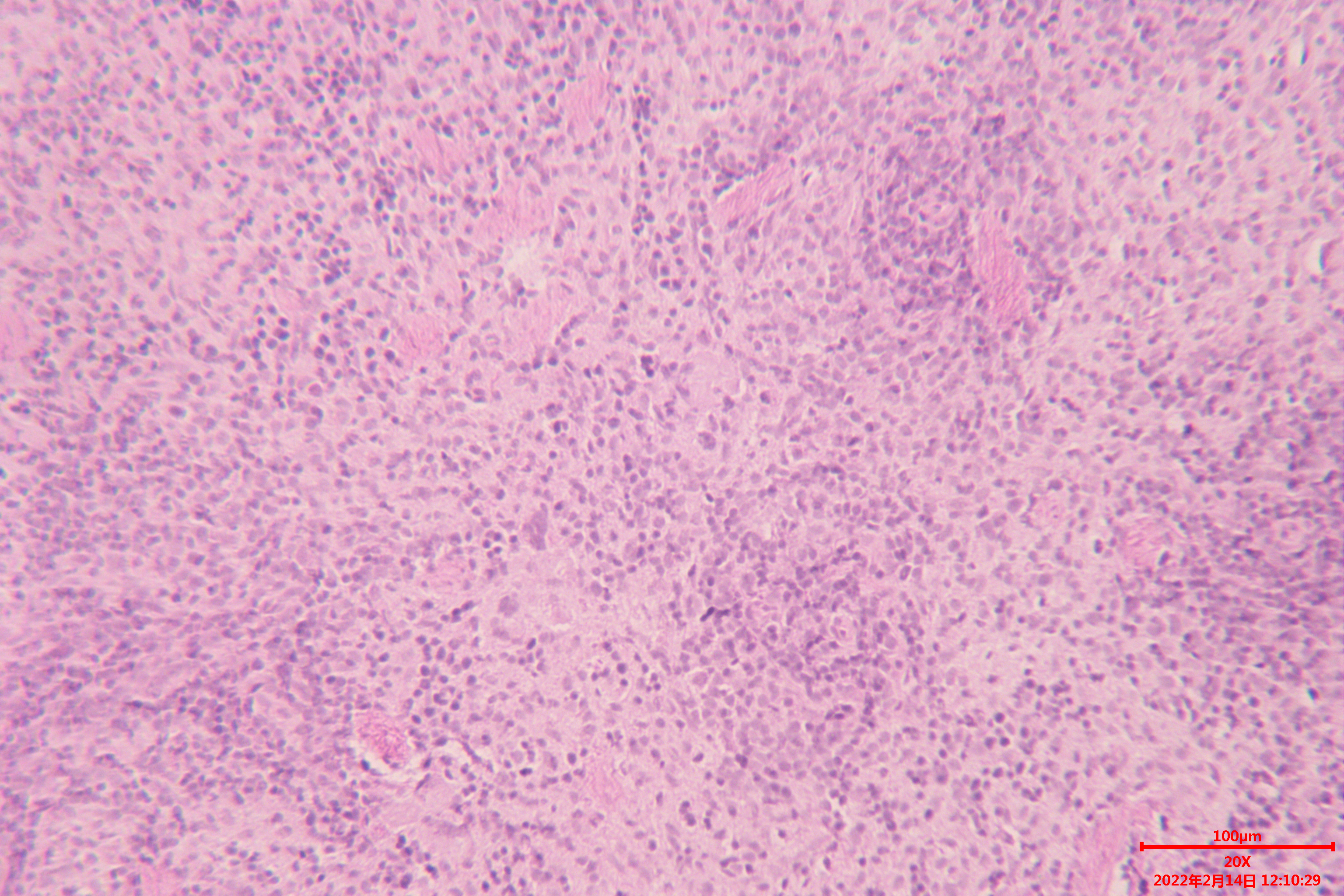

Supplement: Supplementary material — Original Images for Fig S10_4.zip [file IDRD_A_2585599_SM5407.zip › Original Image for Fig S10 G7 (spleen).tif]

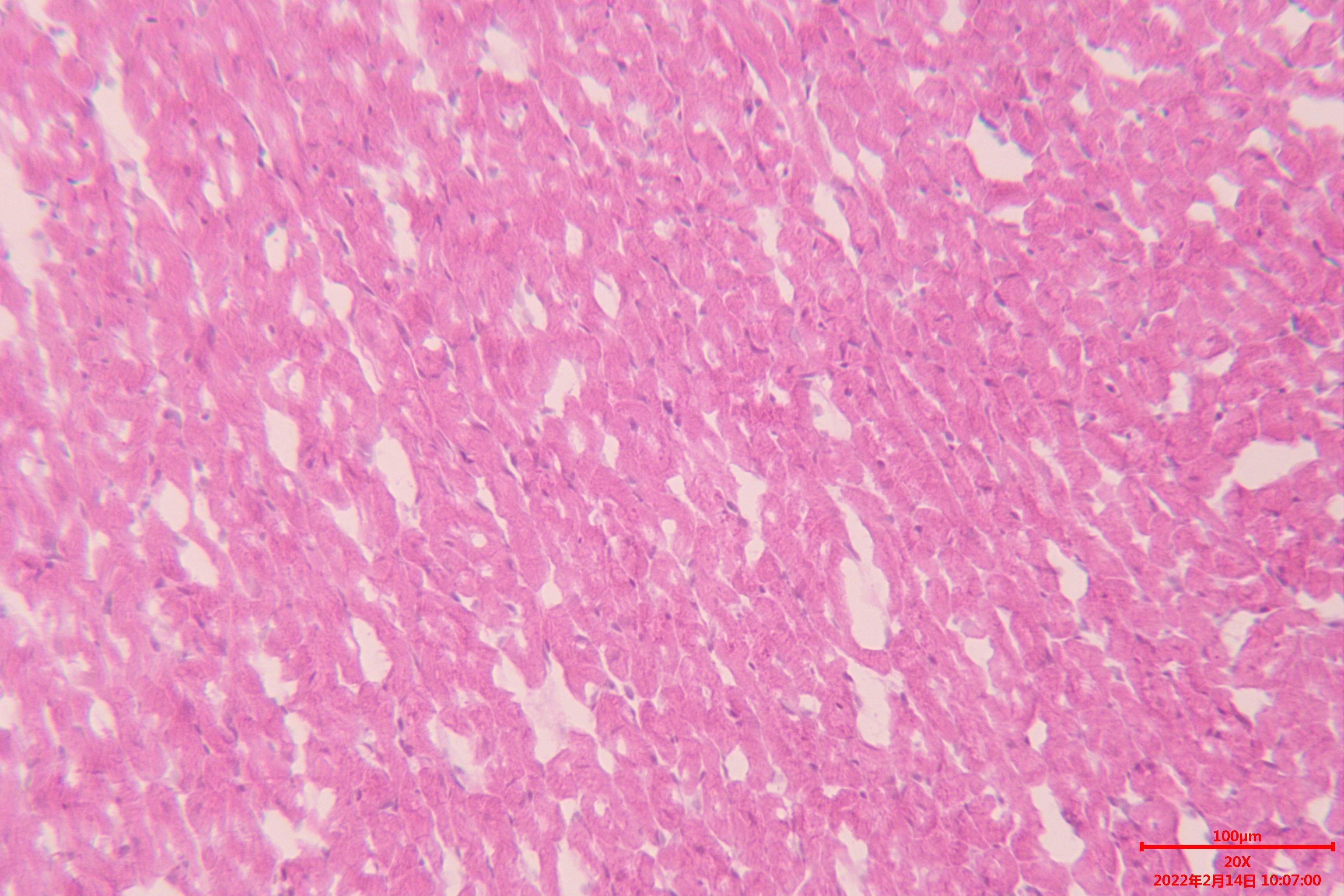

Supplement: Supplementary material — Original Images for Fig S10_4.zip [file IDRD_A_2585599_SM5407.zip › Original Image for Fig S10 Sham (heart).tif]

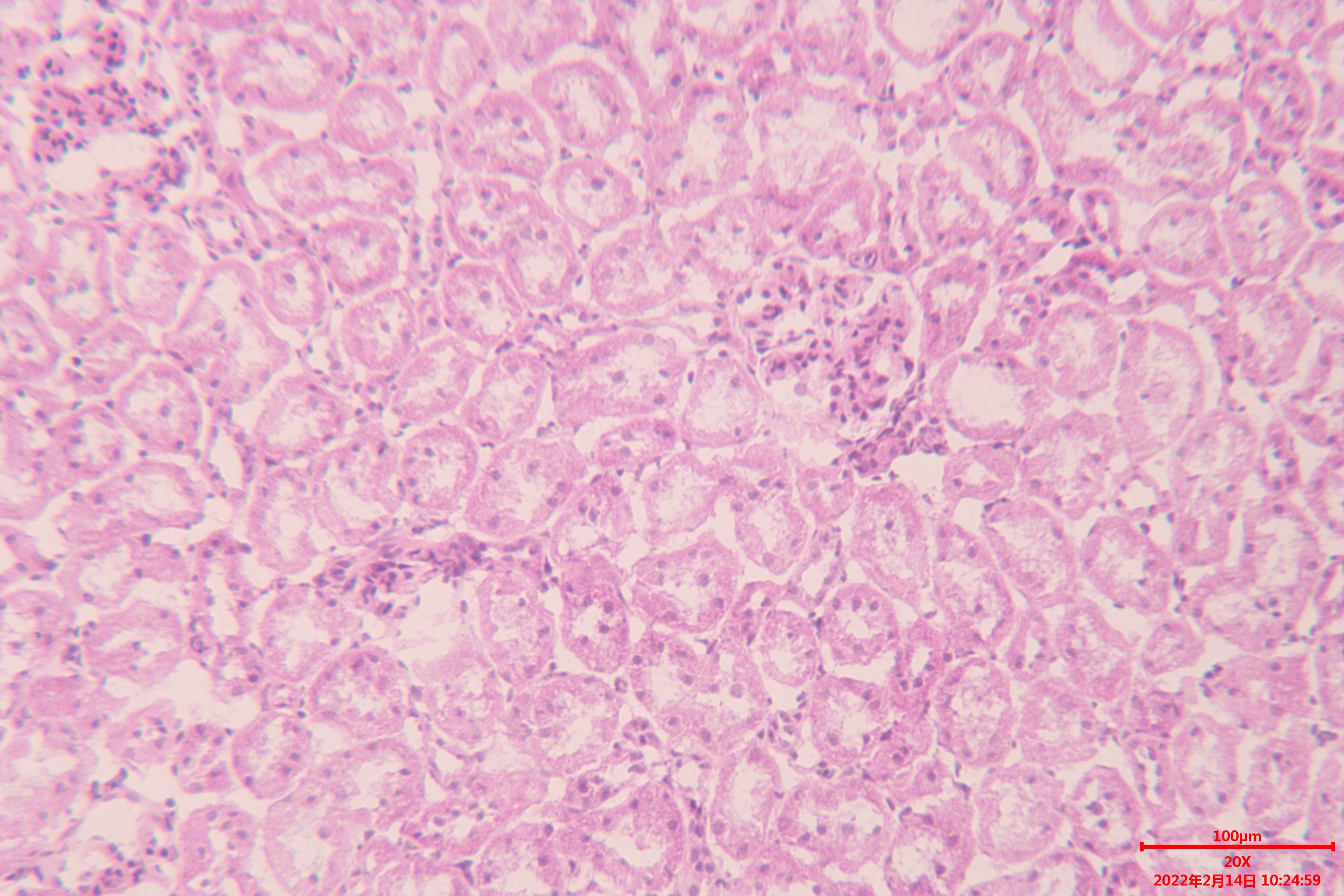

Supplement: Supplementary material — Original Images for Fig S10_4.zip [file IDRD_A_2585599_SM5407.zip › Original Image for Fig S10 Sham (kidney).tif]

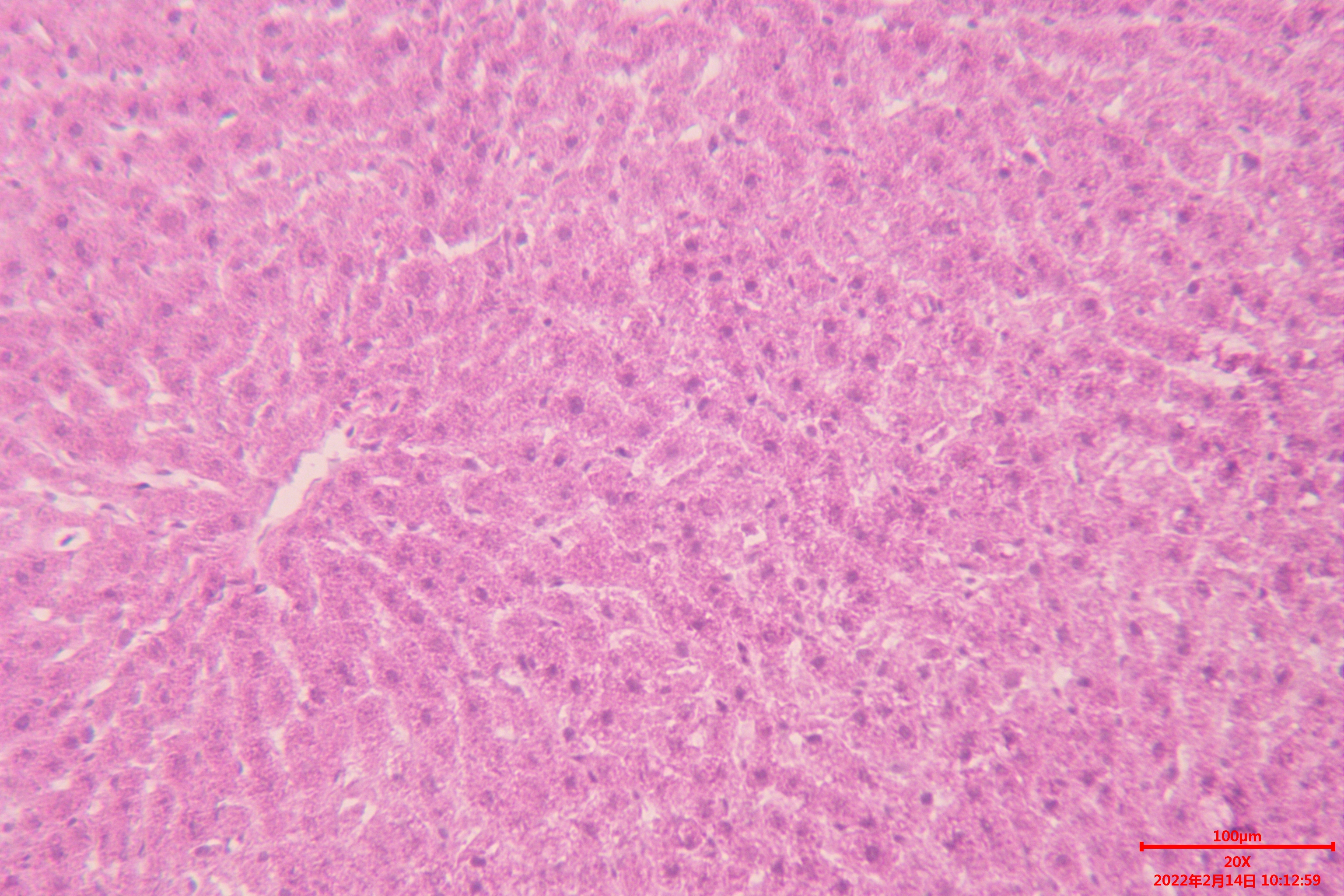

Supplement: Supplementary material — Original Images for Fig S10_4.zip [file IDRD_A_2585599_SM5407.zip › Original Image for Fig S10 Sham (liver).tif]

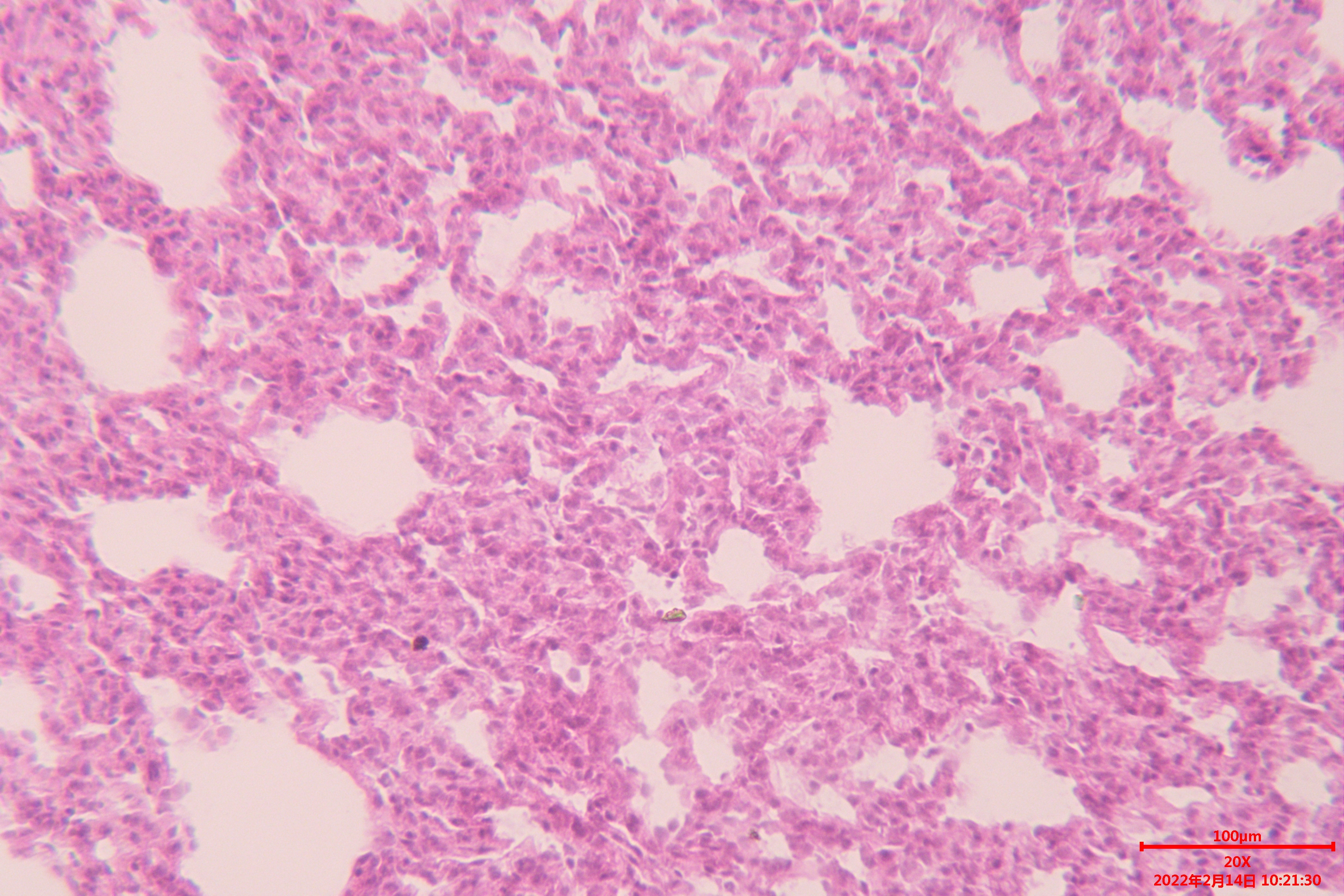

Supplement: Supplementary material — Original Images for Fig S10_4.zip [file IDRD_A_2585599_SM5407.zip › Original Image for Fig S10 Sham (lung).tif]

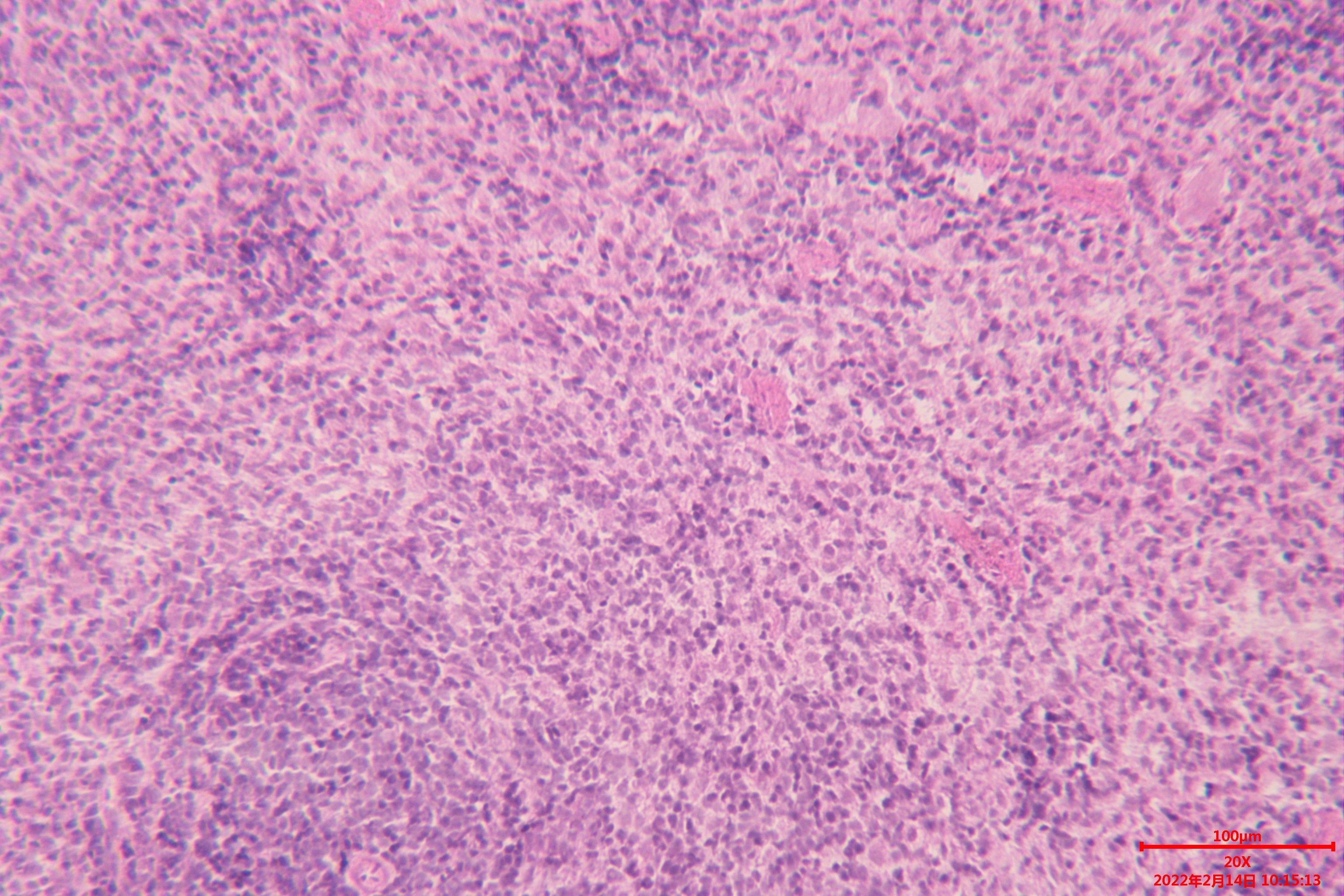

Supplement: Supplementary material — Original Images for Fig S10_4.zip [file IDRD_A_2585599_SM5407.zip › Original Image for Fig S10 Sham (spleen).tif]

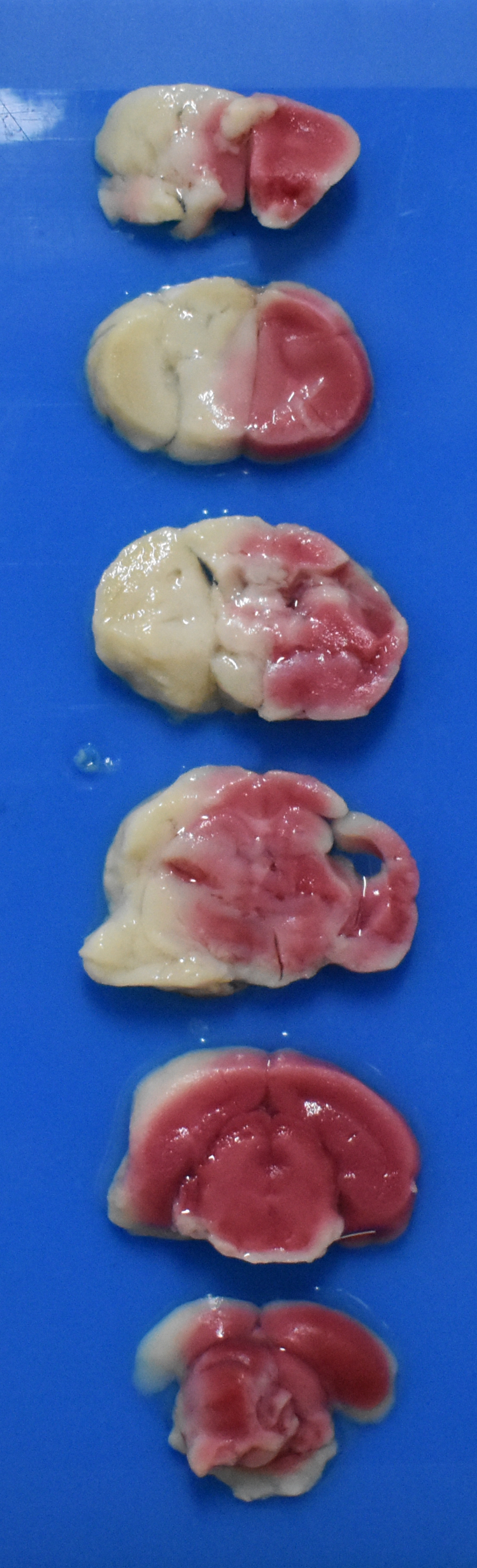

Supplement: Supplementary material — Original Images for Fig 6.zip [file IDRD_A_2585599_SM5409.zip › Original Image for Fig 6B (G1).tif]

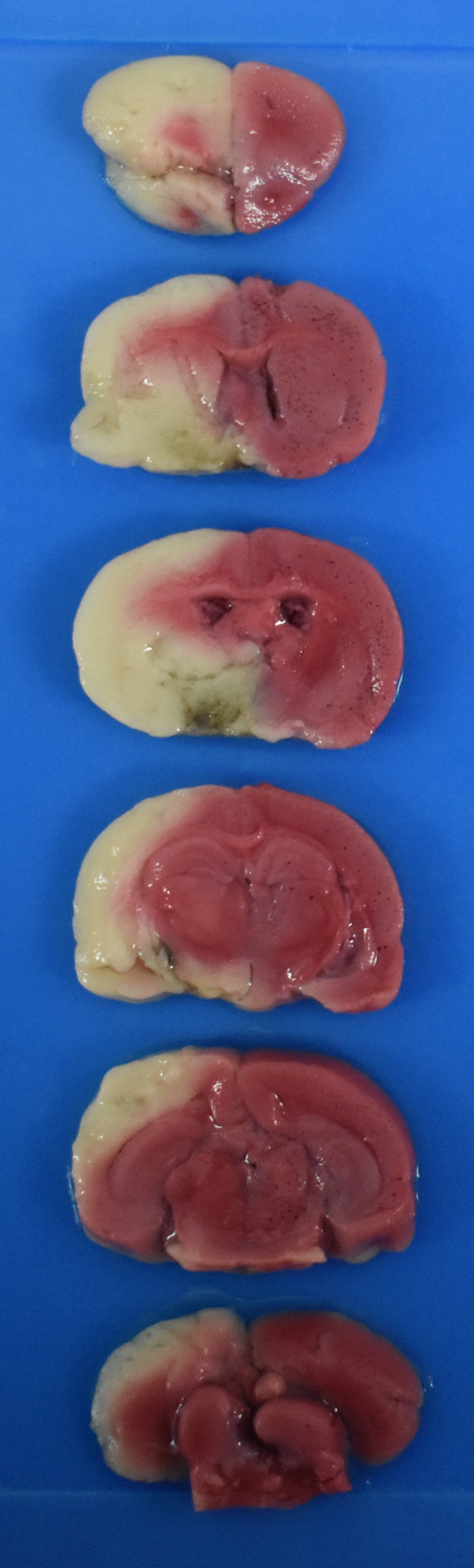

Supplement: Supplementary material — Original Images for Fig 6.zip [file IDRD_A_2585599_SM5409.zip › Original Image for Fig 6B (G2).tif]
