# Supplementary figures and images for: Targeted strategy by curcumin and tideglusib biomimetic nano-systems alleviates oxidative stress and inflammation under ischemic stroke (part 3 of 3)
Source: Drug Deliv. 2025 Nov 25;32(1):2585599. doi: 10.1080/10717544.2025.2585599 (PMC12667299; doi:10.1080/10717544.2025.2585599)

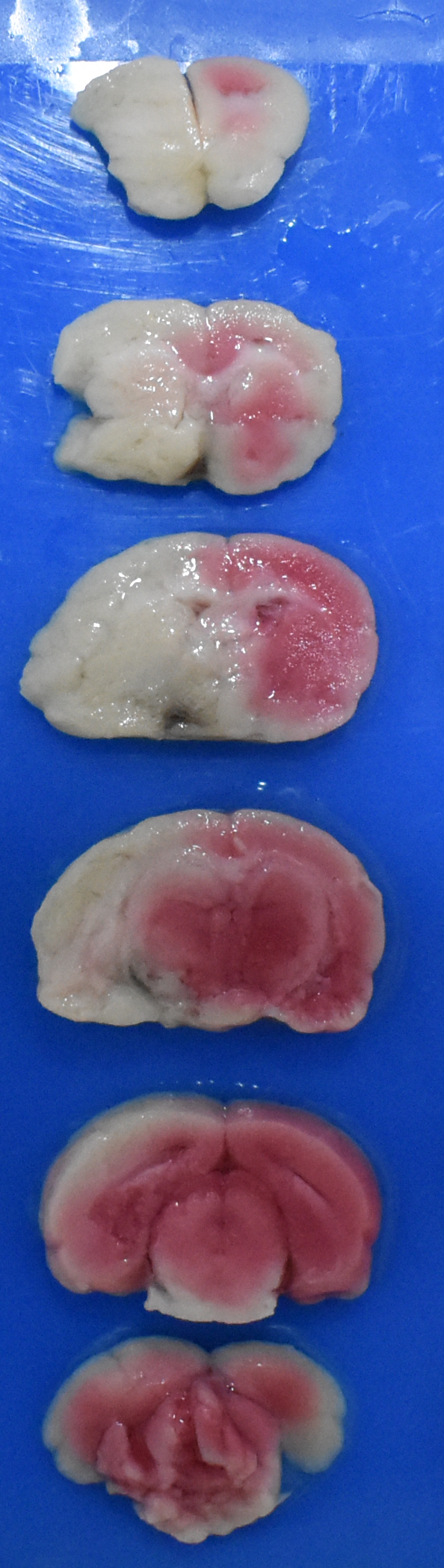

Supplement: Supplementary material — Original Images for Fig 6.zip [file IDRD_A_2585599_SM5409.zip › Original Image for Fig 6B (G3).tif]

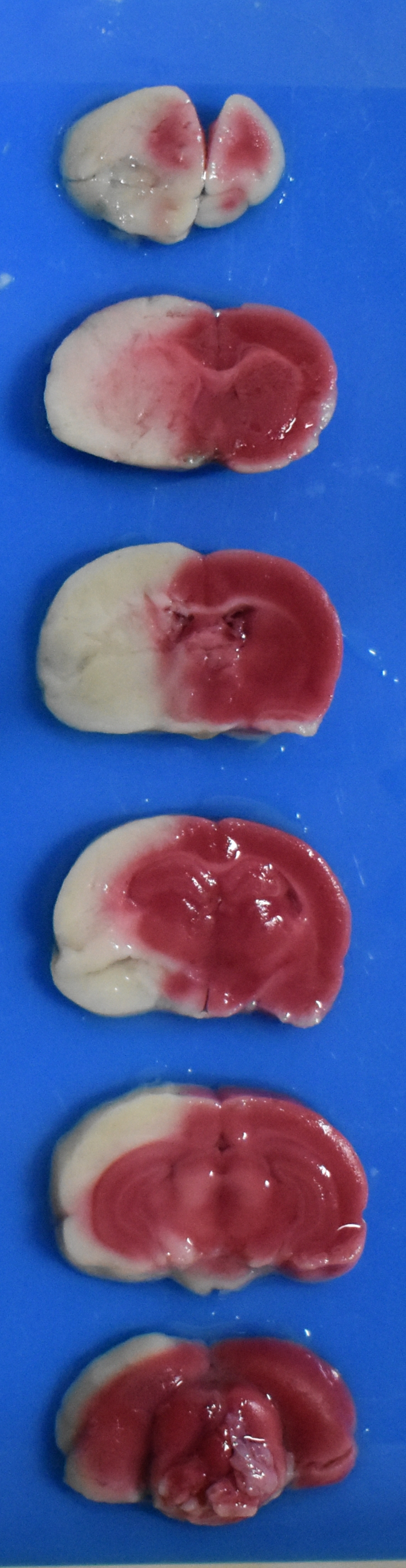

Supplement: Supplementary material — Original Images for Fig 6.zip [file IDRD_A_2585599_SM5409.zip › Original Image for Fig 6B (G4).tif]

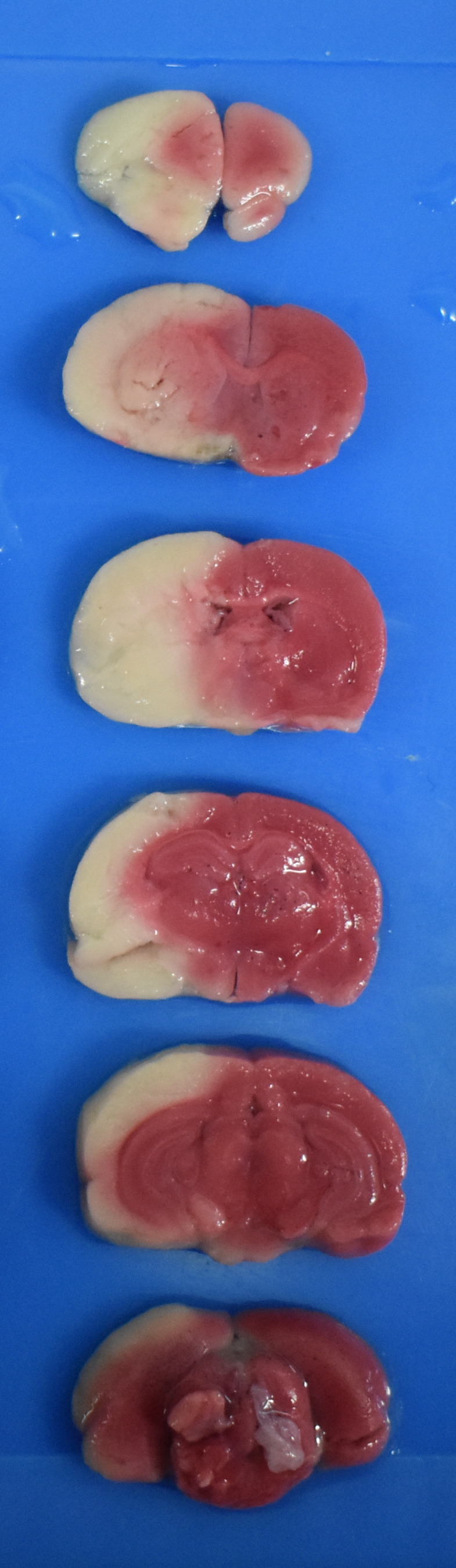

Supplement: Supplementary material — Original Images for Fig 6.zip [file IDRD_A_2585599_SM5409.zip › Original Image for Fig 6B (G5).tif]

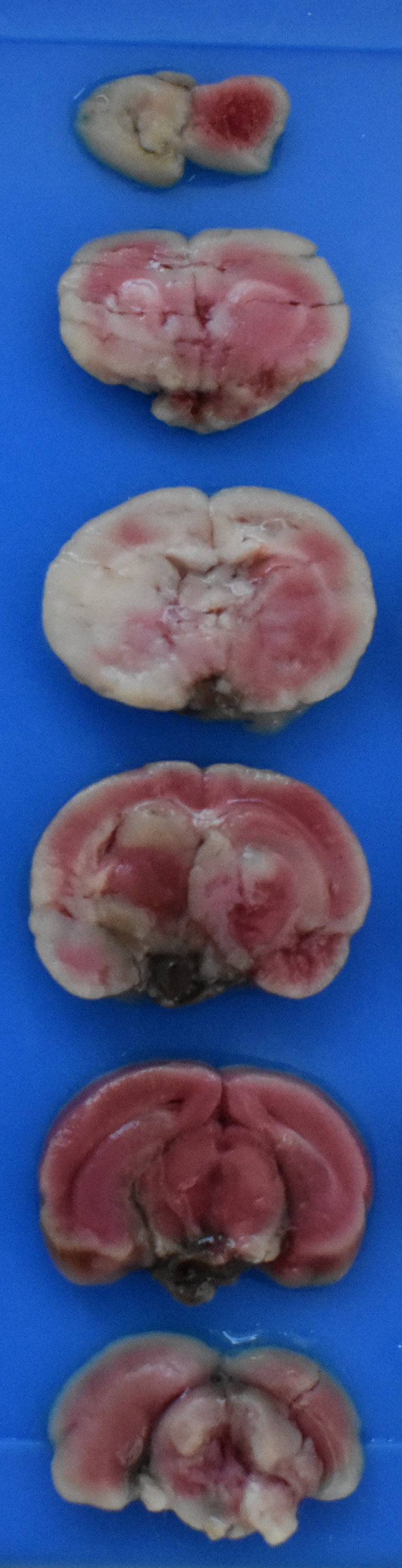

Supplement: Supplementary material — Original Images for Fig 6.zip [file IDRD_A_2585599_SM5409.zip › Original Image for Fig 6B (G6).tif]

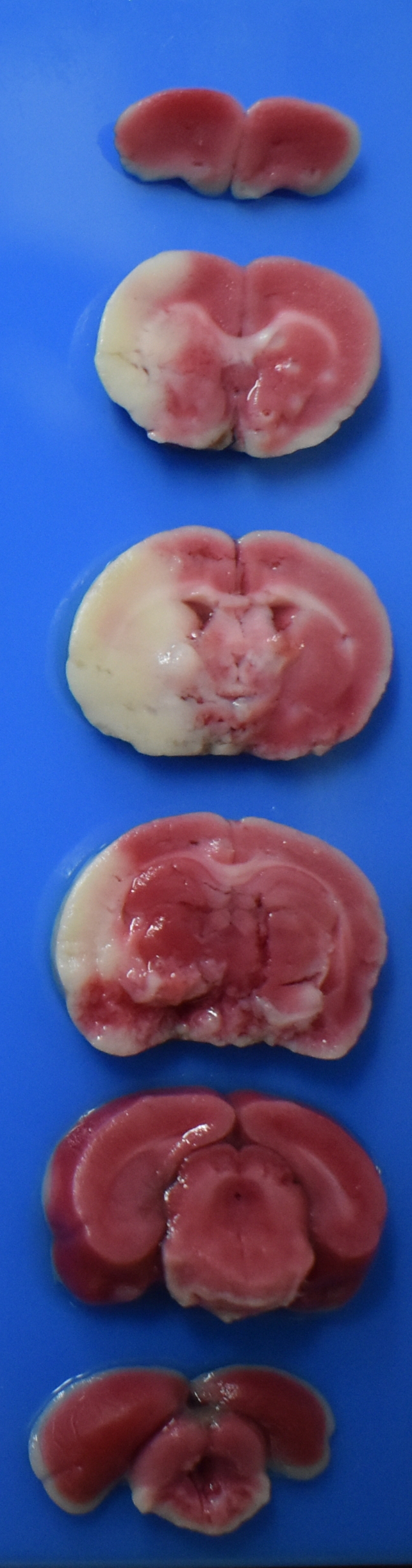

Supplement: Supplementary material — Original Images for Fig 6.zip [file IDRD_A_2585599_SM5409.zip › Original Image for Fig 6B (G7).tif]

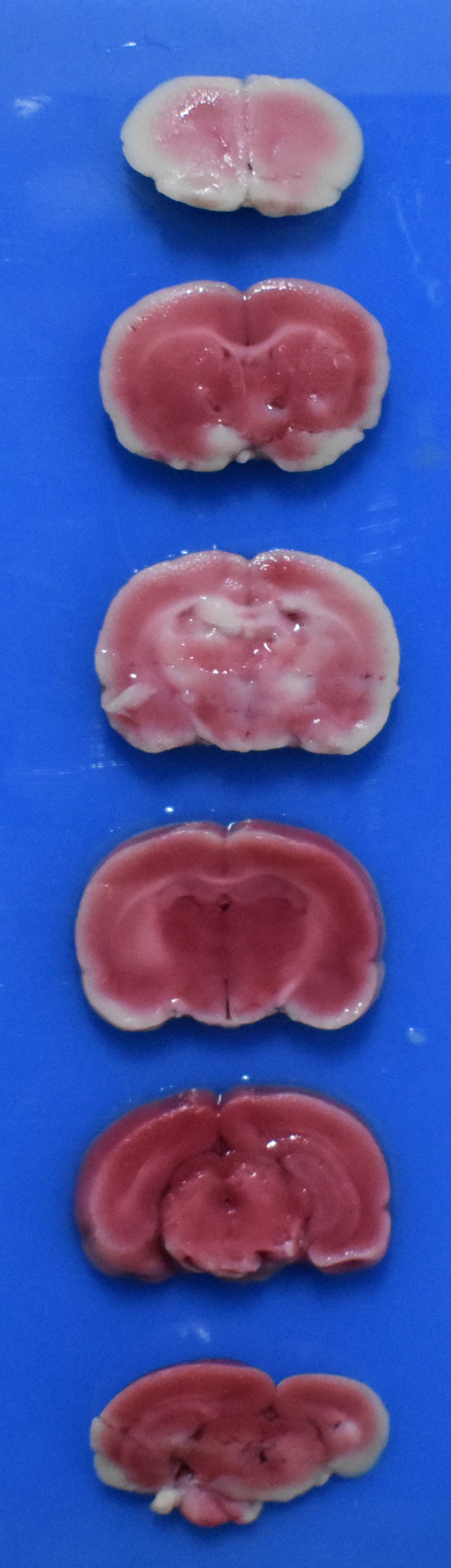

Supplement: Supplementary material — Original Images for Fig 6.zip [file IDRD_A_2585599_SM5409.zip › Original Image for Fig 6B (Sham).tif]

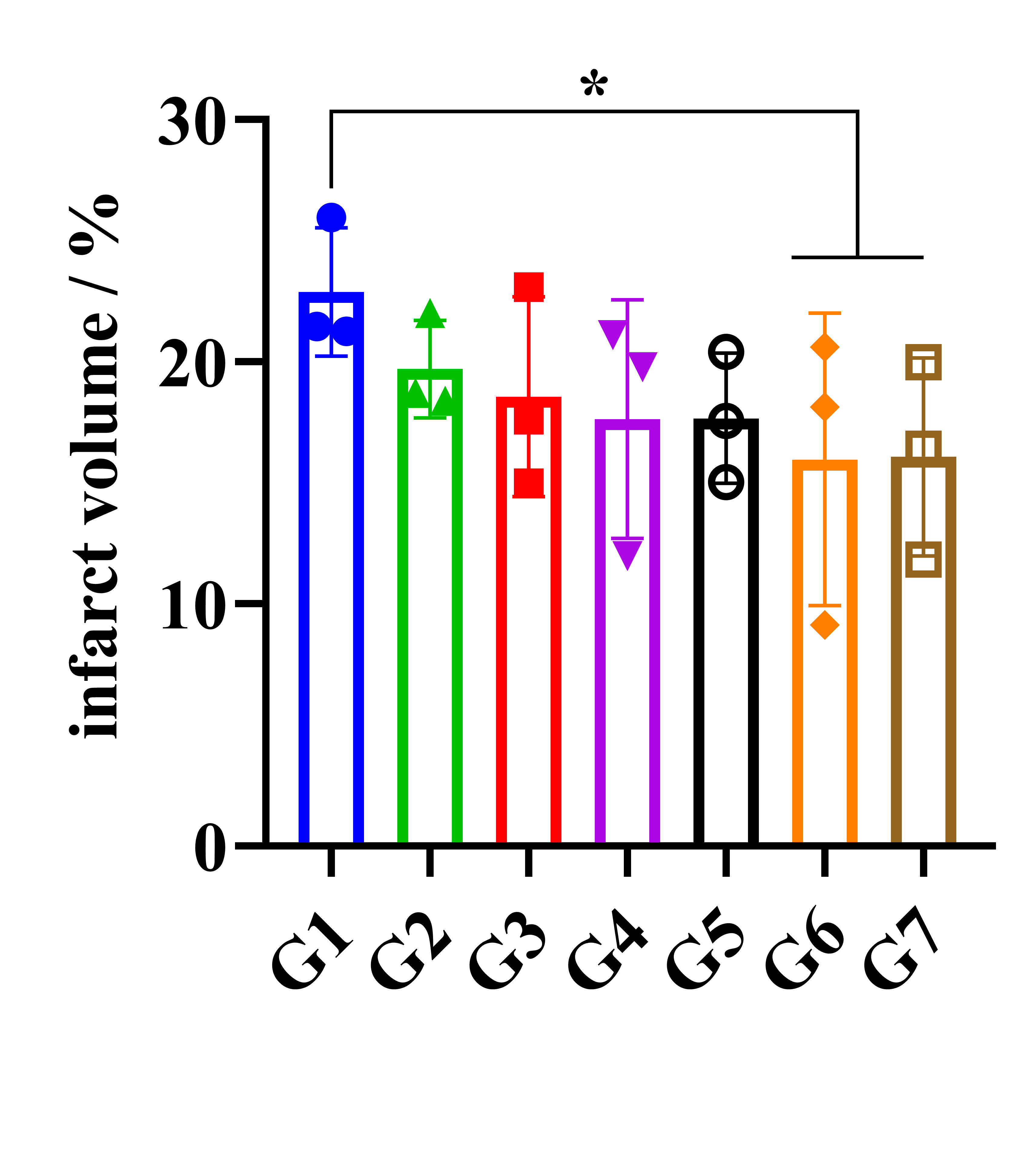

Supplement: Supplementary material — Original Images for Fig 6.zip [file IDRD_A_2585599_SM5409.zip › Original Image for Fig 6C.tif]

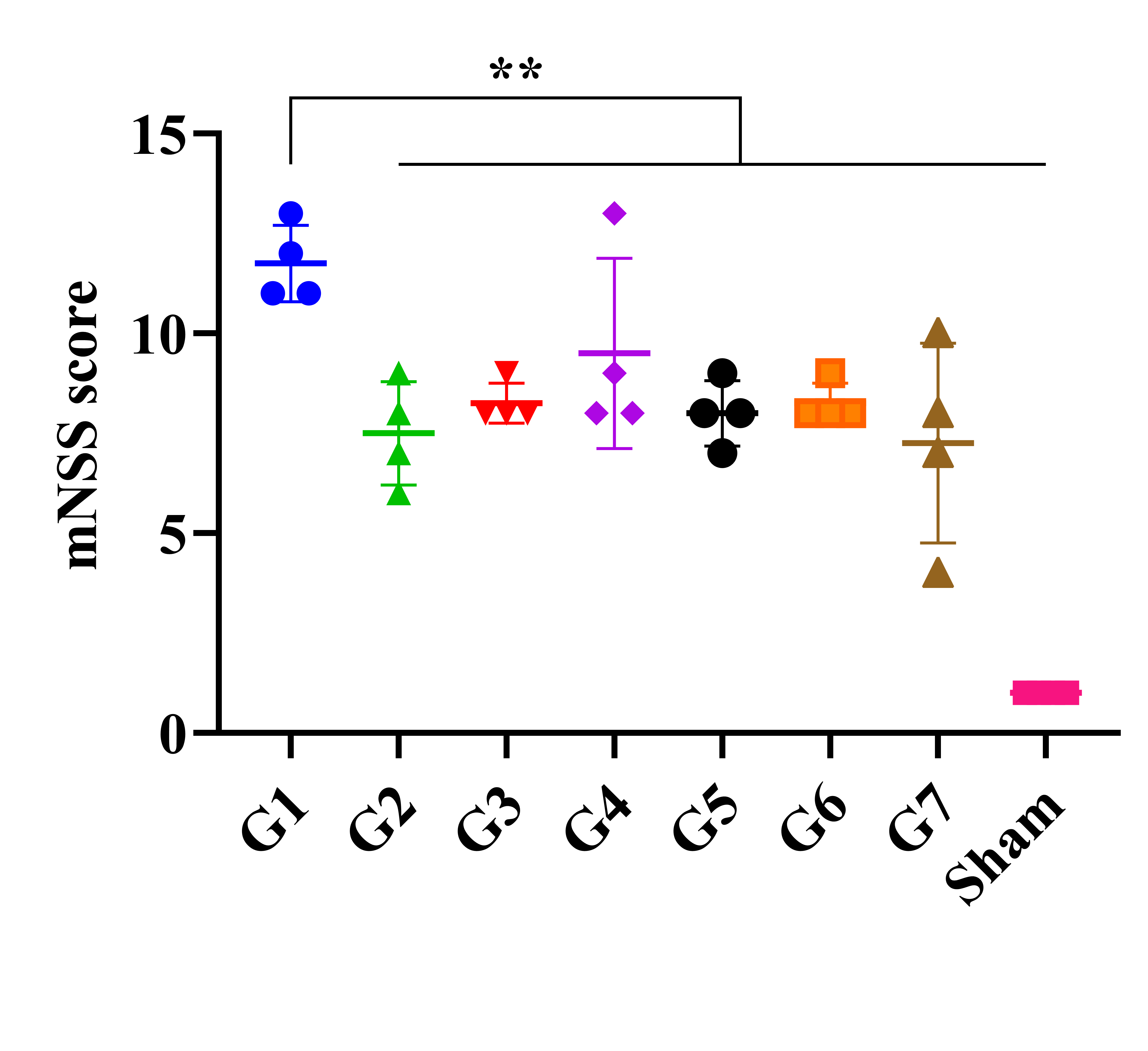

Supplement: Supplementary material — Original Images for Fig 6.zip [file IDRD_A_2585599_SM5409.zip › Original Image for Fig 6D.tif]

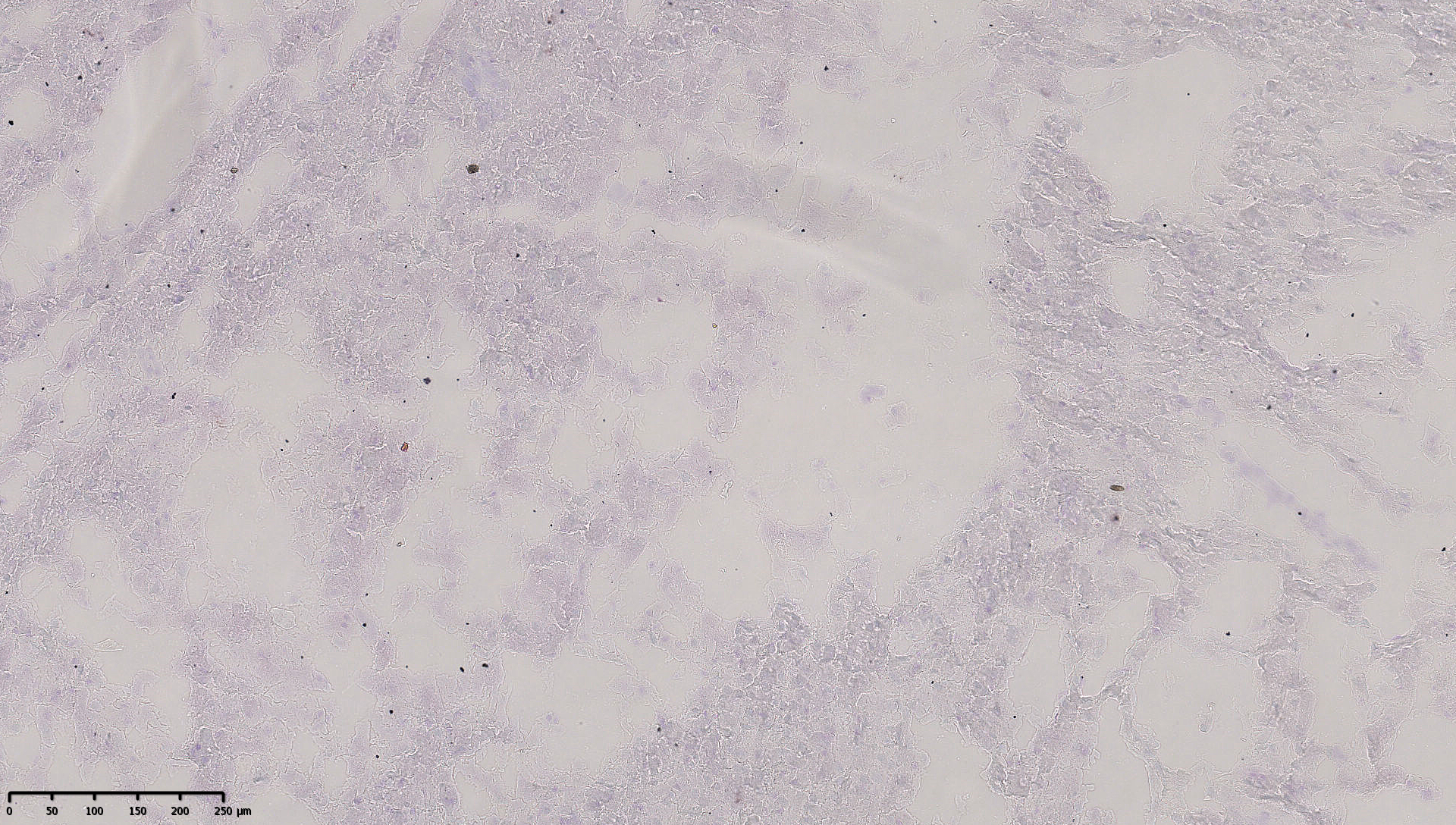

Supplement: Supplementary material — Original Images for Fig 6.zip [file IDRD_A_2585599_SM5409.zip › Original Image for Fig 6E (G1-left).tif]

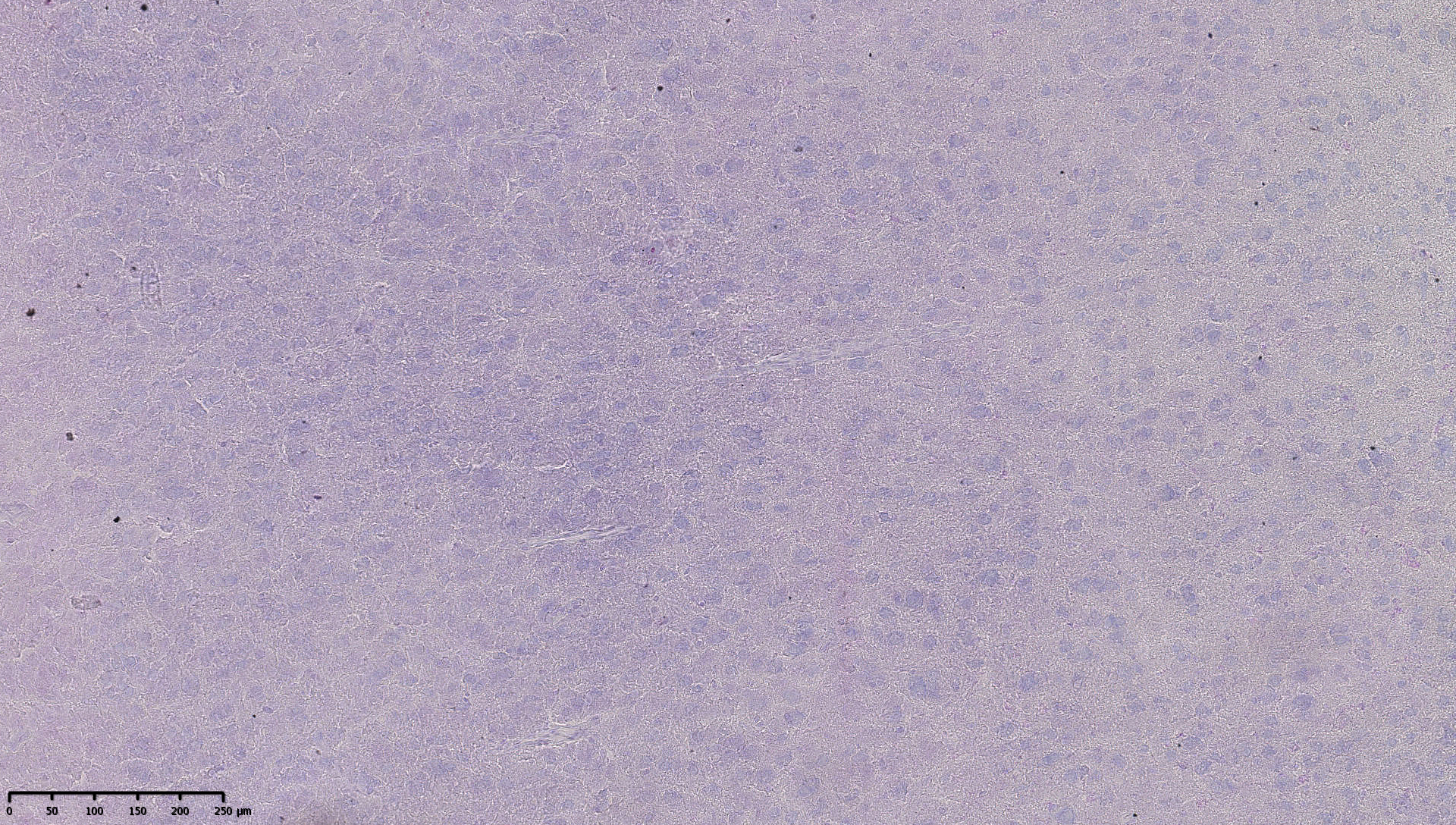

Supplement: Supplementary material — Original Images for Fig 6.zip [file IDRD_A_2585599_SM5409.zip › Original Image for Fig 6E (G1-right).tif]

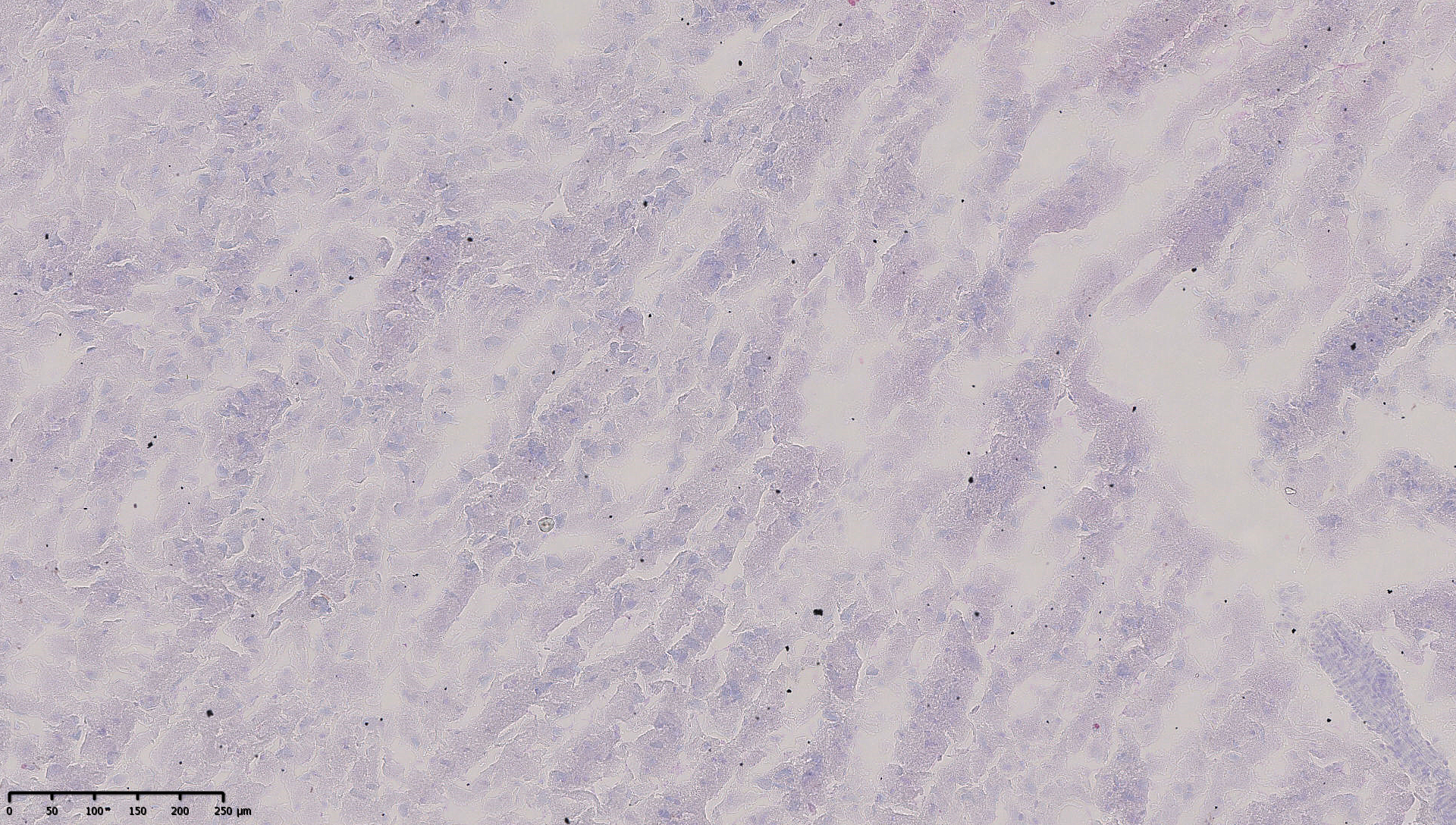

Supplement: Supplementary material — Original Images for Fig 6.zip [file IDRD_A_2585599_SM5409.zip › Original Image for Fig 6E (G2-left).tif]

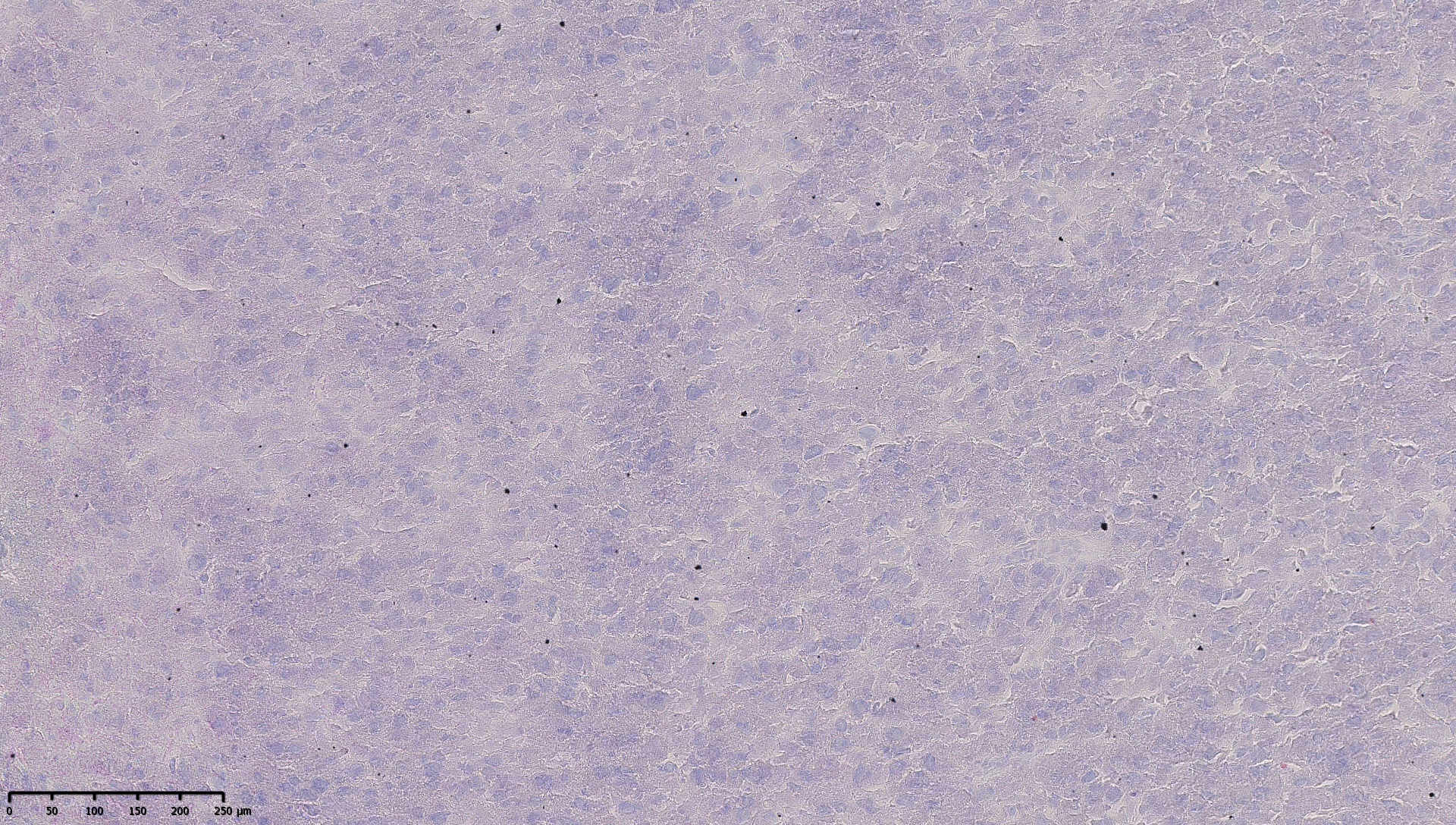

Supplement: Supplementary material — Original Images for Fig 6.zip [file IDRD_A_2585599_SM5409.zip › Original Image for Fig 6E (G2-right).tif]

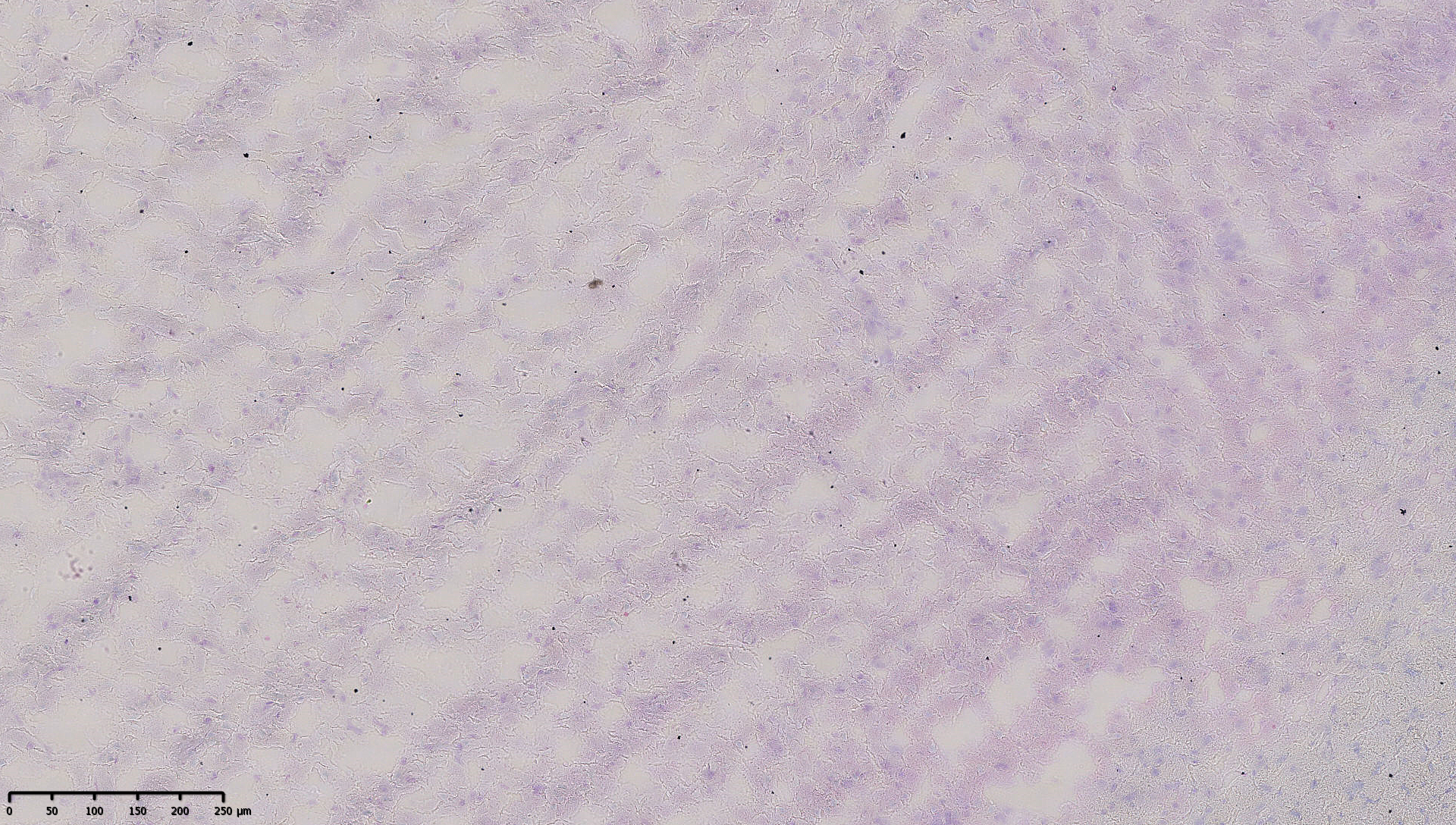

Supplement: Supplementary material — Original Images for Fig 6.zip [file IDRD_A_2585599_SM5409.zip › Original Image for Fig 6E (G3-left).tif]

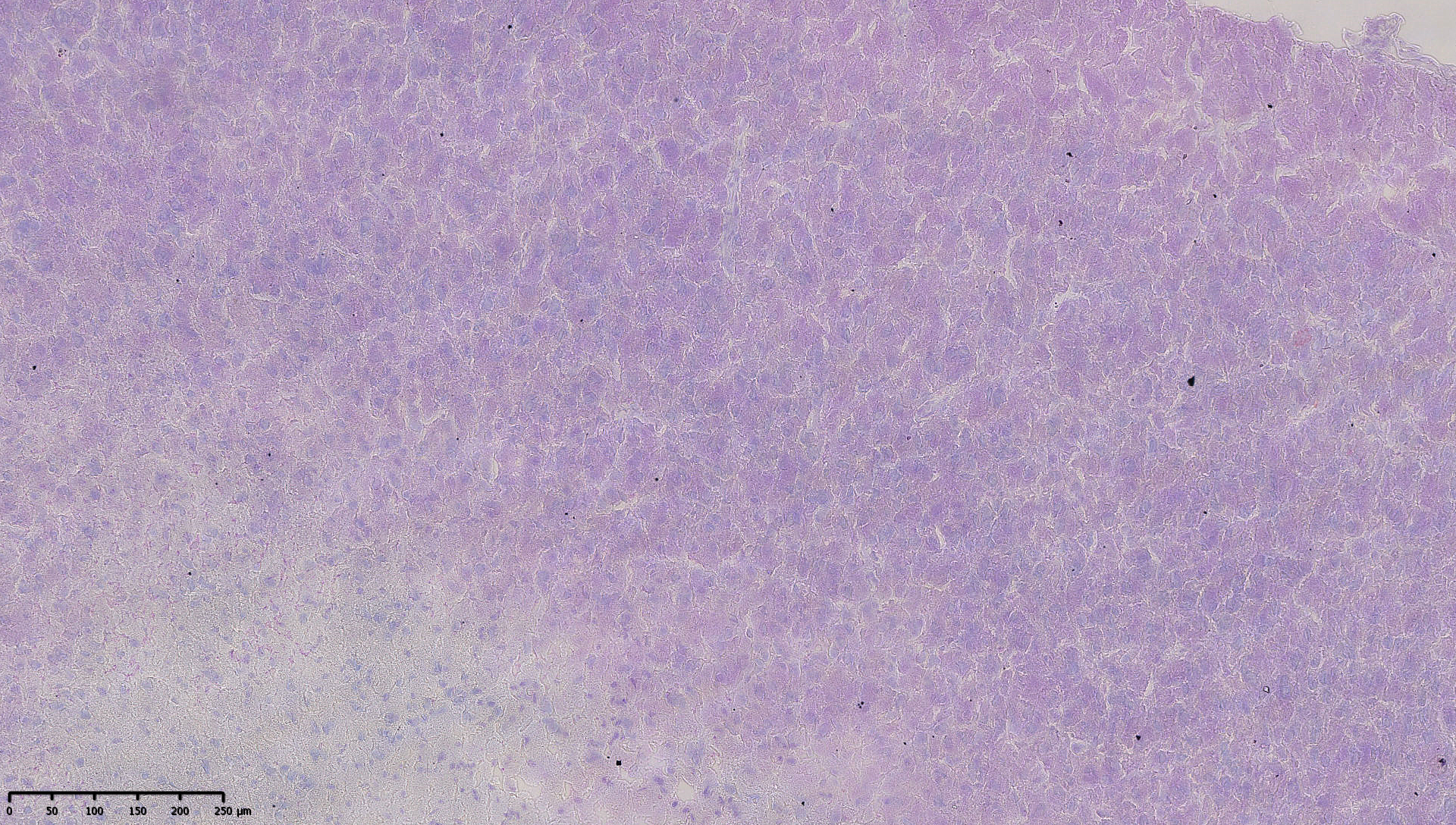

Supplement: Supplementary material — Original Images for Fig 6.zip [file IDRD_A_2585599_SM5409.zip › Original Image for Fig 6E (G3-right).tif]

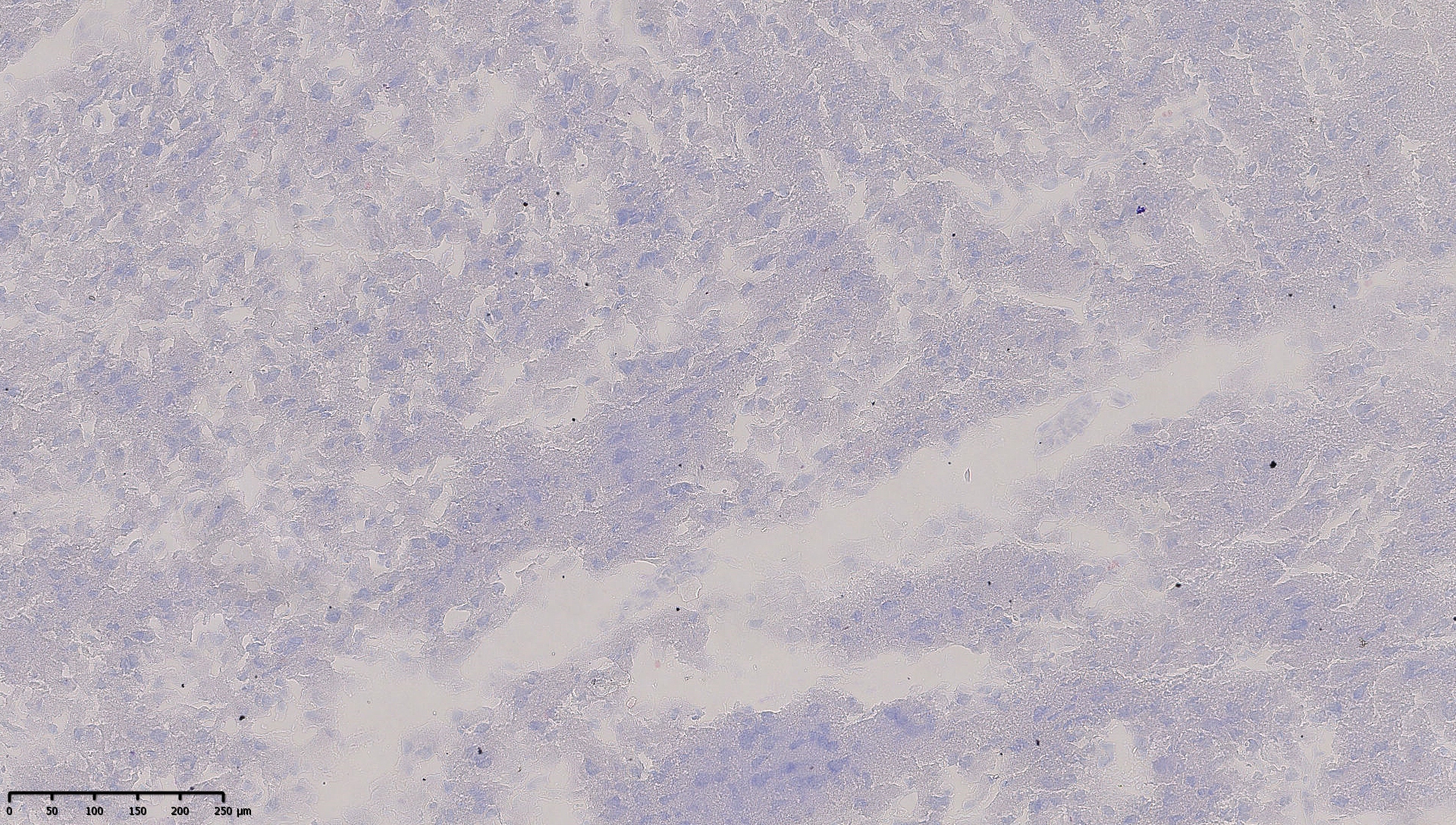

Supplement: Supplementary material — Original Images for Fig 6.zip [file IDRD_A_2585599_SM5409.zip › Original Image for Fig 6E (G4-left).tif]

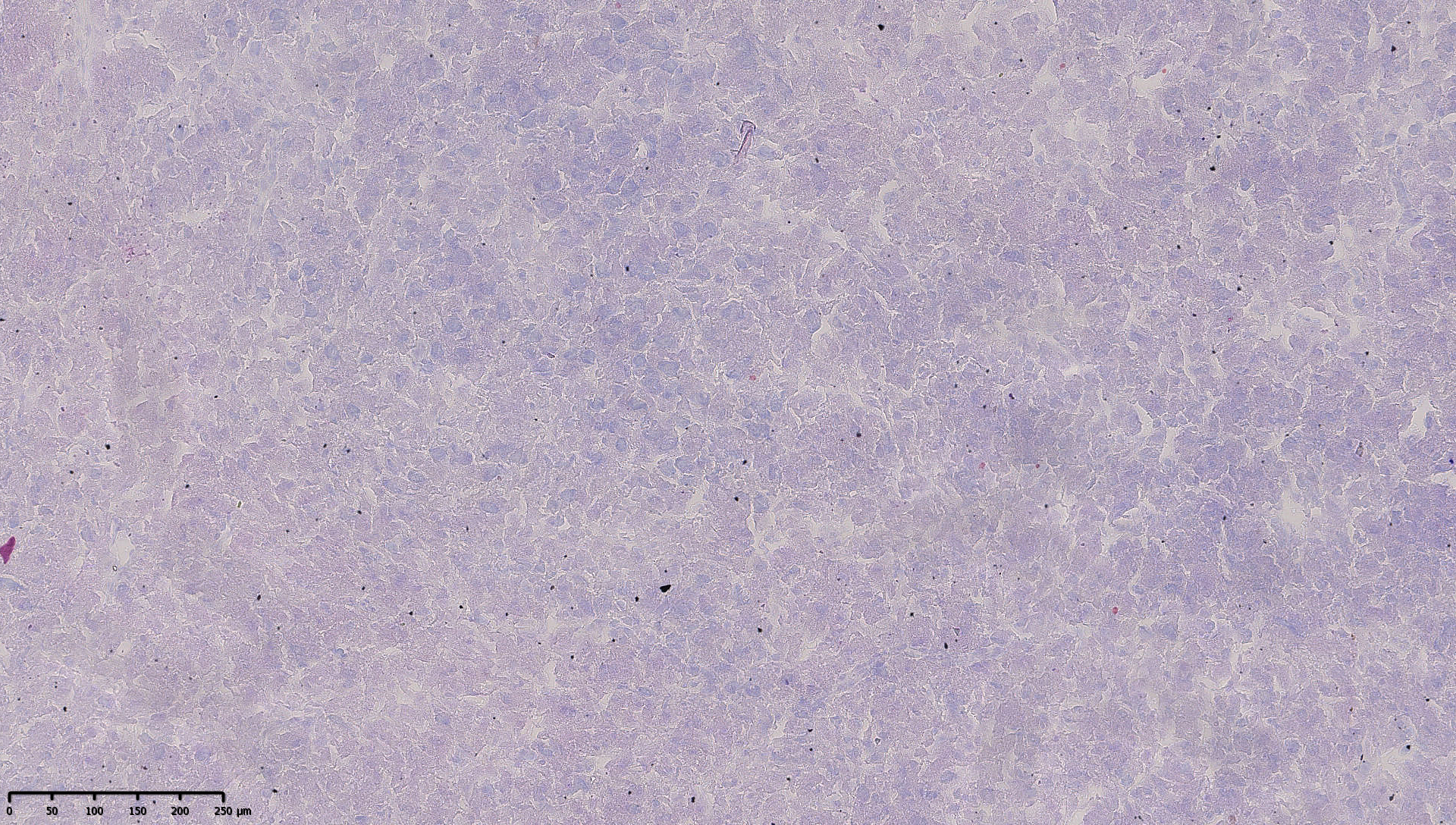

Supplement: Supplementary material — Original Images for Fig 6.zip [file IDRD_A_2585599_SM5409.zip › Original Image for Fig 6E (G4-right).tif]

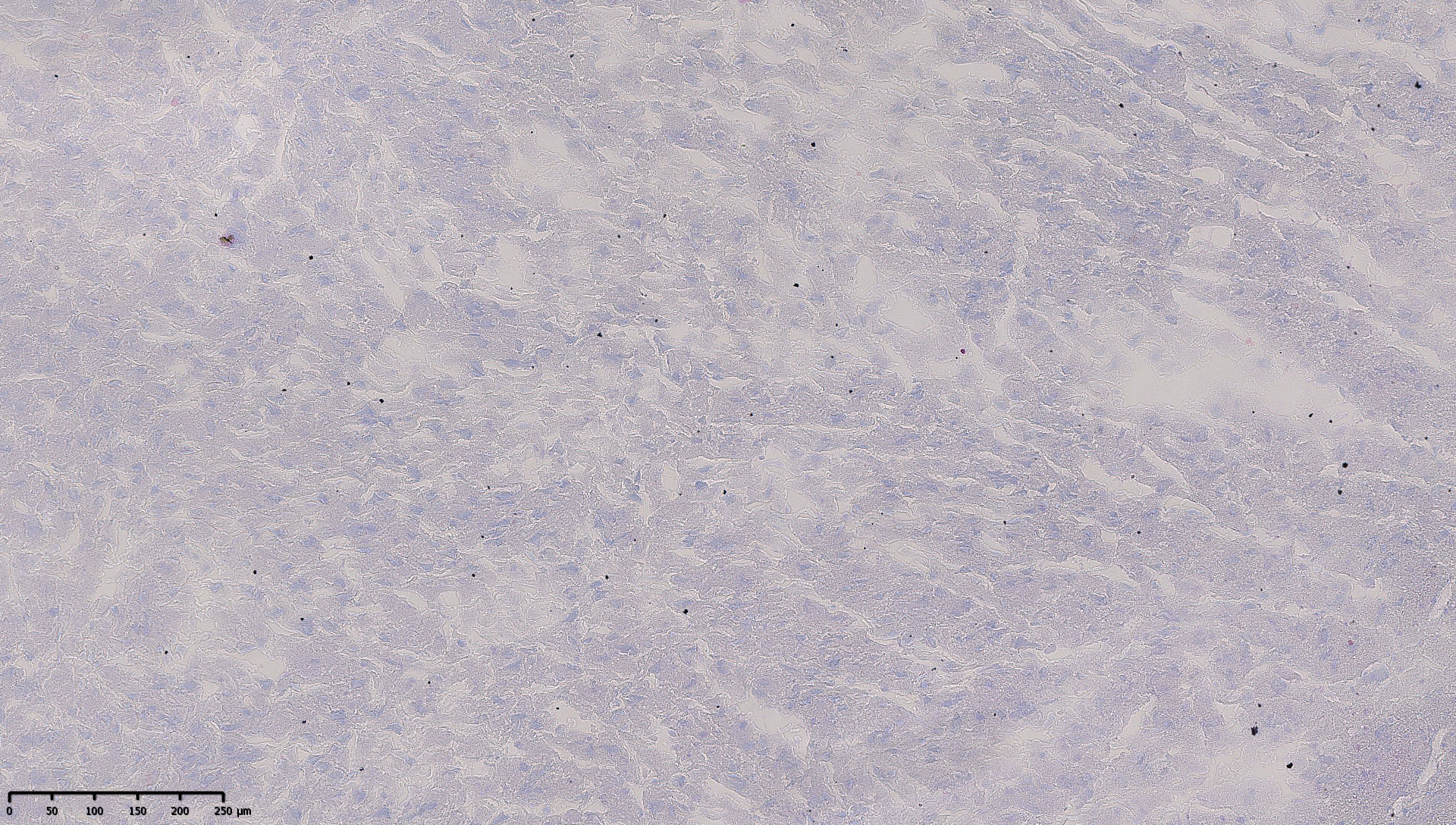

Supplement: Supplementary material — Original Images for Fig 6.zip [file IDRD_A_2585599_SM5409.zip › Original Image for Fig 6E (G5-left).tif]

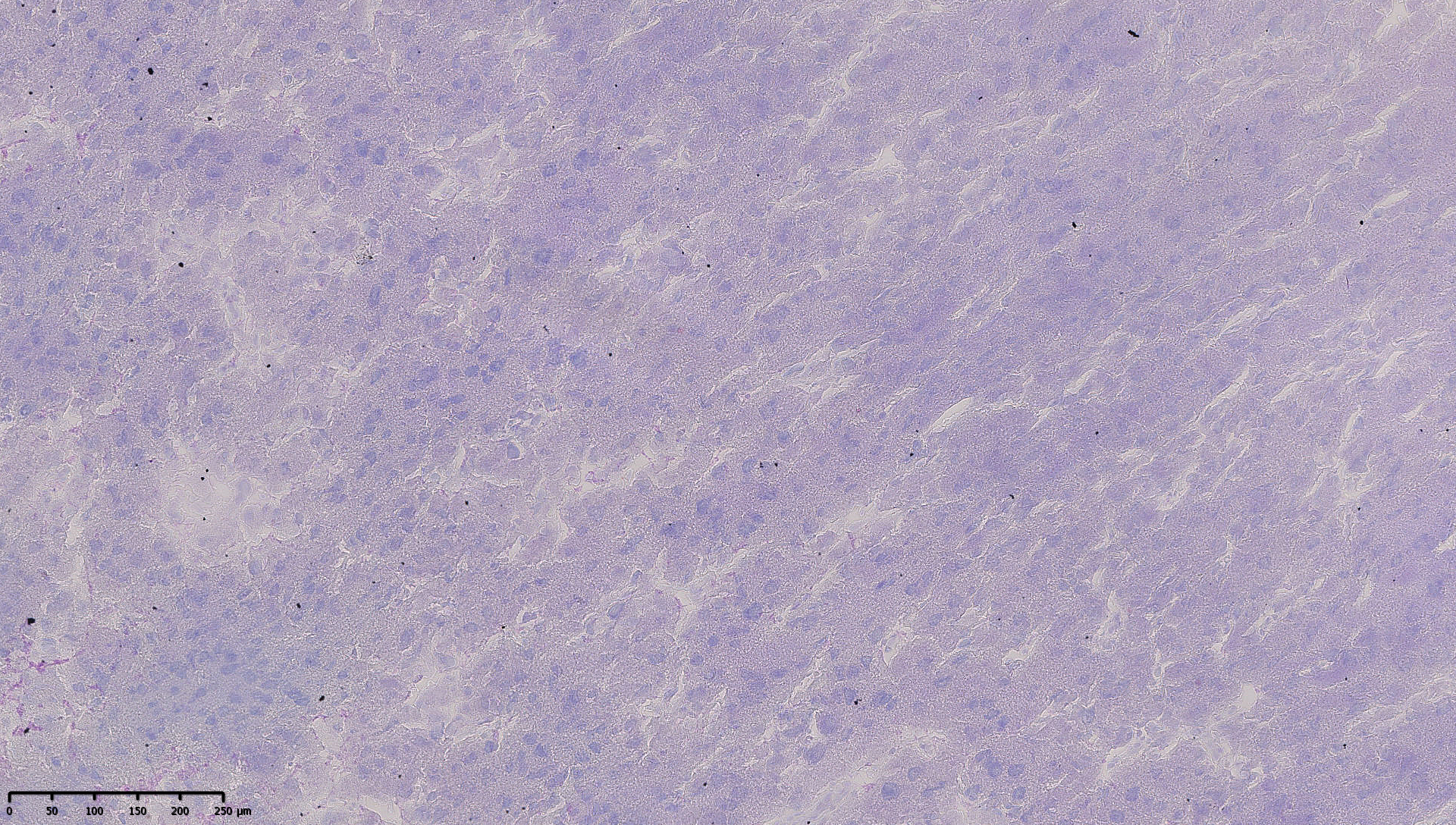

Supplement: Supplementary material — Original Images for Fig 6.zip [file IDRD_A_2585599_SM5409.zip › Original Image for Fig 6E (G5-right).tif]

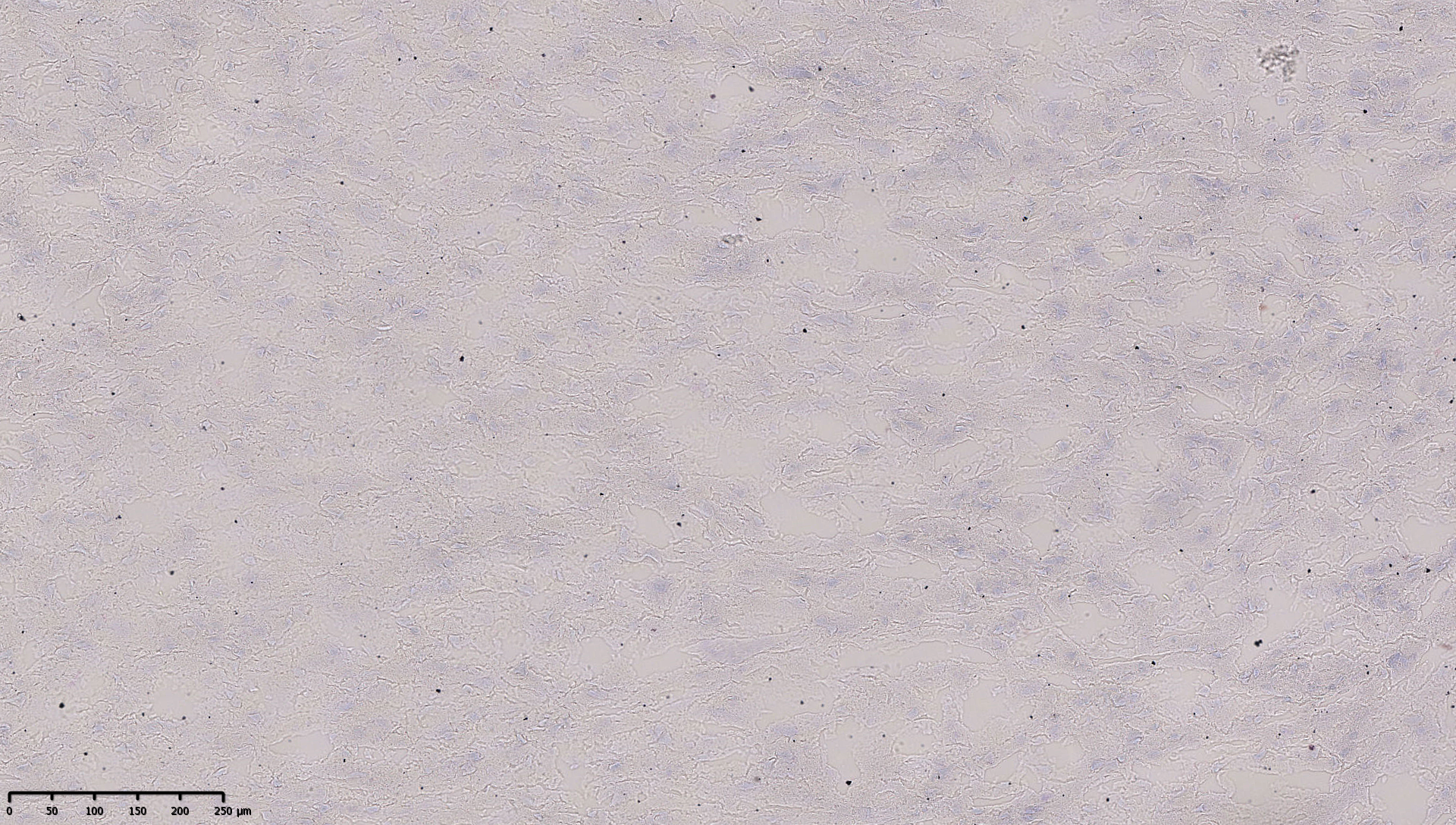

Supplement: Supplementary material — Original Images for Fig 6.zip [file IDRD_A_2585599_SM5409.zip › Original Image for Fig 6E (G6-left).tif]

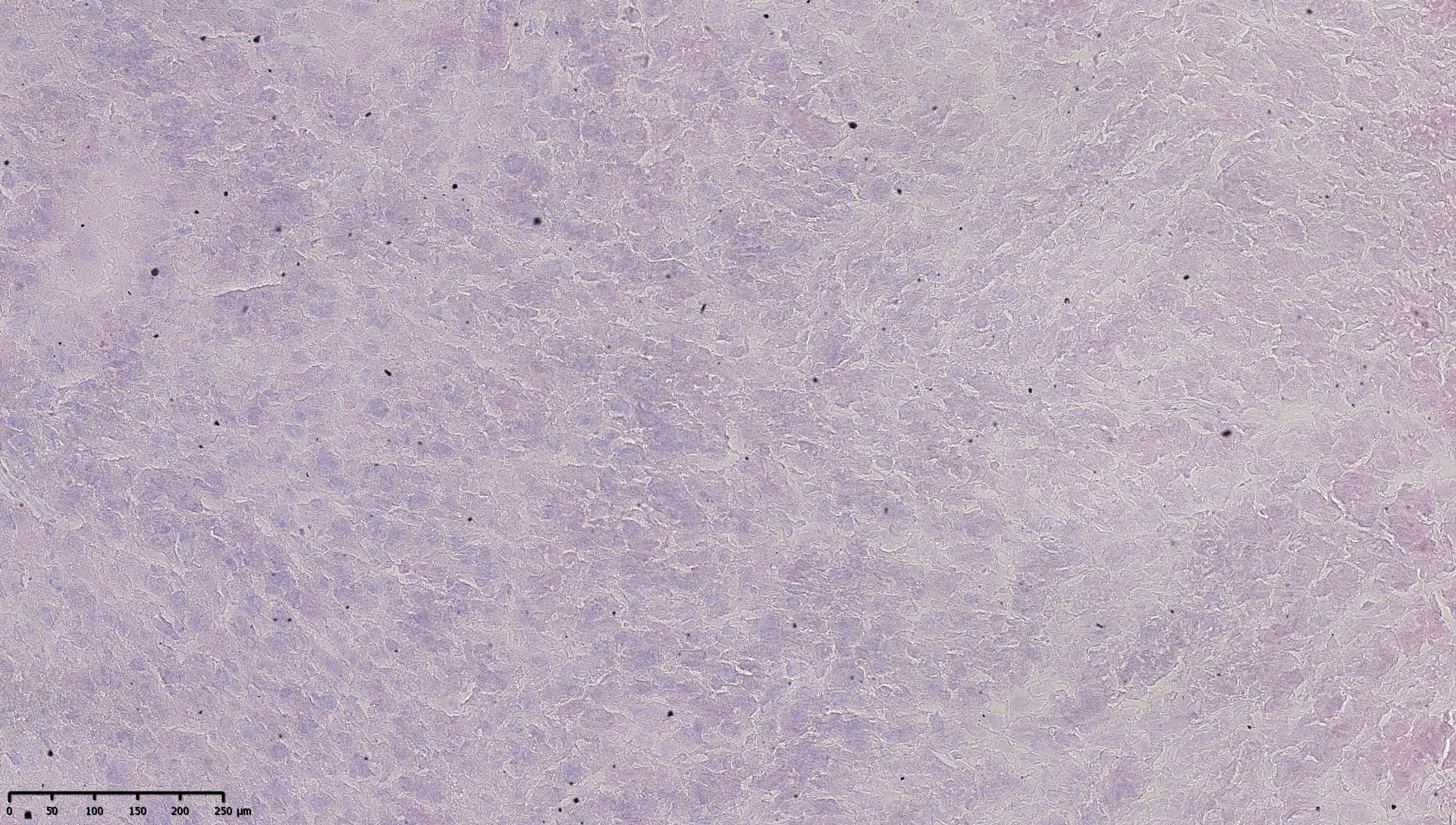

Supplement: Supplementary material — Original Images for Fig 6.zip [file IDRD_A_2585599_SM5409.zip › Original Image for Fig 6E (G6-right).tif]

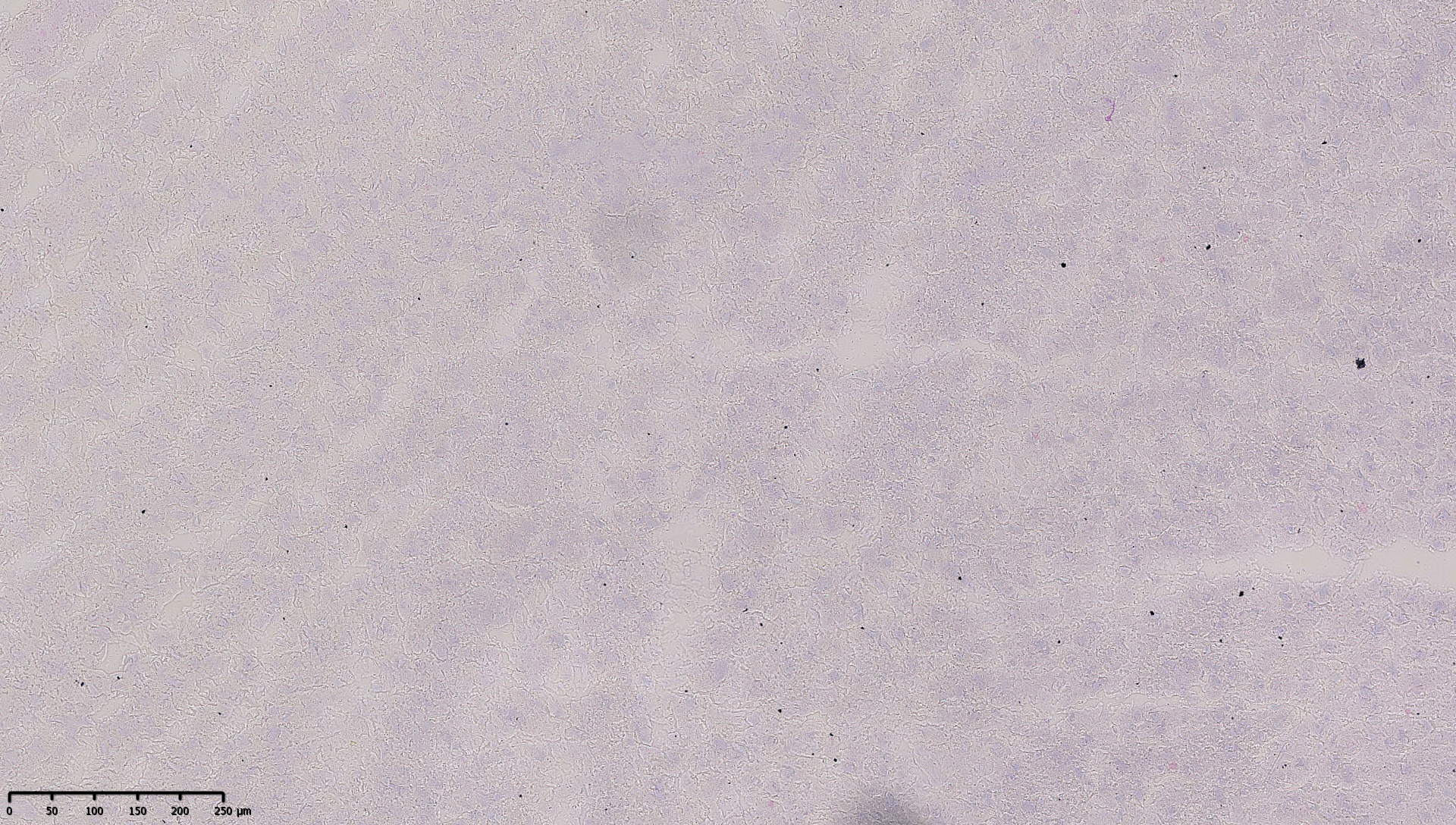

Supplement: Supplementary material — Original Images for Fig 6.zip [file IDRD_A_2585599_SM5409.zip › Original Image for Fig 6E (G7-left).tif]

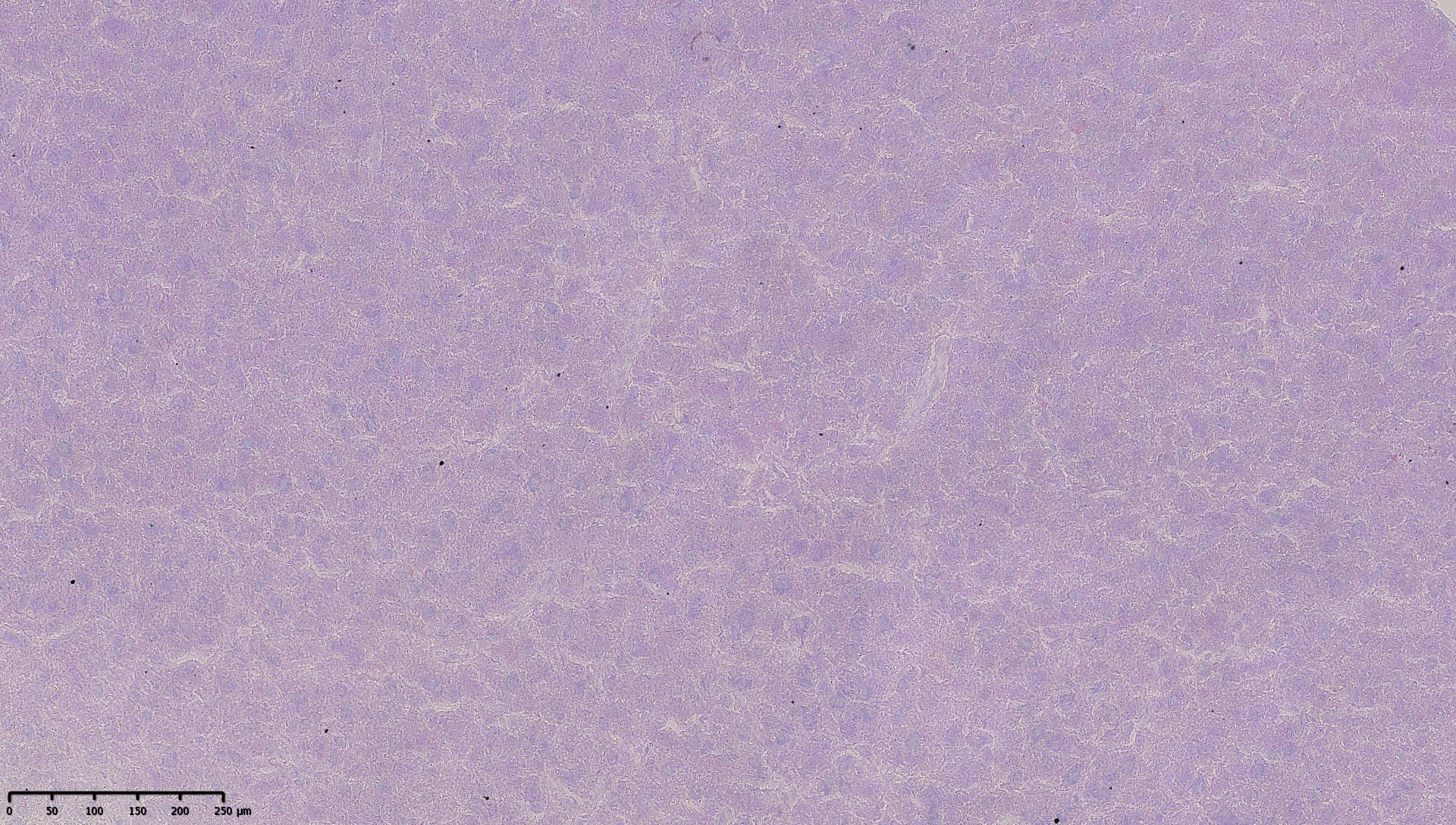

Supplement: Supplementary material — Original Images for Fig 6.zip [file IDRD_A_2585599_SM5409.zip › Original Image for Fig 6E (G7-right).tif]

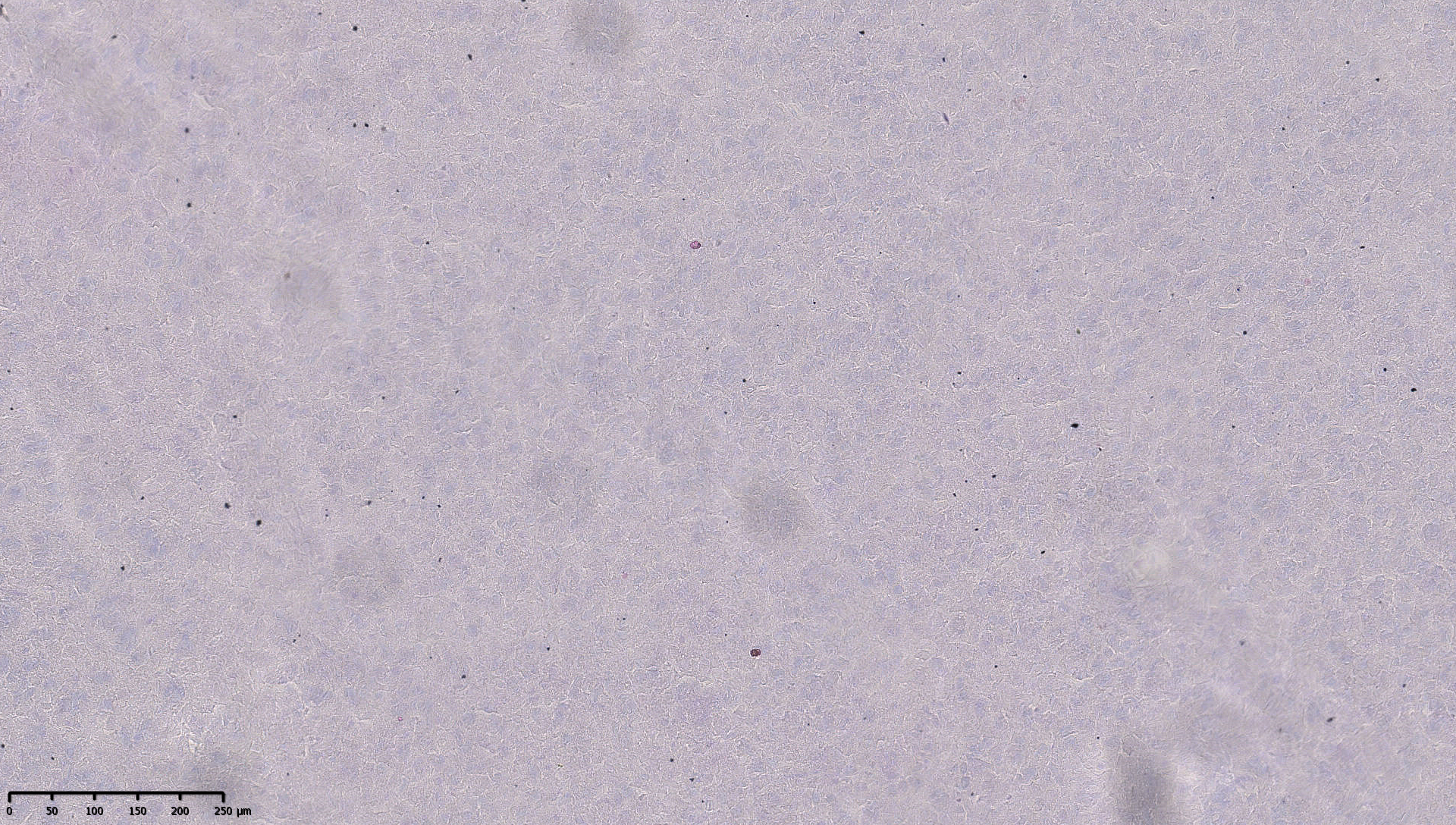

Supplement: Supplementary material — Original Images for Fig 6.zip [file IDRD_A_2585599_SM5409.zip › Original Image for Fig 6E (Sham-left).tif]

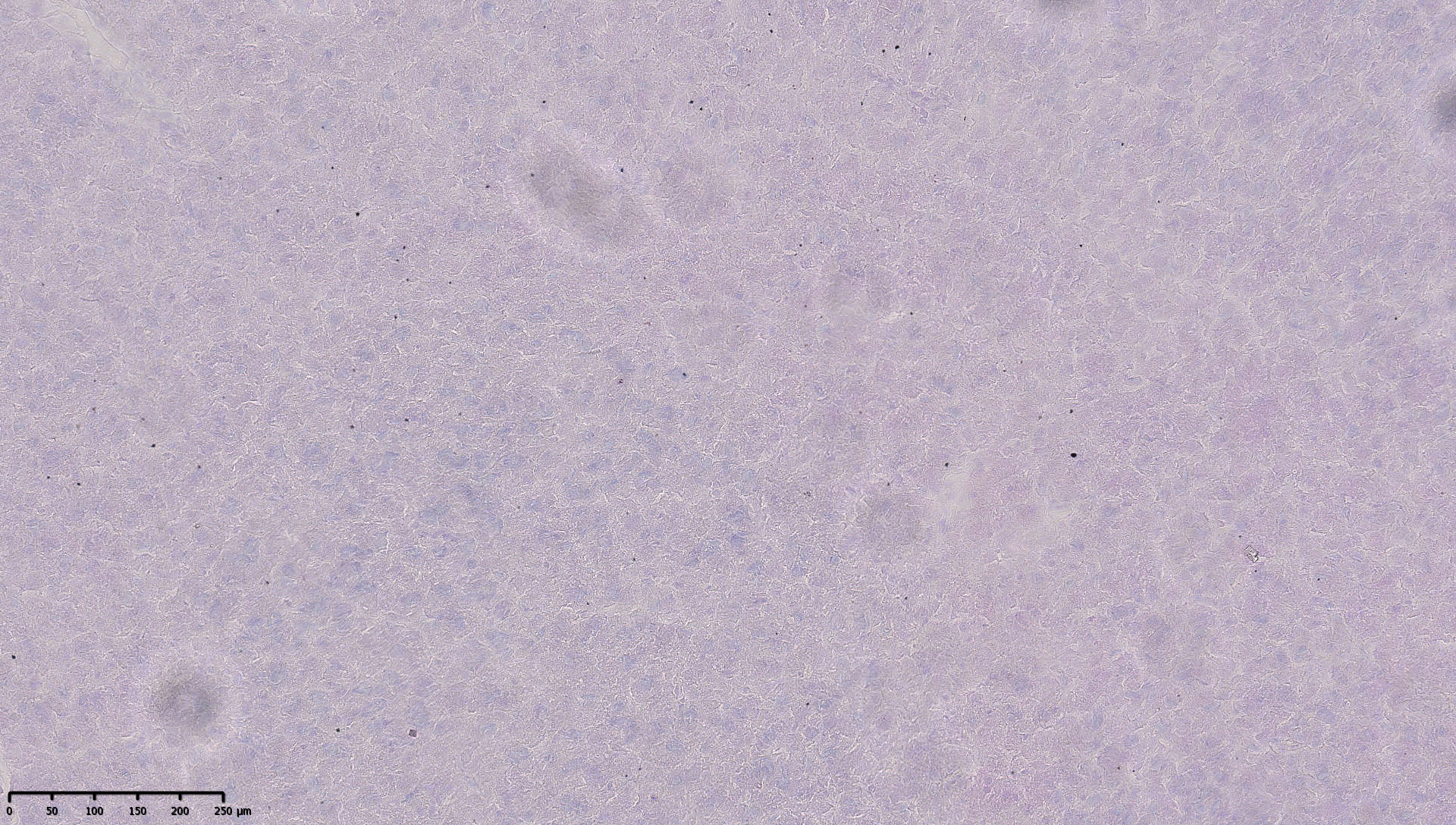

Supplement: Supplementary material — Original Images for Fig 6.zip [file IDRD_A_2585599_SM5409.zip › Original Image for Fig 6E (Sham-right).tif]

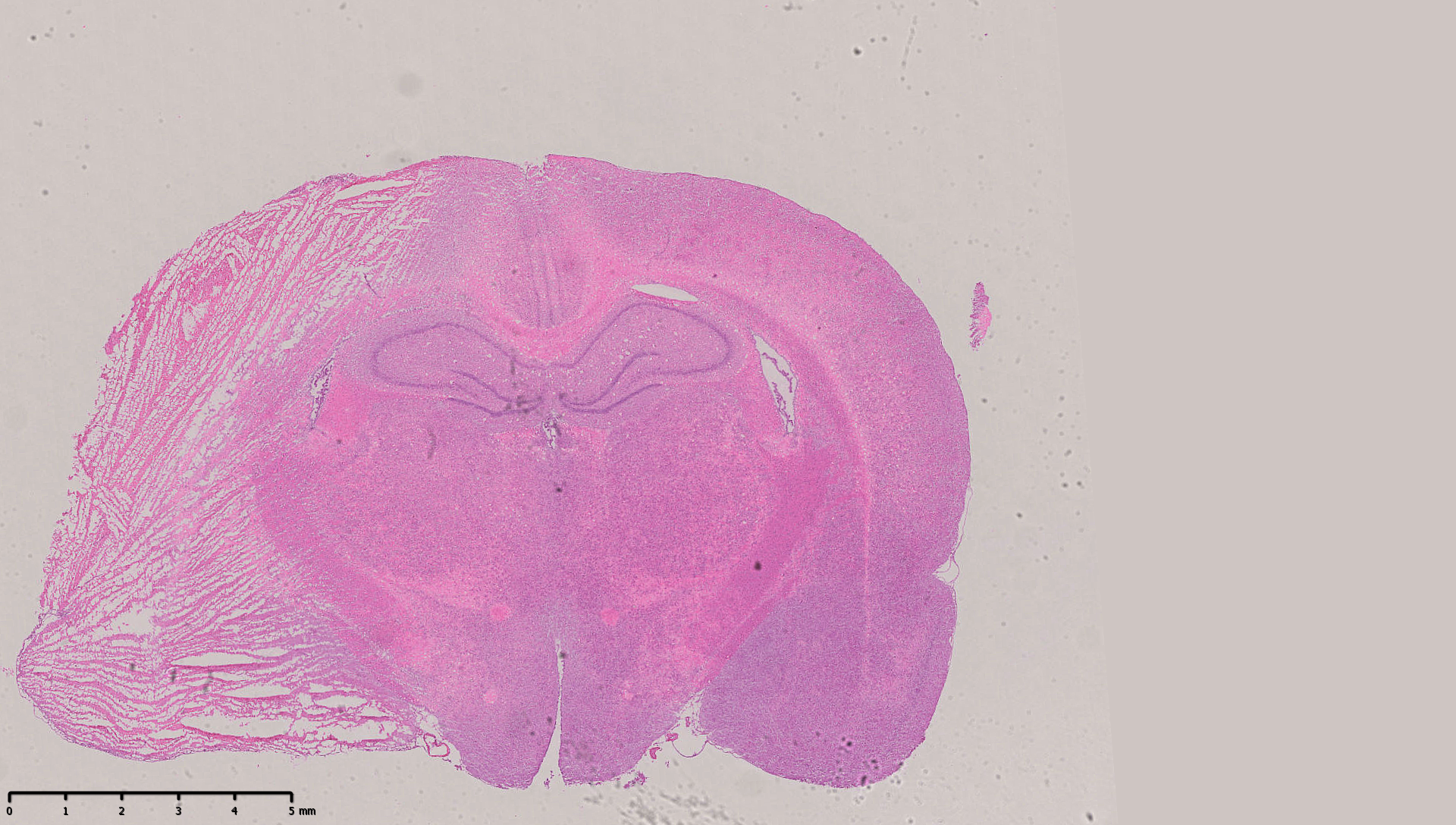

Supplement: Supplementary material — Original Images for Fig 6.zip [file IDRD_A_2585599_SM5409.zip › Original Image for Fig 6F (G1).tif]

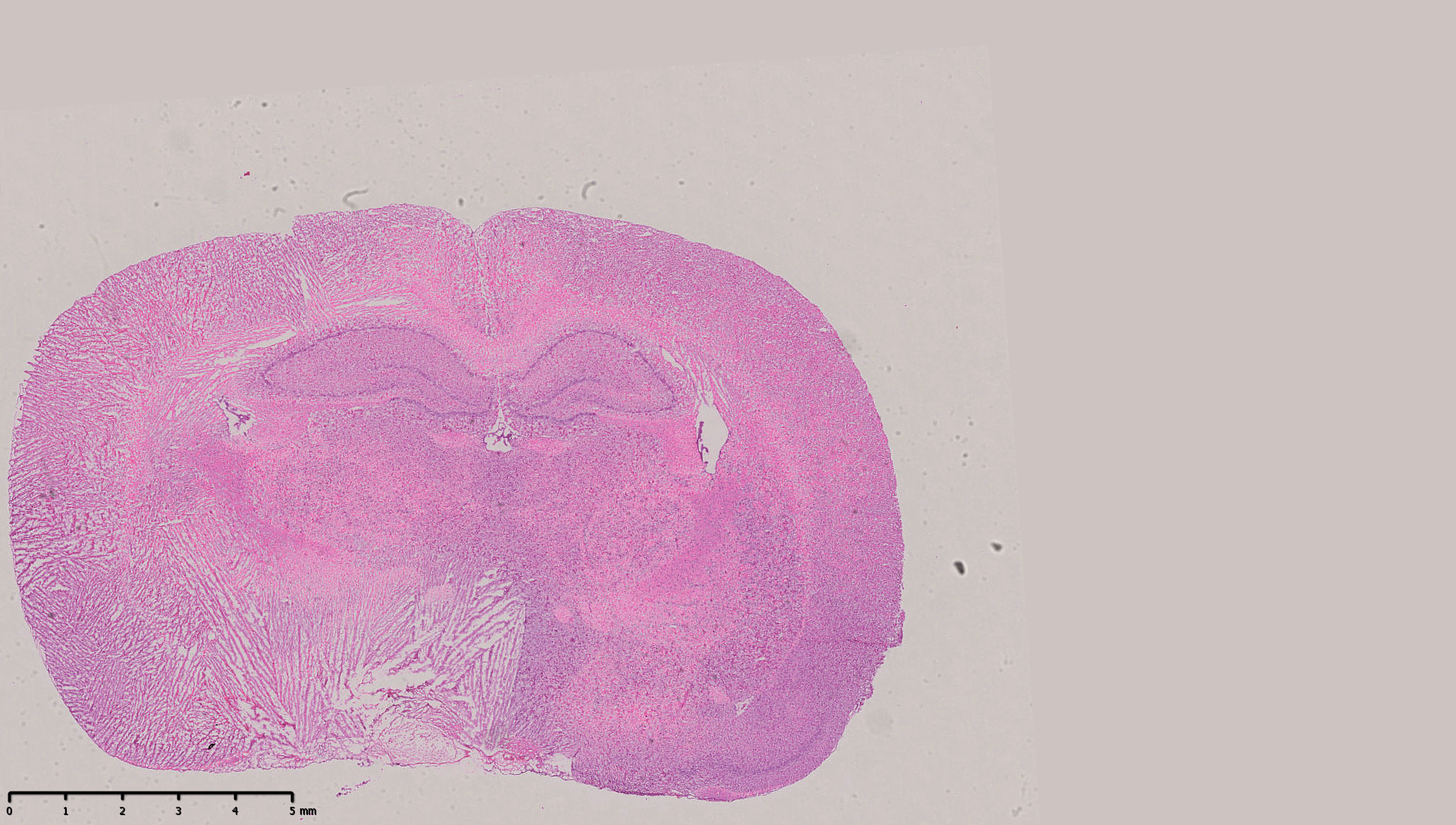

Supplement: Supplementary material — Original Images for Fig 6.zip [file IDRD_A_2585599_SM5409.zip › Original Image for Fig 6F (G2).tif]

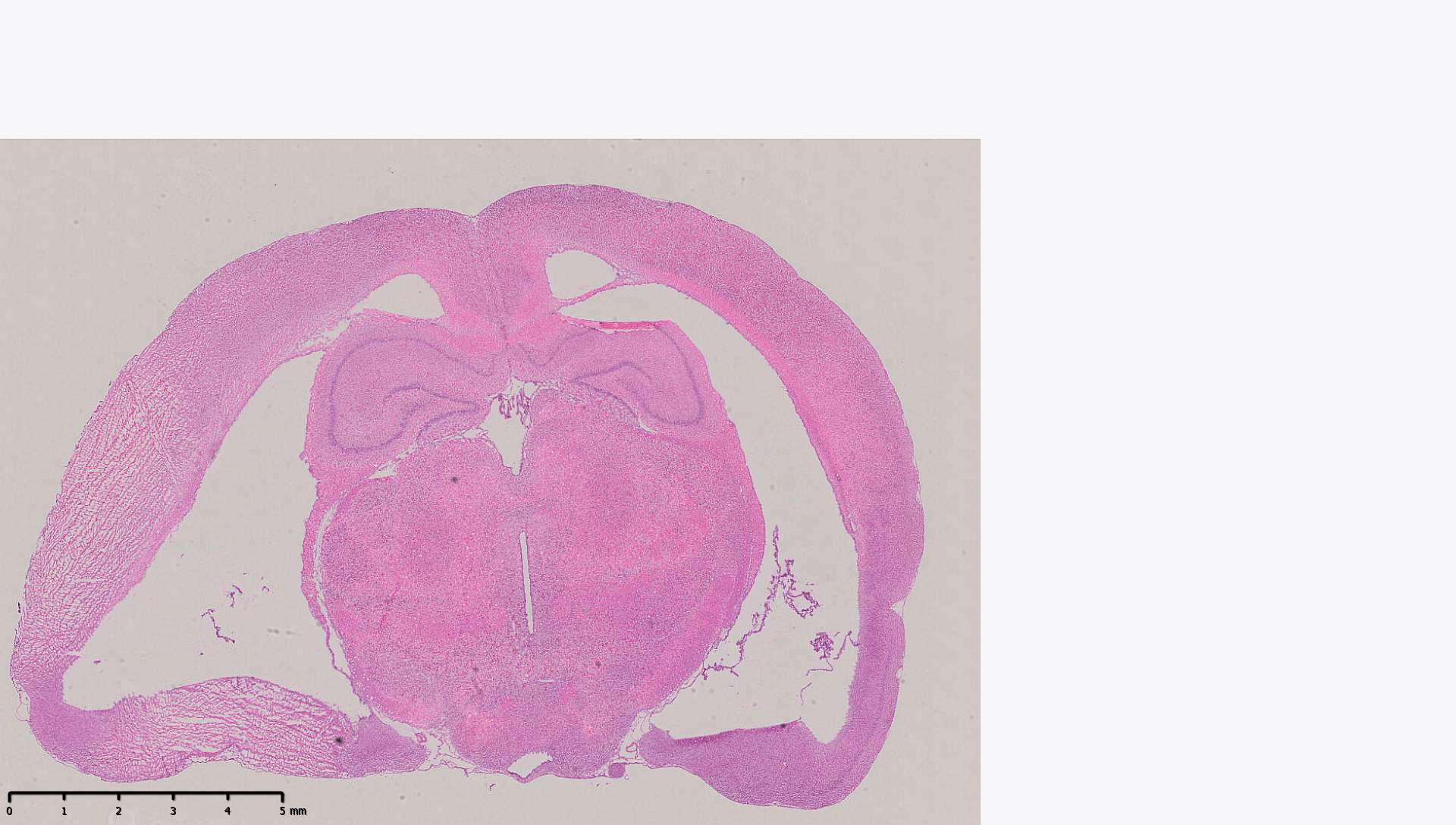

Supplement: Supplementary material — Original Images for Fig 6.zip [file IDRD_A_2585599_SM5409.zip › Original Image for Fig 6F (G3).tif]

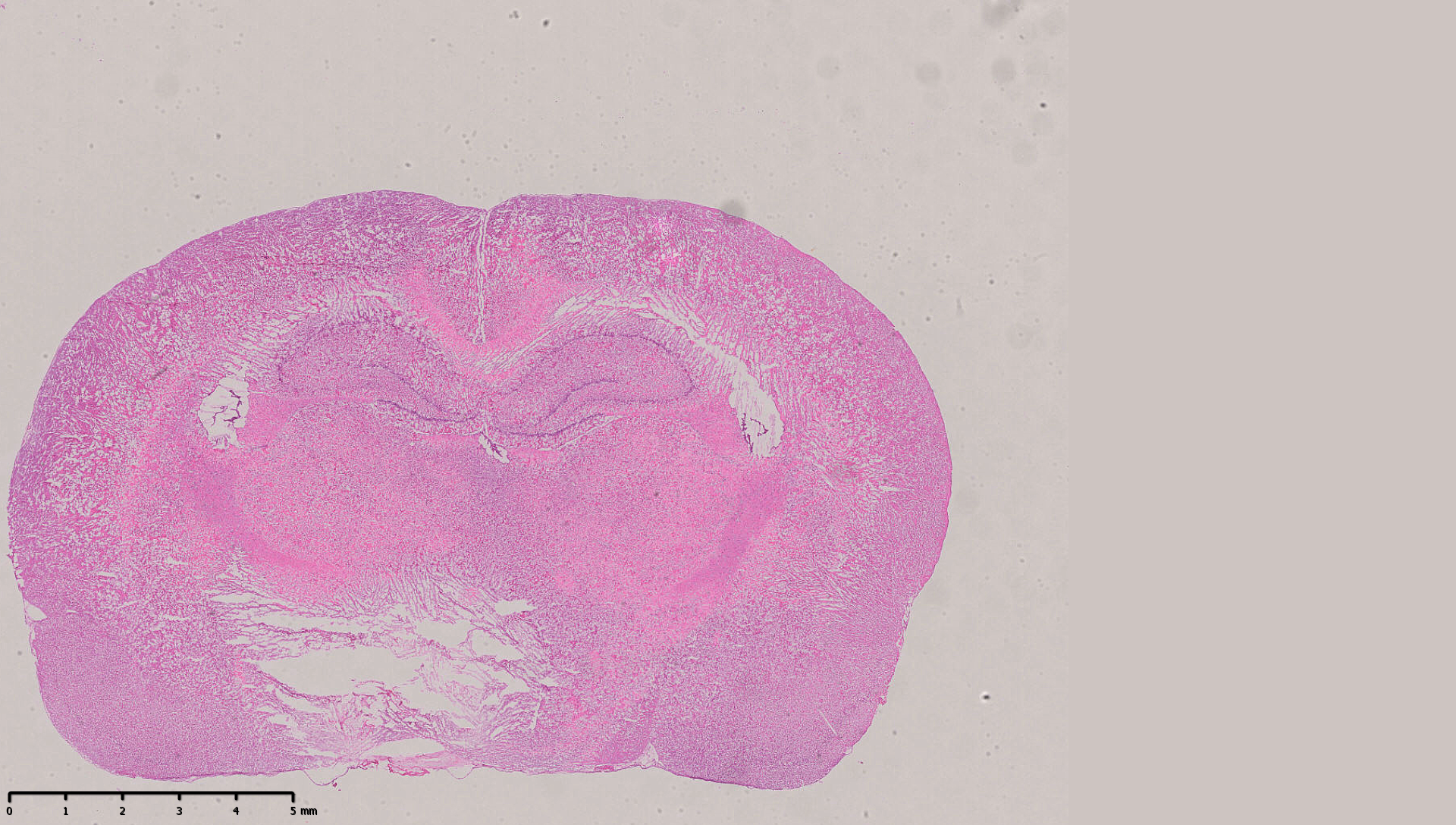

Supplement: Supplementary material — Original Images for Fig 6.zip [file IDRD_A_2585599_SM5409.zip › Original Image for Fig 6F (G4).tif]

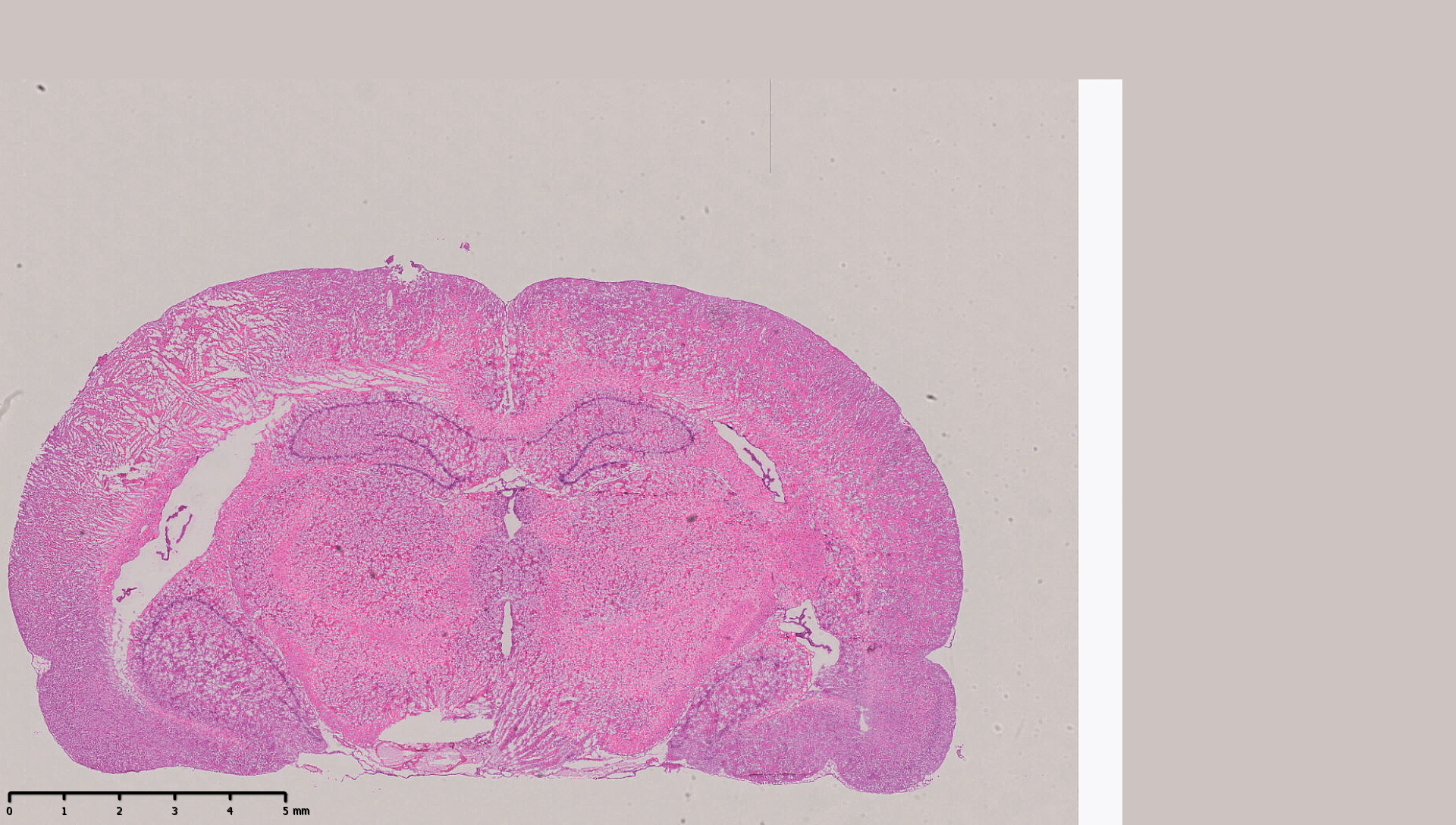

Supplement: Supplementary material — Original Images for Fig 6.zip [file IDRD_A_2585599_SM5409.zip › Original Image for Fig 6F (G5).tif]

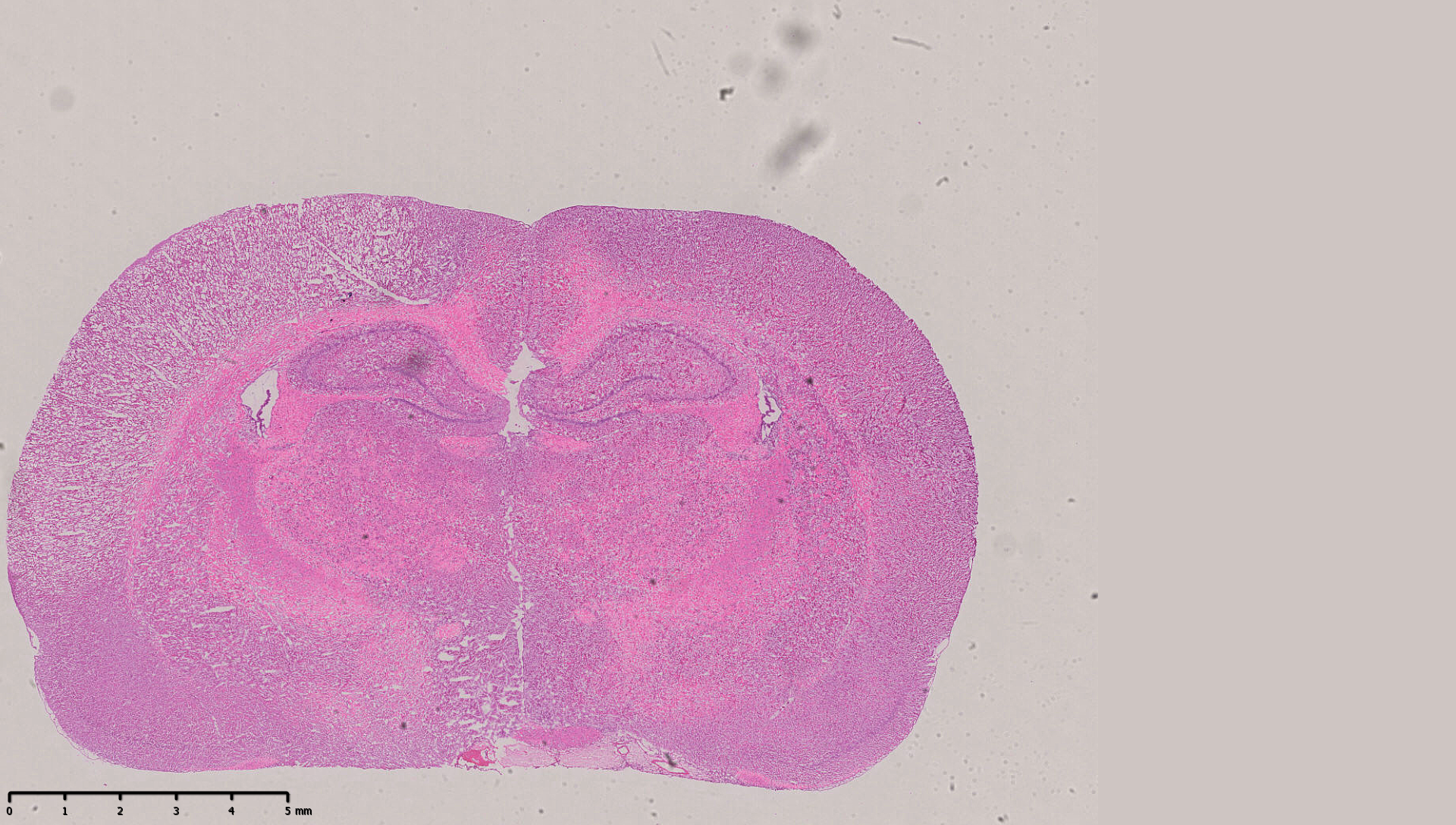

Supplement: Supplementary material — Original Images for Fig 6.zip [file IDRD_A_2585599_SM5409.zip › Original Image for Fig 6F (G6).tif]

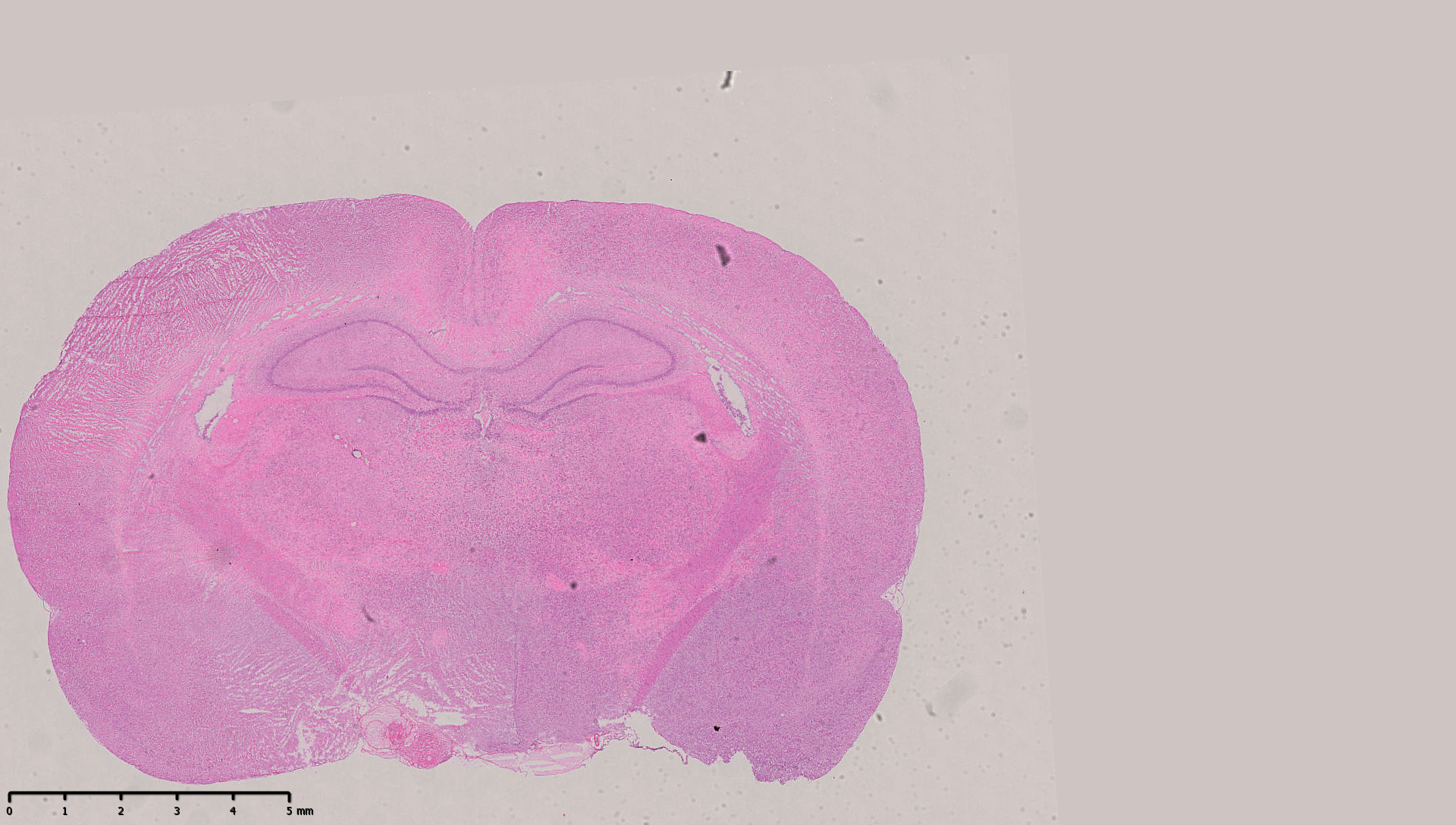

Supplement: Supplementary material — Original Images for Fig 6.zip [file IDRD_A_2585599_SM5409.zip › Original Image for Fig 6F (G7).tif]

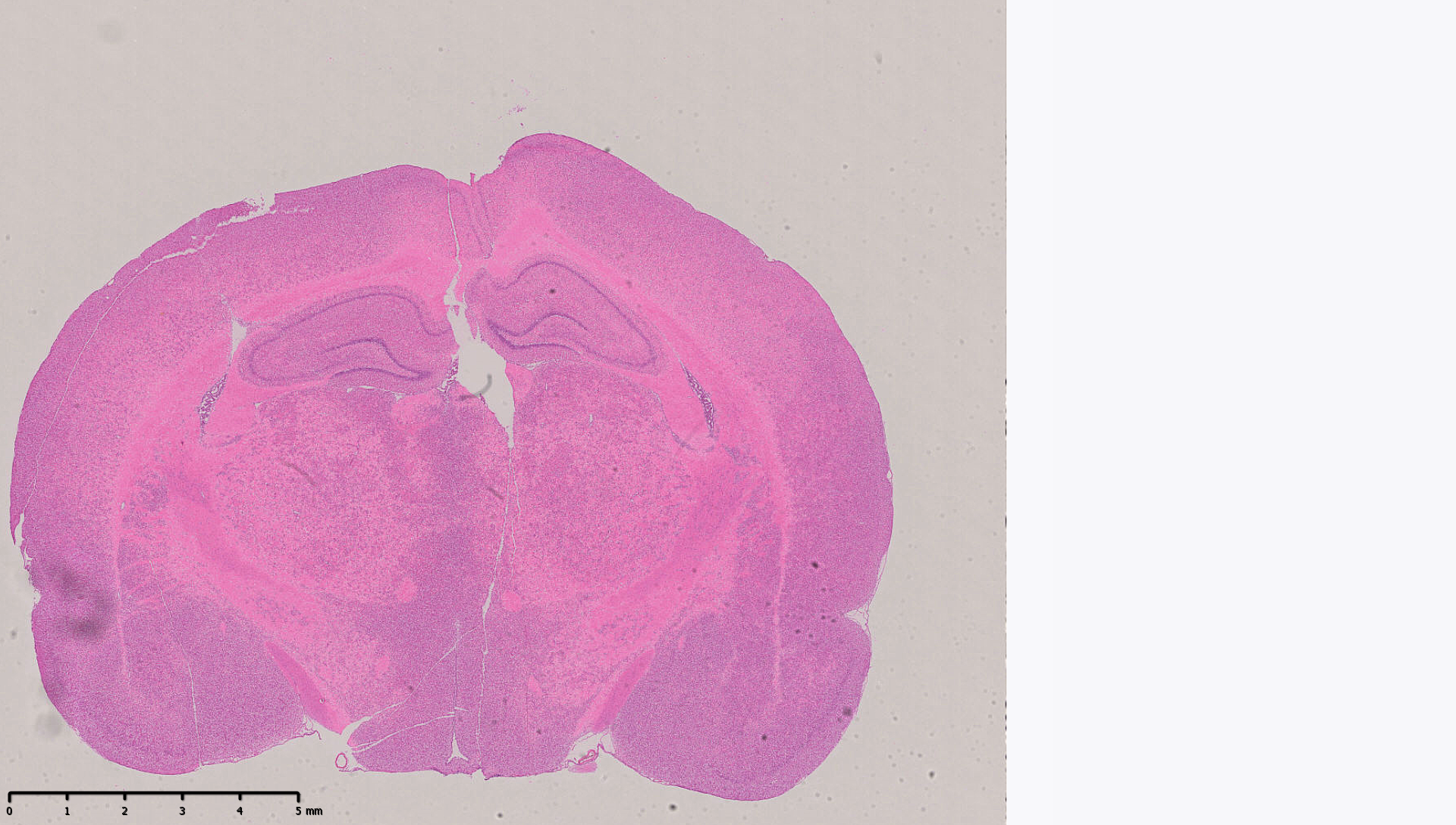

Supplement: Supplementary material — Original Images for Fig 6.zip [file IDRD_A_2585599_SM5409.zip › Original Image for Fig 6F (Sham).tif]

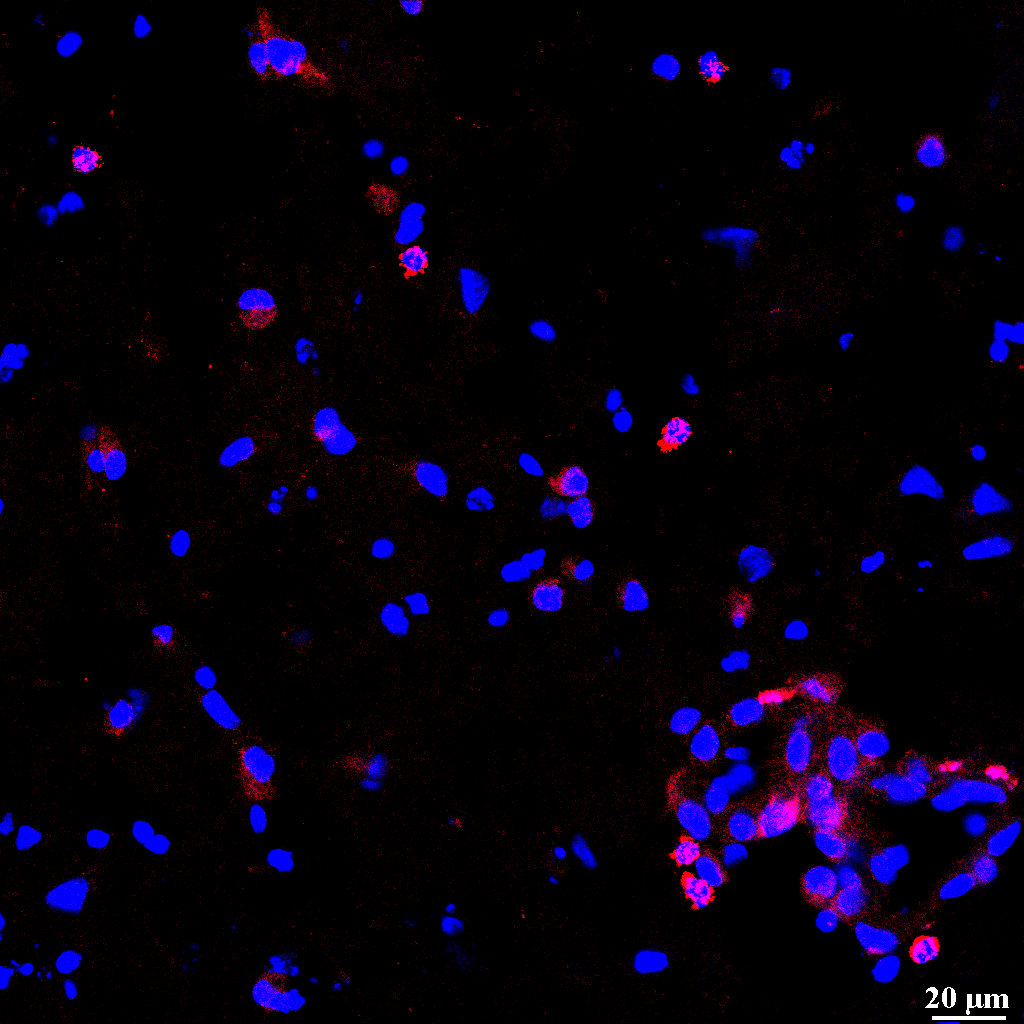

Supplement: Supplementary material — Original Images for Fig 6.zip [file IDRD_A_2585599_SM5409.zip › Original Image for Fig 6G control.tif]

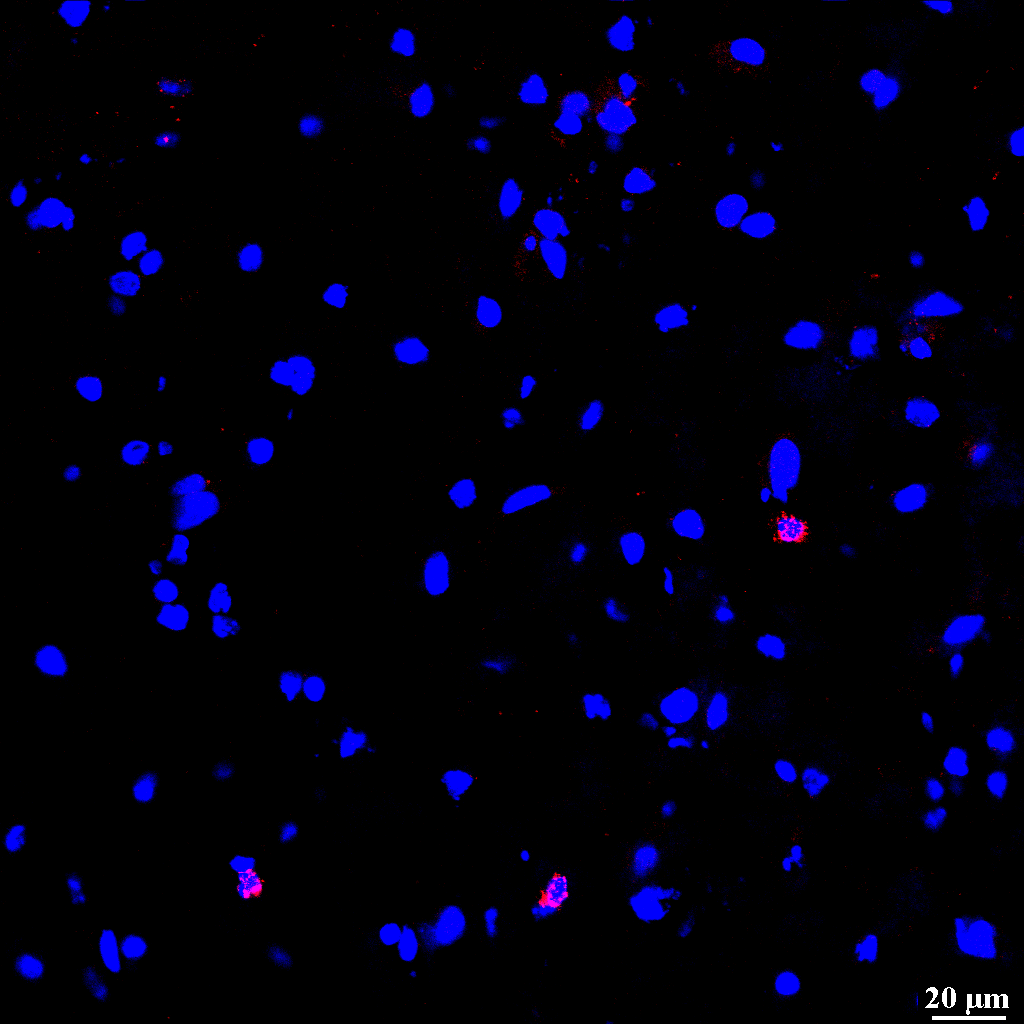

Supplement: Supplementary material — Original Images for Fig 6.zip [file IDRD_A_2585599_SM5409.zip › Original Image for Fig 6G Tid@PLTM.tif]

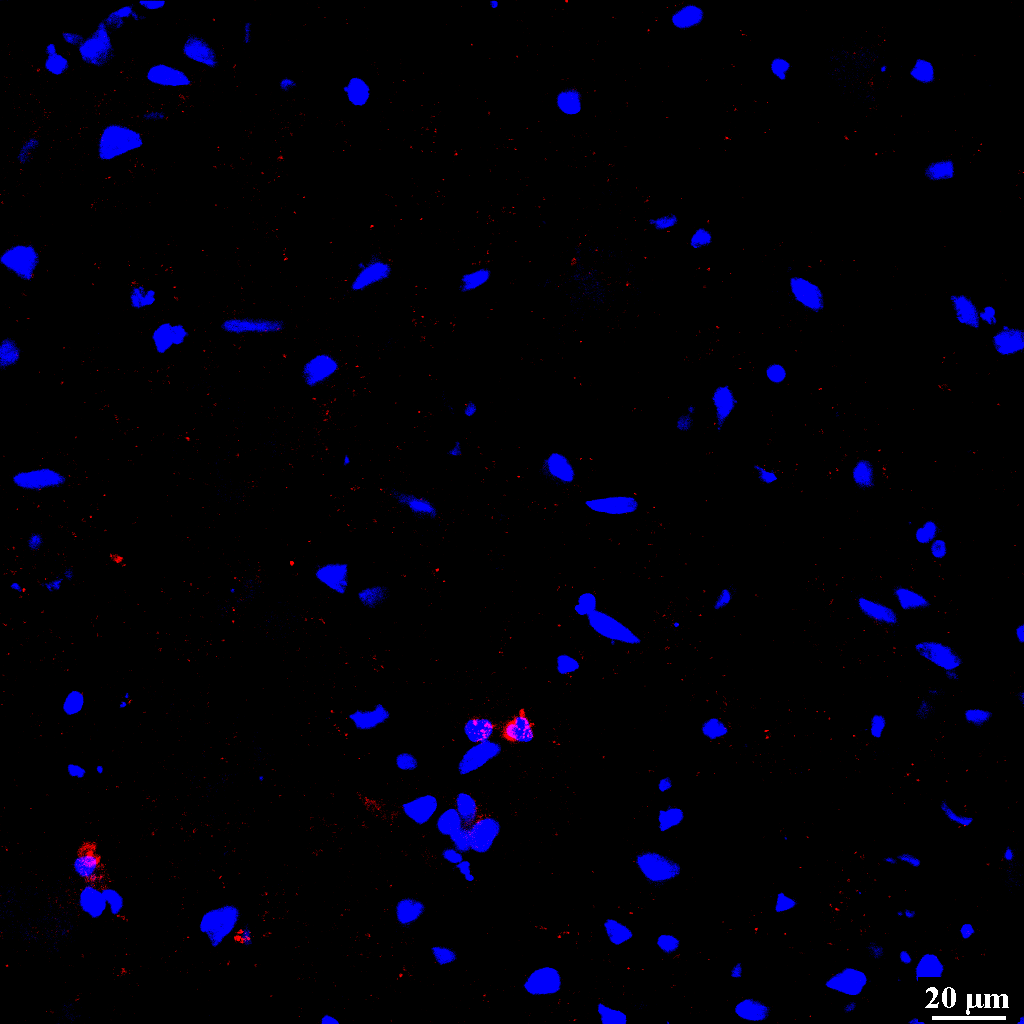

Supplement: Supplementary material — Original Images for Fig 6.zip [file IDRD_A_2585599_SM5409.zip › Original Image for Fig 6H control.tif]

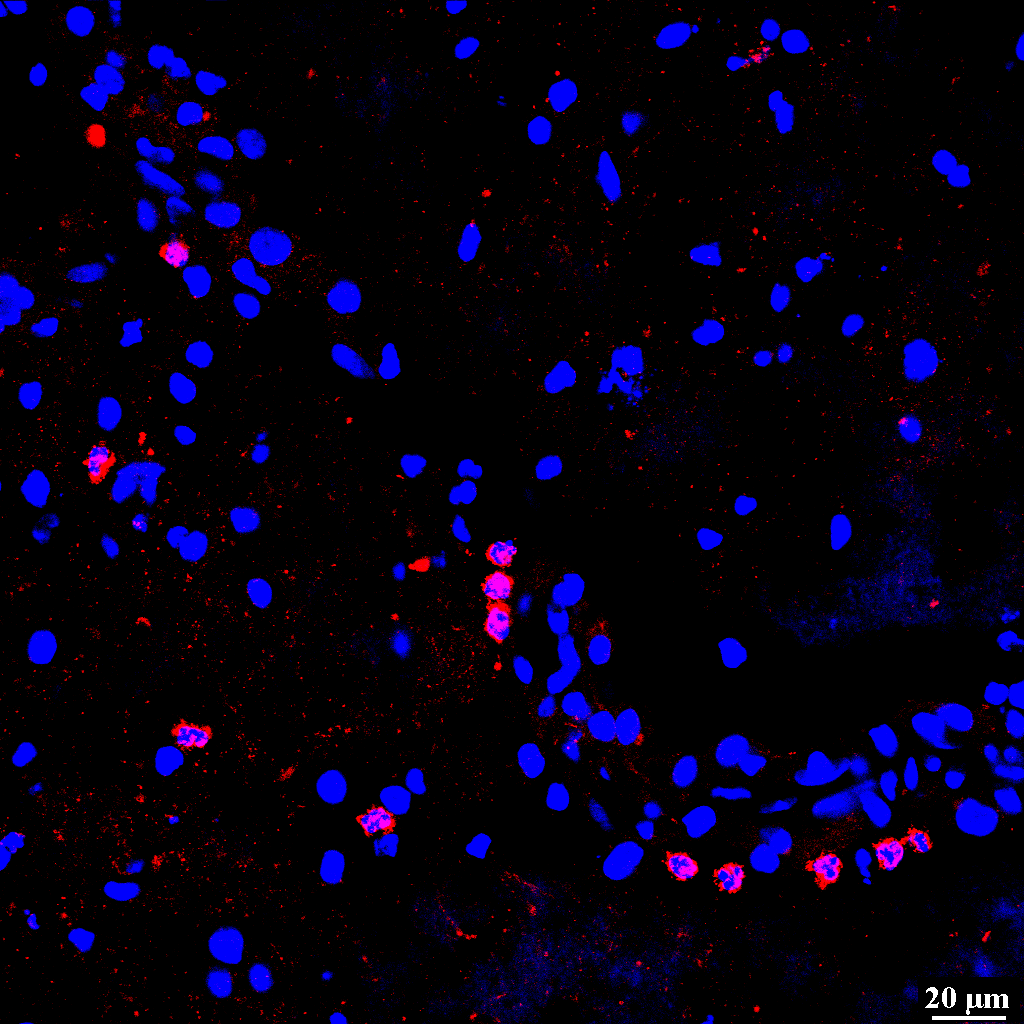

Supplement: Supplementary material — Original Images for Fig 6.zip [file IDRD_A_2585599_SM5409.zip › Original Image for Fig 6H Tid@PLTM.tif]
